# Changing the eligibility criteria for welfare payments at the end of life – a budget impact analysis for England and Wales

**DOI:** 10.1186/s12913-021-06390-8

**Published:** 2021-05-04

**Authors:** Edward J. D. Webb, David Meads, Clare Gardiner

**Affiliations:** 1 Leeds Institute of Health Sciences, University of Leeds, Clarendon Way, Leeds, LS2 9NL UK; 2 Health Sciences School, The University of Sheffield, Barber House Annexe, 387 Glossop Road, Sheffield, S10 2HQ UK

## Abstract

**Background:**

Terminal illness can cause a financial burden for many households. In England and Wales, fast-track access to welfare payments is available through special rules for the terminally ill (SRTI). Individuals are eligible for SRTI if they are judged to have 6 months or less to live. This criterion has been criticised as lacking a clinical basis, and being unfair for people with conditions where life-expectancy is difficult to accurately assess.

**Aim:**

To conduct a budget impact analysis on the possible increase in expenditure of personal independence payments (PIP) following a change in England and Wales to SRTI so that everyone with a terminal illness is eligible.

**Methods:**

The fraction of individuals with a given long-term condition was estimated by combining data from the Health Survey for England, the Office for National Statistics (ONS) and the Department for Work and Pensions. Logistic growth modelling and ONS population projections were used to project PIP expenditure from 2020 to 2025. The increased expenditure was calculated for hypothetical scenarios which may occur following an SRTI regime change, specifically an increase of 1, 2 and 3 percentage points in the fraction of individuals claiming PIP under SRTI. Data from the literature on the projected prevalence of mild, moderate and severe dementia was used to calculate the cost if everyone with a given severity of dementia claimed PIP under SRTI.

**Results:**

Under the current SRTI regime, PIP expenditure under SRTI was projected to increase from £0.231bn in 2020 to £0.260bn in 2025, compared to equivalent figures of £11.1bn and £12.7bn under non-SRTI. Expenditure in 2025 following an increase in the fraction claiming of 1, 2 and 3 percentage points was projected to be £1.1bn, £1.9bn and £2.7bn respectively. In 2025, PIP expenditure was estimated to be £7.4bn if everyone with dementia claimed under SRTI, compared to £6.4bn if only individuals with moderate and severe dementia claimed, and £4.7bn if only individuals with severe dementia claimed.

**Conclusion:**

Changes in SRTI are projected to lead to increases in PIP expenditure. However, the increased cost is small compared to expenditure under non-SRTI, especially as the highest costs were associated with extreme scenarios.

**Supplementary Information:**

The online version contains supplementary material available at 10.1186/s12913-021-06390-8.

## Background

Terminal illness presents a financial challenge to many households across the UK. Illness may increase household expenses directly with the costs of travel and medicines, or indirectly by limiting the amount of paid work that can be done by a patient or by their family carer [1–3]. The total cost of living with a terminal illness in the UK has been estimated to be between £12,000 and £16,000 per year, with up to 60% of people living with terminal illness dependent on benefits as their main source of income [4]. Families who are socio-economically disadvantaged are often worst hit and can spend large fractions of their income on the added costs brought on by terminal illness, leading to savings being depleted [4]. Financial difficulties in terminal illness have been linked to various negative outcomes for the person with the illness as well as their caregivers and family members. These negative outcomes can include worse mental and physical health, difficulties with coping and family conflict, poor bereavement outcomes and ongoing debt [2, 5, 6].

Whilst financial support does exist in many countries, patients and their carers report difficulties understanding eligibility for benefits and navigating complex application systems whilst juggling the demands of illness, on-going treatments and caregiving [5, 7].. A study on terminal lung cancer patients’ perceptions of claiming state benefits in the UK reported that many people did not know that they could claim financial benefits and found claim forms complicated. Some people had to ‘struggle’ to obtain much-needed benefits to which they were entitled, and many were shocked by how hard it was to obtain the information needed to make claims [8]. Many benefits to which patients are entitled go unclaimed [9]. Research has also identified financial hardship and a need for more financial help for patients with other life limiting conditions such as heart failure and chronic obstructive pulmonary disease (COPD) [10, 11], where an unpredictable and prolonged disease trajectory can exacerbate financial burden at the end of life.

Currently, people who are terminally ill in England and Wales can have their application for certain benefits fast tracked under the Special Rules for Terminal Illness (SRTI) scheme. The SRTI scheme means that where there is a ‘reasonable expectation of death within 6 months’ a person can have their benefit claim fast tracked and will automatically receive the higher benefit rate (https://www.gov.uk/terminal-illness-benefits). However, the 6 months eligibility rule is problematic, as many people with terminal conditions have unpredictable disease trajectories, where it is difficult to know at what point someone is within 6 months of death. This applies to many different conditions including cancer, but is a particular issue for people with non-cancer conditions where accurate prognostication is notoriously difficult [4, 12]. This includes motor neurone disease (MND), COPD, heart failure, stroke, dementia and many other conditions. The uncertainty around disease trajectories makes clinicians reluctant to confirm the eligibility of people with these conditions for the SRTI scheme [13]. The administrative burden and time delay in applying under non-SRTI means many people will die without receiving any benefits.

In 2020, a change in the SRTI scheme will come into effect in Scotland, with the 6 months eligibility criteria replaced by a clinician’s judgement that an individual has a progressive disease that can reasonably be expected to cause death [14]. There are campaigns for similar changes to be enacted in England and Wales [13], which have led to the government in England announcing a review of the benefits system for terminally ill people. Such changes may lead to more people claiming, both due to increased eligibility as well as greater ease of demonstrating eligibility for people with unpredictable disease categories. Some people may also claim for longer due to accessing funds earlier than they would have otherwise. One important question is the potential increase in public expenditure caused by a change to the SRTI eligibility criteria, as too great a cost would make such a policy change unattractive.

This article attempts to inform policy by projecting the scale of, and possible increase in, public expenditure in England and Wales on one particular benefit following a change to the SRTI scheme. In addition, a case study was conducted in the area of dementia. As dementia is rapidly growing in prevalence [15], a policy concern is that changing the SRTI regime may result in a large number of people with dementia being eligible for many years. The case study examined the maximum possible expenditure if all people with mild, moderate and severe dementia claimed under SRTI.

The SRTI scheme applies to a range of benefits including Personal Independence Payment (PIP), Disability Living Allowance (DLA), Attendance Allowance (AA), Employment Support Allowance (ESA) and Universal Credit (UC) .A detailed analysis of all benefits included in the SRTI scheme is beyond the scope of this paper. Thus we chose to focus only on PIP. One reason for this is that the available data on PIP claims made predictions easier to estimate compared to other benefits (see the discussion for more details). Expenditure on PIP is also larger than most other benefits covered by SRTI, with payments in England and Wales totalling £9.5 million in 2018/19, compared to £7.2 billion for DL, £5.2 billion for AA, and £7.3 billion for UC. Only ESA had greater expenditure, at £13.3 billion.^1^ The reader should bear in mind that our results should be seen in the context of PIP being one part of a wider landscape of available benefits.

## Methods

PIP is a welfare benefit which aims to help people with long-term conditions with their cost of living. It has two components, mobility and daily living, each of which can be paid at a standard or enhanced rate. In 2020, the standard and enhanced rates were £23.60 and £62.25 per week respectively for mobility and £59.70 and £89.15 per week respectively for daily living. Eligibility is based primarily on a condition’s practical effect on an individual’s life. Individuals must also be aged 16 or over and either be under State Pension age or to have been claiming prior to reaching State Pension age. There are no eligibility requirements based on income, assets or employment data. PIP was introduced as a replacement to disability living allowance in 2013 with a gradual rollout. As of 2019, around 3.1 million people receive PIP, of which around 50,000 claim under SRTI.^2^

Expenditure was calculated in the following way: The number of people in England and Wales with a given condition was estimated. We then estimated the fraction of people with a given condition claiming personal independence payments (PIP) under SRTI and normal rules (referred to hereafter as non-SRTI). Modelling was used to project how these fractions will change in the future, under ‘new rules’ for SRTI. This was combined with population projections to estimate the number of people claiming PIP, and hence the expected cost. To model alternative scenarios, the parameters of the models were changed to increase the fraction of individuals claiming and/or claims’ growth rate.

**Fig. 1:**
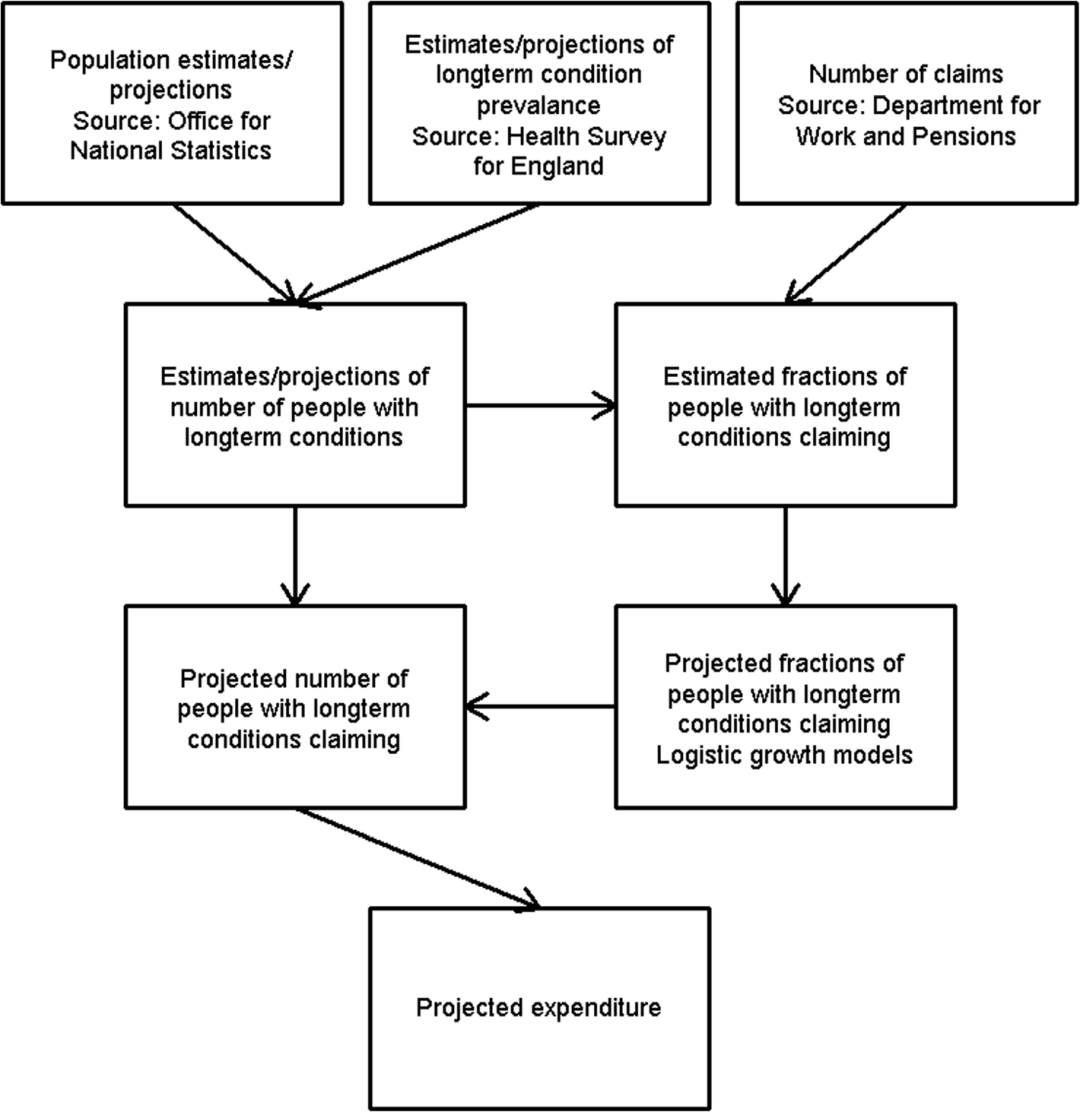
Flow diagram illustrating analysis strategy

A diagram illustrating the stages of analysis is given in Fig. 1.

### Data

#### Population estimates and projections

Midyear population estimates from 1995 to 2019 were obtained from the Office for National Statistics (ONS) [16], as were population projections from 2020 to 2025 [17].

#### Health survey for England

The Health Survey for England (HSE) is an annual large scale survey which gathers data from several thousand individuals about their lives and health. Data from the surveys from 1995 until 2018, the latest year available at the time of performing the analysis, were included [18–42]. In each survey, data was collected on whether individuals had a long-term condition, categorised according to the International Statistical Classification of Diseases and Related Health Problems, 10th edition (ICD-10) [43] at chapter level. ICD-10 chapter-level categories, including examples of conditions for each category, are given in Table 1.

**Table 1:** Chapter-level ICD-10 disease classifications

Code	Description	Example conditions
A00–B99	Certain infectious and parasitic diseases	TB, polio, HIV, mumps
C00–D48	Neoplasms	Cancers, benign tumours
D50–D89	Diseases of the blood and blood-forming organs and certain disorders involving the immune mechanism	Anemia, Haemophillia
E00–E90	Endocrine, nutritional and metabolic diseases	Diabetes, malnutrition, lactose intolerance
F00–F99	Mental and behavioural disorders	Dementia due to Alzheimers, schizophrenia
G00–G99	Diseases of the nervous system	Meningitis, MND, Parkinsons, Alzheimers
H00–H59	Diseases of the eye and adnexa	Conjunctivitis, cataracts, glaucoma
H60–H95	Diseases of the ear and mastoid process	Hearing loss
I00–I99	Diseases of the circulatory system	Angina, heart disease, varicose veins
J00–J99	Diseases of the respiratory system	Cold, sinitus, bronchitis
K00–K93	Diseases of the digestive system	Caries, ulcers, coeliac disease
L00–L99	Diseases of the skin and subcutaneous tissue	Impetigo, dermititis, psoriasis
M00–M99	Diseases of the musculoskeletal system and connective tissue	Arthritis, sciatica, osteoporosis
N00–N99	Diseases of the genitourinary system	Renal failure, urinary tract infections, infertility
O00–O99	Pregnancy, childbirth and the puerperium	Ectopic pregnancy, long/obstructed labour
P00–P96	Certain conditions originating in the perinatal period	Slow fetal growth, fetal blood loss
Q00–Q99	Congenital malformations, deformations and chromosomal abnormalities	Microcephaly, cleft palate, Down syndrome
R00–R99	Symptoms, signs and abnormal clinical and laboratory findings, not elsewhere classified	Gangrene, nausea, vomiting, chronic pain
S00–T98	Injury, poisoning and certain other consequences of external causes	Wounds, injuries, concussions
V01–Y98	External causes of morbidity and mortality	Accidents, self-harm, assault
Z00–Z99	Factors influencing health status and contact with health services	Examinations, vaccinations

#### Welfare claims

Data on the number and type of welfare claims was obtained from the Department for Work and Pensions’ (DWP) Stat-Xplore service.^3^ The PIP Cases with Entitlement dataset was used to extract the number of claims per month in England and Wales from April 2013, the first available time point, until January 2020. Claims were broken down by the rules under which they were made (non-SRTI/SRTI), type and levels of award, gender, age and disability categorised by chapter-level ICD-10 code.

Payment levels for each benefit were found from the DWP website.^4^ Payments increase in line with inflation, so that the current value of future payments in 2020 pounds will remain the same in future. This means that all results are expressed in 2020 prices.

#### Welfare expenditure

The Stat-Xplore dataset DWP Expenditure was used to obtain the total expenditure on PIP in each financial year from 2013/14 to 2018/19.

#### Case study: dementia

Data on the current and projected prevalence of dementia in England and Wales stratified by severity was obtained from Wittenberg et al. [15].

### Analysis

All analysis was performed in R.

#### Disease prevalence

The probability of an HSE respondent *i* having a given condition was estimated using a series of logit models of the form

$$ {p}_i(condition)={\beta}_0+{\beta}_t\frac{t_i}{w_i}+{\beta}_f\frac{female_i}{w_i}+{\boldsymbol{\beta}}_{\boldsymbol{i}}\frac{{\boldsymbol{age}}_{\boldsymbol{i}}}{w_i}+{\varepsilon}_i $$

where *p*_*i*_(*condition*) is the probability of *i* having the given condition, *t*_*i*_ is the year *i* completed the survey, *female*_*i*_ is 1 if *i* is female and 0 otherwise, ***age***_***i***_ is a vector of dummy variables indicating *i* ’s age in 5 year age brackets, *w*_*i*_ is the individual level HSE weight for *i*, the ***β***_***i***_ s are parameters to be estimated and *ε*_*i*_ is an extreme valued error term. Fitted values from the models were used as estimates of disease prevalence at a given time among individuals in a given gender and age group. HSE has respondents from England only, and it was assumed that disease prevalence in Wales would be the same as for England. We discuss the impact of this assumption on our results in section 4.

#### Number of people with long-term conditions

The results for disease prevalence were combined with estimates of the mid-year populations of England and Wales to produce yearly estimates of the number of people with a given long-term condition. Monthly and quarterly estimates were then interpolated using cubic splines. Cubic spline interpolation was performed using the Forsythe, Malcolm and Moler method [44], as implemented in the spline function of the Stats package for R.

#### Fraction of individuals with a condition who are claimants

Combining estimates of the number of people with a long-term condition with data on the number of welfare (PIPS) claimants resulted in estimates of the fraction of individuals with a given condition who claim a given benefit at a given level, and whether under non-SRTI or SRTI.^5^

Logistic growth models [45, 46] were then fitted to the data on the fraction of individuals claiming benefits. Separate models were estimated for non-SRTI and SRTI, for each level of each benefit, for each five-year age category and for males and females, provided at least three periods with a positive number of claimants was observed.

The logistic growth models had the form

$$ {f}_t=\frac{A}{1+{e}^{-\left( rt-k\right)}}+{\varepsilon}_t. $$

Here *f*_*t*_ is the fraction of individuals claiming at time *t*, *A* is the long-run value of *f*_*t*_, which it approaches asymptotically, and *r* is the growth rate, with higher values of *r* meaning that *f*_*t*_ approaches *A* more quickly. *k* is a constant and *ε*_*t*_ is an error term. Models were estimated using non-linear least squares as implemented in the Stats package for R. In some cases, models failed to converge. In this instance, the models were re-arranged to be linear:

$$ \mathit{\ln}\left(\frac{\frac{f_t}{A}}{1-\frac{f_t}{A}}\right)= rt-k+{\mu}_t $$

Several models of this form were then estimated using OLS with $\mathit{\ln}\left(\frac{\frac{f_t}{A}}{1-\frac{f_t}{A}}\right)$ as the dependent variable and a range of values chosen for *A*. The final *A* was then chosen as the one from the model with the highest *R*^2^.

The estimated parameters were used to project the fraction of claimants from 2020 until 2025. In cases where fewer than three periods had a positive number of claimants, zero claimants were projected in future.

Hypothetical scenarios were constructed by choosing alternative values for *A* and/or *r*. The alternative value of the constant *k* was calculated using

$$ {k}^{hyp}=\mathit{\ln}\left(\frac{A^{hyp}}{f_T^{est}}-1\right)+{r}^{hyp}T $$

where *k*^*hyp*^, *A*^*hyp*^ and *r*^*hyp*^ are the parameters for the hypothetical scenario and *T* is the final period of estimation and ${f}_T^{est}$ is the estimated fraction claiming in *T*. This ensures that in period *T* the fraction of individuals claiming is identical for both the estimates based on current rules and for the hypothetical scenario.

#### Current expenditure

Projections of fractions of individuals claiming, either based on current rules or hypothetical scenarios under ‘new rules’ for SRTI were combined with the estimates of the number of people with given conditions in order to calculate the number of claimants, and from these expenditure. Monthly results were converted to annual results to aid comprehension.

The estimated expenditure on PIP in each financial year from 2013/14 to 2018/19 was compared to observed expenditure. Projected expenditure was calibrated by multiplying it by the average ratio of observed to estimated expenditure.

#### Hypothetical scenarios for claims under ‘new rules SRTI’

Three main scenarios were modelled: low, medium and high increases in the number of individuals claiming under ‘new rules’ SRTI. Various assumptions were common to each scenario about where increases would be seen and how big they would be, largely guided by the work of Etkind et al. [47]. Each scenario had the following assumptions in common:-

1. Increases would be seen in disease categories previously used by Etkind et al. [47] and others [48, 49] to estimate and project palliative care need. These categories are: infectious and parasitic diseases; neoplasms; mental and behavioural disorders; diseases of the nervous system; diseases of the circulatory system; diseases of the respiratory system; diseases of the digestive system; and diseases of the genitourinary system.
2. No increase in claims by individuals under the age of 40. (on the basis of age related trends in palliative care need) [47].
3. To reflect the requirement to have been claiming PIP before the State Pension age, lower increases were predicted among older age groups. Specifically, increases for individuals aged 65–69 were assumed to be 75% of the increases for people under 65, and increases for people aged over 70 were assumed to be 10% of the increases for people under 65. These fractions were chosen to be similar to the current fractions of individuals claiming aged 60–64 and 65 and over.
4. No change to the rate of increase of claims (i.e. *r* in the logistic growth model equation.)
5. A total of 10% of new claimants would otherwise have claimed under non-SRTI.

For the remaining disease and age categories, the following assumptions were made about the increase in the long-run fraction of individuals claiming (i.e. *A* in the logistic growth model equation):-

1. Low scenario: an increase of 1 percentage point;
2. Medium scenario: an increase of 2 percentage points;
3. High scenario: an increase of 3 percentage points.

These numbers were chosen as they lead to projected numbers of claimants of similar orders of magnitude as projections of numbers requiring palliative care. The assumptions detailed above are summarised in Table 2.

**Table 2:** Hypothetical scenarios under ‘new rules SRTI’

Scenario	Long-run claim increase	Growth rate	Age threshold for claim increase	Fraction of increased claims from non-SRTI
Main scenarios				
Low increase	1 pp	1 x current rate	40	10%
Medium increase	2 pp	1 x current rate	40	10%
High increase	3 pp	1 x current rate	40	10%
Robustness – varying growth rate				
Low increase, fast growth	1 pp	5 x current rate	40	10%
Low increase, very fast growth	1 pp	10 x current rate	40	10%
Medium increase, fast growth	2 pp	5 x current rate	40	10%
Medium increase, very fast growth	2 pp	10 x current rate	40	10%
High increase, fast growth	3 pp	5 x current rate	40	10%
High increase, very fast growth	3 pp	10 x current rate	40	10%
Robustness – varying claims from non-SRTI				
Low increase, low claims from non-SRTI	1 pp	1 x current rate	40	0%
Low increase, medium claims from non-SRTI	1 pp	1 x current rate	40	5%
Low increase, high claims from non-SRTI	1 pp	1 x current rate	40	20%
Medium increase, low claims from non-SRTI	2 pp	1 x current rate	40	0%
Medium increase, medium claims from non-SRTI	2 pp	1 x current rate	40	5%
Medium increase, high claims from non-SRTI	2 pp	1 x current rate	40	20%
High increase, low claims from non-SRTI	3 pp	1 x current rate	40	0%
High increase, medium claims from non-SRTI	3 pp	1 x current rate	40	5%
High increase, high claims from non-SRTI	3 pp	1 x current rate	40	20%
Robustness – varying age threshold for claim increase				
Low increase, age 20 threshold	1 pp	1 x current rate	20	10%
Low increase, age 30 threshold	1 pp	1 x current rate	30	10%
Low increase, age 50 threshold	1 pp	1 x current rate	50	10%
Medium increase, age 20 threshold	2 pp	1 x current rate	20	10%
Medium increase, age 30 threshold	2 pp	1 x current rate	30	10%
Medium increase, age 50 threshold	2 pp	1 x current rate	50	10%
High increase, age 20 threshold	3 pp	1 x current rate	20	10%
High increase, age 30 threshold	3 pp	1 x current rate	30	10%
High increase, age 50 threshold	3 pp	1 x current rate	50	10%

Each scenario has the following assumptions in common:- (1) Increased SRTI claims were only seen in the disease categories infectious and parasitic diseases; neoplasms; mental and behavioural disorders; diseases of the nervous system; diseases of the circulatory system; diseases of the respiratory system; diseases of the digestive system; and diseases of the genitourinary system. (2) Long-run claim increases for 65–69 year olds and over 70s were 75 and 10% respectively of the increases for individuals aged under 65

*SRTI* special rules for the terminally ill, *pp* percentage point(s)

#### Robustness tests

For each main scenario, several alternative scenarios were modelled making alternative assumptions about the growth rate of claims, age threshold for increased claims and fraction of the individuals who otherwise would have claimed under non-SRTI, as detailed in Table 2.

#### Case study: dementia

Wittenberg et al. [28] provide projections of dementia prevalence in England and Wales for each of the years 2019, 2020 and 2025. In each year, the numbers were broken down by severity of dementia according to the Mini-Mental State Examination score (mild dementia = 21–26; moderate dementia = 10–20; and severe dementia = 0–10) [50]. Wittenberg et al.’s results were used to create annual projections from 2019 to 2025 of the number of people with each level of dementia severity. This was achieved using cubic spline interpolation, specifically the Forsythe, Malcolm and Moler method [44] as implemented in the spline function of the Stats package for R. The prediction of the number of people were used to calculate the maximum possible increase in PIP expenditure assuming that:-

1. Everyone with dementia claims;
2. Only people with severe and moderate dementia claim;
3. Only people with severe dementia claim.

As Wittenberg et al’s results do not provide a breakdown of their figures by age, it is difficult to assess how many people would be able to start claiming before the State Pension age, as required by PIP rules. The aim of the exercise was to estimate an upper limit to PIP expenditure, and so we simply made the (unrealistic) assumption that all individuals with a given severity of dementia would be able to claim.

## Results

### Disease prevalence

**Fig. 2:**
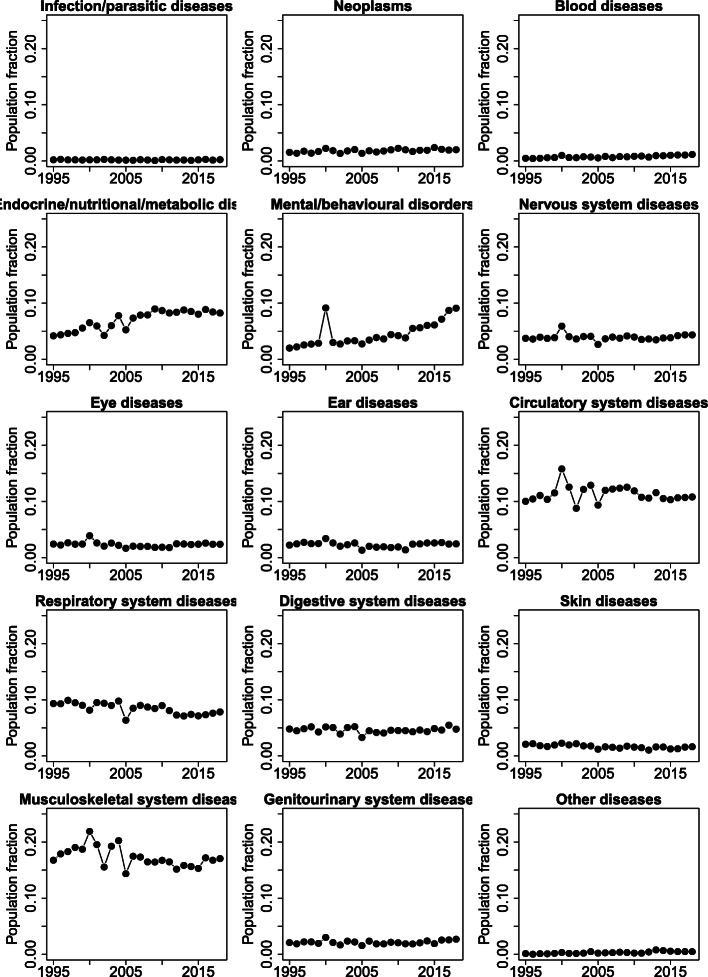
Estimated disease prevalence based on Health Survey for England

Figure 2 illustrates the estimated prevalence of long-term conditions over time classified using ICD-10. The most common category was ‘Diseases of the musculoskeletal system and connective tissue’, with a little under 20% of the population estimated to have a condition. Most categories were stable over time, but there were upward trends for ‘Mental and behavioural disorders’ and ‘Endocrine, nutritional and metabolic diseases’.

### Number of people with long-term conditions

**Fig. 3:**
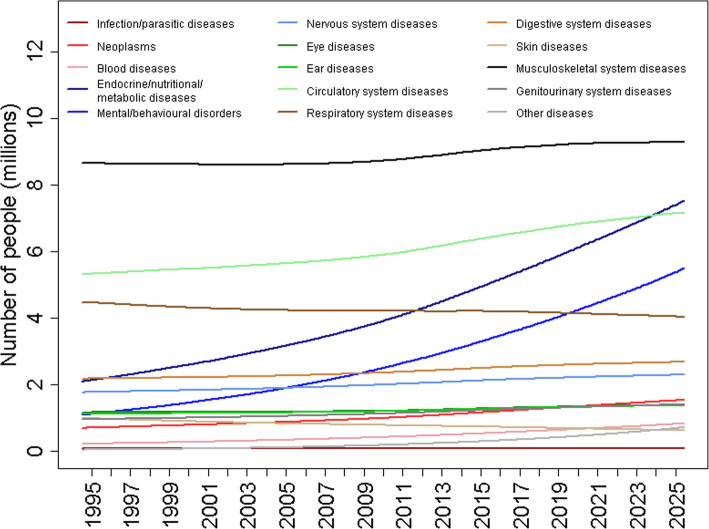
Estimated and projected number of people with long-term conditions

Figure 3 illustrates the estimates of the number of people with long-term conditions, accounting for a growing and aging population, between 1995 and 2025. There is an upward trend for most disease categories due to the growing population, with the trends exaggerated for categories where the fraction of individuals with a condition is also growing.

### Fraction of individuals with a disease who are claimants

Table 3 lists the estimated maximum percentages of the disease population claiming PIP for each disease category. For many categories, very few claims (< 1%) were seen, for example diseases of the blood, diseases of the ear and diseases of the circulatory system. Several categories had a significant fraction of individuals claiming under non-SRTI, for example diseases of the nervous system with between 2 and 9% of individuals claiming depending on the award and level. The greatest fraction of claims was seen for mental and behavioural disorders, where over 11% of individuals were estimated to claim the daily living award at the standard rate.

**Table 3:** Estimated long-term percentages of disease populations claiming PIP

Disease	Percentage of disease population claiming					
	SRTI		Non-SRTI			
	Daily living	Mobility	Daily living		Mobility	
	Enhanced rate	Enhanced rate	Standard rate	Enhanced rate	Standard rate	Enhanced rate
Certain Infectious and Parasitic Diseases (A00 - B99)	0.01	0.01	3.39	5.24	5.48	2.26
Neoplasms (C00 - D48)	1.64	1.63	1.49	1.84	1.74	0.89
Diseases of the Blood and Blood forming organs and certain diseases involving the immune mechanism (D50 - D89	0	0	0.33	0.55	0.54	0.27
Endocrine, Nutritional and Metabolic Diseases (E00 - E90)	0	0	0.2	0.26	0.28	0.13
Mental and Behavioural Disorders (F00 - F99)	0.01	0.01	11.37	5.99	7.27	4.91
Diseases of the Nervous System (G00 - G99)	0.06	0.06	6.87	4	8.77	1.99
Diseases of the Eye and Adnexa (H00 - H59)	0	0	2.01	0.44	2.44	0.31
Diseases of the Ear and Mastoid Process (H60 - H95)	0	0	0.38	0.46	0.23	1.2
Diseases of the Circulatory System (I00 - I99)	0.01	0	0.19	0.43	0.34	0.24
Diseases of the Respiratory System (J00 - J99)	0.02	0.02	0.65	1.26	1.19	0.69
Diseases of the Digestive System (K00 - K93)	0.01	0.01	0.25	0.47	0.28	0.22
Diseases of the Skin and Subcutaneous System (L00 - L99)	0	0	0.22	0.42	0.37	0.2
Diseases of the Musculoskeletal system and Connective Tissue (M00 - M99)	0.01	0.01	1.78	4.9	2.72	2.41

For all disease categories, a small proportion of people were estimated to claim under SRTI. The exception was neoplasms, where approximately 1.6% of individuals were estimated to claim both the daily living and mobility awards. In all other disease categories less than 0.1% of people claimed under SRTI.

Splitting data on the fraction of claimants by disease, PIP component, level, rules claimed under, age category and gender resulted in 2340 sub-datasets. Of these, 596 had fewer than three periods with a positive number of claims, meaning zero claims were projected in those groups in future. Logistic regression models were run on the remaining 1744 sub-datasets, and in 155 (8.0%) of cases failed to converge The mean number of monthly claimants in the cases where logistic growth models failed to converge was 9.7. The mean residual standard error of converged logistic growth models was 7.8 × 10^− 4^. Full model results are provided as supplementary online material.

### Current expenditure

The average ratio of estimated PIP expenditure from data on the number of claimants to actual expenditure from published figures was 1.13. Predicted future expenditure under the current SRTI regime and hypothetical future SRTI regimes was calibrated by multiplying by this ratio. Table 4 lists the estimated expenditure each year under the current regime. Expenditure was projected to increase over the period from 2020 to 2025 for both award types (daily living and mobility) under both non-SRTI and SRTI. However, expenditure on SRTI was considerably lower than for non-SRTI.

**Table 4:** Estimated future Personal Independence Payment expenditure under current SRTI regime

Rules	Component	Expenditure (£ millions)					
		2020	2021	2022	2023	2024	2025
Non-SRTI	Daily Living Enhanced	4641	4822	4988	5143	5297	5455
	Daily Living Standard	3261	3343	3413	3472	3526	3582
	Mobility Enhanced	3154	3312	3427	3518	3598	3676
	Mobility Standard	749	776	799	819	839	858
SRTI	Daily Living Enhanced	137	141	144	148	151	154
	Mobility Enhanced	94	97	99	102	104	106

### Hypothetical scenarios for claims under ‘new rules SRTI’

**Fig. 4:**
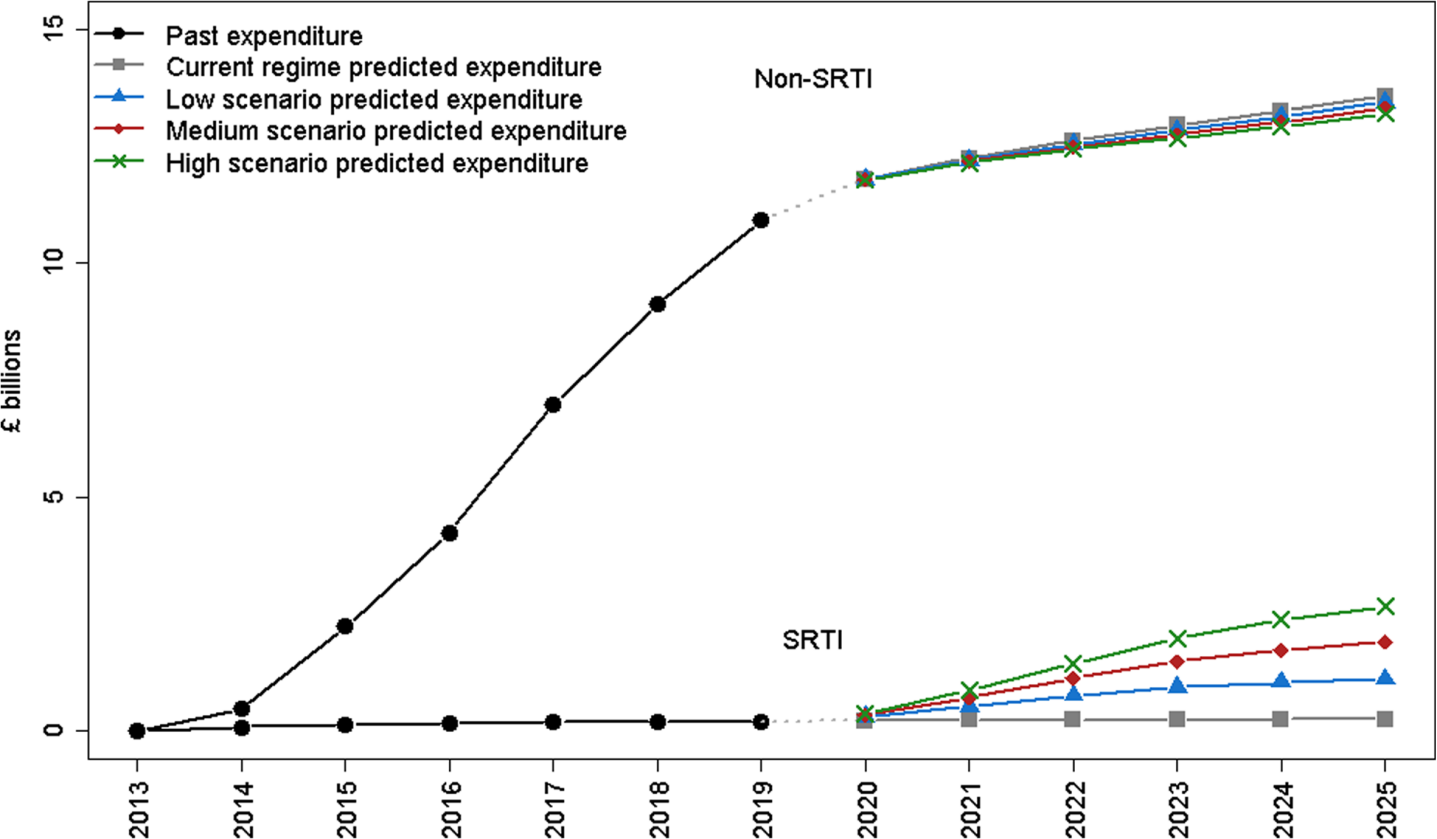
Past and predicted personal independence payment (PIP) expenditure, comparing the current SRTI scheme and the proposed new SRTI scheme, under different scenarios for low, medium and high increases in claims

Figure 4 compares projected expenditure under the current SRTI regime and under the three main scenarios of low, medium and high increases in claims under a new SRTI regime, with full numbers available in Table of the online supplementary material. As expected, SRTI expenditure was higher under the hypothetical scenarios representing possible new SRTI regimes than projections under the current regime, going from around £0.260 billion per year in the current regime to £1.1 billion per year by 2025 in the low scenario, £1.9 billion pounds in the medium scenario and £2.6 billion in the high scenario. However, even in the high increase scenario, expenditure under non-SRTI was still many times higher than under SRTI.

### Robustness tests

**Fig. 5:**
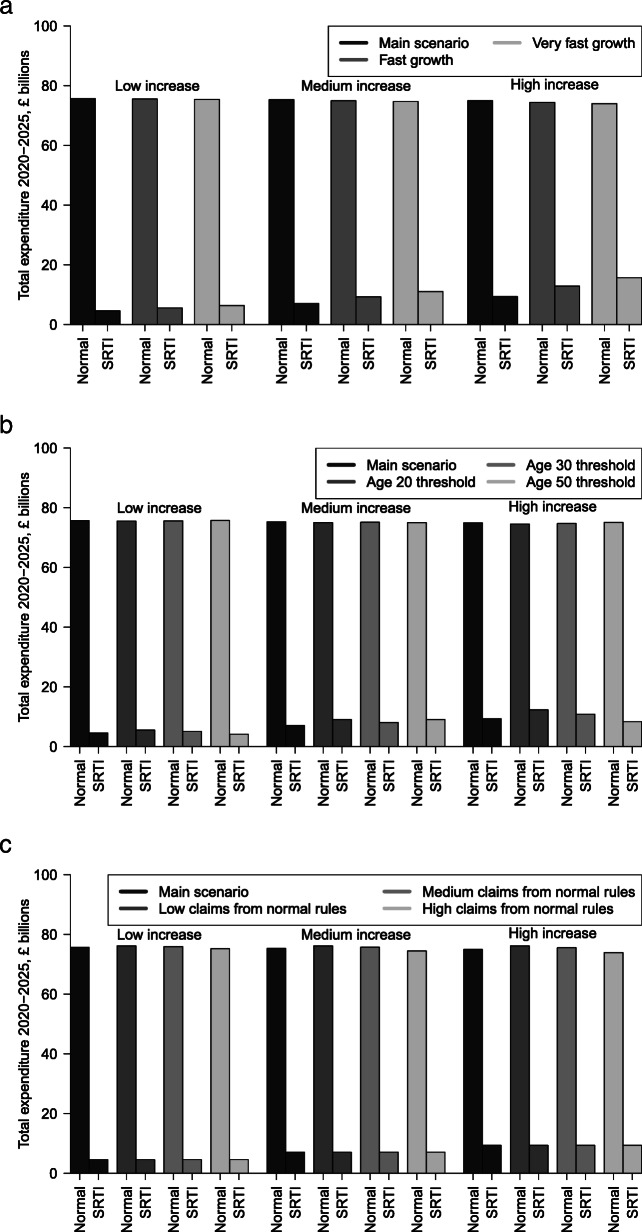
Comparison of modelling scenarios with varying assumptions about **a** growth rate, **b** age thresholds for claim increases and **c** fraction of increased claims from non-special rules for the terminally ill

Figure 5a illustrates how robust the scenarios are to varying the assumptions about the rate of increase of SRTI claims, with full numbers available in Table of the supplementary online material. It can be seen that if claims grow to their new long-term levels at a rate either five or 10 times the current estimated growth rate, expenditure under SRTI increased. This effect was more pronounced for the high increase scenario. However, the overall conclusion that expenditure under non-SRTI is several times higher than SRTI expenditure remains unchanged.

Figure 5b illustrates the impact of varying the age threshold at which increases in SRTI claims occur, with full numbers available as supplementary on-line material. Some variation in expenditure was seen, however the amount of variation was not as large as seen when varying the growth rate. Figure 5c shows the impact of varying the fraction of new SRTI claims which otherwise would have been made under non-SRTI. Little variation of non-SRTI expenditure was seen, indicating that any cost offsets were probably small.

### Case study: dementia

Table 5 lists the projected expenditure of all individuals with varying severity of dementia claimed under SRTI. The majority of expenditure was on individuals with severe dementia, and there was an increasing trend in expenditure.

**Table 5:** Maximum expenditure if individuals with varying severity of dementia are eligible to claim under SRTI

Dementia severity	Cost (£ billions)						
	2019	2020	2021	2022	2023	2024	2025
All	6.15	6.33	6.51	6.71	6.92	7.15	7.38
Moderate and severe	5.27	5.43	5.61	5.79	5.99	6.19	6.41
Severe only	3.57	3.8	4.01	4.2	4.37	4.53	4.68

## Discussion

The overall finding from projecting several different scenarios is that, although SRTI expenditure will increase following any change to eligibility, it will remain a fraction of expenditure under non-SRTI. For example, even in the high increase scenario, the projected cost of PIP under SRTI of just over £2.5 billion in 2025 is still only around a fifth of the projected cost under non-SRTI. This conclusion appears to be robust to the assumptions made about how quickly new SRTI claims will grow, the age profile of new SRTI claimants and how many SRTI claims would otherwise have been made under non-SRTI.

To examine the plausibility of the hypothetical scenarios, we can compare the number of PIP claims made under each scenario to the number who are predicted to require palliative care. This comparison is useful as it is likely that the population eligible to claim under any future SRTI scheme will overlap closely with the population requiring palliative care.

Etkind et al. [47] estimated that the number of people in England and Wales requiring palliative care will rise from between 375,000 and 385,000 in 2020 to between 410,000 and 445,000 by 2030. By comparison, in the high increase scenario, PIP claims under SRTI are projected to be over 600,000 by 2025. However, guidelines in Scotland where a change in the SRTI regime has already occurred state that to be eligible an individual’s disease should typically be advanced, progressive, not amenable to further curative treatment, and/or with worsening symptoms despite optimal management. Under any similar regime it seems unlikely that so many people would be eligible for SRTI while not requiring palliative care, indicating the high increase scenario represents an extreme case which is unlikely to occur in reality.

Under the medium and low increase scenarios, the number of people claiming under a new SRTI scheme is projected by 2025 to reach 438,000 and 255,000, respectively. Such numbers seem more plausible, and which scenario is closer to reality may be determined by what fraction of eligible people claim.

The modelling approach of assuming the long-run fractions of individuals claiming remain stable over time means the analysis is able to take account of demographic changes. Thus, for example, the number of people with dementia is projected to rise as the number of older people in the UK also rises. However, the analysis takes account of this by assuming that in the long run the fraction of, for example, women aged 65–69 with dementia who claim PIP under SRTI will remain stable.

Dementia is an important case study to examine, as it is a common condition which is growing with the aging population. In the case in which all people with dementia claim under SRTI, the increased expenditure is large, rising from £6.3 billion in 2020 to £7.4 billion in 2025. However, the expenditure should still be seen in the context of being only just over half the projected expenditure under non-SRTI in 2025.

The dementia scenarios presented here should all be regarded as extreme scenarios, and it is extremely implausible that any would occur. For example, they would either require all individuals to start claiming before the State Pension age, or for the abolition of that rule, a remote possibility given PIP’s function as an income-replacement benefit. Even for people below the age threshold, if any new SRTI regime had similar guidelines to those in Scotland cited above, it seems unlikely that even all people with severe dementia would be eligible, meaning a maximum expenditure of £4.7 billion by 2025. In addition, it is unlikely that all those eligible would in fact claim PIP, further reducing potential costs, and it is implausible that there would be no cost offsets in the form of reduced PIP claims under non-SRTI. Although the scenarios are unrealistic, we believe they are still useful to present in the light of policy concerns about potentially very large costs associated with dementia [50–52].

Most PIP claims under SRTI were from people with cancer, and very few SRTI claims were seen from any other disease category. This confirms previous findings which show that clinicians were reluctant to certify patients in disease areas other than cancer [13], and also that the trajectory of terminal illness such as COPD or dementia are much more difficult to predict than cancer [12].

The main implication of the current report for policy is that whilst changes to the SRTI scheme will increase expenditure on PIPS and other benefits, the cost implications will be small compared to the overall cost of PIP. The affordability of any change is ultimately a political question, however this research shows that it is unlikely to lead to significant increases to PIP expenditure in percentage terms.

In this report a wide variety of possible scenarios were modelled. It will be important in future research to compare the changes in Scotland, where a new regime is already in place, with the potential scenarios modelled here for England and Wales.

The analysis has several limitations. It only examines the potential impact of changes to SRTI on PIP, and does not address other welfare payments eligible under the SRTI scheme such as Attendance Allowance (AA), Employment Support Allowance (ESA) or Universal Credit (UC). While a similar pattern of expenditure could be expected for other payments, the population claiming them are not necessarily similar to people claiming PIP. For example ESA, unlike PIP, is considered an in-work benefit, and only people over 65 are eligible for AA.

It is a limitation of this study that its results may not be generalizable to other benefits. However, a full analysis of the impact of an SRTI regime change on the whole England and Wales benefit system was beyond the scope of our research project, as we instead sought to provide a detailed analysis of one welfare payment. The reason for choosing PIP was the available data made it more amenable to analyse than AA, ESA or UC. From the available data, it is clear what rules individuals are claiming under, unlike ESA. Individuals’ primary long-term condition is categorised according to a widely used system with PIP, making comparisons with other datasets on e.g. disease prevalence easier, unlike AA which uses a system apparently unique to DWP statistics. PIP data is also reported monthly, unlike AA and ESA which is reported quarterly. Finally, there will inevitably be a period of adjustment after any change of eligibility. As PIP began in 2013 and the number of claims have grown steadily since then, it is possible to use the data to estimate the dynamics of an adjustment period. Further work is necessary to analyse the full expenditure impact of a new SRTI regime.

While the analysis controlled for disease category, the available data only classified claims according to chapter-level ICD-10 codes. There are very broad, for example the category mental and behavioural disorders includes dementia, where potentially a large number of claims under SRTI could be made, as well as many non-terminal conditions such as schizophrenia and learning difficulties. In addition, disease prevalence was calculated for both England and Wales using data only from England. However, we do not expect this will have a large impact on results. England and Wales have many similarities, both being constituent parts of the United Kingdom, and the relative population sizes (around 55 million for England compared to 3 million for Wales) will tend to be dominated by those for England.

Due to the complexity of the calculations, confidence intervals were not explicitly computed for any given scenario. However, a range of scenarios were modelled, and robustness tests were performed with several key variables in order to give greater confidence that the conclusions were robust even to extreme situations in which twice as many people were claiming PIP under SRTI as were in need of palliative care.

This analysis only addresses the costs of any potential change to SRTI. It does not attempt to quantify any economic offsets or other benefits that may occur. The relationship between financial support and economic outcomes is complex and there are various ways that improved welfare provision can lead to economic benefits. These benefits may include lower health care costs at the end of life [53, 54]; decreased carer burden amongst informal/family caregivers [55]; improved bereavement outcomes for family carers [56, 57]; impacts on family carer employment status and ability to continue in/return to the workforce [58, 59]; impacts on place of death and achieving preferred place of death [54, 60].

## Conclusion

A change to the SRTI scheme which expands eligibility to any person with terminal illness will lead to increased government expenditure on PIP, and this increase is likely to be mirrored by similar increases in expenditure on other eligible benefits. However, the cost implications of a change to SRTI will be small compared to the overall cost of PIP. In the most plausible ‘low increase’ and ‘medium increase’ scenarios, the projected annual costs of PIP claims under the new SRTI scheme are around £1.1 billion and £1.9 billion respectively. This compares to the projected cost of over £12 billion under non-SRTI. There are several ways that changes could be implemented, with varying consequences for eligibility and uptake. However, a range of scenarios were modelled, and the conclusions were robust even to extreme situations. Careful consideration should be given to how terminal illness is defined under any new SRTI scheme, as this is likely to have significant implications for eligibility and subsequently on expenditure. Finally, any increase to the overall cost is likely to be offset, at least to some extent, by wider economic benefits that are realised through improving financial support for people with terminal illness.

## Supplementary Information

**Additional file 1: Table S1.** Detailed model results.

**Additional file 2: Table A 1.** Estimated future personal independence payment expenditure under different scenarios.

## Data Availability

All data is publically available. Population data comes from the Office of National Statistics [16, 17]. Data on disease prevalence taken from the Health Survey for England 1995–2018 [18–42]. Data on welfare claims comes from the Department for Work and Pensions’ Stat-Xplore service: https://stat-xplore.dwp.gov.uk/.

## Footnotes

1 https://www.gov.uk/government/publications/benefit-expenditure-and-caseload-tables-2019, accessed 03/02/21

2 Data from https://stat-xplore.dwp.gov.uk

3 https://stat-xplore.dwp.gov.uk/

4 https://www.gov.uk/pip/what-youll-get accessed 4/5/2020.

5 For a small number of five-year age categories in the disease category Certain infections and parasitic diseases, more people were observed to claim than were estimated to exist in the population. Low absolute numbers of people were involved, which led to the underestimation of the disease prevalence. In these cases, the estimated population was assumed to split evenly between claiming all levels of a given benefit.
