# Supplementary material for: Changing the eligibility criteria for welfare payments at the end of life – a budget impact analysis for England and Wales
Source: BMC Health Serv Res. 2021 May 4;21:421. doi: 10.1186/s12913-021-06390-8 (PMC8094542; doi:10.1186/s12913-021-06390-8)
Supplement: Supplementary file 1 — Additional file 1: Table S1. Detailed model results. [file 12913_2021_6390_MOESM1_ESM.pdf]

Table 1: Detailed model results

ICD-10 code	Component	Level	Rules	Ages	Female	Model type	A	s.e.	r	s.e.	k	s.e	RSE
A00-B99	Daily living	Enhanced	SRTI	16-17	Y	No model - fewer than 3 periods with positive claims							
A00-B99	Daily living	Enhanced	SRTI	16-17	N	No model - fewer than 3 periods with positive claims							
A00-B99	Daily living	Enhanced	SRTI	18-19	Y	No model - fewer than 3 periods with positive claims							
A00-B99	Daily living	Enhanced	SRTI	18-19	N	No model - fewer than 3 periods with positive claims							
A00-B99	Daily living	Enhanced	SRTI	20-24	Y	No model - fewer than 3 periods with positive claims							
A00-B99	Daily living	Enhanced	SRTI	20-24	N	No model - fewer than 3 periods with positive claims							
A00-B99	Daily living	Enhanced	SRTI	25-29	Y	No model - fewer than 3 periods with positive claims							
A00-B99	Daily living	Enhanced	SRTI	25-29	N	No model - fewer than 3 periods with positive claims							
A00-B99	Daily living	Enhanced	SRTI	30-34	Y	No model - fewer than 3 periods with positive claims							
A00-B99	Daily living	Enhanced	SRTI	30-34	N	No model - fewer than 3 periods with positive claims							
A00-B99	Daily living	Enhanced	SRTI	35-39	Y	No model - fewer than 3 periods with positive claims							
A00-B99	Daily living	Enhanced	SRTI	35-39	N	No model - fewer than 3 periods with positive claims							
A00-B99	Daily living	Enhanced	SRTI	40-44	Y	No model - fewer than 3 periods with positive claims							
A00-B99	Daily living	Enhanced	SRTI	40-44	N	No model - fewer than 3 periods with positive claims							
A00-B99	Daily living	Enhanced	SRTI	45-49	Y	No model - fewer than 3 periods with positive claims							
A00-B99	Daily living	Enhanced	SRTI	45-49	N	No model - fewer than 3 periods with positive claims							
A00-B99	Daily living	Enhanced	SRTI	50-54	Y	No model - fewer than 3 periods with positive claims							
A00-B99	Daily living	Enhanced	SRTI	50-54	N	No model - fewer than 3 periods with positive claims							
A00-B99	Daily living	Enhanced	SRTI	55-59	Y	No model - fewer than 3 periods with positive claims							
A00-B99	Daily living	Enhanced	SRTI	55-59	N	No model - fewer than 3 periods with positive claims							
A00-B99	Daily living	Enhanced	SRTI	60-64	Y	No model - fewer than 3 periods with positive claims							
A00-B99	Daily living	Enhanced	SRTI	60-64	N	LM	2.77×10^{-3}	—	0.157	0.019	-10.254	1.48	0.17
A00-B99	Daily living	Enhanced	SRTI	65-69	Y	No model - fewer than 3 periods with positive claims							
A00-B99	Daily living	Enhanced	SRTI	65-69	N	No model - fewer than 3 periods with positive claims							
A00-B99	Daily living	Enhanced	SRTI	70+	Y	No model - fewer than 3 periods with positive claims							
A00-B99	Daily living	Enhanced	SRTI	70+	N	No model - fewer than 3 periods with positive claims							
A00-B99	Daily living	Enhanced	non-SRTI	16-17	Y	LM	0.018	—	0.038	0.021	3.96×10^{-3}	0.876	0.494
A00-B99	Daily living	Enhanced	non-SRTI	16-17	N	LM	0.018	—	-0.144	0.073	10.611	5.129	0.947
A00-B99	Daily living	Enhanced	non-SRTI	18-19	Y	LM	6.75×10^{-3}	—	-3.40×10^{-3}	0.013	0.474	0.798	0.852
A00-B99	Daily living	Enhanced	non-SRTI	18-19	N	LM	2.26×10^{-3}	—	0.034	0.023	-1.101	1.685	0.403
A00-B99	Daily living	Enhanced	non-SRTI	20-24	Y	LG	0.01	3.13×10^{-4}	2.448	1.331	-146.596	79.737	1.44×10^{-3}
A00-B99	Daily living	Enhanced	non-SRTI	20-24	N	LG	3.79×10^{-3}	1.83×10^{-4}	3.032	5.634	-79.036	146.585	1.36×10^{-3}
A00-B99	Daily living	Enhanced	non-SRTI	25-29	Y	LM	0.019	—	0.033	3.62×10^{-3}	-2.969	0.199	0.52
A00-B99	Daily living	Enhanced	non-SRTI	25-29	N	LG	4.92×10^{-3}	3.79×10^{-4}	0.075	0.015	-2.604	0.421	1.03×10^{-3}
A00-B99	Daily living	Enhanced	non-SRTI	30-34	Y	LG	0.017	5.37×10^{-4}	0.112	0.011	-4.523	0.415	1.85×10^{-3}
A00-B99	Daily living	Enhanced	non-SRTI	30-34	N	LG	9.62×10^{-3}	2.63×10^{-4}	0.13	0.016	-3.951	0.464	1.29×10^{-3}
A00-B99	Daily living	Enhanced	non-SRTI	35-39	Y	LG	0.014	3.82×10^{-4}	0.105	0.011	-3.41	0.324	1.57×10^{-3}
A00-B99	Daily living	Enhanced	non-SRTI	35-39	N	LG	0.012	3.83×10^{-4}	0.087	7.36×10^{-3}	-3.337	0.236	1.10×10^{-3}
A00-B99	Daily living	Enhanced	non-SRTI	40-44	Y	LG	0.028	5.89×10^{-4}	0.109	8.95×10^{-3}	-3.353	0.256	2.57×10^{-3}
A00-B99	Daily living	Enhanced	non-SRTI	40-44	N	LG	0.03	1.04×10^{-3}	0.08	6.48×10^{-3}	-3.233	0.207	2.49×10^{-3}
A00-B99	Daily living	Enhanced	non-SRTI	45-49	Y	LG	0.028	4.14×10^{-4}	0.11	5.44×10^{-3}	-4.333	0.193	1.46×10^{-3}
A00-B99	Daily living	Enhanced	non-SRTI	45-49	N	LG	0.034	5.50×10^{-4}	0.09	3.88×10^{-3}	-3.702	0.133	1.49×10^{-3}
A00-B99	Daily living	Enhanced	non-SRTI	50-54	Y	LG	0.045	1.09×10^{-3}	0.086	4.25×10^{-3}	-4.089	0.159	2.09×10^{-3}
A00-B99	Daily living	Enhanced	non-SRTI	50-54	N	LG	0.058	8.26×10^{-4}	0.087	2.56×10^{-3}	-4.176	0.097	1.61×10^{-3}
A00-B99	Daily living	Enhanced	non-SRTI	55-59	Y	LG	0.033	7.76×10^{-4}	0.089	5.04×10^{-3}	-3.932	0.182	1.86×10^{-3}
A00-B99	Daily living	Enhanced	non-SRTI	55-59	N	LG	0.062	1.44×10^{-3}	0.08	2.82×10^{-3}	-4.379	0.109	1.76×10^{-3}
A00-B99	Daily living	Enhanced	non-SRTI	60-64	Y	LG	0.116	1.31×10^{-3}	0.129	4.71×10^{-3}	-5.971	0.201	4.15×10^{-3}
A00-B99	Daily living	Enhanced	non-SRTI	60-64	N	LG	0.118	2.19×10^{-3}	0.091	2.97×10^{-3}	-4.916	0.124	3.26×10^{-3}
A00-B99	Daily living	Enhanced	non-SRTI	65-69	Y	LG	0.158	9.12×10^{-4}	0.225	6.29×10^{-3}	-10.961	0.301	3.81×10^{-3}
A00-B99	Daily living	Enhanced	non-SRTI	65-69	N	LG	0.092	6.84×10^{-4}	0.174	5.22×10^{-3}	-8.381	0.241	2.56×10^{-3}
A00-B99	Daily living	Enhanced	non-SRTI	70+	Y	LG	0.028	6.32×10^{-4}	0.243	7.69×10^{-3}	-17.948	0.52	4.00×10^{-4}

Continued on next page

Table 1 – continued from previous page

ICD-10 code	Component	Level	Rules	Ages	Female	Model type	A	s.e.	r	s.e.	k	s.e	RSE
A00-B99	Daily living	Enhanced	non-SRTI	70+	N	LG	0.014	2.2×10^{-4}	0.302	0.013	-20.945	0.857	3.6×10^{-4}
A00-B99	Mobility	Enhanced	non-SRTI	16-17	Y	No model - fewer than 3 periods with positive claims							
A00-B99	Mobility	Enhanced	non-SRTI	16-17	N	No model - fewer than 3 periods with positive claims							
A00-B99	Mobility	Enhanced	non-SRTI	18-19	Y	LM	3×10^{-3}	—	0.082	0.046	-2.167	2.988	1.355
A00-B99	Mobility	Enhanced	non-SRTI	18-19	N	No model - fewer than 3 periods with positive claims							
A00-B99	Mobility	Enhanced	non-SRTI	20-24	Y	LM	0.01	—	4.85×10^{-3}	0.014	0.42	0.905	1.138
A00-B99	Mobility	Enhanced	non-SRTI	20-24	N	LG	5.50×10^{-3}	1.60×10^{-3}	0.055	0.018	-2.962	0.46	1.24×10^{-3}
A00-B99	Mobility	Enhanced	non-SRTI	25-29	Y	LG	7.75×10^{-3}	1.99×10^{-4}	0.275	0.054	-7.803	1.514	1.27×10^{-3}
A00-B99	Mobility	Enhanced	non-SRTI	25-29	N	LM	0.014	—	0.026	4.28×10^{-3}	-2.154	0.237	0.575
A00-B99	Mobility	Enhanced	non-SRTI	30-34	Y	LG	0.021	2.91×10^{-4}	0.191	0.015	-6.103	0.484	1.63×10^{-3}
A00-B99	Mobility	Enhanced	non-SRTI	30-34	N	LG	0.018	3.74×10^{-4}	0.123	9.68×10^{-3}	-4.596	0.338	1.52×10^{-3}
A00-B99	Mobility	Enhanced	non-SRTI	35-39	Y	LG	0.025	4.76×10^{-4}	0.093	5.30×10^{-3}	-3.521	0.173	1.50×10^{-3}
A00-B99	Mobility	Enhanced	non-SRTI	35-39	N	LG	0.02	3.51×10^{-4}	0.102	5.69×10^{-3}	-3.908	0.193	1.19×10^{-3}
A00-B99	Mobility	Enhanced	non-SRTI	40-44	Y	LG	0.056	7.38×10^{-4}	0.106	3.98×10^{-3}	-4.651	0.154	2.12×10^{-3}
A00-B99	Mobility	Enhanced	non-SRTI	40-44	N	LG	0.053	6.24×10^{-4}	0.097	2.94×10^{-3}	-4.343	0.112	1.59×10^{-3}
A00-B99	Mobility	Enhanced	non-SRTI	45-49	Y	LG	0.051	1.17×10^{-3}	0.08	3.16×10^{-3}	-4.116	0.118	1.72×10^{-3}
A00-B99	Mobility	Enhanced	non-SRTI	45-49	N	LG	0.044	5.16×10^{-4}	0.099	2.99×10^{-3}	-4.477	0.115	1.31×10^{-3}
A00-B99	Mobility	Enhanced	non-SRTI	50-54	Y	LG	0.069	8.77×10^{-4}	0.101	2.90×10^{-3}	-5.054	0.122	1.82×10^{-3}
A00-B99	Mobility	Enhanced	non-SRTI	50-54	N	LG	0.076	9.69×10^{-4}	0.102	2.82×10^{-3}	-5.309	0.122	1.87×10^{-3}
A00-B99	Mobility	Enhanced	non-SRTI	55-59	Y	LG	0.052	8.97×10^{-4}	0.115	5.78×10^{-3}	-5.297	0.239	2.57×10^{-3}
A00-B99	Mobility	Enhanced	non-SRTI	55-59	N	LG	0.077	1.83×10^{-3}	0.081	2.84×10^{-3}	-4.474	0.111	2.17×10^{-3}
A00-B99	Mobility	Enhanced	non-SRTI	60-64	Y	LG	0.201	2.04×10^{-3}	0.12	3.39×10^{-3}	-5.933	0.15	5.36×10^{-3}
A00-B99	Mobility	Enhanced	non-SRTI	60-64	N	LG	0.194	1.97×10^{-3}	0.107	2.56×10^{-3}	-5.448	0.112	4.26×10^{-3}
A00-B99	Mobility	Enhanced	non-SRTI	65-69	Y	LG	0.237	1.36×10^{-3}	0.213	5.99×10^{-3}	-9.986	0.274	5.89×10^{-3}
A00-B99	Mobility	Enhanced	non-SRTI	65-69	N	LG	0.171	7.47×10^{-4}	0.191	3.69×10^{-3}	-9.076	0.17	3.00×10^{-3}
A00-B99	Mobility	Enhanced	non-SRTI	70+	Y	LG	0.043	1.46×10^{-3}	0.217	0.012	-15.603	0.758	1.15×10^{-3}
A00-B99	Mobility	Enhanced	non-SRTI	70+	N	LG	0.03	5.85×10^{-4}	0.251	9.67×10^{-3}	-17.793	0.644	6.33×10^{-4}
A00-B99	Daily living	Standard	SRTI	16-17	Y	No model - fewer than 3 periods with positive claims							
A00-B99	Daily living	Standard	SRTI	16-17	N	No model - fewer than 3 periods with positive claims							
A00-B99	Daily living	Standard	SRTI	18-19	Y	No model - fewer than 3 periods with positive claims							
A00-B99	Daily living	Standard	SRTI	18-19	N	No model - fewer than 3 periods with positive claims							
A00-B99	Daily living	Standard	SRTI	20-24	Y	No model - fewer than 3 periods with positive claims							
A00-B99	Daily living	Standard	SRTI	20-24	N	No model - fewer than 3 periods with positive claims							
A00-B99	Daily living	Standard	SRTI	25-29	Y	No model - fewer than 3 periods with positive claims							
A00-B99	Daily living	Standard	SRTI	25-29	N	No model - fewer than 3 periods with positive claims							
A00-B99	Daily living	Standard	SRTI	30-34	Y	No model - fewer than 3 periods with positive claims							
A00-B99	Daily living	Standard	SRTI	30-34	N	No model - fewer than 3 periods with positive claims							
A00-B99	Daily living	Standard	SRTI	35-39	Y	No model - fewer than 3 periods with positive claims							
A00-B99	Daily living	Standard	SRTI	35-39	N	No model - fewer than 3 periods with positive claims							
A00-B99	Daily living	Standard	SRTI	40-44	Y	No model - fewer than 3 periods with positive claims							
A00-B99	Daily living	Standard	SRTI	40-44	N	No model - fewer than 3 periods with positive claims							
A00-B99	Daily living	Standard	SRTI	45-49	Y	No model - fewer than 3 periods with positive claims							
A00-B99	Daily living	Standard	SRTI	45-49	N	No model - fewer than 3 periods with positive claims							
A00-B99	Daily living	Standard	SRTI	50-54	Y	No model - fewer than 3 periods with positive claims							
A00-B99	Daily living	Standard	SRTI	50-54	N	No model - fewer than 3 periods with positive claims							
A00-B99	Daily living	Standard	SRTI	55-59	Y	No model - fewer than 3 periods with positive claims							
A00-B99	Daily living	Standard	SRTI	55-59	N	No model - fewer than 3 periods with positive claims							
A00-B99	Daily living	Standard	SRTI	60-64	Y	No model - fewer than 3 periods with positive claims							
A00-B99	Daily living	Standard	SRTI	60-64	N	LM	2.77×10^{-3}	—	0.157	0.019	-10.254	1.48	0.17
A00-B99	Daily living	Standard	SRTI	65-69	Y	No model - fewer than 3 periods with positive claims							
A00-B99	Daily living	Standard	SRTI	65-69	N	No model - fewer than 3 periods with positive claims							
A00-B99	Daily living	Standard	SRTI	70+	Y	No model - fewer than 3 periods with positive claims							
A00-B99	Daily living	Standard	SRTI	70+	N	No model - fewer than 3 periods with positive claims							

Continued on next page

Table 1 – continued from previous page

ICD-10 code	Component	Level	Rules	Ages	Female	Model type	A	s.e.	r	s.e.	k	s.e	RSE
A00-B99	Daily living	Standard	non-SRTI	16-17	Y	LM	0.02	—	0.038	0.031	-0.174	1.251	1.007
A00-B99	Daily living	Standard	non-SRTI	16-17	N	LM	9.52×10^{-3}	—	0.098	6.53×10^{-3}	-2.306	0.458	0.085
A00-B99	Daily living	Standard	non-SRTI	18-19	Y	LM	6.39×10^{-3}	—	-9.30×10^{-3}	0.013	0.754	0.764	0.936
A00-B99	Daily living	Standard	non-SRTI	18-19	N	LM	1.53×10^{-3}	—	0.691	0.136	-36.11	8.311	0.193
A00-B99	Daily living	Standard	non-SRTI	20-24	Y	LG	9.63×10^{-3}	5.90×10^{-4}	0.221	0.056	-12.046	2.974	2.02×10^{-3}
A00-B99	Daily living	Standard	non-SRTI	20-24	N	LG	5.34×10^{-3}	2.96×10^{-4}	0.303	0.09	-16.275	4.794	1.21×10^{-3}
A00-B99	Daily living	Standard	non-SRTI	25-29	Y	LG	7.27×10^{-3}	1.73×10^{-4}	0.254	0.039	-9.502	1.444	9.58×10^{-4}
A00-B99	Daily living	Standard	non-SRTI	25-29	N	LG	4.90×10^{-3}	2.14×10^{-4}	0.125	0.018	-5.612	0.734	6.97×10^{-4}
A00-B99	Daily living	Standard	non-SRTI	30-34	Y	LG	0.021	5.58×10^{-4}	0.109	8.85×10^{-3}	-4.536	0.33	1.80×10^{-3}
A00-B99	Daily living	Standard	non-SRTI	30-34	N	LG	0.012	2.76×10^{-4}	0.12	0.01	-4.768	0.374	1.03×10^{-3}
A00-B99	Daily living	Standard	non-SRTI	35-39	Y	LG	0.025	8.80×10^{-4}	0.074	4.64×10^{-3}	-3.607	0.159	1.36×10^{-3}
A00-B99	Daily living	Standard	non-SRTI	35-39	N	LG	0.016	5.27×10^{-4}	0.081	5.81×10^{-3}	-3.57	0.199	1.14×10^{-3}
A00-B99	Daily living	Standard	non-SRTI	40-44	Y	LG	0.042	8.12×10^{-4}	0.091	4.24×10^{-3}	-4.07	0.156	1.93×10^{-3}
A00-B99	Daily living	Standard	non-SRTI	40-44	N	LG	0.045	1.12×10^{-3}	0.082	3.57×10^{-3}	-4.148	0.135	1.74×10^{-3}
A00-B99	Daily living	Standard	non-SRTI	45-49	Y	LG	0.045	9.24×10^{-4}	0.081	2.91×10^{-3}	-4.129	0.11	1.41×10^{-3}
A00-B99	Daily living	Standard	non-SRTI	45-49	N	LG	0.045	8.97×10^{-4}	0.082	2.93×10^{-3}	-4.13	0.111	1.42×10^{-3}
A00-B99	Daily living	Standard	non-SRTI	50-54	Y	LG	0.061	1.20×10^{-3}	0.096	3.84×10^{-3}	-4.994	0.162	2.15×10^{-3}
A00-B99	Daily living	Standard	non-SRTI	50-54	N	LG	0.068	9.78×10^{-4}	0.102	3.30×10^{-3}	-5.188	0.141	2.00×10^{-3}
A00-B99	Daily living	Standard	non-SRTI	55-59	Y	LG	0.049	6.18×10^{-4}	0.107	3.80×10^{-3}	-4.773	0.149	1.76×10^{-3}
A00-B99	Daily living	Standard	non-SRTI	55-59	N	LG	0.076	1.20×10^{-3}	0.091	2.46×10^{-3}	-5.007	0.104	1.70×10^{-3}
A00-B99	Daily living	Standard	non-SRTI	60-64	Y	LG	0.229	1.60×10^{-3}	0.136	3.01×10^{-3}	-6.641	0.136	4.80×10^{-3}
A00-B99	Daily living	Standard	non-SRTI	60-64	N	LG	0.209	3.85×10^{-3}	0.091	2.77×10^{-3}	-5.121	0.119	5.11×10^{-3}
A00-B99	Daily living	Standard	non-SRTI	65-69	Y	LG	0.308	2.21×10^{-3}	0.228	8.23×10^{-3}	-10.911	0.386	9.58×10^{-3}
A00-B99	Daily living	Standard	non-SRTI	65-69	N	LG	0.178	7.16×10^{-4}	0.204	3.81×10^{-3}	-9.68	0.176	2.97×10^{-3}
A00-B99	Daily living	Standard	non-SRTI	70+	Y	LG	0.06	1.70×10^{-3}	0.219	8.57×10^{-3}	-16.005	0.566	1.11×10^{-3}
A00-B99	Daily living	Standard	non-SRTI	70+	N	LG	0.035	6.26×10^{-4}	0.253	8.93×10^{-3}	-17.962	0.596	6.77×10^{-4}
A00-B99	Mobility	Standard	non-SRTI	16-17	Y	No model - fewer than 3 periods with positive claims							
A00-B99	Mobility	Standard	non-SRTI	16-17	N	No model - fewer than 3 periods with positive claims							
A00-B99	Mobility	Standard	non-SRTI	18-19	Y	LM	2.78×10^{-3}	—	0.231	0.021	-3.456	0.766	0.03
A00-B99	Mobility	Standard	non-SRTI	18-19	N	No model - fewer than 3 periods with positive claims							
A00-B99	Mobility	Standard	non-SRTI	20-24	Y	LM	7.01×10^{-3}	—	-0.018	0.033	1.556	2.111	1.242
A00-B99	Mobility	Standard	non-SRTI	20-24	N	LM	2.58×10^{-3}	—	-0.031	0.013	2.719	0.545	0.74
A00-B99	Mobility	Standard	non-SRTI	25-29	Y	LM	6.02×10^{-3}	—	-6.11×10^{-3}	3.93×10^{-3}	-0.511	0.2	0.478
A00-B99	Mobility	Standard	non-SRTI	25-29	N	LM	7.24×10^{-3}	—	3.29×10^{-3}	7.69×10^{-3}	-0.727	0.457	0.796
A00-B99	Mobility	Standard	non-SRTI	30-34	Y	LG	0.013	3.66×10^{-4}	0.173	0.024	-6.322	0.863	1.83×10^{-3}
A00-B99	Mobility	Standard	non-SRTI	30-34	N	LG	0.01	2.08×10^{-4}	0.175	0.018	-6.496	0.644	1.03×10^{-3}
A00-B99	Mobility	Standard	non-SRTI	35-39	Y	LG	0.011	2.51×10^{-4}	0.143	0.017	-4.061	0.457	1.35×10^{-3}
A00-B99	Mobility	Standard	non-SRTI	35-39	N	LG	9.17×10^{-3}	2.06×10^{-4}	0.131	0.013	-4.199	0.397	9.86×10^{-4}
A00-B99	Mobility	Standard	non-SRTI	40-44	Y	LG	0.027	3.88×10^{-4}	0.139	8.55×10^{-3}	-5.124	0.3	1.73×10^{-3}
A00-B99	Mobility	Standard	non-SRTI	40-44	N	LG	0.024	4.43×10^{-4}	0.097	5.39×10^{-3}	-3.741	0.181	1.43×10^{-3}
A00-B99	Mobility	Standard	non-SRTI	45-49	Y	LG	0.019	3.42×10^{-4}	0.13	9.04×10^{-3}	-4.87	0.319	1.43×10^{-3}
A00-B99	Mobility	Standard	non-SRTI	45-49	N	LG	0.022	4.11×10^{-4}	0.107	6.62×10^{-3}	-4.028	0.226	1.47×10^{-3}
A00-B99	Mobility	Standard	non-SRTI	50-54	Y	LG	0.035	9.52×10^{-4}	0.086	4.85×10^{-3}	-4.087	0.181	1.87×10^{-3}
A00-B99	Mobility	Standard	non-SRTI	50-54	N	LG	0.039	5.91×10^{-4}	0.087	2.77×10^{-3}	-4.169	0.105	1.17×10^{-3}
A00-B99	Mobility	Standard	non-SRTI	55-59	Y	LG	0.027	6.18×10^{-4}	0.106	6.59×10^{-3}	-4.766	0.26	1.71×10^{-3}
A00-B99	Mobility	Standard	non-SRTI	55-59	N	LG	0.039	1.48×10^{-3}	0.076	4.36×10^{-3}	-4.004	0.159	1.85×10^{-3}
A00-B99	Mobility	Standard	non-SRTI	60-64	Y	LG	0.071	1.33×10^{-3}	0.109	5.81×10^{-3}	-4.87	0.23	3.80×10^{-3}
A00-B99	Mobility	Standard	non-SRTI	60-64	N	LG	0.083	1.07×10^{-3}	0.104	3.34×10^{-3}	-5.023	0.138	2.53×10^{-3}
A00-B99	Mobility	Standard	non-SRTI	65-69	Y	LG	0.079	5.72×10^{-4}	0.214	7.85×10^{-3}	-9.695	0.348	2.57×10^{-3}

Continued on next page

ICD-10 code	Component	Level	Rules	Ages	Female	Model type	A	s.e.	r	s.e.	k	s.e	RSE
A00-B99	Mobility	Standard	non-SRTI	65-69	N	LG	0.07	5.37×10^{-4}	0.17	5.25×10^{-3}	-8.052	0.239	2.02×10^{-3}
A00-B99	Mobility	Standard	non-SRTI	70+	Y	LG	0.014	5.89×10^{-4}	0.221	0.013	-16.171	0.854	3.71×10^{-4}
A00-B99	Mobility	Standard	non-SRTI	70+	N	LG	9.80×10^{-3}	1.39×10^{-4}	0.279	9.60×10^{-3}	-19.519	0.642	1.95×10^{-4}
C00-D48	Daily living	Enhanced	SRTI	16-17	Y	LM	8.39×10^{-3}	—	-0.012	8.16×10^{-3}	0.813	0.421	0.651
C00-D48	Daily living	Enhanced	SRTI	16-17	N	LM	0.013	—	-0.012	8×10^{-3}	0.448	0.453	0.84
C00-D48	Daily living	Enhanced	SRTI	18-19	Y	LG	5.20×10^{-3}	2.03×10^{-4}	0.536	0.25	-11.133	5.193	1.51×10^{-3}
C00-D48	Daily living	Enhanced	SRTI	18-19	N	LM	0.018	—	3.63×10^{-3}	2.92×10^{-3}	-0.803	0.159	0.413
C00-D48	Daily living	Enhanced	SRTI	20-24	Y	LG	5.25×10^{-3}	9.29×10^{-5}	0.376	0.07	-4.858	0.918	7.19×10^{-4}
C00-D48	Daily living	Enhanced	SRTI	20-24	N	LG	6.84×10^{-3}	1.14×10^{-4}	0.256	0.035	-4.337	0.597	8.13×10^{-4}
C00-D48	Daily living	Enhanced	SRTI	25-29	Y	LG	0.012	1.72×10^{-4}	0.192	0.022	-2.456	0.294	1.21×10^{-3}
C00-D48	Daily living	Enhanced	SRTI	25-29	N	LG	8.75×10^{-3}	1.32×10^{-4}	0.131	0.012	-2.011	0.2	8.15×10^{-4}
C00-D48	Daily living	Enhanced	SRTI	30-34	Y	LG	0.018	1.23×10^{-4}	0.205	0.011	-2.76	0.149	8.73×10^{-4}
C00-D48	Daily living	Enhanced	SRTI	30-34	N	LG	0.012	1.40×10^{-4}	0.117	6.97×10^{-3}	-2.269	0.137	7.77×10^{-4}
C00-D48	Daily living	Enhanced	SRTI	35-39	Y	LG	0.028	1.87×10^{-4}	0.167	7.52×10^{-3}	-2.576	0.121	1.25×10^{-3}
C00-D48	Daily living	Enhanced	SRTI	35-39	N	LG	0.018	1.71×10^{-4}	0.122	6.15×10^{-3}	-2.496	0.126	9.60×10^{-4}
C00-D48	Daily living	Enhanced	SRTI	40-44	Y	LG	0.033	2.17×10^{-4}	0.153	6.55×10^{-3}	-2.401	0.108	1.41×10^{-3}
C00-D48	Daily living	Enhanced	SRTI	40-44	N	LG	0.019	1.89×10^{-4}	0.141	9.04×10^{-3}	-2.06	0.142	1.20×10^{-3}
C00-D48	Daily living	Enhanced	SRTI	45-49	Y	LG	0.035	1.98×10^{-4}	0.16	6.05×10^{-3}	-2.437	0.097	1.31×10^{-3}
C00-D48	Daily living	Enhanced	SRTI	45-49	N	LG	0.021	1.22×10^{-4}	0.18	7.80×10^{-3}	-2.427	0.111	8.47×10^{-4}
C00-D48	Daily living	Enhanced	SRTI	50-54	Y	LG	0.045	2.24×10^{-4}	0.162	5.41×10^{-3}	-2.536	0.088	1.48×10^{-3}
C00-D48	Daily living	Enhanced	SRTI	50-54	N	LG	0.032	2.04×10^{-4}	0.156	6.87×10^{-3}	-2.122	0.101	1.36×10^{-3}
C00-D48	Daily living	Enhanced	SRTI	55-59	Y	LG	0.039	2.02×10^{-4}	0.171	6.20×10^{-3}	-2.486	0.095	1.37×10^{-3}
C00-D48	Daily living	Enhanced	SRTI	55-59	N	LG	0.034	1.73×10^{-4}	0.191	7.57×10^{-3}	-2.396	0.101	1.22×10^{-3}
C00-D48	Daily living	Enhanced	SRTI	60-64	Y	LG	0.046	2.45×10^{-4}	0.157	5.71×10^{-3}	-2.3	0.089	1.62×10^{-3}
C00-D48	Daily living	Enhanced	SRTI	60-64	N	LG	0.049	2.90×10^{-4}	0.174	7.51×10^{-3}	-2.261	0.105	2.00×10^{-3}
C00-D48	Daily living	Enhanced	SRTI	65-69	Y	LG	0.013	8.20×10^{-5}	0.107	2.63×10^{-3}	-3.342	0.076	3.48×10^{-4}
C00-D48	Daily living	Enhanced	SRTI	65-69	N	LG	0.013	8.92×10^{-5}	0.117	3.38×10^{-3}	-3.498	0.095	4.16×10^{-4}
C00-D48	Daily living	Enhanced	SRTI	70+	Y	LG	3.05×10^{-4}	9.43×10^{-6}	0.254	0.018	-17.608	1.19	1.28×10^{-5}
C00-D48	Daily living	Enhanced	SRTI	70+	N	LG	3.18×10^{-4}	1.07×10^{-5}	0.267	0.018	-19.063	1.234	1.17×10^{-5}
C00-D48	Daily living	Enhanced	non-SRTI	16-17	Y	LG	0.053	9.05×10^{-4}	0.234	0.029	-5.285	0.66	6.02×10^{-3}
C00-D48	Daily living	Enhanced	non-SRTI	16-17	N	LG	0.058	1.05×10^{-3}	0.229	0.029	-5.581	0.708	6.80×10^{-3}
C00-D48	Daily living	Enhanced	non-SRTI	18-19	Y	LG	0.048	5.48×10^{-4}	0.16	0.01	-4.239	0.262	3.15×10^{-3}
C00-D48	Daily living	Enhanced	non-SRTI	18-19	N	LG	0.058	9.42×10^{-4}	0.139	0.011	-3.961	0.301	4.96×10^{-3}
C00-D48	Daily living	Enhanced	non-SRTI	20-24	Y	LG	0.035	3.05×10^{-4}	0.159	7.74×10^{-3}	-4.144	0.199	1.76×10^{-3}
C00-D48	Daily living	Enhanced	non-SRTI	20-24	N	LG	0.039	5.63×10^{-4}	0.097	4.85×10^{-3}	-3.104	0.14	2.18×10^{-3}
C00-D48	Daily living	Enhanced	non-SRTI	25-29	Y	LG	0.029	2.39×10^{-4}	0.148	6.67×10^{-3}	-3.7	0.164	1.37×10^{-3}
C00-D48	Daily living	Enhanced	non-SRTI	25-29	N	LG	0.024	4.19×10^{-4}	0.106	7.38×10^{-3}	-3.02	0.197	1.88×10^{-3}
C00-D48	Daily living	Enhanced	non-SRTI	30-34	Y	LG	0.032	2.57×10^{-4}	0.164	7.72×10^{-3}	-4.021	0.187	1.54×10^{-3}
C00-D48	Daily living	Enhanced	non-SRTI	30-34	N	LG	0.019	1.97×10^{-4}	0.135	7.25×10^{-3}	-3.319	0.175	1.09×10^{-3}
C00-D48	Daily living	Enhanced	non-SRTI	35-39	Y	LG	0.035	3.52×10^{-4}	0.143	7.46×10^{-3}	-3.757	0.192	1.94×10^{-3}
C00-D48	Daily living	Enhanced	non-SRTI	35-39	N	LG	0.019	2.90×10^{-4}	0.109	6.89×10^{-3}	-3.137	0.186	1.32×10^{-3}
C00-D48	Daily living	Enhanced	non-SRTI	40-44	Y	LG	0.037	2.43×10^{-4}	0.196	8.50×10^{-3}	-4.465	0.193	1.55×10^{-3}
C00-D48	Daily living	Enhanced	non-SRTI	40-44	N	LG	0.017	1.40×10^{-4}	0.196	0.01	-4.632	0.244	8.88×10^{-4}
C00-D48	Daily living	Enhanced	non-SRTI	45-49	Y	LG	0.037	2.35×10^{-4}	0.194	8.12×10^{-3}	-4.384	0.183	1.50×10^{-3}
C00-D48	Daily living	Enhanced	non-SRTI	45-49	N	LG	0.017	1.44×10^{-4}	0.155	7.30×10^{-3}	-3.879	0.18	8.35×10^{-4}
C00-D48	Daily living	Enhanced	non-SRTI	50-54	Y	LG	0.04	3.19×10^{-4}	0.159	7.20×10^{-3}	-3.92	0.175	1.88×10^{-3}
C00-D48	Daily living	Enhanced	non-SRTI	50-54	N	LG	0.022	1.45×10^{-4}	0.189	7.89×10^{-3}	-4.351	0.181	9.17×10^{-4}
C00-D48	Daily living	Enhanced	non-SRTI	55-59	Y	LG	0.03	2.58×10^{-4}	0.149	6.78×10^{-3}	-3.76	0.168	1.47×10^{-3}
C00-D48	Daily living	Enhanced	non-SRTI	55-59	N	LG	0.022	1.42×10^{-4}	0.186	7.61×10^{-3}	-4.317	0.176	8.92×10^{-4}

Continued on next page

Table 1 – continued from previous page

ICD-10 code	Component	Level	Rules	Ages	Female	Model type	A	s.e.	r	s.e.	k	s.e	RSE
C00-D48	Daily living	Enhanced	non-SRTI	60-64	Y	LG	0.032	2.48×10^{-4}	0.134	5.06×10^{-3}	-3.653	0.133	1.31×10^{-3}
C00-D48	Daily living	Enhanced	non-SRTI	60-64	N	LG	0.03	2.18×10^{-4}	0.154	6.15×10^{-3}	-3.757	0.148	1.27×10^{-3}
C00-D48	Daily living	Enhanced	non-SRTI	65-69	Y	LG	0.015	1.26×10^{-4}	0.113	2.83×10^{-3}	-5.003	0.112	3.85×10^{-4}
C00-D48	Daily living	Enhanced	non-SRTI	65-69	N	LG	0.017	1.16×10^{-4}	0.109	2.23×10^{-3}	-4.736	0.086	3.53×10^{-4}
C00-D48	Daily living	Enhanced	non-SRTI	70+	Y	LG	1.16×10^{-3}	2.95×10^{-5}	0.229	9.51×10^{-3}	-16.484	0.63	2.44×10^{-5}
C00-D48	Daily living	Enhanced	non-SRTI	70+	N	LG	1.26×10^{-3}	2.46×10^{-5}	0.256	0.011	-18.098	0.701	2.89×10^{-5}
C00-D48	Mobility	Enhanced	non-SRTI	16-17	Y	LG	0.027	7.33×10^{-4}	0.114	0.01	-4.644	0.373	2.53×10^{-3}
C00-D48	Mobility	Enhanced	non-SRTI	16-17	N	LG	0.021	4.92×10^{-4}	0.122	0.012	-3.832	0.36	2.28×10^{-3}
C00-D48	Mobility	Enhanced	non-SRTI	18-19	Y	LG	0.024	1.16×10^{-3}	0.07	7.59×10^{-3}	-2.677	0.215	2.55×10^{-3}
C00-D48	Mobility	Enhanced	non-SRTI	18-19	N	LG	0.026	1.68×10^{-3}	0.069	7.85×10^{-3}	-3.189	0.247	2.62×10^{-3}
C00-D48	Mobility	Enhanced	non-SRTI	20-24	Y	LG	0.021	2.78×10^{-4}	0.133	7.51×10^{-3}	-4.513	0.243	1.28×10^{-3}
C00-D48	Mobility	Enhanced	non-SRTI	20-24	N	LG	0.02	4.50×10^{-4}	0.101	7.29×10^{-3}	-3.812	0.244	1.54×10^{-3}
C00-D48	Mobility	Enhanced	non-SRTI	25-29	Y	LG	0.026	6.75×10^{-4}	0.075	3.99×10^{-3}	-3.213	0.128	1.34×10^{-3}
C00-D48	Mobility	Enhanced	non-SRTI	25-29	N	LG	0.017	3.67×10^{-4}	0.089	5.30×10^{-3}	-3.566	0.177	1.02×10^{-3}
C00-D48	Mobility	Enhanced	non-SRTI	30-34	Y	LG	0.033	6.29×10^{-4}	0.078	3.19×10^{-3}	-3.404	0.106	1.31×10^{-3}
C00-D48	Mobility	Enhanced	non-SRTI	30-34	N	LG	0.019	6.21×10^{-4}	0.072	3.94×10^{-3}	-3.532	0.133	9.03×10^{-4}
C00-D48	Mobility	Enhanced	non-SRTI	35-39	Y	LG	0.056	4.05×10^{-3}	0.055	3.82×10^{-3}	-3.177	0.1	2.56×10^{-3}
C00-D48	Mobility	Enhanced	non-SRTI	35-39	N	LG	0.017	2.48×10^{-4}	0.086	2.92×10^{-3}	-3.704	0.102	5.89×10^{-4}
C00-D48	Mobility	Enhanced	non-SRTI	40-44	Y	LG	0.045	1.41×10^{-3}	0.068	3.89×10^{-3}	-3.037	0.118	2.32×10^{-3}
C00-D48	Mobility	Enhanced	non-SRTI	40-44	N	LG	0.018	4.20×10^{-4}	0.074	3.73×10^{-3}	-3.081	0.116	8.71×10^{-4}
C00-D48	Mobility	Enhanced	non-SRTI	45-49	Y	LG	0.043	1.04×10^{-3}	0.072	3.63×10^{-3}	-3.005	0.111	2.08×10^{-3}
C00-D48	Mobility	Enhanced	non-SRTI	45-49	N	LG	0.017	2.32×10^{-4}	0.089	3.47×10^{-3}	-3.339	0.11	6.99×10^{-4}
C00-D48	Mobility	Enhanced	non-SRTI	50-54	Y	LG	0.052	1.13×10^{-3}	0.075	3.55×10^{-3}	-3.082	0.11	2.42×10^{-3}
C00-D48	Mobility	Enhanced	non-SRTI	50-54	N	LG	0.024	4.05×10^{-4}	0.081	3.30×10^{-3}	-3.263	0.106	9.93×10^{-4}
C00-D48	Mobility	Enhanced	non-SRTI	55-59	Y	LG	0.044	1.03×10^{-3}	0.072	3.21×10^{-3}	-3.213	0.102	1.82×10^{-3}
C00-D48	Mobility	Enhanced	non-SRTI	55-59	N	LG	0.027	5.12×10^{-4}	0.078	3.48×10^{-3}	-3.211	0.111	1.19×10^{-3}
C00-D48	Mobility	Enhanced	non-SRTI	60-64	Y	LG	0.045	7.86×10^{-4}	0.077	2.77×10^{-3}	-3.395	0.092	1.57×10^{-3}
C00-D48	Mobility	Enhanced	non-SRTI	60-64	N	LG	0.039	6.17×10^{-4}	0.082	3.10×10^{-3}	-3.351	0.101	1.50×10^{-3}
C00-D48	Mobility	Enhanced	non-SRTI	65-69	Y	LG	0.021	2.61×10^{-4}	0.093	2.20×10^{-3}	-4.896	0.092	4.34×10^{-4}
C00-D48	Mobility	Enhanced	non-SRTI	65-69	N	LG	0.022	2.16×10^{-4}	0.095	1.97×10^{-3}	-4.856	0.082	4.06×10^{-4}
C00-D48	Mobility	Enhanced	non-SRTI	70+	Y	LG	1.09×10^{-3}	2.12×10^{-5}	0.255	8.88×10^{-3}	-18.348	0.598	1.96×10^{-5}
C00-D48	Mobility	Enhanced	non-SRTI	70+	N	LG	1.37×10^{-3}	4.07×10^{-5}	0.22	9.73×10^{-3}	-15.936	0.641	2.98×10^{-5}
C00-D48	Daily living	Standard	SRTI	16-17	Y	LM	8.39×10^{-3}	—	-0.012	8.16×10^{-3}	0.813	0.421	0.651
C00-D48	Daily living	Standard	SRTI	16-17	N	LM	0.013	—	-0.012	8×10^{-3}	0.448	0.453	0.84
C00-D48	Daily living	Standard	SRTI	18-19	Y	LG	5.20×10^{-3}	2.03×10^{-4}	0.536	0.25	-11.133	5.193	1.51×10^{-3}
C00-D48	Daily living	Standard	SRTI	18-19	N	LM	0.018	—	3.63×10^{-3}	2.92×10^{-3}	-0.803	0.159	0.413
C00-D48	Daily living	Standard	SRTI	20-24	Y	LG	5.25×10^{-3}	9.29×10^{-5}	0.376	0.07	-4.858	0.918	7.19×10^{-4}
C00-D48	Daily living	Standard	SRTI	20-24	N	LG	6.84×10^{-3}	1.14×10^{-4}	0.256	0.035	-4.337	0.597	8.13×10^{-4}
C00-D48	Daily living	Standard	SRTI	25-29	Y	LG	0.012	1.71×10^{-4}	0.192	0.022	-2.458	0.294	1.21×10^{-3}
C00-D48	Daily living	Standard	SRTI	25-29	N	LG	8.75×10^{-3}	1.32×10^{-4}	0.131	0.012	-2.011	0.2	8.15×10^{-4}
C00-D48	Daily living	Standard	SRTI	30-34	Y	LG	0.018	1.29×10^{-4}	0.206	0.011	-2.773	0.159	9.19×10^{-4}
C00-D48	Daily living	Standard	SRTI	30-34	N	LG	0.012	1.40×10^{-4}	0.117	6.97×10^{-3}	-2.269	0.137	7.77×10^{-4}
C00-D48	Daily living	Standard	SRTI	35-39	Y	LG	0.028	1.92×10^{-4}	0.167	7.75×10^{-3}	-2.573	0.124	1.28×10^{-3}
C00-D48	Daily living	Standard	SRTI	35-39	N	LG	0.018	1.69×10^{-4}	0.122	6.14×10^{-3}	-2.499	0.126	9.52×10^{-4}
C00-D48	Daily living	Standard	SRTI	40-44	Y	LG	0.033	2.14×10^{-4}	0.152	6.48×10^{-3}	-2.395	0.107	1.39×10^{-3}
C00-D48	Daily living	Standard	SRTI	40-44	N	LG	0.019	1.89×10^{-4}	0.14	9.00×10^{-3}	-2.052	0.142	1.21×10^{-3}
C00-D48	Daily living	Standard	SRTI	45-49	Y	LG	0.035	1.97×10^{-4}	0.16	6.05×10^{-3}	-2.443	0.097	1.31×10^{-3}
C00-D48	Daily living	Standard	SRTI	45-49	N	LG	0.021	1.23×10^{-4}	0.179	7.82×10^{-3}	-2.423	0.112	8.54×10^{-4}
C00-D48	Daily living	Standard	SRTI	50-54	Y	LG	0.045	2.23×10^{-4}	0.162	5.39×10^{-3}	-2.535	0.088	1.47×10^{-3}

Continued on next page

Table 1 – continued from previous page

ICD-10 code	Component	Level	Rules	Ages	Female	Model type	A	s.e.	r	s.e.	k	s.e	RSE
C00-D48	Daily living	Standard	SRTI	50-54	N	LG	0.032	2.04×10^{-4}	0.156	6.88×10^{-3}	-2.12	0.101	1.36×10^{-3}
C00-D48	Daily living	Standard	SRTI	55-59	Y	LG	0.039	2.03×10^{-4}	0.17	6.17×10^{-3}	-2.476	0.094	1.38×10^{-3}
C00-D48	Daily living	Standard	SRTI	55-59	N	LG	0.034	1.73×10^{-4}	0.191	7.55×10^{-3}	-2.391	0.101	1.23×10^{-3}
C00-D48	Daily living	Standard	SRTI	60-64	Y	LG	0.046	2.44×10^{-4}	0.156	5.64×10^{-3}	-2.297	0.088	1.61×10^{-3}
C00-D48	Daily living	Standard	SRTI	60-64	N	LG	0.049	2.90×10^{-4}	0.173	7.45×10^{-3}	-2.258	0.104	2.00×10^{-3}
C00-D48	Daily living	Standard	SRTI	65-69	Y	LG	0.012	8.06×10^{-5}	0.108	2.75×10^{-3}	-3.338	0.079	3.49×10^{-4}
C00-D48	Daily living	Standard	SRTI	65-69	N	LG	0.013	8.69×10^{-5}	0.119	3.53×10^{-3}	-3.506	0.098	4.15×10^{-4}
C00-D48	Daily living	Standard	SRTI	70+	Y	LG	2.75×10^{-4}	8.76×10^{-6}	0.254	0.019	-17.52	1.245	1.23×10^{-5}
C00-D48	Daily living	Standard	SRTI	70+	N	LG	2.99×10^{-4}	1.07×10^{-5}	0.255	0.016	-18.369	1.098	9.74×10^{-6}
C00-D48	Daily living	Standard	non-SRTI	16-17	Y	LG	0.054	9.68×10^{-4}	0.156	0.015	-4.098	0.393	5.53×10^{-3}
C00-D48	Daily living	Standard	non-SRTI	16-17	N	LG	0.059	8.56×10^{-4}	0.169	0.014	-4.483	0.369	5.01×10^{-3}
C00-D48	Daily living	Standard	non-SRTI	18-19	Y	LG	0.053	9.86×10^{-4}	0.091	5.53×10^{-3}	-3.054	0.162	3.48×10^{-3}
C00-D48	Daily living	Standard	non-SRTI	18-19	N	LG	0.057	7.78×10^{-4}	0.12	6.74×10^{-3}	-3.84	0.203	3.54×10^{-3}
C00-D48	Daily living	Standard	non-SRTI	20-24	Y	LG	0.036	3.60×10^{-4}	0.126	5.46×10^{-3}	-3.849	0.159	1.73×10^{-3}
C00-D48	Daily living	Standard	non-SRTI	20-24	N	LG	0.047	1.21×10^{-3}	0.069	3.38×10^{-3}	-2.959	0.102	2.15×10^{-3}
C00-D48	Daily living	Standard	non-SRTI	25-29	Y	LG	0.034	5.66×10^{-4}	0.082	3.83×10^{-3}	-2.877	0.112	1.69×10^{-3}
C00-D48	Daily living	Standard	non-SRTI	25-29	N	LG	0.029	8.88×10^{-4}	0.07	4.68×10^{-3}	-2.737	0.134	1.86×10^{-3}
C00-D48	Daily living	Standard	non-SRTI	30-34	Y	LG	0.038	5.71×10^{-4}	0.082	3.45×10^{-3}	-2.981	0.103	1.65×10^{-3}
C00-D48	Daily living	Standard	non-SRTI	30-34	N	LG	0.024	4.22×10^{-4}	0.078	3.41×10^{-3}	-3.017	0.104	1.07×10^{-3}
C00-D48	Daily living	Standard	non-SRTI	35-39	Y	LG	0.042	7.49×10^{-4}	0.082	3.70×10^{-3}	-3.168	0.116	1.96×10^{-3}
C00-D48	Daily living	Standard	non-SRTI	35-39	N	LG	0.025	8.19×10^{-4}	0.066	3.77×10^{-3}	-2.944	0.112	1.27×10^{-3}
C00-D48	Daily living	Standard	non-SRTI	40-44	Y	LG	0.041	4.95×10^{-4}	0.095	3.92×10^{-3}	-3.123	0.115	1.85×10^{-3}
C00-D48	Daily living	Standard	non-SRTI	40-44	N	LG	0.019	2.03×10^{-4}	0.11	4.72×10^{-3}	-3.313	0.133	9.00×10^{-4}
C00-D48	Daily living	Standard	non-SRTI	45-49	Y	LG	0.04	5.26×10^{-4}	0.094	4.27×10^{-3}	-2.992	0.121	1.99×10^{-3}
C00-D48	Daily living	Standard	non-SRTI	45-49	N	LG	0.02	2.78×10^{-4}	0.091	4.07×10^{-3}	-3.094	0.121	9.58×10^{-4}
C00-D48	Daily living	Standard	non-SRTI	50-54	Y	LG	0.046	7.28×10^{-4}	0.087	4.28×10^{-3}	-2.944	0.124	2.42×10^{-3}
C00-D48	Daily living	Standard	non-SRTI	50-54	N	LG	0.024	2.93×10^{-4}	0.101	4.58×10^{-3}	-3.126	0.129	1.21×10^{-3}
C00-D48	Daily living	Standard	non-SRTI	55-59	Y	LG	0.037	6.25×10^{-4}	0.082	3.87×10^{-3}	-2.922	0.114	1.83×10^{-3}
C00-D48	Daily living	Standard	non-SRTI	55-59	N	LG	0.026	3.10×10^{-4}	0.093	3.68×10^{-3}	-3.057	0.107	1.13×10^{-3}
C00-D48	Daily living	Standard	non-SRTI	60-64	Y	LG	0.039	4.64×10^{-4}	0.09	3.25×10^{-3}	-3.21	0.1	1.51×10^{-3}
C00-D48	Daily living	Standard	non-SRTI	60-64	N	LG	0.035	4.23×10^{-4}	0.093	3.66×10^{-3}	-3.045	0.106	1.53×10^{-3}
C00-D48	Daily living	Standard	non-SRTI	65-69	Y	LG	0.018	1.79×10^{-4}	0.106	2.70×10^{-3}	-4.981	0.11	4.51×10^{-4}
C00-D48	Daily living	Standard	non-SRTI	65-69	N	LG	0.02	1.39×10^{-4}	0.102	1.82×10^{-3}	-4.749	0.073	3.44×10^{-4}
C00-D48	Daily living	Standard	non-SRTI	70+	Y	LG	1.19×10^{-3}	2.75×10^{-5}	0.235	9.24×10^{-3}	-16.897	0.614	2.41×10^{-5}
C00-D48	Daily living	Standard	non-SRTI	70+	N	LG	1.46×10^{-3}	3.77×10^{-5}	0.232	0.011	-16.553	0.702	3.55×10^{-5}
C00-D48	Mobility	Standard	non-SRTI	16-17	Y	LM	0.041	—	1.03×10^{-3}	3.49×10^{-3}	-0.812	0.188	0.516
C00-D48	Mobility	Standard	non-SRTI	16-17	N	LG	0.01	9.22×10^{-4}	-0.181	0.355	15.326	27.949	7.00×10^{-3}
C00-D48	Mobility	Standard	non-SRTI	18-19	Y	LM	0.037	—	9.36×10^{-3}	3.18×10^{-3}	-1.133	0.171	0.47
C00-D48	Mobility	Standard	non-SRTI	18-19	N	LG	0.015	1.99×10^{-4}	0.607	0.101	-14.218	2.37	1.45×10^{-3}
C00-D48	Mobility	Standard	non-SRTI	20-24	Y	LG	0.011	1.36×10^{-4}	0.271	0.027	-5.928	0.595	9.33×10^{-4}
C00-D48	Mobility	Standard	non-SRTI	20-24	N	LG	0.013	1.95×10^{-4}	0.235	0.026	-6.241	0.68	1.24×10^{-3}
C00-D48	Mobility	Standard	non-SRTI	25-29	Y	LG	0.012	1.79×10^{-4}	0.212	0.021	-5.035	0.491	1.15×10^{-3}
C00-D48	Mobility	Standard	non-SRTI	25-29	N	LG	9.97×10^{-3}	1.09×10^{-4}	0.166	0.01	-4.473	0.27	6.29×10^{-4}
C00-D48	Mobility	Standard	non-SRTI	30-34	Y	LG	0.015	1.58×10^{-4}	0.201	0.013	-5.041	0.335	9.89×10^{-4}
C00-D48	Mobility	Standard	non-SRTI	30-34	N	LG	7.20×10^{-3}	1.18×10^{-4}	0.221	0.026	-4.914	0.57	7.78×10^{-4}
C00-D48	Mobility	Standard	non-SRTI	35-39	Y	LG	0.018	2.38×10^{-4}	0.184	0.015	-4.486	0.359	1.47×10^{-3}
C00-D48	Mobility	Standard	non-SRTI	35-39	N	LG	8.12×10^{-3}	8.09×10^{-5}	0.195	0.013	-4.707	0.301	5.09×10^{-4}
C00-D48	Mobility	Standard	non-SRTI	40-44	Y	LG	0.019	9.99×10^{-5}	0.244	9.63×10^{-3}	-5.562	0.219	6.68×10^{-4}
C00-D48	Mobility	Standard	non-SRTI	40-44	N	LG	8.37×10^{-3}	7.07×10^{-5}	0.233	0.014	-5.396	0.331	4.67×10^{-4}

Continued on next page

Table 1 – continued from previous page

ICD-10 code	Component	Level	Rules	Ages	Female	Model type	A	s.e.	r	s.e.	k	s.e	RSE
C00-D48	Mobility	Standard	non-SRTI	45-49	Y	LG	0.02	1.16×10^{-4}	0.225	9.37×10^{-3}	-5.113	0.213	7.65×10^{-4}
C00-D48	Mobility	Standard	non-SRTI	45-49	N	LG	8.50×10^{-3}	6.47×10^{-5}	0.204	0.01	-4.794	0.243	4.15×10^{-4}
C00-D48	Mobility	Standard	non-SRTI	50-54	Y	LG	0.024	1.62×10^{-4}	0.163	6.27×10^{-3}	-4.216	0.16	9.46×10^{-4}
C00-D48	Mobility	Standard	non-SRTI	50-54	N	LG	0.012	6.90×10^{-5}	0.191	7.02×10^{-3}	-4.503	0.165	4.35×10^{-4}
C00-D48	Mobility	Standard	non-SRTI	55-59	Y	LG	0.019	1.93×10^{-4}	0.152	8.31×10^{-3}	-3.939	0.211	1.10×10^{-3}
C00-D48	Mobility	Standard	non-SRTI	55-59	N	LG	0.013	6.41×10^{-5}	0.201	6.68×10^{-3}	-4.771	0.158	4.07×10^{-4}
C00-D48	Mobility	Standard	non-SRTI	60-64	Y	LG	0.021	2.28×10^{-4}	0.126	6.25×10^{-3}	-3.64	0.173	1.14×10^{-3}
C00-D48	Mobility	Standard	non-SRTI	60-64	N	LG	0.02	1.53×10^{-4}	0.15	6.15×10^{-3}	-3.978	0.16	8.59×10^{-4}
C00-D48	Mobility	Standard	non-SRTI	65-69	Y	LG	0.011	1.10×10^{-4}	0.095	2.10×10^{-3}	-4.515	0.083	2.42×10^{-4}
C00-D48	Mobility	Standard	non-SRTI	65-69	N	LG	0.012	6.17×10^{-5}	0.102	1.39×10^{-3}	-4.599	0.054	1.62×10^{-4}
C00-D48	Mobility	Standard	non-SRTI	70+	Y	LG	6.86×10^{-4}	1.73×10^{-5}	0.242	9.98×10^{-3}	-17.535	0.669	1.41×10^{-5}
C00-D48	Mobility	Standard	non-SRTI	70+	N	LG	8.28×10^{-4}	1.90×10^{-5}	0.25	0.011	-17.863	0.729	1.93×10^{-5}
D50-D89	Daily living	Enhanced	SRTI	16-17	Y	No model - fewer than 3 periods with positive claims							
D50-D89	Daily living	Enhanced	SRTI	16-17	N	No model - fewer than 3 periods with positive claims							
D50-D89	Daily living	Enhanced	SRTI	18-19	Y	No model - fewer than 3 periods with positive claims							
D50-D89	Daily living	Enhanced	SRTI	18-19	N	No model - fewer than 3 periods with positive claims							
D50-D89	Daily living	Enhanced	SRTI	20-24	Y	No model - fewer than 3 periods with positive claims							
D50-D89	Daily living	Enhanced	SRTI	20-24	N	No model - fewer than 3 periods with positive claims							
D50-D89	Daily living	Enhanced	SRTI	25-29	Y	No model - fewer than 3 periods with positive claims							
D50-D89	Daily living	Enhanced	SRTI	25-29	N	No model - fewer than 3 periods with positive claims							
D50-D89	Daily living	Enhanced	SRTI	30-34	Y	No model - fewer than 3 periods with positive claims							
D50-D89	Daily living	Enhanced	SRTI	30-34	N	No model - fewer than 3 periods with positive claims							
D50-D89	Daily living	Enhanced	SRTI	35-39	Y	No model - fewer than 3 periods with positive claims							
D50-D89	Daily living	Enhanced	SRTI	35-39	N	No model - fewer than 3 periods with positive claims							
D50-D89	Daily living	Enhanced	SRTI	40-44	Y	No model - fewer than 3 periods with positive claims							
D50-D89	Daily living	Enhanced	SRTI	40-44	N	No model - fewer than 3 periods with positive claims							
D50-D89	Daily living	Enhanced	SRTI	45-49	Y	LM	1.73×10^{-4}	—	-0.07	0.011	6.678	0.528	0.178
D50-D89	Daily living	Enhanced	SRTI	45-49	N	No model - fewer than 3 periods with positive claims							
D50-D89	Daily living	Enhanced	SRTI	50-54	Y	No model - fewer than 3 periods with positive claims							
D50-D89	Daily living	Enhanced	SRTI	50-54	N	No model - fewer than 3 periods with positive claims							
D50-D89	Daily living	Enhanced	SRTI	55-59	Y	LM	1.86×10^{-4}	—	-0.169	9.59×10^{-3}	15.322	0.685	0.092
D50-D89	Daily living	Enhanced	SRTI	55-59	N	No model - fewer than 3 periods with positive claims							
D50-D89	Daily living	Enhanced	SRTI	60-64	Y	No model - fewer than 3 periods with positive claims							
D50-D89	Daily living	Enhanced	SRTI	60-64	N	LM	4.97×10^{-4}	—	-0.141	1.30×10^{-3}	7.515	0.045	0.019
D50-D89	Daily living	Enhanced	SRTI	65-69	Y	No model - fewer than 3 periods with positive claims							
D50-D89	Daily living	Enhanced	SRTI	65-69	N	No model - fewer than 3 periods with positive claims							
D50-D89	Daily living	Enhanced	SRTI	70+	Y	No model - fewer than 3 periods with positive claims							
D50-D89	Daily living	Enhanced	SRTI	70+	N	No model - fewer than 3 periods with positive claims							
D50-D89	Daily living	Enhanced	non-SRTI	16-17	Y	LG	4.58×10^{-3}	1.60×10^{-4}	0.23	0.052	-7.702	1.704	9.22×10^{-4}
D50-D89	Daily living	Enhanced	non-SRTI	16-17	N	LG	7.28×10^{-3}	2.69×10^{-4}	0.247	0.071	-5.141	1.47	1.84×10^{-3}
D50-D89	Daily living	Enhanced	non-SRTI	18-19	Y	LG	3.40×10^{-3}	1.55×10^{-4}	0.102	0.014	-4.293	0.497	4.66×10^{-4}
D50-D89	Daily living	Enhanced	non-SRTI	18-19	N	LG	5.68×10^{-3}	1.84×10^{-4}	0.121	0.016	-3.758	0.485	8.55×10^{-4}
D50-D89	Daily living	Enhanced	non-SRTI	20-24	Y	LG	3.46×10^{-3}	8.26×10^{-5}	0.113	9.74×10^{-3}	-4.154	0.328	3.22×10^{-4}
D50-D89	Daily living	Enhanced	non-SRTI	20-24	N	LG	7.01×10^{-3}	1.56×10^{-4}	0.102	6.72×10^{-3}	-4.243	0.244	4.79×10^{-4}
D50-D89	Daily living	Enhanced	non-SRTI	25-29	Y	LG	3.30×10^{-3}	2.93×10^{-4}	0.053	6.08×10^{-3}	-2.561	0.148	2.82×10^{-4}
D50-D89	Daily living	Enhanced	non-SRTI	25-29	N	LG	5.69×10^{-3}	2.38×10^{-4}	0.087	7.68×10^{-3}	-4.178	0.291	4.70×10^{-4}
D50-D89	Daily living	Enhanced	non-SRTI	30-34	Y	LG	3.36×10^{-3}	9.15×10^{-5}	0.096	8.68×10^{-3}	-3.284	0.263	3.32×10^{-4}
D50-D89	Daily living	Enhanced	non-SRTI	30-34	N	LG	5.42×10^{-3}	3.01×10^{-4}	0.071	8.10×10^{-3}	-2.994	0.247	5.89×10^{-4}
D50-D89	Daily living	Enhanced	non-SRTI	35-39	Y	LG	3.51×10^{-3}	7.90×10^{-5}	0.101	7.77×10^{-3}	-3.427	0.238	2.98×10^{-4}
D50-D89	Daily living	Enhanced	non-SRTI	35-39	N	LG	5.94×10^{-3}	2.53×10^{-4}	0.076	6.57×10^{-3}	-3.34	0.216	4.93×10^{-4}
D50-D89	Daily living	Enhanced	non-SRTI	40-44	Y	LG	3.85×10^{-3}	1.09×10^{-4}	0.094	8.68×10^{-3}	-3.22	0.261	3.88×10^{-4}
D50-D89	Daily living	Enhanced	non-SRTI	40-44	N	LG	7.71×10^{-3}	3.25×10^{-4}	0.075	6.18×10^{-3}	-3.343	0.203	6.02×10^{-4}

Continued on next page

Table 1 – continued from previous page

ICD-10 code	Component	Level	Rules	Ages	Female	Model type	A	s.e.	r	s.e.	k	s.e	RSE
D50-D89	Daily living	Enhanced	non-SRTI	45-49	Y	LG	3.88×10^{-3}	9.53×10^{-5}	0.087	5.83×10^{-3}	-3.309	0.186	2.75×10^{-4}
D50-D89	Daily living	Enhanced	non-SRTI	45-49	N	LG	7.15×10^{-3}	2.30×10^{-4}	0.082	5.90×10^{-3}	-3.529	0.2	5.10×10^{-4}
D50-D89	Daily living	Enhanced	non-SRTI	50-54	Y	LG	5.30×10^{-3}	1.12×10^{-4}	0.089	5.49×10^{-3}	-3.207	0.17	3.53×10^{-4}
D50-D89	Daily living	Enhanced	non-SRTI	50-54	N	LG	0.01	3.72×10^{-4}	0.074	5.24×10^{-3}	-3.26	0.169	6.98×10^{-4}
D50-D89	Daily living	Enhanced	non-SRTI	55-59	Y	LG	4.84×10^{-3}	9.67×10^{-5}	0.089	4.60×10^{-3}	-3.641	0.157	2.59×10^{-4}
D50-D89	Daily living	Enhanced	non-SRTI	55-59	N	LG	6.87×10^{-3}	3.19×10^{-4}	0.069	5.54×10^{-3}	-3.204	0.175	4.93×10^{-4}
D50-D89	Daily living	Enhanced	non-SRTI	60-64	Y	LG	4.62×10^{-3}	9.41×10^{-5}	0.095	4.72×10^{-3}	-4.302	0.179	2.27×10^{-4}
D50-D89	Daily living	Enhanced	non-SRTI	60-64	N	LG	7.19×10^{-3}	1.64×10^{-4}	0.101	6.73×10^{-3}	-4.162	0.242	5.00×10^{-4}
D50-D89	Daily living	Enhanced	non-SRTI	65-69	Y	LG	3.23×10^{-3}	6.77×10^{-5}	0.097	3.95×10^{-3}	-5.171	0.17	1.14×10^{-4}
D50-D89	Daily living	Enhanced	non-SRTI	65-69	N	LG	5.18×10^{-3}	1.40×10^{-4}	0.108	7.97×10^{-3}	-5.024	0.324	3.75×10^{-4}
D50-D89	Daily living	Enhanced	non-SRTI	70+	Y	LG	2.32×10^{-4}	1.01×10^{-5}	0.225	0.017	-16.03	1.1	9.15×10^{-6}
D50-D89	Daily living	Enhanced	non-SRTI	70+	N	LG	4.45×10^{-4}	1.29×10^{-5}	0.302	0.025	-20.898	1.645	2.17×10^{-5}
D50-D89	Mobility	Enhanced	non-SRTI	16-17	Y	LG	5.42×10^{-3}	1.73×10^{-4}	0.121	0.013	-4.944	0.508	6.29×10^{-4}
D50-D89	Mobility	Enhanced	non-SRTI	16-17	N	LG	0.011	3.94×10^{-4}	0.152	0.021	-6.968	0.922	1.45×10^{-3}
D50-D89	Mobility	Enhanced	non-SRTI	18-19	Y	LG	3.71×10^{-3}	1.68×10^{-4}	0.088	9.26×10^{-3}	-3.977	0.338	3.82×10^{-4}
D50-D89	Mobility	Enhanced	non-SRTI	18-19	N	LG	5.45×10^{-3}	1.65×10^{-4}	0.132	0.016	-5.103	0.573	6.80×10^{-4}
D50-D89	Mobility	Enhanced	non-SRTI	20-24	Y	LG	4.81×10^{-3}	1.25×10^{-4}	0.096	7.37×10^{-3}	-3.71	0.248	3.94×10^{-4}
D50-D89	Mobility	Enhanced	non-SRTI	20-24	N	LG	7.55×10^{-3}	2.16×10^{-4}	0.113	0.01	-4.658	0.388	7.25×10^{-4}
D50-D89	Mobility	Enhanced	non-SRTI	25-29	Y	LG	4.94×10^{-3}	1.31×10^{-4}	0.09	6.22×10^{-3}	-3.736	0.215	3.51×10^{-4}
D50-D89	Mobility	Enhanced	non-SRTI	25-29	N	LG	8.19×10^{-3}	1.72×10^{-4}	0.099	6.05×10^{-3}	-4.025	0.214	5.25×10^{-4}
D50-D89	Mobility	Enhanced	non-SRTI	30-34	Y	LG	6.06×10^{-3}	1.43×10^{-4}	0.088	4.97×10^{-3}	-3.871	0.178	3.40×10^{-4}
D50-D89	Mobility	Enhanced	non-SRTI	30-34	N	LG	0.012	5.19×10^{-4}	0.071	4.49×10^{-3}	-3.812	0.156	5.89×10^{-4}
D50-D89	Mobility	Enhanced	non-SRTI	35-39	Y	LG	4.94×10^{-3}	1.07×10^{-4}	0.087	5.23×10^{-3}	-3.325	0.167	3.12×10^{-4}
D50-D89	Mobility	Enhanced	non-SRTI	35-39	N	LG	8.69×10^{-3}	1.69×10^{-4}	0.105	5.68×10^{-3}	-4.695	0.222	4.72×10^{-4}
D50-D89	Mobility	Enhanced	non-SRTI	40-44	Y	LG	8.05×10^{-3}	1.41×10^{-4}	0.094	4.78×10^{-3}	-3.622	0.159	4.36×10^{-4}
D50-D89	Mobility	Enhanced	non-SRTI	40-44	N	LG	9.76×10^{-3}	3.30×10^{-4}	0.085	6.21×10^{-3}	-3.862	0.224	6.92×10^{-4}
D50-D89	Mobility	Enhanced	non-SRTI	45-49	Y	LG	6.70×10^{-3}	1.22×10^{-4}	0.094	5.09×10^{-3}	-3.611	0.168	3.85×10^{-4}
D50-D89	Mobility	Enhanced	non-SRTI	45-49	N	LG	8.93×10^{-3}	1.37×10^{-4}	0.098	4.46×10^{-3}	-3.846	0.153	4.32×10^{-4}
D50-D89	Mobility	Enhanced	non-SRTI	50-54	Y	LG	8.72×10^{-3}	9.26×10^{-5}	0.101	3.46×10^{-3}	-3.793	0.115	3.21×10^{-4}
D50-D89	Mobility	Enhanced	non-SRTI	50-54	N	LG	0.014	2.06×10^{-4}	0.102	4.01×10^{-3}	-4.571	0.155	5.56×10^{-4}
D50-D89	Mobility	Enhanced	non-SRTI	55-59	Y	LG	8.61×10^{-3}	1.82×10^{-4}	0.079	3.33×10^{-3}	-3.601	0.115	3.45×10^{-4}
D50-D89	Mobility	Enhanced	non-SRTI	55-59	N	LG	0.015	5.22×10^{-4}	0.068	3.31×10^{-3}	-3.589	0.11	5.90×10^{-4}
D50-D89	Mobility	Enhanced	non-SRTI	60-64	Y	LG	9.36×10^{-3}	2.09×10^{-4}	0.077	2.99×10^{-3}	-3.865	0.107	3.18×10^{-4}
D50-D89	Mobility	Enhanced	non-SRTI	60-64	N	LG	0.014	4.32×10^{-4}	0.074	4.24×10^{-3}	-3.386	0.14	7.66×10^{-4}
D50-D89	Mobility	Enhanced	non-SRTI	65-69	Y	LG	5.21×10^{-3}	9.91×10^{-5}	0.108	4.64×10^{-3}	-5.616	0.206	2.01×10^{-4}
D50-D89	Mobility	Enhanced	non-SRTI	65-69	N	LG	0.01	2.19×10^{-4}	0.095	3.68×10^{-3}	-5.2	0.159	3.34×10^{-4}
D50-D89	Mobility	Enhanced	non-SRTI	70+	Y	LG	1.92×10^{-4}	4.98×10^{-6}	0.314	0.023	-21.902	1.533	8.12×10^{-6}
D50-D89	Mobility	Enhanced	non-SRTI	70+	N	LG	6.94×10^{-4}	3.67×10^{-5}	0.247	0.016	-18.638	1.068	1.76×10^{-5}
D50-D89	Daily living	Standard	SRTI	16-17	Y	No model - fewer than 3 periods with positive claims							
D50-D89	Daily living	Standard	SRTI	16-17	N	No model - fewer than 3 periods with positive claims							
D50-D89	Daily living	Standard	SRTI	18-19	Y	No model - fewer than 3 periods with positive claims							
D50-D89	Daily living	Standard	SRTI	18-19	N	No model - fewer than 3 periods with positive claims							
D50-D89	Daily living	Standard	SRTI	20-24	Y	No model - fewer than 3 periods with positive claims							
D50-D89	Daily living	Standard	SRTI	20-24	N	No model - fewer than 3 periods with positive claims							
D50-D89	Daily living	Standard	SRTI	25-29	Y	No model - fewer than 3 periods with positive claims							
D50-D89	Daily living	Standard	SRTI	25-29	N	No model - fewer than 3 periods with positive claims							
D50-D89	Daily living	Standard	SRTI	30-34	Y	No model - fewer than 3 periods with positive claims							
D50-D89	Daily living	Standard	SRTI	30-34	N	No model - fewer than 3 periods with positive claims							
D50-D89	Daily living	Standard	SRTI	35-39	Y	No model - fewer than 3 periods with positive claims							
D50-D89	Daily living	Standard	SRTI	35-39	N	No model - fewer than 3 periods with positive claims							

Continued on next page

Table 1 – continued from previous page

ICD-10 code	Component	Level	Rules	Ages	Female	Model type	A	s.e.	r	s.e.	k	s.e	RSE
D50-D89	Daily living	Standard	SRTI	40-44	Y	No model - fewer than 3 periods with positive claims							
D50-D89	Daily living	Standard	SRTI	40-44	N	No model - fewer than 3 periods with positive claims							
D50-D89	Daily living	Standard	SRTI	45-49	Y	LM	1.73×10^{-4}	—	-0.07	0.011	6.678	0.528	0.178
D50-D89	Daily living	Standard	SRTI	45-49	N	No model - fewer than 3 periods with positive claims							
D50-D89	Daily living	Standard	SRTI	50-54	Y	No model - fewer than 3 periods with positive claims							
D50-D89	Daily living	Standard	SRTI	50-54	N	No model - fewer than 3 periods with positive claims							
D50-D89	Daily living	Standard	SRTI	55-59	Y	LM	1.86×10^{-4}	—	-0.169	9.59×10^{-3}	15.322	0.685	0.092
D50-D89	Daily living	Standard	SRTI	55-59	N	No model - fewer than 3 periods with positive claims							
D50-D89	Daily living	Standard	SRTI	60-64	Y	No model - fewer than 3 periods with positive claims							
D50-D89	Daily living	Standard	SRTI	60-64	N	LM	4.97×10^{-4}	—	-0.141	1.30×10^{-3}	7.515	0.045	0.019
D50-D89	Daily living	Standard	SRTI	65-69	Y	No model - fewer than 3 periods with positive claims							
D50-D89	Daily living	Standard	SRTI	65-69	N	No model - fewer than 3 periods with positive claims							
D50-D89	Daily living	Standard	SRTI	70+	Y	No model - fewer than 3 periods with positive claims							
D50-D89	Daily living	Standard	SRTI	70+	N	No model - fewer than 3 periods with positive claims							
D50-D89	Daily living	Standard	non-SRTI	16-17	Y	LG	4.38×10^{-3}	1.43×10^{-4}	0.197	0.034	-7.769	1.306	7.06×10^{-4}
D50-D89	Daily living	Standard	non-SRTI	16-17	N	LG	7.50×10^{-3}	4.71×10^{-4}	0.089	0.015	-3.533	0.507	1.34×10^{-3}
D50-D89	Daily living	Standard	non-SRTI	18-19	Y	LG	2.66×10^{-3}	1.65×10^{-4}	0.107	0.012	-6.191	0.571	2.37×10^{-4}
D50-D89	Daily living	Standard	non-SRTI	18-19	N	LG	0.015	4.75×10^{-3}	0.052	7.10×10^{-3}	-4.155	0.194	7.15×10^{-4}
D50-D89	Daily living	Standard	non-SRTI	20-24	Y	LG	3.17×10^{-3}	5.47×10^{-5}	0.145	0.011	-5.451	0.388	2.45×10^{-4}
D50-D89	Daily living	Standard	non-SRTI	20-24	N	LG	6.99×10^{-3}	6.09×10^{-4}	0.063	6.88×10^{-3}	-3.354	0.21	5.85×10^{-4}
D50-D89	Daily living	Standard	non-SRTI	25-29	Y	LG	4.50×10^{-3}	2.46×10^{-4}	0.07	4.96×10^{-3}	-3.928	0.173	2.41×10^{-4}
D50-D89	Daily living	Standard	non-SRTI	25-29	N	LG	8.44×10^{-3}	3.72×10^{-4}	0.075	4.77×10^{-3}	-4.074	0.175	4.29×10^{-4}
D50-D89	Daily living	Standard	non-SRTI	30-34	Y	LG	4.73×10^{-3}	1.98×10^{-4}	0.069	4.66×10^{-3}	-3.376	0.151	2.75×10^{-4}
D50-D89	Daily living	Standard	non-SRTI	30-34	N	LG	0.012	1.22×10^{-3}	0.06	4.79×10^{-3}	-3.965	0.141	5.42×10^{-4}
D50-D89	Daily living	Standard	non-SRTI	35-39	Y	LG	3.90×10^{-3}	8.45×10^{-5}	0.093	5.85×10^{-3}	-3.545	0.192	2.62×10^{-4}
D50-D89	Daily living	Standard	non-SRTI	35-39	N	LG	0.011	4.02×10^{-4}	0.08	4.25×10^{-3}	-4.537	0.167	4.44×10^{-4}
D50-D89	Daily living	Standard	non-SRTI	40-44	Y	LG	7.56×10^{-3}	4.29×10^{-4}	0.061	4.21×10^{-3}	-3.318	0.126	3.87×10^{-4}
D50-D89	Daily living	Standard	non-SRTI	40-44	N	LG	0.018	2.07×10^{-3}	0.057	4.53×10^{-3}	-3.913	0.122	7.16×10^{-4}
D50-D89	Daily living	Standard	non-SRTI	45-49	Y	LG	5.16×10^{-3}	7.89×10^{-5}	0.093	3.93×10^{-3}	-3.778	0.136	2.27×10^{-4}
D50-D89	Daily living	Standard	non-SRTI	45-49	N	LG	0.011	2.33×10^{-4}	0.093	4.56×10^{-3}	-4.435	0.178	5.03×10^{-4}
D50-D89	Daily living	Standard	non-SRTI	50-54	Y	LG	7.77×10^{-3}	1.63×10^{-4}	0.08	3.60×10^{-3}	-3.515	0.122	3.41×10^{-4}
D50-D89	Daily living	Standard	non-SRTI	50-54	N	LG	0.017	4.33×10^{-4}	0.076	3.21×10^{-3}	-3.906	0.116	5.97×10^{-4}
D50-D89	Daily living	Standard	non-SRTI	55-59	Y	LG	7.70×10^{-3}	1.67×10^{-4}	0.079	3.45×10^{-3}	-3.671	0.12	3.15×10^{-4}
D50-D89	Daily living	Standard	non-SRTI	55-59	N	LG	0.016	1.23×10^{-3}	0.055	3.40×10^{-3}	-3.501	0.09	5.71×10^{-4}
D50-D89	Daily living	Standard	non-SRTI	60-64	Y	LG	8.25×10^{-3}	2.41×10^{-4}	0.075	3.00×10^{-3}	-4.17	0.111	2.57×10^{-4}
D50-D89	Daily living	Standard	non-SRTI	60-64	N	LG	0.013	3.63×10^{-4}	0.083	4.52×10^{-3}	-3.895	0.164	6.88×10^{-4}
D50-D89	Daily living	Standard	non-SRTI	65-69	Y	LG	4.89×10^{-3}	9.16×10^{-5}	0.096	3.36×10^{-3}	-5.219	0.145	1.45×10^{-4}
D50-D89	Daily living	Standard	non-SRTI	65-69	N	LG	8.88×10^{-3}	2.17×10^{-4}	0.098	4.73×10^{-3}	-5.172	0.203	3.75×10^{-4}
D50-D89	Daily living	Standard	non-SRTI	70+	Y	LG	2.87×10^{-4}	1.84×10^{-5}	0.198	0.014	-14.674	0.927	8.92×10^{-6}
D50-D89	Daily living	Standard	non-SRTI	70+	N	LG	5.72×10^{-4}	1.22×10^{-5}	0.304	0.016	-21.563	1.069	1.63×10^{-5}
D50-D89	Mobility	Standard	non-SRTI	16-17	Y	LM	4.67×10^{-3}	—	0.01	8.20×10^{-3}	-0.251	0.462	1.021
D50-D89	Mobility	Standard	non-SRTI	16-17	N	LG	5.89×10^{-3}	2.52×10^{-4}	0.151	0.033	-4.351	0.931	1.37×10^{-3}
D50-D89	Mobility	Standard	non-SRTI	18-19	Y	LG	2.48×10^{-3}	8.71×10^{-5}	0.178	0.032	-6.49	1.15	4.41×10^{-4}
D50-D89	Mobility	Standard	non-SRTI	18-19	N	LG	4.16×10^{-3}	8.95×10^{-5}	0.188	0.022	-6.512	0.756	4.77×10^{-4}
D50-D89	Mobility	Standard	non-SRTI	20-24	Y	LG	3.31×10^{-3}	1.25×10^{-4}	0.083	7.45×10^{-3}	-3.497	0.251	2.99×10^{-4}
D50-D89	Mobility	Standard	non-SRTI	20-24	N	LG	5.53×10^{-3}	1.99×10^{-4}	0.097	9.58×10^{-3}	-4.071	0.346	5.64×10^{-4}
D50-D89	Mobility	Standard	non-SRTI	25-29	Y	LG	1.91×10^{-3}	3.75×10^{-5}	0.277	0.043	-7.21	1.104	2.47×10^{-4}
D50-D89	Mobility	Standard	non-SRTI	25-29	N	LG	4.05×10^{-3}	1.24×10^{-4}	0.108	0.011	-3.973	0.381	4.64×10^{-4}
D50-D89	Mobility	Standard	non-SRTI	30-34	Y	LG	2.69×10^{-3}	5.08×10^{-5}	0.122	9.87×10^{-3}	-3.763	0.288	2.39×10^{-4}
D50-D89	Mobility	Standard	non-SRTI	30-34	N	LG	4.35×10^{-3}	1.40×10^{-4}	0.106	0.013	-3.223	0.374	6.07×10^{-4}

Continued on next page

Table 1 – continued from previous page

ICD-10 code	Component	Level	Rules	Ages	Female	Model type	A	s.e.	r	s.e.	k	s.e	RSE
D50-D89	Mobility	Standard	non-SRTI	35-39	Y	LG	3.39×10^{-3}	1.53×10^{-4}	0.069	6.41×10^{-3}	-2.766	0.185	3.03×10^{-4}
D50-D89	Mobility	Standard	non-SRTI	35-39	N	LG	3.44×10^{-3}	7.48×10^{-5}	0.16	0.018	-5.265	0.562	3.88×10^{-4}
D50-D89	Mobility	Standard	non-SRTI	40-44	Y	LG	3.69×10^{-3}	7.20×10^{-5}	0.105	7.48×10^{-3}	-3.436	0.224	2.92×10^{-4}
D50-D89	Mobility	Standard	non-SRTI	40-44	N	LG	4.87×10^{-3}	7.96×10^{-5}	0.155	0.013	-4.795	0.388	4.22×10^{-4}
D50-D89	Mobility	Standard	non-SRTI	45-49	Y	LG	3.40×10^{-3}	8.94×10^{-5}	0.093	8.05×10^{-3}	-3.057	0.234	3.24×10^{-4}
D50-D89	Mobility	Standard	non-SRTI	45-49	N	LG	4.29×10^{-3}	1.01×10^{-4}	0.103	9.26×10^{-3}	-3.121	0.259	4.29×10^{-4}
D50-D89	Mobility	Standard	non-SRTI	50-54	Y	LG	4.26×10^{-3}	7.80×10^{-5}	0.099	6.35×10^{-3}	-3.276	0.189	3.00×10^{-4}
D50-D89	Mobility	Standard	non-SRTI	50-54	N	LG	5.45×10^{-3}	1.02×10^{-4}	0.126	0.01	-3.907	0.303	4.87×10^{-4}
D50-D89	Mobility	Standard	non-SRTI	55-59	Y	LG	3.84×10^{-3}	6.70×10^{-5}	0.104	6.14×10^{-3}	-3.7	0.198	2.49×10^{-4}
D50-D89	Mobility	Standard	non-SRTI	55-59	N	LG	5.99×10^{-3}	1.13×10^{-4}	0.113	7.60×10^{-3}	-4.239	0.26	4.32×10^{-4}
D50-D89	Mobility	Standard	non-SRTI	60-64	Y	LG	4.11×10^{-3}	9.88×10^{-5}	0.093	6.44×10^{-3}	-3.544	0.212	3.04×10^{-4}
D50-D89	Mobility	Standard	non-SRTI	60-64	N	LG	6.01×10^{-3}	1.16×10^{-4}	0.116	9.15×10^{-3}	-3.557	0.265	5.28×10^{-4}
D50-D89	Mobility	Standard	non-SRTI	65-69	Y	LG	2.40×10^{-3}	3.98×10^{-5}	0.17	0.011	-8.153	0.517	1.48×10^{-4}
D50-D89	Mobility	Standard	non-SRTI	65-69	N	LG	4.98×10^{-3}	2.18×10^{-4}	0.074	5.44×10^{-3}	-3.689	0.189	3.19×10^{-4}
D50-D89	Mobility	Standard	non-SRTI	70+	Y	LG	1.37×10^{-4}	4.07×10^{-6}	0.302	0.025	-20.873	1.678	6.85×10^{-6}
D50-D89	Mobility	Standard	non-SRTI	70+	N	LG	1.32×10^{-3}	7.30×10^{-4}	0.163	0.021	-13.95	0.978	2.20×10^{-5}
E00-E90	Daily living	Enhanced	SRTI	16-17	Y	No model - fewer than 3 periods with positive claims							
E00-E90	Daily living	Enhanced	SRTI	16-17	N	No model - fewer than 3 periods with positive claims							
E00-E90	Daily living	Enhanced	SRTI	18-19	Y	No model - fewer than 3 periods with positive claims							
E00-E90	Daily living	Enhanced	SRTI	18-19	N	No model - fewer than 3 periods with positive claims							
E00-E90	Daily living	Enhanced	SRTI	20-24	Y	No model - fewer than 3 periods with positive claims							
E00-E90	Daily living	Enhanced	SRTI	20-24	N	No model - fewer than 3 periods with positive claims							
E00-E90	Daily living	Enhanced	SRTI	25-29	Y	No model - fewer than 3 periods with positive claims							
E00-E90	Daily living	Enhanced	SRTI	25-29	N	No model - fewer than 3 periods with positive claims							
E00-E90	Daily living	Enhanced	SRTI	30-34	Y	LM	1.95×10^{-4}	—	0.032	0.02	-1.463	1.343	1.121
E00-E90	Daily living	Enhanced	SRTI	30-34	N	No model - fewer than 3 periods with positive claims							
E00-E90	Daily living	Enhanced	SRTI	35-39	Y	LG	1.06×10^{-15}	8.14×10^{-6}	14.053	1.74727E+14	-1119.899	1.39781E+16	1.15×10^{-5}
E00-E90	Daily living	Enhanced	SRTI	35-39	N	No model - fewer than 3 periods with positive claims							
E00-E90	Daily living	Enhanced	SRTI	40-44	Y	LM	5.36×10^{-5}	—	-0.029	9.33×10^{-5}	4.001	7.42×10^{-3}	3.90×10^{-4}
E00-E90	Daily living	Enhanced	SRTI	40-44	N	No model - fewer than 3 periods with positive claims							
E00-E90	Daily living	Enhanced	SRTI	45-49	Y	LM	2.53×10^{-5}	—	-0.346	0.03	31.762	2.27	0.043
E00-E90	Daily living	Enhanced	SRTI	45-49	N	No model - fewer than 3 periods with positive claims							
E00-E90	Daily living	Enhanced	SRTI	50-54	Y	No model - fewer than 3 periods with positive claims							
E00-E90	Daily living	Enhanced	SRTI	50-54	N	LM	3.68×10^{-5}	—	0.029	0.023	-0.121	1.002	0.711
E00-E90	Daily living	Enhanced	SRTI	55-59	Y	LG	1.36×10^{-5}	1.52×10^{-6}	0.327	0.263	-13.37	10.683	8.37×10^{-6}
E00-E90	Daily living	Enhanced	SRTI	55-59	N	LM	4.98×10^{-5}	—	0.012	0.017	-0.888	1.001	0.83
E00-E90	Daily living	Enhanced	SRTI	60-64	Y	LG	7.19×10^{-5}	1.94×10^{-5}	0.096	0.022	-6.679	1.033	8.56×10^{-6}
E00-E90	Daily living	Enhanced	SRTI	60-64	N	LG	6.40×10^{-5}	6.18×10^{-6}	0.162	0.042	-9.53	2.263	1.31×10^{-5}
E00-E90	Daily living	Enhanced	SRTI	65-69	Y	LM	2.79×10^{-5}	—	0.013	6.57×10^{-3}	-0.653	0.384	0.576
E00-E90	Daily living	Enhanced	SRTI	65-69	N	LM	2.00×10^{-5}	—	-9.20×10^{-5}	9.71×10^{-3}	1.267	0.579	0.577
E00-E90	Daily living	Enhanced	SRTI	70+	Y	No model - fewer than 3 periods with positive claims							
E00-E90	Daily living	Enhanced	SRTI	70+	N	No model - fewer than 3 periods with positive claims							
E00-E90	Daily living	Enhanced	non-SRTI	16-17	Y	LG	0.014	1.11×10^{-4}	0.264	0.016	-6.524	0.39	7.34×10^{-4}
E00-E90	Daily living	Enhanced	non-SRTI	16-17	N	LG	0.015	2.10×10^{-4}	0.287	0.032	-6.977	0.777	1.41×10^{-3}
E00-E90	Daily living	Enhanced	non-SRTI	18-19	Y	LG	9.07×10^{-3}	1.11×10^{-4}	0.12	5.55×10^{-3}	-4.342	0.188	4.54×10^{-4}
E00-E90	Daily living	Enhanced	non-SRTI	18-19	N	LG	0.01	1.24×10^{-4}	0.153	8.77×10^{-3}	-5.454	0.302	5.93×10^{-4}
E00-E90	Daily living	Enhanced	non-SRTI	20-24	Y	LG	0.01	1.19×10^{-4}	0.092	2.98×10^{-3}	-3.687	0.101	3.45×10^{-4}
E00-E90	Daily living	Enhanced	non-SRTI	20-24	N	LG	0.011	1.76×10^{-4}	0.086	2.88×10^{-3}	-4.081	0.108	3.40×10^{-4}
E00-E90	Daily living	Enhanced	non-SRTI	25-29	Y	LG	6.01×10^{-3}	9.29×10^{-5}	0.085	3.15×10^{-3}	-3.645	0.109	2.23×10^{-4}
E00-E90	Daily living	Enhanced	non-SRTI	25-29	N	LG	5.16×10^{-3}	8.30×10^{-5}	0.09	3.21×10^{-3}	-4.217	0.122	1.77×10^{-4}
E00-E90	Daily living	Enhanced	non-SRTI	30-34	Y	LG	5.94×10^{-3}	1.30×10^{-4}	0.073	3.07×10^{-3}	-3.258	0.099	2.34×10^{-4}

Continued on next page

Table 1 – continued from previous page

ICD-10 code	Component	Level	Rules	Ages	Female	Model type	A	s.e.	r	s.e.	k	s.e	RSE
E00-E90	Daily living	Enhanced	non-SRTI	30-34	N	LG	5.51×10^{-3}	1.40×10^{-4}	0.072	3.31×10^{-3}	-3.359	0.108	2.30×10^{-4}
E00-E90	Daily living	Enhanced	non-SRTI	35-39	Y	LG	2.95×10^{-3}	6.07×10^{-5}	0.082	4.36×10^{-3}	-3.159	0.136	1.63×10^{-4}
E00-E90	Daily living	Enhanced	non-SRTI	35-39	N	LG	3.16×10^{-3}	1.26×10^{-4}	0.064	3.80×10^{-3}	-3.109	0.114	1.57×10^{-4}
E00-E90	Daily living	Enhanced	non-SRTI	40-44	Y	LG	2.64×10^{-3}	4.77×10^{-5}	0.088	4.85×10^{-3}	-3.022	0.143	1.58×10^{-4}
E00-E90	Daily living	Enhanced	non-SRTI	40-44	N	LG	2.75×10^{-3}	4.87×10^{-5}	0.091	5.21×10^{-3}	-3.057	0.153	1.71×10^{-4}
E00-E90	Daily living	Enhanced	non-SRTI	45-49	Y	LG	2.04×10^{-3}	3.46×10^{-5}	0.096	5.90×10^{-3}	-2.907	0.162	1.39×10^{-4}
E00-E90	Daily living	Enhanced	non-SRTI	45-49	N	LG	2.15×10^{-3}	3.16×10^{-5}	0.12	7.87×10^{-3}	-3.348	0.21	1.56×10^{-4}
E00-E90	Daily living	Enhanced	non-SRTI	50-54	Y	LG	2.56×10^{-3}	4.85×10^{-5}	0.088	5.17×10^{-3}	-2.922	0.149	1.64×10^{-4}
E00-E90	Daily living	Enhanced	non-SRTI	50-54	N	LG	2.83×10^{-3}	4.57×10^{-5}	0.099	5.87×10^{-3}	-3.067	0.165	1.86×10^{-4}
E00-E90	Daily living	Enhanced	non-SRTI	55-59	Y	LG	2.13×10^{-3}	3.07×10^{-5}	0.095	4.58×10^{-3}	-3.134	0.134	1.13×10^{-4}
E00-E90	Daily living	Enhanced	non-SRTI	55-59	N	LG	2.45×10^{-3}	4.52×10^{-5}	0.098	6.59×10^{-3}	-2.982	0.182	1.83×10^{-4}
E00-E90	Daily living	Enhanced	non-SRTI	60-64	Y	LG	2.48×10^{-3}	5.71×10^{-5}	0.079	4.82×10^{-3}	-2.842	0.141	1.58×10^{-4}
E00-E90	Daily living	Enhanced	non-SRTI	60-64	N	LG	3.24×10^{-3}	5.36×10^{-5}	0.095	5.34×10^{-3}	-3.123	0.156	2.00×10^{-4}
E00-E90	Daily living	Enhanced	non-SRTI	65-69	Y	LG	1.59×10^{-3}	1.28×10^{-5}	0.107	2.19×10^{-3}	-5.164	0.091	3.11×10^{-5}
E00-E90	Daily living	Enhanced	non-SRTI	65-69	N	LG	2.26×10^{-3}	1.18×10^{-5}	0.124	1.92×10^{-3}	-5.92	0.083	3.39×10^{-5}
E00-E90	Daily living	Enhanced	non-SRTI	70+	Y	LG	1.80×10^{-4}	3.68×10^{-6}	0.246	8.19×10^{-3}	-17.889	0.552	2.95×10^{-6}
E00-E90	Daily living	Enhanced	non-SRTI	70+	N	LG	2.96×10^{-4}	4.62×10^{-6}	0.259	7.76×10^{-3}	-18.601	0.523	4.65×10^{-6}
E00-E90	Mobility	Enhanced	non-SRTI	16-17	Y	LG	0.011	1.77×10^{-4}	0.181	0.018	-4.481	0.436	1.08×10^{-3}
E00-E90	Mobility	Enhanced	non-SRTI	16-17	N	LG	0.012	2.03×10^{-4}	0.185	0.019	-5.369	0.536	1.18×10^{-3}
E00-E90	Mobility	Enhanced	non-SRTI	18-19	Y	LG	6.60×10^{-3}	9.01×10^{-5}	0.121	6.74×10^{-3}	-4.008	0.21	4.02×10^{-4}
E00-E90	Mobility	Enhanced	non-SRTI	18-19	N	LG	6.24×10^{-3}	8.70×10^{-5}	0.127	6.56×10^{-3}	-5.095	0.245	3.36×10^{-4}
E00-E90	Mobility	Enhanced	non-SRTI	20-24	Y	LG	6.79×10^{-3}	1.63×10^{-4}	0.092	6.75×10^{-3}	-3.302	0.21	5.38×10^{-4}
E00-E90	Mobility	Enhanced	non-SRTI	20-24	N	LG	7.36×10^{-3}	2.40×10^{-4}	0.068	3.45×10^{-3}	-3.328	0.11	3.19×10^{-4}
E00-E90	Mobility	Enhanced	non-SRTI	25-29	Y	LG	4.92×10^{-3}	7.09×10^{-5}	0.091	3.57×10^{-3}	-3.601	0.12	2.03×10^{-4}
E00-E90	Mobility	Enhanced	non-SRTI	25-29	N	LG	4.83×10^{-3}	1.39×10^{-4}	0.074	4.00×10^{-3}	-3.392	0.132	2.42×10^{-4}
E00-E90	Mobility	Enhanced	non-SRTI	30-34	Y	LG	5.72×10^{-3}	8.64×10^{-5}	0.083	2.89×10^{-3}	-3.595	0.099	1.97×10^{-4}
E00-E90	Mobility	Enhanced	non-SRTI	30-34	N	LG	5.48×10^{-3}	1.61×10^{-4}	0.071	3.26×10^{-3}	-3.621	0.111	2.11×10^{-4}
E00-E90	Mobility	Enhanced	non-SRTI	35-39	Y	LG	3.75×10^{-3}	7.95×10^{-5}	0.08	4.13×10^{-3}	-3.131	0.129	1.98×10^{-4}
E00-E90	Mobility	Enhanced	non-SRTI	35-39	N	LG	2.82×10^{-3}	4.51×10^{-5}	0.09	4.42×10^{-3}	-3.121	0.133	1.50×10^{-4}
E00-E90	Mobility	Enhanced	non-SRTI	40-44	Y	LG	3.78×10^{-3}	3.68×10^{-5}	0.125	5.29×10^{-3}	-3.805	0.154	1.76×10^{-4}
E00-E90	Mobility	Enhanced	non-SRTI	40-44	N	LG	3.55×10^{-3}	4.18×10^{-5}	0.12	6.05×10^{-3}	-3.555	0.171	1.99×10^{-4}
E00-E90	Mobility	Enhanced	non-SRTI	45-49	Y	LG	3.04×10^{-3}	4.28×10^{-5}	0.105	5.50×10^{-3}	-3.319	0.16	1.77×10^{-4}
E00-E90	Mobility	Enhanced	non-SRTI	45-49	N	LG	3.30×10^{-3}	4.21×10^{-5}	0.102	4.66×10^{-3}	-3.357	0.139	1.67×10^{-4}
E00-E90	Mobility	Enhanced	non-SRTI	50-54	Y	LG	3.74×10^{-3}	6.16×10^{-5}	0.094	5.17×10^{-3}	-3.141	0.152	2.25×10^{-4}
E00-E90	Mobility	Enhanced	non-SRTI	50-54	N	LG	3.98×10^{-3}	6.37×10^{-5}	0.109	6.85×10^{-3}	-3.305	0.194	2.80×10^{-4}
E00-E90	Mobility	Enhanced	non-SRTI	55-59	Y	LG	3.01×10^{-3}	5.50×10^{-5}	0.093	5.62×10^{-3}	-3.084	0.164	1.99×10^{-4}
E00-E90	Mobility	Enhanced	non-SRTI	55-59	N	LG	3.83×10^{-3}	5.67×10^{-5}	0.101	5.47×10^{-3}	-3.175	0.157	2.30×10^{-4}
E00-E90	Mobility	Enhanced	non-SRTI	60-64	Y	LG	3.94×10^{-3}	6.64×10^{-5}	0.09	4.52×10^{-3}	-3.223	0.139	2.14×10^{-4}
E00-E90	Mobility	Enhanced	non-SRTI	60-64	N	LG	5.22×10^{-3}	7.00×10^{-5}	0.101	4.72×10^{-3}	-3.377	0.143	2.69×10^{-4}
E00-E90	Mobility	Enhanced	non-SRTI	65-69	Y	LG	2.44×10^{-3}	2.47×10^{-5}	0.101	2.37×10^{-3}	-4.99	0.099	5.35×10^{-5}
E00-E90	Mobility	Enhanced	non-SRTI	65-69	N	LG	3.59×10^{-3}	2.75×10^{-5}	0.105	1.98×10^{-3}	-5.058	0.082	6.48×10^{-5}
E00-E90	Mobility	Enhanced	non-SRTI	70+	Y	LG	2.26×10^{-4}	7.22×10^{-6}	0.233	0.013	-16.592	0.885	6.97×10^{-6}
E00-E90	Mobility	Enhanced	non-SRTI	70+	N	LG	3.76×10^{-4}	8.72×10^{-6}	0.245	0.011	-17.28	0.756	9.77×10^{-6}
E00-E90	Daily living	Standard	SRTI	16-17	Y	No model - fewer than 3 periods with positive claims							
E00-E90	Daily living	Standard	SRTI	16-17	N	No model - fewer than 3 periods with positive claims							
E00-E90	Daily living	Standard	SRTI	18-19	Y	No model - fewer than 3 periods with positive claims							
E00-E90	Daily living	Standard	SRTI	18-19	N	No model - fewer than 3 periods with positive claims							
E00-E90	Daily living	Standard	SRTI	20-24	Y	No model - fewer than 3 periods with positive claims							
E00-E90	Daily living	Standard	SRTI	20-24	N	No model - fewer than 3 periods with positive claims							
E00-E90	Daily living	Standard	SRTI	25-29	Y	No model - fewer than 3 periods with positive claims							

Continued on next page

Table 1 – continued from previous page

ICD-10 code	Component	Level	Rules	Ages	Female	Model type	A	s.e.	r	s.e.	k	s.e	RSE
E00-E90	Daily living	Standard	SRTI	25-29	N	No model - fewer than 3 periods with positive claims							
E00-E90	Daily living	Standard	SRTI	30-34	Y	LM	1.95×10^{-4}	—	0.032	0.02	-1.463	1.343	1.121
E00-E90	Daily living	Standard	SRTI	30-34	N	No model - fewer than 3 periods with positive claims							
E00-E90	Daily living	Standard	SRTI	35-39	Y	LG	1.06×10^{-15}	8.14×10^{-6}	14.053	1.74727E+14	-1119.899	1.39781E+16	1.15×10^{-5}
E00-E90	Daily living	Standard	SRTI	35-39	N	No model - fewer than 3 periods with positive claims							
E00-E90	Daily living	Standard	SRTI	40-44	Y	LM	5.36×10^{-5}	—	-0.029	9.33×10^{-5}	4.001	7.42×10^{-3}	3.90×10^{-4}
E00-E90	Daily living	Standard	SRTI	40-44	N	No model - fewer than 3 periods with positive claims							
E00-E90	Daily living	Standard	SRTI	45-49	Y	LM	2.53×10^{-5}	—	-0.346	0.03	31.762	2.27	0.043
E00-E90	Daily living	Standard	SRTI	45-49	N	No model - fewer than 3 periods with positive claims							
E00-E90	Daily living	Standard	SRTI	50-54	Y	No model - fewer than 3 periods with positive claims							
E00-E90	Daily living	Standard	SRTI	50-54	N	LM	3.68×10^{-5}	—	0.029	0.023	-0.121	1.002	0.711
E00-E90	Daily living	Standard	SRTI	55-59	Y	LG	1.36×10^{-5}	1.52×10^{-6}	0.327	0.263	-13.37	10.683	8.37×10^{-6}
E00-E90	Daily living	Standard	SRTI	55-59	N	LM	3.76×10^{-5}	—	0.032	0.029	-1.194	1.767	1.551
E00-E90	Daily living	Standard	SRTI	60-64	Y	LG	7.19×10^{-5}	1.94×10^{-5}	0.096	0.022	-6.679	1.033	8.56×10^{-6}
E00-E90	Daily living	Standard	SRTI	60-64	N	LG	7.60×10^{-5}	1.01×10^{-5}	0.131	0.032	-8.081	1.691	1.34×10^{-5}
E00-E90	Daily living	Standard	SRTI	65-69	Y	LM	3.01×10^{-5}	—	0.012	5.67×10^{-3}	-0.682	0.329	0.492
E00-E90	Daily living	Standard	SRTI	65-69	N	LM	1.71×10^{-5}	—	-0.025	0.029	3.789	1.557	1.251
E00-E90	Daily living	Standard	SRTI	70+	Y	No model - fewer than 3 periods with positive claims							
E00-E90	Daily living	Standard	SRTI	70+	N	No model - fewer than 3 periods with positive claims							
E00-E90	Daily living	Standard	non-SRTI	16-17	Y	LG	0.024	3.09×10^{-3}	0.05	4.37×10^{-3}	-3.363	0.105	1.05×10^{-3}
E00-E90	Daily living	Standard	non-SRTI	16-17	N	LG	0.013	4.14×10^{-4}	0.081	6.42×10^{-3}	-3.156	0.201	1.06×10^{-3}
E00-E90	Daily living	Standard	non-SRTI	18-19	Y	LG	5.89×10^{-3}	1.58×10^{-4}	0.085	5.26×10^{-3}	-3.764	0.186	3.58×10^{-4}
E00-E90	Daily living	Standard	non-SRTI	18-19	N	LG	5.77×10^{-3}	1.05×10^{-4}	0.117	6.98×10^{-3}	-4.957	0.269	3.54×10^{-4}
E00-E90	Daily living	Standard	non-SRTI	20-24	Y	LG	7.87×10^{-3}	1.07×10^{-4}	0.092	3.03×10^{-3}	-4.072	0.112	2.60×10^{-4}
E00-E90	Daily living	Standard	non-SRTI	20-24	N	LG	8.52×10^{-3}	1.45×10^{-4}	0.09	3.12×10^{-3}	-4.438	0.123	2.73×10^{-4}
E00-E90	Daily living	Standard	non-SRTI	25-29	Y	LG	6.57×10^{-3}	1.07×10^{-4}	0.08	2.20×10^{-3}	-4.151	0.083	1.55×10^{-4}
E00-E90	Daily living	Standard	non-SRTI	25-29	N	LG	5.83×10^{-3}	1.70×10^{-4}	0.071	2.88×10^{-3}	-3.845	0.101	1.87×10^{-4}
E00-E90	Daily living	Standard	non-SRTI	30-34	Y	LG	7.26×10^{-3}	3.17×10^{-4}	0.059	2.70×10^{-3}	-3.421	0.078	2.26×10^{-4}
E00-E90	Daily living	Standard	non-SRTI	30-34	N	LG	6.79×10^{-3}	1.53×10^{-4}	0.073	2.54×10^{-3}	-3.727	0.088	2.00×10^{-4}
E00-E90	Daily living	Standard	non-SRTI	35-39	Y	LG	4.00×10^{-3}	1.35×10^{-4}	0.065	3.18×10^{-3}	-3.304	0.099	1.59×10^{-4}
E00-E90	Daily living	Standard	non-SRTI	35-39	N	LG	4.16×10^{-3}	1.79×10^{-4}	0.063	3.23×10^{-3}	-3.473	0.1	1.58×10^{-4}
E00-E90	Daily living	Standard	non-SRTI	40-44	Y	LG	3.53×10^{-3}	7.34×10^{-5}	0.079	4.00×10^{-3}	-3.063	0.123	1.83×10^{-4}
E00-E90	Daily living	Standard	non-SRTI	40-44	N	LG	3.80×10^{-3}	8.00×10^{-5}	0.079	4.04×10^{-3}	-3.115	0.126	1.98×10^{-4}
E00-E90	Daily living	Standard	non-SRTI	45-49	Y	LG	3.23×10^{-3}	7.84×10^{-5}	0.072	3.72×10^{-3}	-2.937	0.112	1.62×10^{-4}
E00-E90	Daily living	Standard	non-SRTI	45-49	N	LG	3.45×10^{-3}	8.41×10^{-5}	0.077	4.56×10^{-3}	-2.955	0.137	2.08×10^{-4}
E00-E90	Daily living	Standard	non-SRTI	50-54	Y	LG	3.87×10^{-3}	1.04×10^{-4}	0.07	3.87×10^{-3}	-2.876	0.115	2.05×10^{-4}
E00-E90	Daily living	Standard	non-SRTI	50-54	N	LG	4.49×10^{-3}	1.19×10^{-4}	0.072	3.99×10^{-3}	-2.938	0.12	2.42×10^{-4}
E00-E90	Daily living	Standard	non-SRTI	55-59	Y	LG	3.40×10^{-3}	9.26×10^{-5}	0.068	3.59×10^{-3}	-2.912	0.107	1.66×10^{-4}
E00-E90	Daily living	Standard	non-SRTI	55-59	N	LG	3.96×10^{-3}	8.71×10^{-5}	0.078	4.28×10^{-3}	-2.96	0.129	2.23×10^{-4}
E00-E90	Daily living	Standard	non-SRTI	60-64	Y	LG	4.09×10^{-3}	1.42×10^{-4}	0.064	3.76×10^{-3}	-2.868	0.109	2.11×10^{-4}
E00-E90	Daily living	Standard	non-SRTI	60-64	N	LG	5.35×10^{-3}	1.14×10^{-4}	0.077	3.78×10^{-3}	-3.11	0.118	2.63×10^{-4}
E00-E90	Daily living	Standard	non-SRTI	65-69	Y	LG	2.31×10^{-3}	1.75×10^{-5}	0.104	1.86×10^{-3}	-5.184	0.079	3.83×10^{-5}
E00-E90	Daily living	Standard	non-SRTI	65-69	N	LG	3.45×10^{-3}	1.76×10^{-5}	0.113	1.54×10^{-3}	-5.508	0.066	4.47×10^{-5}
E00-E90	Daily living	Standard	non-SRTI	70+	Y	LG	2.52×10^{-4}	5.61×10^{-6}	0.234	8.38×10^{-3}	-16.92	0.558	4.50×10^{-6}
E00-E90	Daily living	Standard	non-SRTI	70+	N	LG	4.13×10^{-4}	6.96×10^{-6}	0.258	8.71×10^{-3}	-18.395	0.584	7.52×10^{-6}
E00-E90	Mobility	Standard	non-SRTI	16-17	Y	LG	5.49×10^{-3}	7.33×10^{-5}	0.36	0.045	-8.286	1.036	5.15×10^{-4}
E00-E90	Mobility	Standard	non-SRTI	16-17	N	LG	6.27×10^{-3}	8.15×10^{-5}	0.322	0.036	-7.992	0.896	5.54×10^{-4}
E00-E90	Mobility	Standard	non-SRTI	18-19	Y	LG	3.52×10^{-3}	6.34×10^{-5}	0.153	0.014	-4.545	0.407	3.40×10^{-4}
E00-E90	Mobility	Standard	non-SRTI	18-19	N	LG	4.42×10^{-3}	7.28×10^{-5}	0.174	0.015	-6.199	0.512	3.71×10^{-4}
E00-E90	Mobility	Standard	non-SRTI	20-24	Y	LG	4.91×10^{-3}	1.09×10^{-4}	0.094	6.25×10^{-3}	-3.471	0.201	3.53×10^{-4}

Continued on next page

Table 1 – continued from previous page

ICD-10 code	Component	Level	Rules	Ages	Female	Model type	A	s.e.	r	s.e.	k	s.e	RSE
E00-E90	Mobility	Standard	non-SRTI	20-24	N	LG	4.99×10^{-3}	1.45×10^{-4}	0.08	4.73×10^{-3}	-3.633	0.164	2.82×10^{-4}
E00-E90	Mobility	Standard	non-SRTI	25-29	Y	LG	2.63×10^{-3}	3.84×10^{-5}	0.119	6.64×10^{-3}	-4.203	0.219	1.60×10^{-4}
E00-E90	Mobility	Standard	non-SRTI	25-29	N	LG	3.02×10^{-3}	9.04×10^{-5}	0.074	4.22×10^{-3}	-3.337	0.138	1.61×10^{-4}
E00-E90	Mobility	Standard	non-SRTI	30-34	Y	LG	3.20×10^{-3}	6.91×10^{-5}	0.083	4.45×10^{-3}	-3.316	0.144	1.77×10^{-4}
E00-E90	Mobility	Standard	non-SRTI	30-34	N	LG	2.60×10^{-3}	6.56×10^{-5}	0.075	3.97×10^{-3}	-3.216	0.127	1.33×10^{-4}
E00-E90	Mobility	Standard	non-SRTI	35-39	Y	LG	1.93×10^{-3}	3.81×10^{-5}	0.085	4.83×10^{-3}	-2.969	0.143	1.18×10^{-4}
E00-E90	Mobility	Standard	non-SRTI	35-39	N	LG	1.37×10^{-3}	1.56×10^{-5}	0.125	6.50×10^{-3}	-3.501	0.175	7.86×10^{-5}
E00-E90	Mobility	Standard	non-SRTI	40-44	Y	LG	1.98×10^{-3}	2.55×10^{-5}	0.133	8.21×10^{-3}	-3.673	0.219	1.34×10^{-4}
E00-E90	Mobility	Standard	non-SRTI	40-44	N	LG	1.86×10^{-3}	1.67×10^{-5}	0.183	0.01	-4.351	0.24	1.04×10^{-4}
E00-E90	Mobility	Standard	non-SRTI	45-49	Y	LG	1.45×10^{-3}	1.27×10^{-5}	0.176	9.37×10^{-3}	-4.121	0.219	7.85×10^{-5}
E00-E90	Mobility	Standard	non-SRTI	45-49	N	LG	1.60×10^{-3}	1.37×10^{-5}	0.152	7.05×10^{-3}	-3.853	0.176	7.85×10^{-5}
E00-E90	Mobility	Standard	non-SRTI	50-54	Y	LG	1.81×10^{-3}	1.50×10^{-5}	0.171	8.21×10^{-3}	-4.408	0.209	8.88×10^{-5}
E00-E90	Mobility	Standard	non-SRTI	50-54	N	LG	2.08×10^{-3}	1.93×10^{-5}	0.18	0.01	-4.414	0.249	1.18×10^{-4}
E00-E90	Mobility	Standard	non-SRTI	55-59	Y	LG	1.40×10^{-3}	1.62×10^{-5}	0.156	0.01	-3.889	0.247	9.50×10^{-5}
E00-E90	Mobility	Standard	non-SRTI	55-59	N	LG	1.82×10^{-3}	1.94×10^{-5}	0.18	0.012	-4.136	0.273	1.22×10^{-4}
E00-E90	Mobility	Standard	non-SRTI	60-64	Y	LG	1.83×10^{-3}	2.54×10^{-5}	0.109	6.11×10^{-3}	-3.223	0.169	1.14×10^{-4}
E00-E90	Mobility	Standard	non-SRTI	60-64	N	LG	2.49×10^{-3}	2.92×10^{-5}	0.143	8.72×10^{-3}	-3.684	0.22	1.63×10^{-4}
E00-E90	Mobility	Standard	non-SRTI	65-69	Y	LG	1.22×10^{-3}	1.43×10^{-5}	0.099	2.72×10^{-3}	-4.709	0.109	3.24×10^{-5}
E00-E90	Mobility	Standard	non-SRTI	65-69	N	LG	1.75×10^{-3}	1.19×10^{-5}	0.111	2.27×10^{-3}	-4.866	0.089	3.62×10^{-5}
E00-E90	Mobility	Standard	non-SRTI	70+	Y	LG	1.23×10^{-4}	3.21×10^{-6}	0.243	0.011	-17.405	0.753	2.98×10^{-6}
E00-E90	Mobility	Standard	non-SRTI	70+	N	LG	2.33×10^{-4}	6.28×10^{-6}	0.228	0.011	-16.181	0.721	6.05×10^{-6}
F00-F99	Daily living	Enhanced	SRTI	16-17	Y	No model - fewer than 3 periods with positive claims							
F00-F99	Daily living	Enhanced	SRTI	16-17	N	No model - fewer than 3 periods with positive claims							
F00-F99	Daily living	Enhanced	SRTI	18-19	Y	No model - fewer than 3 periods with positive claims							
F00-F99	Daily living	Enhanced	SRTI	18-19	N	No model - fewer than 3 periods with positive claims							
F00-F99	Daily living	Enhanced	SRTI	20-24	Y	No model - fewer than 3 periods with positive claims							
F00-F99	Daily living	Enhanced	SRTI	20-24	N	No model - fewer than 3 periods with positive claims							
F00-F99	Daily living	Enhanced	SRTI	25-29	Y	No model - fewer than 3 periods with positive claims							
F00-F99	Daily living	Enhanced	SRTI	25-29	N	No model - fewer than 3 periods with positive claims							
F00-F99	Daily living	Enhanced	SRTI	30-34	Y	No model - fewer than 3 periods with positive claims							
F00-F99	Daily living	Enhanced	SRTI	30-34	N	No model - fewer than 3 periods with positive claims							
F00-F99	Daily living	Enhanced	SRTI	35-39	Y	No model - fewer than 3 periods with positive claims							
F00-F99	Daily living	Enhanced	SRTI	35-39	N	No model - fewer than 3 periods with positive claims							
F00-F99	Daily living	Enhanced	SRTI	40-44	Y	LG	6.75×10^{-5}	1.20×10^{-5}	0.121	0.044	-6.929	2.196	2.01×10^{-5}
F00-F99	Daily living	Enhanced	SRTI	40-44	N	LM	1.37×10^{-4}	—	-5.35×10^{-3}	0.034	0.359	2.5	0.931
F00-F99	Daily living	Enhanced	SRTI	45-49	Y	LM	1.55×10^{-4}	—	0.033	5.90×10^{-3}	-2.879	0.352	0.606
F00-F99	Daily living	Enhanced	SRTI	45-49	N	LG	1.38×10^{-4}	1.23×10^{-5}	0.138	0.026	-8.371	1.366	1.93×10^{-5}
F00-F99	Daily living	Enhanced	SRTI	50-54	Y	LG	1.13×10^{-4}	4.05×10^{-6}	0.455	0.135	-20.09	5.962	2.23×10^{-5}
F00-F99	Daily living	Enhanced	SRTI	50-54	N	LG	1.66×10^{-4}	4.94×10^{-6}	0.375	0.077	-18.031	3.694	2.45×10^{-5}
F00-F99	Daily living	Enhanced	SRTI	55-59	Y	LG	1.81×10^{-4}	8.74×10^{-6}	0.144	0.018	-8.208	0.931	1.87×10^{-5}
F00-F99	Daily living	Enhanced	SRTI	55-59	N	LG	2.25×10^{-4}	9.04×10^{-6}	0.129	0.015	-6.543	0.686	2.40×10^{-5}
F00-F99	Daily living	Enhanced	SRTI	60-64	Y	LG	3.42×10^{-4}	2.35×10^{-5}	0.094	0.012	-5.069	0.503	3.65×10^{-5}
F00-F99	Daily living	Enhanced	SRTI	60-64	N	LG	4.22×10^{-4}	1.56×10^{-5}	0.169	0.023	-8.586	1.104	5.15×10^{-5}
F00-F99	Daily living	Enhanced	SRTI	65-69	Y	LG	2.17×10^{-4}	7.53×10^{-6}	1.157	0.513	-57.893	25.67	4.07×10^{-5}
F00-F99	Daily living	Enhanced	SRTI	65-69	N	LG	3.70×10^{-4}	2.45×10^{-5}	0.195	0.035	-12.209	2.076	4.90×10^{-5}
F00-F99	Daily living	Enhanced	SRTI	70+	Y	LM	2.14×10^{-5}	—	0.083	0.066	-4.546	4.9	1.21
F00-F99	Daily living	Enhanced	SRTI	70+	N	LG	3.17×10^{-5}	7.30×10^{-7}	0.929	0.102	-69.259	7.548	1.46×10^{-6}
F00-F99	Daily living	Enhanced	non-SRTI	16-17	Y	LG	0.269	2.06×10^{-3}	0.179	7.84×10^{-3}	-5.302	0.228	0.012
F00-F99	Daily living	Enhanced	non-SRTI	16-17	N	LG	0.806	7.3×10^{-3}	0.199	0.011	-5.725	0.316	0.043
F00-F99	Daily living	Enhanced	non-SRTI	18-19	Y	LG	0.157	1.07×10^{-3}	0.101	2.11×10^{-3}	-4.039	0.074	3.44×10^{-3}

Continued on next page

Table 1 – continued from previous page

ICD-10 code	Component	Level	Rules	Ages	Female	Model type	A	s.e.	r	s.e.	k	s.e	RSE
F00-F99	Daily living	Enhanced	non-SRTI	18-19	N	LG	0.444	1.92×10^{-3}	0.112	1.56×10^{-3}	-4.601	0.058	6.46×10^{-3}
F00-F99	Daily living	Enhanced	non-SRTI	20-24	Y	LG	0.142	2.17×10^{-3}	0.079	2.63×10^{-3}	-3.399	0.087	4.65×10^{-3}
F00-F99	Daily living	Enhanced	non-SRTI	20-24	N	LG	0.361	4.66×10^{-3}	0.079	1.91×10^{-3}	-3.834	0.069	7.93×10^{-3}
F00-F99	Daily living	Enhanced	non-SRTI	25-29	Y	LG	0.09	1.37×10^{-3}	0.082	3.09×10^{-3}	-3.27	0.099	3.49×10^{-3}
F00-F99	Daily living	Enhanced	non-SRTI	25-29	N	LG	0.193	3.24×10^{-3}	0.076	2.31×10^{-3}	-3.594	0.079	5.38×10^{-3}
F00-F99	Daily living	Enhanced	non-SRTI	30-34	Y	LG	0.099	1.42×10^{-3}	0.086	3.33×10^{-3}	-3.271	0.106	4.04×10^{-3}
F00-F99	Daily living	Enhanced	non-SRTI	30-34	N	LG	0.179	2.53×10^{-3}	0.08	2.52×10^{-3}	-3.481	0.085	5.50×10^{-3}
F00-F99	Daily living	Enhanced	non-SRTI	35-39	Y	LG	0.074	1.02×10^{-3}	0.089	3.59×10^{-3}	-3.256	0.112	3.19×10^{-3}
F00-F99	Daily living	Enhanced	non-SRTI	35-39	N	LG	0.125	1.72×10^{-3}	0.082	2.62×10^{-3}	-3.438	0.087	4.01×10^{-3}
F00-F99	Daily living	Enhanced	non-SRTI	40-44	Y	LG	0.088	9.80×10^{-4}	0.1	3.80×10^{-3}	-3.384	0.116	3.69×10^{-3}
F00-F99	Daily living	Enhanced	non-SRTI	40-44	N	LG	0.137	1.54×10^{-3}	0.091	2.95×10^{-3}	-3.489	0.096	4.70×10^{-3}
F00-F99	Daily living	Enhanced	non-SRTI	45-49	Y	LG	0.079	8.83×10^{-4}	0.095	3.33×10^{-3}	-3.441	0.105	2.99×10^{-3}
F00-F99	Daily living	Enhanced	non-SRTI	45-49	N	LG	0.119	1.29×10^{-3}	0.089	2.47×10^{-3}	-3.668	0.085	3.42×10^{-3}
F00-F99	Daily living	Enhanced	non-SRTI	50-54	Y	LG	0.095	1.06×10^{-3}	0.09	2.80×10^{-3}	-3.53	0.093	3.10×10^{-3}
F00-F99	Daily living	Enhanced	non-SRTI	50-54	N	LG	0.135	1.56×10^{-3}	0.086	2.37×10^{-3}	-3.671	0.082	3.73×10^{-3}
F00-F99	Daily living	Enhanced	non-SRTI	55-59	Y	LG	0.087	1.03×10^{-3}	0.086	2.51×10^{-3}	-3.636	0.086	2.57×10^{-3}
F00-F99	Daily living	Enhanced	non-SRTI	55-59	N	LG	0.119	1.43×10^{-3}	0.085	2.30×10^{-3}	-3.753	0.081	3.18×10^{-3}
F00-F99	Daily living	Enhanced	non-SRTI	60-64	Y	LG	0.124	1.06×10^{-3}	0.092	1.94×10^{-3}	-4.12	0.072	2.58×10^{-3}
F00-F99	Daily living	Enhanced	non-SRTI	60-64	N	LG	0.172	1.52×10^{-3}	0.091	1.91×10^{-3}	-4.048	0.07	3.59×10^{-3}
F00-F99	Daily living	Enhanced	non-SRTI	65-69	Y	LG	0.096	5.11×10^{-4}	0.144	2.61×10^{-3}	-6.943	0.118	1.66×10^{-3}
F00-F99	Daily living	Enhanced	non-SRTI	65-69	N	LG	0.14	6.27×10^{-4}	0.148	2.32×10^{-3}	-7.092	0.105	2.09×10^{-3}
F00-F99	Daily living	Enhanced	non-SRTI	70+	Y	LG	8.15×10^{-3}	1.49×10^{-4}	0.254	9.37×10^{-3}	-18.023	0.625	1.65×10^{-4}
F00-F99	Daily living	Enhanced	non-SRTI	70+	N	LG	0.014	2.78×10^{-4}	0.246	9.53×10^{-3}	-17.479	0.634	2.84×10^{-4}
F00-F99	Mobility	Enhanced	non-SRTI	16-17	Y	LG	0.054	2.99×10^{-4}	0.182	5.89×10^{-3}	-5.326	0.17	1.72×10^{-3}
F00-F99	Mobility	Enhanced	non-SRTI	16-17	N	LG	0.155	1.53×10^{-3}	0.209	0.013	-6.281	0.386	9.04×10^{-3}
F00-F99	Mobility	Enhanced	non-SRTI	18-19	Y	LG	0.039	3.07×10^{-4}	0.114	3.39×10^{-3}	-3.957	0.109	1.26×10^{-3}
F00-F99	Mobility	Enhanced	non-SRTI	18-19	N	LG	0.089	5.10×10^{-4}	0.111	2.14×10^{-3}	-4.298	0.075	1.84×10^{-3}
F00-F99	Mobility	Enhanced	non-SRTI	20-24	Y	LG	0.055	5.37×10^{-4}	0.101	3.08×10^{-3}	-3.818	0.104	1.81×10^{-3}
F00-F99	Mobility	Enhanced	non-SRTI	20-24	N	LG	0.089	9.55×10^{-4}	0.093	2.75×10^{-3}	-3.796	0.095	2.74×10^{-3}
F00-F99	Mobility	Enhanced	non-SRTI	25-29	Y	LG	0.054	6.58×10^{-4}	0.091	2.92×10^{-3}	-3.761	0.101	1.80×10^{-3}
F00-F99	Mobility	Enhanced	non-SRTI	25-29	N	LG	0.077	9.74×10^{-4}	0.087	2.66×10^{-3}	-3.794	0.094	2.34×10^{-3}
F00-F99	Mobility	Enhanced	non-SRTI	30-34	Y	LG	0.074	9.68×10^{-4}	0.086	2.62×10^{-3}	-3.733	0.092	2.25×10^{-3}
F00-F99	Mobility	Enhanced	non-SRTI	30-34	N	LG	0.098	1.25×10^{-3}	0.086	2.45×10^{-3}	-3.846	0.088	2.74×10^{-3}
F00-F99	Mobility	Enhanced	non-SRTI	35-39	Y	LG	0.058	6.43×10^{-4}	0.089	2.41×10^{-3}	-3.836	0.085	1.59×10^{-3}
F00-F99	Mobility	Enhanced	non-SRTI	35-39	N	LG	0.078	9.07×10^{-4}	0.088	2.38×10^{-3}	-3.949	0.086	2.07×10^{-3}
F00-F99	Mobility	Enhanced	non-SRTI	40-44	Y	LG	0.071	7.13×10^{-4}	0.093	2.50×10^{-3}	-3.88	0.088	1.97×10^{-3}
F00-F99	Mobility	Enhanced	non-SRTI	40-44	N	LG	0.096	9.15×10^{-4}	0.091	2.08×10^{-3}	-4.049	0.076	2.19×10^{-3}
F00-F99	Mobility	Enhanced	non-SRTI	45-49	Y	LG	0.062	6.89×10^{-4}	0.089	2.46×10^{-3}	-3.843	0.087	1.72×10^{-3}
F00-F99	Mobility	Enhanced	non-SRTI	45-49	N	LG	0.083	9.73×10^{-4}	0.084	2.04×10^{-3}	-3.966	0.075	1.91×10^{-3}
F00-F99	Mobility	Enhanced	non-SRTI	50-54	Y	LG	0.069	7.30×10^{-4}	0.089	2.25×10^{-3}	-3.874	0.08	1.75×10^{-3}
F00-F99	Mobility	Enhanced	non-SRTI	50-54	N	LG	0.087	1.01×10^{-3}	0.085	2.03×10^{-3}	-3.997	0.075	1.97×10^{-3}
F00-F99	Mobility	Enhanced	non-SRTI	55-59	Y	LG	0.062	9.03×10^{-4}	0.079	2.16×10^{-3}	-3.779	0.077	1.55×10^{-3}
F00-F99	Mobility	Enhanced	non-SRTI	55-59	N	LG	0.074	1.21×10^{-3}	0.078	2.15×10^{-3}	-3.935	0.078	1.78×10^{-3}
F00-F99	Mobility	Enhanced	non-SRTI	60-64	Y	LG	0.078	1.05×10^{-3}	0.08	1.82×10^{-3}	-4.085	0.068	1.56×10^{-3}
F00-F99	Mobility	Enhanced	non-SRTI	60-64	N	LG	0.092	1.27×10^{-3}	0.081	1.97×10^{-3}	-4.117	0.074	1.97×10^{-3}
F00-F99	Mobility	Enhanced	non-SRTI	65-69	Y	LG	0.045	9.31×10^{-4}	0.095	3.19×10^{-3}	-5.429	0.141	1.18×10^{-3}
F00-F99	Mobility	Enhanced	non-SRTI	65-69	N	LG	0.055	7.56×10^{-4}	0.101	2.55×10^{-3}	-5.673	0.116	1.14×10^{-3}
F00-F99	Mobility	Enhanced	non-SRTI	70+	Y	LG	2.18×10^{-3}	4.88×10^{-5}	0.248	0.011	-17.551	0.747	5.46×10^{-5}
F00-F99	Mobility	Enhanced	non-SRTI	70+	N	LG	3.40×10^{-3}	7.96×10^{-5}	0.232	8.85×10^{-3}	-16.751	0.588	6.53×10^{-5}

Continued on next page

Table 1 – continued from previous page

ICD-10 code	Component	Level	Rules	Ages	Female	Model type	A	s.e.	r	s.e.	k	s.e	RSE
F00-F99	Daily living	Standard	SRTI	16-17	Y	No model - fewer than 3 periods with positive claims							
F00-F99	Daily living	Standard	SRTI	16-17	N	No model - fewer than 3 periods with positive claims							
F00-F99	Daily living	Standard	SRTI	18-19	Y	No model - fewer than 3 periods with positive claims							
F00-F99	Daily living	Standard	SRTI	18-19	N	No model - fewer than 3 periods with positive claims							
F00-F99	Daily living	Standard	SRTI	20-24	Y	No model - fewer than 3 periods with positive claims							
F00-F99	Daily living	Standard	SRTI	20-24	N	No model - fewer than 3 periods with positive claims							
F00-F99	Daily living	Standard	SRTI	25-29	Y	No model - fewer than 3 periods with positive claims							
F00-F99	Daily living	Standard	SRTI	25-29	N	No model - fewer than 3 periods with positive claims							
F00-F99	Daily living	Standard	SRTI	30-34	Y	No model - fewer than 3 periods with positive claims							
F00-F99	Daily living	Standard	SRTI	30-34	N	No model - fewer than 3 periods with positive claims							
F00-F99	Daily living	Standard	SRTI	35-39	Y	No model - fewer than 3 periods with positive claims							
F00-F99	Daily living	Standard	SRTI	35-39	N	No model - fewer than 3 periods with positive claims							
F00-F99	Daily living	Standard	SRTI	40-44	Y	LG	6.58×10^{-5}	1.35×10^{-5}	0.109	0.038	-6.506	1.855	1.74×10^{-5}
F00-F99	Daily living	Standard	SRTI	40-44	N	LM	1.37×10^{-4}	—	-5.35×10^{-3}	0.034	0.359	2.5	0.931
F00-F99	Daily living	Standard	SRTI	45-49	Y	LM	1.55×10^{-4}	—	0.033	5.90×10^{-3}	-2.879	0.352	0.606
F00-F99	Daily living	Standard	SRTI	45-49	N	LG	1.38×10^{-4}	1.23×10^{-5}	0.138	0.026	-8.371	1.366	1.93×10^{-5}
F00-F99	Daily living	Standard	SRTI	50-54	Y	LG	1.13×10^{-4}	4.05×10^{-6}	0.455	0.135	-20.09	5.962	2.23×10^{-5}
F00-F99	Daily living	Standard	SRTI	50-54	N	LG	1.66×10^{-4}	4.96×10^{-6}	0.368	0.076	-17.715	3.615	2.45×10^{-5}
F00-F99	Daily living	Standard	SRTI	55-59	Y	LG	1.81×10^{-4}	8.74×10^{-6}	0.144	0.018	-8.208	0.931	1.87×10^{-5}
F00-F99	Daily living	Standard	SRTI	55-59	N	LG	2.17×10^{-4}	8.66×10^{-6}	0.136	0.017	-6.776	0.767	2.49×10^{-5}
F00-F99	Daily living	Standard	SRTI	60-64	Y	LG	3.30×10^{-4}	2.27×10^{-5}	0.091	0.011	-4.882	0.478	3.53×10^{-5}
F00-F99	Daily living	Standard	SRTI	60-64	N	LG	4.22×10^{-4}	1.56×10^{-5}	0.169	0.023	-8.586	1.104	5.15×10^{-5}
F00-F99	Daily living	Standard	SRTI	65-69	Y	LG	3.33×10^{-4}	3.35×10^{-4}	0.068	0.027	-5.477	0.743	4.25×10^{-5}
F00-F99	Daily living	Standard	SRTI	65-69	N	LG	4.10×10^{-4}	1.55×10^{-4}	0.131	0.036	-9.914	1.983	4.28×10^{-5}
F00-F99	Daily living	Standard	SRTI	70+	Y	LM	1.68×10^{-5}	—	0.087	0.218	-5.022	17.1	1.411
F00-F99	Daily living	Standard	SRTI	70+	N	LM	2.31×10^{-5}	—	0.576	0.215	-42.721	17.101	0.891
F00-F99	Daily living	Standard	non-SRTI	16-17	Y	LG	0.188	1.36×10^{-3}	0.145	4.84×10^{-3}	-4.879	0.157	6.63×10^{-3}
F00-F99	Daily living	Standard	non-SRTI	16-17	N	LG	0.521	3.21×10^{-3}	0.151	4.48×10^{-3}	-4.942	0.142	0.016
F00-F99	Daily living	Standard	non-SRTI	18-19	Y	LG	0.104	7.09×10^{-4}	0.095	1.42×10^{-3}	-4.645	0.057	1.46×10^{-3}
F00-F99	Daily living	Standard	non-SRTI	18-19	N	LG	0.264	1.26×10^{-3}	0.105	1.26×10^{-3}	-4.997	0.052	3.07×10^{-3}
F00-F99	Daily living	Standard	non-SRTI	20-24	Y	LG	0.095	2.06×10^{-3}	0.075	1.94×10^{-3}	-4.47	0.073	1.74×10^{-3}
F00-F99	Daily living	Standard	non-SRTI	20-24	N	LG	0.21	3.41×10^{-3}	0.084	1.87×10^{-3}	-4.863	0.077	3.53×10^{-3}
F00-F99	Daily living	Standard	non-SRTI	25-29	Y	LG	0.062	1.51×10^{-3}	0.072	1.77×10^{-3}	-4.517	0.065	9.83×10^{-4}
F00-F99	Daily living	Standard	non-SRTI	25-29	N	LG	0.116	2.67×10^{-3}	0.081	2.06×10^{-3}	-5.038	0.084	1.96×10^{-3}
F00-F99	Daily living	Standard	non-SRTI	30-34	Y	LG	0.067	1.89×10^{-3}	0.068	1.80×10^{-3}	-4.267	0.061	1.12×10^{-3}
F00-F99	Daily living	Standard	non-SRTI	30-34	N	LG	0.107	2.38×10^{-3}	0.076	1.78×10^{-3}	-4.743	0.068	1.63×10^{-3}
F00-F99	Daily living	Standard	non-SRTI	35-39	Y	LG	0.05	1.52×10^{-3}	0.064	1.80×10^{-3}	-3.963	0.057	8.95×10^{-4}
F00-F99	Daily living	Standard	non-SRTI	35-39	N	LG	0.072	1.60×10^{-3}	0.072	1.59×10^{-3}	-4.476	0.058	1.03×10^{-3}
F00-F99	Daily living	Standard	non-SRTI	40-44	Y	LG	0.052	1.10×10^{-3}	0.07	2.25×10^{-3}	-3.585	0.075	1.38×10^{-3}
F00-F99	Daily living	Standard	non-SRTI	40-44	N	LG	0.077	1.94×10^{-3}	0.069	2.07×10^{-3}	-3.933	0.071	1.70×10^{-3}
F00-F99	Daily living	Standard	non-SRTI	45-49	Y	LG	0.048	7.98×10^{-4}	0.075	2.15×10^{-3}	-3.715	0.075	1.22×10^{-3}
F00-F99	Daily living	Standard	non-SRTI	45-49	N	LG	0.066	9.58×10^{-4}	0.077	1.69×10^{-3}	-4.132	0.063	1.18×10^{-3}
F00-F99	Daily living	Standard	non-SRTI	50-54	Y	LG	0.062	1.00×10^{-3}	0.076	2.13×10^{-3}	-3.726	0.075	1.53×10^{-3}
F00-F99	Daily living	Standard	non-SRTI	50-54	N	LG	0.08	1.28×10^{-3}	0.077	1.90×10^{-3}	-4.06	0.07	1.64×10^{-3}
F00-F99	Daily living	Standard	non-SRTI	55-59	Y	LG	0.062	9.27×10^{-4}	0.078	2.02×10^{-3}	-3.843	0.073	1.44×10^{-3}
F00-F99	Daily living	Standard	non-SRTI	55-59	N	LG	0.076	1.18×10^{-3}	0.077	1.95×10^{-3}	-3.984	0.071	1.64×10^{-3}
F00-F99	Daily living	Standard	non-SRTI	60-64	Y	LG	0.093	9.58×10^{-4}	0.086	1.78×10^{-3}	-4.146	0.067	1.80×10^{-3}
F00-F99	Daily living	Standard	non-SRTI	60-64	N	LG	0.12	1.26×10^{-3}	0.086	1.77×10^{-3}	-4.223	0.068	2.26×10^{-3}
F00-F99	Daily living	Standard	non-SRTI	65-69	Y	LG	0.077	4.88×10^{-4}	0.137	2.83×10^{-3}	-6.696	0.127	1.49×10^{-3}
F00-F99	Daily living	Standard	non-SRTI	65-69	N	LG	0.102	5.23×10^{-4}	0.149	2.69×10^{-3}	-7.163	0.122	1.75×10^{-3}

Continued on next page

Table 1 – continued from previous page

ICD-10 code	Component	Level	Rules	Ages	Female	Model type	A	s.e.	r	s.e.	k	s.e	RSE
F00-F99	Daily living	Standard	non-SRTI	70+	Y	LG	6.30×10^{-3}	1.24×10^{-4}	0.251	9.95×10^{-3}	-17.776	0.662	1.38×10^{-4}
F00-F99	Daily living	Standard	non-SRTI	70+	N	LG	0.01	2.10×10^{-4}	0.247	0.01	-17.555	0.67	2.24×10^{-4}
F00-F99	Mobility	Standard	non-SRTI	16-17	Y	LG	0.064	7.47×10^{-4}	0.251	0.022	-6.221	0.539	4.91×10^{-3}
F00-F99	Mobility	Standard	non-SRTI	16-17	N	LG	0.238	4.7×10^{-3}	0.312	0.053	-7.521	1.267	0.032
F00-F99	Mobility	Standard	non-SRTI	18-19	Y	LG	0.043	2.25×10^{-4}	0.135	3.13×10^{-3}	-4.404	0.098	1.08×10^{-3}
F00-F99	Mobility	Standard	non-SRTI	18-19	N	LG	0.152	1.39×10^{-3}	0.142	5.88×10^{-3}	-4.9	0.194	6.62×10^{-3}
F00-F99	Mobility	Standard	non-SRTI	20-24	Y	LG	0.058	1.32×10^{-3}	0.07	2.57×10^{-3}	-3.451	0.085	1.85×10^{-3}
F00-F99	Mobility	Standard	non-SRTI	20-24	N	LG	0.153	2.67×10^{-3}	0.075	2.25×10^{-3}	-3.669	0.078	4.07×10^{-3}
F00-F99	Mobility	Standard	non-SRTI	25-29	Y	LG	0.046	1.40×10^{-3}	0.063	2.36×10^{-3}	-3.403	0.072	1.28×10^{-3}
F00-F99	Mobility	Standard	non-SRTI	25-29	N	LG	0.093	3.07×10^{-3}	0.063	2.24×10^{-3}	-3.683	0.07	2.28×10^{-3}
F00-F99	Mobility	Standard	non-SRTI	30-34	Y	LG	0.053	1.56×10^{-3}	0.065	2.52×10^{-3}	-3.415	0.079	1.61×10^{-3}
F00-F99	Mobility	Standard	non-SRTI	30-34	N	LG	0.089	2.76×10^{-3}	0.063	2.25×10^{-3}	-3.579	0.071	2.28×10^{-3}
F00-F99	Mobility	Standard	non-SRTI	35-39	Y	LG	0.038	9.42×10^{-4}	0.068	2.71×10^{-3}	-3.277	0.086	1.30×10^{-3}
F00-F99	Mobility	Standard	non-SRTI	35-39	N	LG	0.063	1.82×10^{-3}	0.064	2.21×10^{-3}	-3.519	0.069	1.62×10^{-3}
F00-F99	Mobility	Standard	non-SRTI	40-44	Y	LG	0.043	6.83×10^{-4}	0.08	2.93×10^{-3}	-3.345	0.096	1.57×10^{-3}
F00-F99	Mobility	Standard	non-SRTI	40-44	N	LG	0.065	1.35×10^{-3}	0.072	2.59×10^{-3}	-3.441	0.086	2.10×10^{-3}
F00-F99	Mobility	Standard	non-SRTI	45-49	Y	LG	0.04	6.44×10^{-4}	0.08	2.93×10^{-3}	-3.369	0.097	1.45×10^{-3}
F00-F99	Mobility	Standard	non-SRTI	45-49	N	LG	0.055	1.11×10^{-3}	0.072	2.46×10^{-3}	-3.471	0.082	1.67×10^{-3}
F00-F99	Mobility	Standard	non-SRTI	50-54	Y	LG	0.046	5.77×10^{-4}	0.083	2.42×10^{-3}	-3.508	0.082	1.35×10^{-3}
F00-F99	Mobility	Standard	non-SRTI	50-54	N	LG	0.059	9.15×10^{-4}	0.077	2.32×10^{-3}	-3.591	0.08	1.65×10^{-3}
F00-F99	Mobility	Standard	non-SRTI	55-59	Y	LG	0.041	6.17×10^{-4}	0.079	2.53×10^{-3}	-3.515	0.086	1.25×10^{-3}
F00-F99	Mobility	Standard	non-SRTI	55-59	N	LG	0.051	8.73×10^{-4}	0.077	2.55×10^{-3}	-3.58	0.088	1.56×10^{-3}
F00-F99	Mobility	Standard	non-SRTI	60-64	Y	LG	0.054	6.86×10^{-4}	0.083	2.13×10^{-3}	-3.874	0.077	1.32×10^{-3}
F00-F99	Mobility	Standard	non-SRTI	60-64	N	LG	0.066	7.50×10^{-4}	0.084	2.11×10^{-3}	-3.807	0.075	1.61×10^{-3}
F00-F99	Mobility	Standard	non-SRTI	65-69	Y	LG	0.032	3.64×10^{-4}	0.113	3.12×10^{-3}	-5.853	0.14	7.98×10^{-4}
F00-F99	Mobility	Standard	non-SRTI	65-69	N	LG	0.044	3.65×10^{-4}	0.119	2.63×10^{-3}	-6.016	0.118	8.98×10^{-4}
F00-F99	Mobility	Standard	non-SRTI	70+	Y	LG	2.17×10^{-3}	4.48×10^{-5}	0.247	9.99×10^{-3}	-17.542	0.665	4.78×10^{-5}
F00-F99	Mobility	Standard	non-SRTI	70+	N	LG	3.44×10^{-3}	7.34×10^{-5}	0.242	9.55×10^{-3}	-17.251	0.635	7.23×10^{-5}
G00-G99	Daily living	Enhanced	SRTI	16-17	Y	No model - fewer than 3 periods with positive claims							
G00-G99	Daily living	Enhanced	SRTI	16-17	N	No model - fewer than 3 periods with positive claims							
G00-G99	Daily living	Enhanced	SRTI	18-19	Y	No model - fewer than 3 periods with positive claims							
G00-G99	Daily living	Enhanced	SRTI	18-19	N	LM	3.96×10^{-4}	—	0.075	0.039	-4.041	2.729	0.254
G00-G99	Daily living	Enhanced	SRTI	20-24	Y	No model - fewer than 3 periods with positive claims							
G00-G99	Daily living	Enhanced	SRTI	20-24	N	LM	2.89×10^{-4}	—	0.389	0.283	-28.897	22.295	1.778
G00-G99	Daily living	Enhanced	SRTI	25-29	Y	No model - fewer than 3 periods with positive claims							
G00-G99	Daily living	Enhanced	SRTI	25-29	N	LM	1.14×10^{-4}	—	0.059	0.028	-1.854	1.815	1.309
G00-G99	Daily living	Enhanced	SRTI	30-34	Y	LM	2.09×10^{-4}	—	9.08×10^{-3}	6.76×10^{-3}	-0.965	0.42	0.548
G00-G99	Daily living	Enhanced	SRTI	30-34	N	LG	2.89×10^{-4}	1.86×10^{-5}	0.168	0.042	-8.053	1.949	6.80×10^{-5}
G00-G99	Daily living	Enhanced	SRTI	35-39	Y	LM	3.2×10^{-4}	—	0.029	0.013	-2.205	0.852	0.87
G00-G99	Daily living	Enhanced	SRTI	35-39	N	LM	4.90×10^{-4}	—	0.032	7.35×10^{-3}	-3.207	0.481	0.571
G00-G99	Daily living	Enhanced	SRTI	40-44	Y	LG	3.65×10^{-4}	1.26×10^{-5}	0.156	0.018	-8.14	0.864	3.71×10^{-5}
G00-G99	Daily living	Enhanced	SRTI	40-44	N	LG	6.62×10^{-4}	1.98×10^{-5}	0.102	0.01	-3.598	0.328	7.35×10^{-5}
G00-G99	Daily living	Enhanced	SRTI	45-49	Y	LG	4.02×10^{-4}	1.66×10^{-5}	0.076	6.56×10^{-3}	-3.32	0.215	3.34×10^{-5}
G00-G99	Daily living	Enhanced	SRTI	45-49	N	LG	6.49×10^{-4}	2.64×10^{-5}	0.067	6.35×10^{-3}	-2.436	0.169	5.93×10^{-5}
G00-G99	Daily living	Enhanced	SRTI	50-54	Y	LG	4.78×10^{-4}	1.24×10^{-5}	0.098	8.88×10^{-3}	-3.127	0.256	4.81×10^{-5}
G00-G99	Daily living	Enhanced	SRTI	50-54	N	LG	1.21×10^{-3}	1.62×10^{-5}	0.099	4.73×10^{-3}	-3.142	0.136	6.42×10^{-5}
G00-G99	Daily living	Enhanced	SRTI	55-59	Y	LG	8.68×10^{-4}	2.91×10^{-5}	0.065	4.40×10^{-3}	-2.566	0.12	5.48×10^{-5}
G00-G99	Daily living	Enhanced	SRTI	55-59	N	LG	1.97×10^{-3}	6.93×10^{-5}	0.056	3.33×10^{-3}	-2.341	0.082	9.71×10^{-5}
G00-G99	Daily living	Enhanced	SRTI	60-64	Y	LG	1.40×10^{-3}	2.76×10^{-5}	0.08	4.14×10^{-3}	-2.913	0.123	7.59×10^{-5}

Continued on next page

Table 1 – continued from previous page

ICD-10 code	Component	Level	Rules	Ages	Female	Model type	A	s.e.	r	s.e.	k	s.e	RSE
G00-G99	Daily living	Enhanced	SRTI	60-64	N	LG	3.32×10^{-3}	1.14×10^{-4}	0.059	3.62×10^{-3}	-2.374	0.091	1.77×10^{-4}
G00-G99	Daily living	Enhanced	SRTI	65-69	Y	LG	8.63×10^{-4}	4.79×10^{-5}	0.088	6.86×10^{-3}	-5.142	0.292	5.04×10^{-5}
G00-G99	Daily living	Enhanced	SRTI	65-69	N	LG	1.51×10^{-3}	3.76×10^{-5}	0.094	5.43×10^{-3}	-4.397	0.21	8.42×10^{-5}
G00-G99	Daily living	Enhanced	SRTI	70+	Y	LG	4.39×10^{-5}	2.57×10^{-6}	0.342	0.058	-24.047	3.98	4.28×10^{-6}
G00-G99	Daily living	Enhanced	SRTI	70+	N	LG	1.70×10^{-4}	4.45×10^{-5}	0.159	0.022	-12.695	1.261	6.16×10^{-6}
G00-G99	Daily living	Enhanced	non-SRTI	16-17	Y	LG	0.055	2.34×10^{-4}	0.168	3.88×10^{-3}	-5.038	0.114	1.30×10^{-3}
G00-G99	Daily living	Enhanced	non-SRTI	16-17	N	LG	0.093	3.78×10^{-4}	0.17	3.76×10^{-3}	-5.239	0.114	2.08×10^{-3}
G00-G99	Daily living	Enhanced	non-SRTI	18-19	Y	LG	0.049	2.55×10^{-4}	0.105	1.61×10^{-3}	-4.527	0.061	7.64×10^{-4}
G00-G99	Daily living	Enhanced	non-SRTI	18-19	N	LG	0.078	2.73×10^{-4}	0.117	1.22×10^{-3}	-5.34	0.05	8.12×10^{-4}
G00-G99	Daily living	Enhanced	non-SRTI	20-24	Y	LG	0.061	8.55×10^{-4}	0.077	1.80×10^{-3}	-3.937	0.065	1.23×10^{-3}
G00-G99	Daily living	Enhanced	non-SRTI	20-24	N	LG	0.087	1.30×10^{-3}	0.084	2.03×10^{-3}	-4.595	0.081	1.72×10^{-3}
G00-G99	Daily living	Enhanced	non-SRTI	25-29	Y	LG	0.043	6.16×10^{-4}	0.077	1.95×10^{-3}	-3.816	0.07	9.71×10^{-4}
G00-G99	Daily living	Enhanced	non-SRTI	25-29	N	LG	0.058	6.53×10^{-4}	0.086	1.64×10^{-3}	-4.597	0.066	9.34×10^{-4}
G00-G99	Daily living	Enhanced	non-SRTI	30-34	Y	LG	0.056	8.70×10^{-4}	0.075	2.01×10^{-3}	-3.675	0.07	1.33×10^{-3}
G00-G99	Daily living	Enhanced	non-SRTI	30-34	N	LG	0.067	9.16×10^{-4}	0.082	1.82×10^{-3}	-4.32	0.07	1.27×10^{-3}
G00-G99	Daily living	Enhanced	non-SRTI	35-39	Y	LG	0.046	6.68×10^{-4}	0.077	2.19×10^{-3}	-3.575	0.075	1.21×10^{-3}
G00-G99	Daily living	Enhanced	non-SRTI	35-39	N	LG	0.056	8.76×10^{-4}	0.074	1.69×10^{-3}	-4.002	0.061	1.03×10^{-3}
G00-G99	Daily living	Enhanced	non-SRTI	40-44	Y	LG	0.061	7.54×10^{-4}	0.083	2.24×10^{-3}	-3.677	0.078	1.62×10^{-3}
G00-G99	Daily living	Enhanced	non-SRTI	40-44	N	LG	0.064	7.68×10^{-4}	0.082	2.05×10^{-3}	-3.785	0.073	1.51×10^{-3}
G00-G99	Daily living	Enhanced	non-SRTI	45-49	Y	LG	0.059	6.52×10^{-4}	0.082	1.90×10^{-3}	-3.782	0.068	1.31×10^{-3}
G00-G99	Daily living	Enhanced	non-SRTI	45-49	N	LG	0.064	8.18×10^{-4}	0.081	2.08×10^{-3}	-3.768	0.074	1.56×10^{-3}
G00-G99	Daily living	Enhanced	non-SRTI	50-54	Y	LG	0.085	9.64×10^{-4}	0.084	1.91×10^{-3}	-4.014	0.071	1.81×10^{-3}
G00-G99	Daily living	Enhanced	non-SRTI	50-54	N	LG	0.098	1.18×10^{-3}	0.079	1.82×10^{-3}	-3.782	0.065	2.08×10^{-3}
G00-G99	Daily living	Enhanced	non-SRTI	55-59	Y	LG	0.094	1.17×10^{-3}	0.082	1.85×10^{-3}	-4.092	0.069	1.89×10^{-3}
G00-G99	Daily living	Enhanced	non-SRTI	55-59	N	LG	0.112	1.49×10^{-3}	0.079	2.08×10^{-3}	-3.68	0.073	2.76×10^{-3}
G00-G99	Daily living	Enhanced	non-SRTI	60-64	Y	LG	0.128	1.31×10^{-3}	0.088	1.86×10^{-3}	-4.309	0.072	2.49×10^{-3}
G00-G99	Daily living	Enhanced	non-SRTI	60-64	N	LG	0.181	2.13×10^{-3}	0.083	2.08×10^{-3}	-3.757	0.074	4.39×10^{-3}
G00-G99	Daily living	Enhanced	non-SRTI	65-69	Y	LG	0.102	5.65×10^{-4}	0.147	2.77×10^{-3}	-7.219	0.127	1.80×10^{-3}
G00-G99	Daily living	Enhanced	non-SRTI	65-69	N	LG	0.154	5.55×10^{-4}	0.144	1.79×10^{-3}	-6.919	0.08	1.82×10^{-3}
G00-G99	Daily living	Enhanced	non-SRTI	70+	Y	LG	0.013	2.81×10^{-4}	0.241	9.14×10^{-3}	-17.257	0.609	2.61×10^{-4}
G00-G99	Daily living	Enhanced	non-SRTI	70+	N	LG	0.021	4.40×10^{-4}	0.242	9.11×10^{-3}	-17.344	0.607	4.18×10^{-4}
G00-G99	Mobility	Enhanced	non-SRTI	16-17	Y	LG	0.018	1.25×10^{-4}	0.16	5.91×10^{-3}	-4.809	0.174	6.82×10^{-4}
G00-G99	Mobility	Enhanced	non-SRTI	16-17	N	LG	0.02	1.58×10^{-4}	0.175	7.65×10^{-3}	-5.338	0.229	8.80×10^{-4}
G00-G99	Mobility	Enhanced	non-SRTI	18-19	Y	LG	0.017	2.13×10^{-4}	0.111	5.05×10^{-3}	-3.899	0.163	8.52×10^{-4}
G00-G99	Mobility	Enhanced	non-SRTI	18-19	N	LG	0.019	2.20×10^{-4}	0.098	3.27×10^{-3}	-4.02	0.116	6.62×10^{-4}
G00-G99	Mobility	Enhanced	non-SRTI	20-24	Y	LG	0.029	4.10×10^{-4}	0.082	2.76×10^{-3}	-3.46	0.092	9.70×10^{-4}
G00-G99	Mobility	Enhanced	non-SRTI	20-24	N	LG	0.025	3.40×10^{-4}	0.082	2.32×10^{-3}	-3.776	0.083	6.70×10^{-4}
G00-G99	Mobility	Enhanced	non-SRTI	25-29	Y	LG	0.027	3.54×10^{-4}	0.083	2.62×10^{-3}	-3.433	0.087	8.51×10^{-4}
G00-G99	Mobility	Enhanced	non-SRTI	25-29	N	LG	0.022	3.11×10^{-4}	0.077	2.06×10^{-3}	-3.606	0.071	5.46×10^{-4}
G00-G99	Mobility	Enhanced	non-SRTI	30-34	Y	LG	0.042	5.82×10^{-4}	0.081	2.55×10^{-3}	-3.499	0.086	1.29×10^{-3}
G00-G99	Mobility	Enhanced	non-SRTI	30-34	N	LG	0.034	7.11×10^{-4}	0.07	2.26×10^{-3}	-3.464	0.075	9.54×10^{-4}
G00-G99	Mobility	Enhanced	non-SRTI	35-39	Y	LG	0.038	5.60×10^{-4}	0.082	2.67×10^{-3}	-3.574	0.091	1.23×10^{-3}
G00-G99	Mobility	Enhanced	non-SRTI	35-39	N	LG	0.029	3.36×10^{-4}	0.083	2.05×10^{-3}	-3.735	0.072	7.03×10^{-4}
G00-G99	Mobility	Enhanced	non-SRTI	40-44	Y	LG	0.052	5.48×10^{-4}	0.09	2.53×10^{-3}	-3.703	0.087	1.49×10^{-3}
G00-G99	Mobility	Enhanced	non-SRTI	40-44	N	LG	0.039	5.02×10^{-4}	0.083	2.42×10^{-3}	-3.633	0.084	1.13×10^{-3}
G00-G99	Mobility	Enhanced	non-SRTI	45-49	Y	LG	0.049	5.81×10^{-4}	0.086	2.32×10^{-3}	-3.772	0.082	1.32×10^{-3}
G00-G99	Mobility	Enhanced	non-SRTI	45-49	N	LG	0.039	4.84×10^{-4}	0.084	2.37×10^{-3}	-3.655	0.082	1.09×10^{-3}
G00-G99	Mobility	Enhanced	non-SRTI	50-54	Y	LG	0.062	7.95×10^{-4}	0.085	2.33×10^{-3}	-3.874	0.084	1.65×10^{-3}
G00-G99	Mobility	Enhanced	non-SRTI	50-54	N	LG	0.055	6.44×10^{-4}	0.087	2.41×10^{-3}	-3.797	0.085	1.51×10^{-3}

Continued on next page

Table 1 – continued from previous page

ICD-10 code	Component	Level	Rules	Ages	Female	Model type	A	s.e.	r	s.e.	k	s.e	RSE
G00-G99	Mobility	Enhanced	non-SRTI	55-59	Y	LG	0.062	7.73×10^{-4}	0.085	2.25×10^{-3}	-3.969	0.082	1.56×10^{-3}
G00-G99	Mobility	Enhanced	non-SRTI	55-59	N	LG	0.062	6.80×10^{-4}	0.09	2.44×10^{-3}	-3.88	0.087	1.69×10^{-3}
G00-G99	Mobility	Enhanced	non-SRTI	60-64	Y	LG	0.083	9.51×10^{-4}	0.087	2.02×10^{-3}	-4.193	0.077	1.80×10^{-3}
G00-G99	Mobility	Enhanced	non-SRTI	60-64	N	LG	0.097	1.07×10^{-3}	0.087	2.25×10^{-3}	-3.833	0.08	2.49×10^{-3}
G00-G99	Mobility	Enhanced	non-SRTI	65-69	Y	LG	0.055	3.94×10^{-4}	0.129	2.72×10^{-3}	-6.446	0.124	1.08×10^{-3}
G00-G99	Mobility	Enhanced	non-SRTI	65-69	N	LG	0.074	4.40×10^{-4}	0.131	2.38×10^{-3}	-6.414	0.107	1.27×10^{-3}
G00-G99	Mobility	Enhanced	non-SRTI	70+	Y	LG	6.08×10^{-3}	1.48×10^{-4}	0.233	9.77×10^{-3}	-16.682	0.647	1.33×10^{-4}
G00-G99	Mobility	Enhanced	non-SRTI	70+	N	LG	8.11×10^{-3}	1.65×10^{-4}	0.242	8.62×10^{-3}	-17.37	0.575	1.49×10^{-4}
G00-G99	Daily living	Standard	SRTI	16-17	Y	No model - fewer than 3 periods with positive claims							
G00-G99	Daily living	Standard	SRTI	16-17	N	No model - fewer than 3 periods with positive claims							
G00-G99	Daily living	Standard	SRTI	18-19	Y	No model - fewer than 3 periods with positive claims							
G00-G99	Daily living	Standard	SRTI	18-19	N	LM	3.96×10^{-4}	—	0.075	0.039	-4.041	2.729	0.254
G00-G99	Daily living	Standard	SRTI	20-24	Y	No model - fewer than 3 periods with positive claims							
G00-G99	Daily living	Standard	SRTI	20-24	N	LM	2.89×10^{-4}	—	0.389	0.283	-28.897	22.295	1.778
G00-G99	Daily living	Standard	SRTI	25-29	Y	No model - fewer than 3 periods with positive claims							
G00-G99	Daily living	Standard	SRTI	25-29	N	LM	1.14×10^{-4}	—	0.059	0.028	-1.854	1.815	1.309
G00-G99	Daily living	Standard	SRTI	30-34	Y	LM	2.09×10^{-4}	—	9.08×10^{-3}	6.76×10^{-3}	-0.965	0.42	0.548
G00-G99	Daily living	Standard	SRTI	30-34	N	LG	2.89×10^{-4}	1.86×10^{-5}	0.168	0.042	-8.053	1.949	6.80×10^{-5}
G00-G99	Daily living	Standard	SRTI	35-39	Y	LM	3.2×10^{-4}	—	0.029	0.013	-2.205	0.852	0.87
G00-G99	Daily living	Standard	SRTI	35-39	N	LM	3.63×10^{-4}	—	0.036	9.32×10^{-3}	-3.041	0.61	0.725
G00-G99	Daily living	Standard	SRTI	40-44	Y	LG	3.65×10^{-4}	1.26×10^{-5}	0.156	0.018	-8.14	0.864	3.71×10^{-5}
G00-G99	Daily living	Standard	SRTI	40-44	N	LG	6.62×10^{-4}	1.98×10^{-5}	0.102	0.01	-3.598	0.328	7.35×10^{-5}
G00-G99	Daily living	Standard	SRTI	45-49	Y	LG	3.87×10^{-4}	1.48×10^{-5}	0.078	6.47×10^{-3}	-3.306	0.211	3.17×10^{-5}
G00-G99	Daily living	Standard	SRTI	45-49	N	LG	6.49×10^{-4}	2.64×10^{-5}	0.067	6.35×10^{-3}	-2.436	0.169	5.93×10^{-5}
G00-G99	Daily living	Standard	SRTI	50-54	Y	LG	4.78×10^{-4}	1.24×10^{-5}	0.098	8.88×10^{-3}	-3.127	0.256	4.81×10^{-5}
G00-G99	Daily living	Standard	SRTI	50-54	N	LG	1.21×10^{-3}	1.62×10^{-5}	0.099	4.73×10^{-3}	-3.142	0.136	6.42×10^{-5}
G00-G99	Daily living	Standard	SRTI	55-59	Y	LG	8.68×10^{-4}	2.91×10^{-5}	0.065	4.40×10^{-3}	-2.566	0.12	5.48×10^{-5}
G00-G99	Daily living	Standard	SRTI	55-59	N	LG	1.96×10^{-3}	6.78×10^{-5}	0.057	3.35×10^{-3}	-2.346	0.083	9.70×10^{-5}
G00-G99	Daily living	Standard	SRTI	60-64	Y	LG	1.40×10^{-3}	2.75×10^{-5}	0.08	4.16×10^{-3}	-2.915	0.123	7.60×10^{-5}
G00-G99	Daily living	Standard	SRTI	60-64	N	LG	3.32×10^{-3}	1.15×10^{-4}	0.059	3.71×10^{-3}	-2.389	0.094	1.81×10^{-4}
G00-G99	Daily living	Standard	SRTI	65-69	Y	LG	7.66×10^{-4}	5.00×10^{-5}	0.086	8.02×10^{-3}	-4.93	0.334	5.48×10^{-5}
G00-G99	Daily living	Standard	SRTI	65-69	N	LG	1.41×10^{-3}	4.73×10^{-5}	0.085	5.86×10^{-3}	-4.069	0.218	9.10×10^{-5}
G00-G99	Daily living	Standard	SRTI	70+	Y	LG	4.10×10^{-5}	1.67×10^{-6}	0.382	0.047	-27.229	3.247	2.75×10^{-6}
G00-G99	Daily living	Standard	SRTI	70+	N	LG	7.00×10^{-5}	2.46×10^{-6}	0.8	0.137	-58.377	9.965	5.49×10^{-6}
G00-G99	Daily living	Standard	non-SRTI	16-17	Y	LG	0.065	6.29×10^{-4}	0.124	4.90×10^{-3}	-4.243	0.158	2.78×10^{-3}
G00-G99	Daily living	Standard	non-SRTI	16-17	N	LG	0.098	6.34×10^{-4}	0.138	4.03×10^{-3}	-4.638	0.129	3.03×10^{-3}
G00-G99	Daily living	Standard	non-SRTI	18-19	Y	LG	0.062	5.67×10^{-4}	0.086	1.59×10^{-3}	-4.228	0.061	1.04×10^{-3}
G00-G99	Daily living	Standard	non-SRTI	18-19	N	LG	0.086	4.68×10^{-4}	0.101	1.28×10^{-3}	-5.004	0.053	1.01×10^{-3}
G00-G99	Daily living	Standard	non-SRTI	20-24	Y	LG	0.093	1.78×10^{-3}	0.068	1.40×10^{-3}	-4.095	0.048	1.31×10^{-3}
G00-G99	Daily living	Standard	non-SRTI	20-24	N	LG	0.11	2.00×10^{-3}	0.077	1.61×10^{-3}	-4.619	0.062	1.60×10^{-3}
G00-G99	Daily living	Standard	non-SRTI	25-29	Y	LG	0.071	1.44×10^{-3}	0.067	1.51×10^{-3}	-3.966	0.051	1.11×10^{-3}
G00-G99	Daily living	Standard	non-SRTI	25-29	N	LG	0.079	1.13×10^{-3}	0.08	1.41×10^{-3}	-4.741	0.056	1.01×10^{-3}
G00-G99	Daily living	Standard	non-SRTI	30-34	Y	LG	0.094	1.96×10^{-3}	0.066	1.38×10^{-3}	-3.99	0.046	1.31×10^{-3}
G00-G99	Daily living	Standard	non-SRTI	30-34	N	LG	0.103	1.99×10^{-3}	0.072	1.43×10^{-3}	-4.488	0.053	1.34×10^{-3}
G00-G99	Daily living	Standard	non-SRTI	35-39	Y	LG	0.073	1.44×10^{-3}	0.068	1.60×10^{-3}	-3.9	0.054	1.25×10^{-3}
G00-G99	Daily living	Standard	non-SRTI	35-39	N	LG	0.086	1.32×10^{-3}	0.072	1.12×10^{-3}	-4.439	0.041	8.79×10^{-4}
G00-G99	Daily living	Standard	non-SRTI	40-44	Y	LG	0.09	1.56×10^{-3}	0.072	1.83×10^{-3}	-3.795	0.064	1.86×10^{-3}
G00-G99	Daily living	Standard	non-SRTI	40-44	N	LG	0.095	1.75×10^{-3}	0.071	1.68×10^{-3}	-3.993	0.059	1.71×10^{-3}
G00-G99	Daily living	Standard	non-SRTI	45-49	Y	LG	0.083	1.16×10^{-3}	0.075	1.59×10^{-3}	-4.003	0.058	1.44×10^{-3}

Continued on next page

Table 1 – continued from previous page

ICD-10 code	Component	Level	Rules	Ages	Female	Model type	A	s.e.	r	s.e.	k	s.e	RSE
G00-G99	Daily living	Standard	non-SRTI	45-49	N	LG	0.088	1.38×10^{-3}	0.074	1.65×10^{-3}	-4.033	0.06	1.55×10^{-3}
G00-G99	Daily living	Standard	non-SRTI	50-54	Y	LG	0.113	1.57×10^{-3}	0.078	1.71×10^{-3}	-4.169	0.064	2.04×10^{-3}
G00-G99	Daily living	Standard	non-SRTI	50-54	N	LG	0.122	1.73×10^{-3}	0.076	1.63×10^{-3}	-4.038	0.06	2.14×10^{-3}
G00-G99	Daily living	Standard	non-SRTI	55-59	Y	LG	0.118	1.79×10^{-3}	0.079	1.79×10^{-3}	-4.273	0.068	2.18×10^{-3}
G00-G99	Daily living	Standard	non-SRTI	55-59	N	LG	0.135	2.08×10^{-3}	0.076	1.82×10^{-3}	-3.935	0.066	2.73×10^{-3}
G00-G99	Daily living	Standard	non-SRTI	60-64	Y	LG	0.157	2.10×10^{-3}	0.084	1.96×10^{-3}	-4.415	0.077	3.13×10^{-3}
G00-G99	Daily living	Standard	non-SRTI	60-64	N	LG	0.205	2.75×10^{-3}	0.079	1.90×10^{-3}	-3.951	0.069	4.36×10^{-3}
G00-G99	Daily living	Standard	non-SRTI	65-69	Y	LG	0.115	7.83×10^{-4}	0.142	3.12×10^{-3}	-7.104	0.145	2.35×10^{-3}
G00-G99	Daily living	Standard	non-SRTI	65-69	N	LG	0.161	7.80×10^{-4}	0.143	2.30×10^{-3}	-6.978	0.105	2.44×10^{-3}
G00-G99	Daily living	Standard	non-SRTI	70+	Y	LG	0.014	3.16×10^{-4}	0.24	9.51×10^{-3}	-17.183	0.633	2.98×10^{-4}
G00-G99	Daily living	Standard	non-SRTI	70+	N	LG	0.021	4.46×10^{-4}	0.243	9.25×10^{-3}	-17.379	0.616	4.32×10^{-4}
G00-G99	Mobility	Standard	non-SRTI	16-17	Y	LG	9.61×10^{-3}	1.01×10^{-4}	0.223	0.016	-5.517	0.401	6.49×10^{-4}
G00-G99	Mobility	Standard	non-SRTI	16-17	N	LG	0.012	1.44×10^{-4}	0.262	0.024	-6.642	0.607	9.43×10^{-4}
G00-G99	Mobility	Standard	non-SRTI	18-19	Y	LG	9.07×10^{-3}	7.06×10^{-5}	0.143	5.41×10^{-3}	-4.31	0.158	3.66×10^{-4}
G00-G99	Mobility	Standard	non-SRTI	18-19	N	LG	0.011	9.07×10^{-5}	0.147	5.31×10^{-3}	-5.221	0.181	4.29×10^{-4}
G00-G99	Mobility	Standard	non-SRTI	20-24	Y	LG	0.013	1.40×10^{-4}	0.101	3.60×10^{-3}	-3.483	0.112	5.19×10^{-4}
G00-G99	Mobility	Standard	non-SRTI	20-24	N	LG	0.014	1.09×10^{-4}	0.107	2.79×10^{-3}	-4.26	0.099	3.73×10^{-4}
G00-G99	Mobility	Standard	non-SRTI	25-29	Y	LG	0.011	1.32×10^{-4}	0.098	3.89×10^{-3}	-3.385	0.12	4.78×10^{-4}
G00-G99	Mobility	Standard	non-SRTI	25-29	N	LG	0.01	1.08×10^{-4}	0.093	2.71×10^{-3}	-3.712	0.093	3.13×10^{-4}
G00-G99	Mobility	Standard	non-SRTI	30-34	Y	LG	0.018	1.72×10^{-4}	0.097	2.82×10^{-3}	-3.591	0.092	5.77×10^{-4}
G00-G99	Mobility	Standard	non-SRTI	30-34	N	LG	0.014	1.68×10^{-4}	0.089	2.95×10^{-3}	-3.429	0.096	4.93×10^{-4}
G00-G99	Mobility	Standard	non-SRTI	35-39	Y	LG	0.017	1.44×10^{-4}	0.108	3.37×10^{-3}	-3.644	0.105	5.78×10^{-4}
G00-G99	Mobility	Standard	non-SRTI	35-39	N	LG	0.012	1.08×10^{-4}	0.109	3.64×10^{-3}	-3.681	0.113	4.42×10^{-4}
G00-G99	Mobility	Standard	non-SRTI	40-44	Y	LG	0.025	1.74×10^{-4}	0.103	2.45×10^{-3}	-3.725	0.079	6.35×10^{-4}
G00-G99	Mobility	Standard	non-SRTI	40-44	N	LG	0.018	1.31×10^{-4}	0.108	2.83×10^{-3}	-3.761	0.09	5.12×10^{-4}
G00-G99	Mobility	Standard	non-SRTI	45-49	Y	LG	0.022	2.19×10^{-4}	0.095	2.89×10^{-3}	-3.525	0.093	7.28×10^{-4}
G00-G99	Mobility	Standard	non-SRTI	45-49	N	LG	0.018	1.50×10^{-4}	0.107	3.25×10^{-3}	-3.639	0.101	5.99×10^{-4}
G00-G99	Mobility	Standard	non-SRTI	50-54	Y	LG	0.029	2.17×10^{-4}	0.098	2.15×10^{-3}	-3.829	0.074	6.86×10^{-4}
G00-G99	Mobility	Standard	non-SRTI	50-54	N	LG	0.028	2.58×10^{-4}	0.094	2.64×10^{-3}	-3.501	0.085	8.42×10^{-4}
G00-G99	Mobility	Standard	non-SRTI	55-59	Y	LG	0.031	3.32×10^{-4}	0.09	2.54×10^{-3}	-3.678	0.087	8.96×10^{-4}
G00-G99	Mobility	Standard	non-SRTI	55-59	N	LG	0.032	2.73×10^{-4}	0.103	3.03×10^{-3}	-3.659	0.097	1.01×10^{-3}
G00-G99	Mobility	Standard	non-SRTI	60-64	Y	LG	0.04	3.04×10^{-4}	0.096	1.95×10^{-3}	-4.123	0.071	8.33×10^{-4}
G00-G99	Mobility	Standard	non-SRTI	60-64	N	LG	0.054	5.16×10^{-4}	0.095	2.77×10^{-3}	-3.584	0.091	1.68×10^{-3}
G00-G99	Mobility	Standard	non-SRTI	65-69	Y	LG	0.029	1.45×10^{-4}	0.137	2.25×10^{-3}	-6.596	0.1	4.51×10^{-4}
G00-G99	Mobility	Standard	non-SRTI	65-69	N	LG	0.044	1.43×10^{-4}	0.14	1.57×10^{-3}	-6.481	0.068	4.84×10^{-4}
G00-G99	Mobility	Standard	non-SRTI	70+	Y	LG	3.45×10^{-3}	8.36×10^{-5}	0.235	0.01	-16.749	0.676	7.98×10^{-5}
G00-G99	Mobility	Standard	non-SRTI	70+	N	LG	5.52×10^{-3}	1.18×10^{-4}	0.242	8.94×10^{-3}	-17.416	0.597	1.05×10^{-4}
H00-H59	Daily living	Enhanced	SRTI	16-17	Y	No model - fewer than 3 periods with positive claims							
H00-H59	Daily living	Enhanced	SRTI	16-17	N	No model - fewer than 3 periods with positive claims							
H00-H59	Daily living	Enhanced	SRTI	18-19	Y	No model - fewer than 3 periods with positive claims							
H00-H59	Daily living	Enhanced	SRTI	18-19	N	No model - fewer than 3 periods with positive claims							
H00-H59	Daily living	Enhanced	SRTI	20-24	Y	No model - fewer than 3 periods with positive claims							
H00-H59	Daily living	Enhanced	SRTI	20-24	N	No model - fewer than 3 periods with positive claims							
H00-H59	Daily living	Enhanced	SRTI	25-29	Y	No model - fewer than 3 periods with positive claims							
H00-H59	Daily living	Enhanced	SRTI	25-29	N	No model - fewer than 3 periods with positive claims							
H00-H59	Daily living	Enhanced	SRTI	30-34	Y	No model - fewer than 3 periods with positive claims							
H00-H59	Daily living	Enhanced	SRTI	30-34	N	No model - fewer than 3 periods with positive claims							
H00-H59	Daily living	Enhanced	SRTI	35-39	Y	No model - fewer than 3 periods with positive claims							
H00-H59	Daily living	Enhanced	SRTI	35-39	N	No model - fewer than 3 periods with positive claims							
H00-H59	Daily living	Enhanced	SRTI	40-44	Y	No model - fewer than 3 periods with positive claims							
H00-H59	Daily living	Enhanced	SRTI	40-44	N	No model - fewer than 3 periods with positive claims							

Continued on next page

Table 1 – continued from previous page

ICD-10 code	Component	Level	Rules	Ages	Female	Model type	A	s.e.	r	s.e.	k	s.e	RSE
H00-H59	Daily living	Enhanced	SRTI	45-49	Y	No model - fewer than 3 periods with positive claims							
H00-H59	Daily living	Enhanced	SRTI	45-49	N	No model - fewer than 3 periods with positive claims							
H00-H59	Daily living	Enhanced	SRTI	50-54	Y	No model - fewer than 3 periods with positive claims							
H00-H59	Daily living	Enhanced	SRTI	50-54	N	No model - fewer than 3 periods with positive claims							
H00-H59	Daily living	Enhanced	SRTI	55-59	Y	No model - fewer than 3 periods with positive claims							
H00-H59	Daily living	Enhanced	SRTI	55-59	N	No model - fewer than 3 periods with positive claims							
H00-H59	Daily living	Enhanced	SRTI	60-64	Y	No model - fewer than 3 periods with positive claims							
H00-H59	Daily living	Enhanced	SRTI	60-64	N	No model - fewer than 3 periods with positive claims							
H00-H59	Daily living	Enhanced	SRTI	65-69	Y	No model - fewer than 3 periods with positive claims							
H00-H59	Daily living	Enhanced	SRTI	65-69	N	LM	1.6×10^{-4}	—	-0.56	1.216	46.292	97.864	2.718
H00-H59	Daily living	Enhanced	SRTI	70+	Y	No model - fewer than 3 periods with positive claims							
H00-H59	Daily living	Enhanced	SRTI	70+	N	No model - fewer than 3 periods with positive claims							
H00-H59	Daily living	Enhanced	non-SRTI	16-17	Y	LG	0.031	3.42×10^{-4}	0.129	6.00×10^{-3}	-4.395	0.193	1.56×10^{-3}
H00-H59	Daily living	Enhanced	non-SRTI	16-17	N	LG	0.034	4.74×10^{-4}	0.103	4.51×10^{-3}	-4.008	0.156	1.58×10^{-3}
H00-H59	Daily living	Enhanced	non-SRTI	18-19	Y	LG	0.035	4.28×10^{-4}	0.113	3.92×10^{-3}	-5.26	0.162	1.18×10^{-3}
H00-H59	Daily living	Enhanced	non-SRTI	18-19	N	LG	0.031	5.59×10^{-4}	0.1	4.39×10^{-3}	-4.752	0.177	1.32×10^{-3}
H00-H59	Daily living	Enhanced	non-SRTI	20-24	Y	LG	0.048	5.78×10^{-4}	0.089	1.98×10^{-3}	-4.694	0.081	9.15×10^{-4}
H00-H59	Daily living	Enhanced	non-SRTI	20-24	N	LG	0.045	8.31×10^{-4}	0.077	2.07×10^{-3}	-4.194	0.078	9.77×10^{-4}
H00-H59	Daily living	Enhanced	non-SRTI	25-29	Y	LG	0.046	7.62×10^{-4}	0.09	2.66×10^{-3}	-4.834	0.11	1.15×10^{-3}
H00-H59	Daily living	Enhanced	non-SRTI	25-29	N	LG	0.042	7.52×10^{-4}	0.09	2.89×10^{-3}	-4.804	0.119	1.14×10^{-3}
H00-H59	Daily living	Enhanced	non-SRTI	30-34	Y	LG	0.069	1.23×10^{-3}	0.086	2.58×10^{-3}	-4.629	0.104	1.74×10^{-3}
H00-H59	Daily living	Enhanced	non-SRTI	30-34	N	LG	0.069	9.21×10^{-4}	0.086	1.91×10^{-3}	-4.644	0.077	1.27×10^{-3}
H00-H59	Daily living	Enhanced	non-SRTI	35-39	Y	LG	0.051	6.57×10^{-4}	0.089	2.05×10^{-3}	-4.704	0.084	1.02×10^{-3}
H00-H59	Daily living	Enhanced	non-SRTI	35-39	N	LG	0.056	9.40×10^{-4}	0.082	1.98×10^{-3}	-4.651	0.079	1.06×10^{-3}
H00-H59	Daily living	Enhanced	non-SRTI	40-44	Y	LG	0.053	5.50×10^{-4}	0.097	2.12×10^{-3}	-4.945	0.089	1.05×10^{-3}
H00-H59	Daily living	Enhanced	non-SRTI	40-44	N	LG	0.057	7.96×10^{-4}	0.094	2.58×10^{-3}	-4.866	0.107	1.38×10^{-3}
H00-H59	Daily living	Enhanced	non-SRTI	45-49	Y	LG	0.048	6.57×10^{-4}	0.091	2.31×10^{-3}	-4.759	0.095	1.07×10^{-3}
H00-H59	Daily living	Enhanced	non-SRTI	45-49	N	LG	0.048	5.60×10^{-4}	0.091	1.97×10^{-3}	-4.815	0.082	9.07×10^{-4}
H00-H59	Daily living	Enhanced	non-SRTI	50-54	Y	LG	0.061	1.05×10^{-3}	0.087	2.58×10^{-3}	-4.611	0.104	1.55×10^{-3}
H00-H59	Daily living	Enhanced	non-SRTI	50-54	N	LG	0.063	8.71×10^{-4}	0.091	2.29×10^{-3}	-4.837	0.095	1.37×10^{-3}
H00-H59	Daily living	Enhanced	non-SRTI	55-59	Y	LG	0.042	7.57×10^{-4}	0.08	2.10×10^{-3}	-4.452	0.082	8.83×10^{-4}
H00-H59	Daily living	Enhanced	non-SRTI	55-59	N	LG	0.04	6.34×10^{-4}	0.087	2.48×10^{-3}	-4.527	0.099	9.82×10^{-4}
H00-H59	Daily living	Enhanced	non-SRTI	60-64	Y	LG	0.051	6.15×10^{-4}	0.094	2.26×10^{-3}	-4.807	0.093	1.11×10^{-3}
H00-H59	Daily living	Enhanced	non-SRTI	60-64	N	LG	0.054	8.08×10^{-4}	0.088	2.40×10^{-3}	-4.6	0.097	1.27×10^{-3}
H00-H59	Daily living	Enhanced	non-SRTI	65-69	Y	LG	0.029	2.08×10^{-4}	0.148	3.44×10^{-3}	-7.495	0.163	6.22×10^{-4}
H00-H59	Daily living	Enhanced	non-SRTI	65-69	N	LG	0.029	2.38×10^{-4}	0.138	3.31×10^{-3}	-7.152	0.158	6.31×10^{-4}
H00-H59	Daily living	Enhanced	non-SRTI	70+	Y	LG	1.98×10^{-3}	3.64×10^{-5}	0.248	8.14×10^{-3}	-17.8	0.545	3.37×10^{-5}
H00-H59	Daily living	Enhanced	non-SRTI	70+	N	LG	1.88×10^{-3}	3.73×10^{-5}	0.25	9.34×10^{-3}	-17.88	0.625	3.71×10^{-5}
H00-H59	Mobility	Enhanced	non-SRTI	16-17	Y	LG	8.49×10^{-3}	2.28×10^{-4}	0.159	0.023	-4.503	0.636	1.28×10^{-3}
H00-H59	Mobility	Enhanced	non-SRTI	16-17	N	LG	8.93×10^{-3}	1.91×10^{-4}	0.305	0.054	-7.712	1.37	1.28×10^{-3}
H00-H59	Mobility	Enhanced	non-SRTI	18-19	Y	LG	9.85×10^{-3}	1.90×10^{-4}	0.158	0.016	-4.666	0.462	1.04×10^{-3}
H00-H59	Mobility	Enhanced	non-SRTI	18-19	N	LG	0.012	1.48×10^{-4}	0.174	0.01	-6.488	0.378	7.31×10^{-4}
H00-H59	Mobility	Enhanced	non-SRTI	20-24	Y	LG	0.015	2.17×10^{-4}	0.092	3.61×10^{-3}	-3.78	0.125	6.08×10^{-4}
H00-H59	Mobility	Enhanced	non-SRTI	20-24	N	LG	0.016	3.07×10^{-4}	0.083	3.03×10^{-3}	-4.077	0.113	5.24×10^{-4}
H00-H59	Mobility	Enhanced	non-SRTI	25-29	Y	LG	0.013	2.94×10^{-4}	0.079	3.48×10^{-3}	-3.704	0.122	5.27×10^{-4}
H00-H59	Mobility	Enhanced	non-SRTI	25-29	N	LG	9.47×10^{-3}	9.55×10^{-5}	0.123	4.55×10^{-3}	-4.847	0.166	3.68×10^{-4}
H00-H59	Mobility	Enhanced	non-SRTI	30-34	Y	LG	0.015	1.65×10^{-4}	0.118	4.68×10^{-3}	-4.511	0.165	6.38×10^{-4}
H00-H59	Mobility	Enhanced	non-SRTI	30-34	N	LG	0.017	2.60×10^{-4}	0.096	3.41×10^{-3}	-4.579	0.135	5.69×10^{-4}
H00-H59	Mobility	Enhanced	non-SRTI	35-39	Y	LG	0.013	2.89×10^{-4}	0.077	3.45×10^{-3}	-3.532	0.117	5.42×10^{-4}
H00-H59	Mobility	Enhanced	non-SRTI	35-39	N	LG	9.65×10^{-3}	2.41×10^{-4}	0.081	4.96×10^{-3}	-3.189	0.157	6.08×10^{-4}

Continued on next page

Table 1 – continued from previous page

ICD-10 code	Component	Level	Rules	Ages	Female	Model type	A	s.e.	r	s.e.	k	s.e	RSE
H00-H59	Mobility	Enhanced	non-SRTI	40-44	Y	LG	0.014	1.43×10^{-4}	0.109	3.67×10^{-3}	-4.37	0.132	4.86×10^{-4}
H00-H59	Mobility	Enhanced	non-SRTI	40-44	N	LG	0.011	1.51×10^{-4}	0.094	3.48×10^{-3}	-3.815	0.12	4.40×10^{-4}
H00-H59	Mobility	Enhanced	non-SRTI	45-49	Y	LG	0.011	1.18×10^{-4}	0.103	3.18×10^{-3}	-4.28	0.116	3.66×10^{-4}
H00-H59	Mobility	Enhanced	non-SRTI	45-49	N	LG	9.63×10^{-3}	1.08×10^{-4}	0.101	3.48×10^{-3}	-3.947	0.12	3.54×10^{-4}
H00-H59	Mobility	Enhanced	non-SRTI	50-54	Y	LG	0.013	1.05×10^{-4}	0.122	3.63×10^{-3}	-4.591	0.128	4.17×10^{-4}
H00-H59	Mobility	Enhanced	non-SRTI	50-54	N	LG	0.013	2.02×10^{-4}	0.085	3.20×10^{-3}	-3.645	0.11	4.81×10^{-4}
H00-H59	Mobility	Enhanced	non-SRTI	55-59	Y	LG	9.05×10^{-3}	9.17×10^{-5}	0.1	2.98×10^{-3}	-4.004	0.105	2.85×10^{-4}
H00-H59	Mobility	Enhanced	non-SRTI	55-59	N	LG	7.61×10^{-3}	9.22×10^{-5}	0.097	3.45×10^{-3}	-3.888	0.12	2.84×10^{-4}
H00-H59	Mobility	Enhanced	non-SRTI	60-64	Y	LG	0.012	1.23×10^{-4}	0.105	3.56×10^{-3}	-4.101	0.124	4.18×10^{-4}
H00-H59	Mobility	Enhanced	non-SRTI	60-64	N	LG	0.011	1.04×10^{-4}	0.1	2.80×10^{-3}	-4.039	0.099	3.23×10^{-4}
H00-H59	Mobility	Enhanced	non-SRTI	65-69	Y	LG	6.51×10^{-3}	3.39×10^{-5}	0.151	2.93×10^{-3}	-7.046	0.129	1.21×10^{-4}
H00-H59	Mobility	Enhanced	non-SRTI	65-69	N	LG	5.61×10^{-3}	4.72×10^{-5}	0.12	2.83×10^{-3}	-5.901	0.125	1.25×10^{-4}
H00-H59	Mobility	Enhanced	non-SRTI	70+	Y	LG	3.98×10^{-4}	9.18×10^{-6}	0.275	0.016	-19.011	1.071	1.40×10^{-5}
H00-H59	Mobility	Enhanced	non-SRTI	70+	N	LG	3.23×10^{-4}	8.98×10^{-6}	0.238	0.012	-16.89	0.826	9.24×10^{-6}
H00-H59	Daily living	Standard	SRTI	16-17	Y	No model - fewer than 3 periods with positive claims							
H00-H59	Daily living	Standard	SRTI	16-17	N	No model - fewer than 3 periods with positive claims							
H00-H59	Daily living	Standard	SRTI	18-19	Y	No model - fewer than 3 periods with positive claims							
H00-H59	Daily living	Standard	SRTI	18-19	N	No model - fewer than 3 periods with positive claims							
H00-H59	Daily living	Standard	SRTI	20-24	Y	No model - fewer than 3 periods with positive claims							
H00-H59	Daily living	Standard	SRTI	20-24	N	No model - fewer than 3 periods with positive claims							
H00-H59	Daily living	Standard	SRTI	25-29	Y	No model - fewer than 3 periods with positive claims							
H00-H59	Daily living	Standard	SRTI	25-29	N	No model - fewer than 3 periods with positive claims							
H00-H59	Daily living	Standard	SRTI	30-34	Y	No model - fewer than 3 periods with positive claims							
H00-H59	Daily living	Standard	SRTI	30-34	N	No model - fewer than 3 periods with positive claims							
H00-H59	Daily living	Standard	SRTI	35-39	Y	No model - fewer than 3 periods with positive claims							
H00-H59	Daily living	Standard	SRTI	35-39	N	No model - fewer than 3 periods with positive claims							
H00-H59	Daily living	Standard	SRTI	40-44	Y	No model - fewer than 3 periods with positive claims							
H00-H59	Daily living	Standard	SRTI	40-44	N	No model - fewer than 3 periods with positive claims							
H00-H59	Daily living	Standard	SRTI	45-49	Y	No model - fewer than 3 periods with positive claims							
H00-H59	Daily living	Standard	SRTI	45-49	N	No model - fewer than 3 periods with positive claims							
H00-H59	Daily living	Standard	SRTI	50-54	Y	No model - fewer than 3 periods with positive claims							
H00-H59	Daily living	Standard	SRTI	50-54	N	No model - fewer than 3 periods with positive claims							
H00-H59	Daily living	Standard	SRTI	55-59	Y	No model - fewer than 3 periods with positive claims							
H00-H59	Daily living	Standard	SRTI	55-59	N	No model - fewer than 3 periods with positive claims							
H00-H59	Daily living	Standard	SRTI	60-64	Y	No model - fewer than 3 periods with positive claims							
H00-H59	Daily living	Standard	SRTI	60-64	N	No model - fewer than 3 periods with positive claims							
H00-H59	Daily living	Standard	SRTI	65-69	Y	No model - fewer than 3 periods with positive claims							
H00-H59	Daily living	Standard	SRTI	65-69	N	No model - fewer than 3 periods with positive claims							
H00-H59	Daily living	Standard	SRTI	70+	Y	No model - fewer than 3 periods with positive claims							
H00-H59	Daily living	Standard	SRTI	70+	N	No model - fewer than 3 periods with positive claims							
H00-H59	Daily living	Standard	non-SRTI	16-17	Y	LG	0.045	3.98×10^{-4}	0.121	4.03×10^{-3}	-4.474	0.139	1.61×10^{-3}
H00-H59	Daily living	Standard	non-SRTI	16-17	N	LG	0.048	6.18×10^{-4}	0.104	3.95×10^{-3}	-4.321	0.145	1.91×10^{-3}
H00-H59	Daily living	Standard	non-SRTI	18-19	Y	LG	0.052	9.49×10^{-4}	0.087	2.99×10^{-3}	-4.402	0.117	1.59×10^{-3}
H00-H59	Daily living	Standard	non-SRTI	18-19	N	LG	0.047	7.44×10^{-4}	0.093	2.79×10^{-3}	-4.84	0.116	1.26×10^{-3}
H00-H59	Daily living	Standard	non-SRTI	20-24	Y	LG	0.066	8.80×10^{-4}	0.086	1.87×10^{-3}	-4.693	0.076	1.18×10^{-3}
H00-H59	Daily living	Standard	non-SRTI	20-24	N	LG	0.063	1.18×10^{-3}	0.077	1.90×10^{-3}	-4.441	0.073	1.17×10^{-3}
H00-H59	Daily living	Standard	non-SRTI	25-29	Y	LG	0.062	9.46×10^{-4}	0.089	2.27×10^{-3}	-4.869	0.095	1.30×10^{-3}
H00-H59	Daily living	Standard	non-SRTI	25-29	N	LG	0.055	9.37×10^{-4}	0.088	2.33×10^{-3}	-4.922	0.097	1.17×10^{-3}
H00-H59	Daily living	Standard	non-SRTI	30-34	Y	LG	0.092	1.55×10^{-3}	0.083	2.08×10^{-3}	-4.611	0.083	1.83×10^{-3}
H00-H59	Daily living	Standard	non-SRTI	30-34	N	LG	0.087	1.09×10^{-3}	0.087	1.66×10^{-3}	-4.899	0.069	1.31×10^{-3}
H00-H59	Daily living	Standard	non-SRTI	35-39	Y	LG	0.067	8.85×10^{-4}	0.086	1.87×10^{-3}	-4.705	0.076	1.19×10^{-3}
H00-H59	Daily living	Standard	non-SRTI	35-39	N	LG	0.066	1.00×10^{-3}	0.082	1.80×10^{-3}	-4.657	0.072	1.13×10^{-3}

Continued on next page

Table 1 – continued from previous page

ICD-10 code	Component	Level	Rules	Ages	Female	Model type	A	s.e.	r	s.e.	k	s.e	RSE
H00-H59	Daily living	Standard	non-SRTI	40-44	Y	LG	0.069	6.31×10^{-4}	0.098	1.89×10^{-3}	-5.068	0.08	1.18×10^{-3}
H00-H59	Daily living	Standard	non-SRTI	40-44	N	LG	0.068	9.14×10^{-4}	0.091	2.25×10^{-3}	-4.782	0.093	1.45×10^{-3}
H00-H59	Daily living	Standard	non-SRTI	45-49	Y	LG	0.059	9.07×10^{-4}	0.087	2.37×10^{-3}	-4.608	0.096	1.38×10^{-3}
H00-H59	Daily living	Standard	non-SRTI	45-49	N	LG	0.059	6.85×10^{-4}	0.091	1.93×10^{-3}	-4.89	0.081	1.06×10^{-3}
H00-H59	Daily living	Standard	non-SRTI	50-54	Y	LG	0.075	1.12×10^{-3}	0.088	2.30×10^{-3}	-4.742	0.094	1.65×10^{-3}
H00-H59	Daily living	Standard	non-SRTI	50-54	N	LG	0.075	9.39×10^{-4}	0.089	1.95×10^{-3}	-4.787	0.081	1.38×10^{-3}
H00-H59	Daily living	Standard	non-SRTI	55-59	Y	LG	0.052	8.57×10^{-4}	0.081	1.98×10^{-3}	-4.48	0.078	1.01×10^{-3}
H00-H59	Daily living	Standard	non-SRTI	55-59	N	LG	0.047	8.03×10^{-4}	0.086	2.57×10^{-3}	-4.579	0.103	1.19×10^{-3}
H00-H59	Daily living	Standard	non-SRTI	60-64	Y	LG	0.064	7.97×10^{-4}	0.09	2.07×10^{-3}	-4.78	0.085	1.26×10^{-3}
H00-H59	Daily living	Standard	non-SRTI	60-64	N	LG	0.063	9.23×10^{-4}	0.089	2.33×10^{-3}	-4.687	0.095	1.42×10^{-3}
H00-H59	Daily living	Standard	non-SRTI	65-69	Y	LG	0.034	2.43×10^{-4}	0.145	3.34×10^{-3}	-7.347	0.158	7.16×10^{-4}
H00-H59	Daily living	Standard	non-SRTI	65-69	N	LG	0.032	2.87×10^{-4}	0.134	3.37×10^{-3}	-7.033	0.161	7.30×10^{-4}
H00-H59	Daily living	Standard	non-SRTI	70+	Y	LG	2.24×10^{-3}	4.53×10^{-5}	0.246	8.95×10^{-3}	-17.672	0.599	4.24×10^{-5}
H00-H59	Daily living	Standard	non-SRTI	70+	N	LG	2.02×10^{-3}	3.95×10^{-5}	0.249	9.07×10^{-3}	-17.771	0.606	3.90×10^{-5}
H00-H59	Mobility	Standard	non-SRTI	16-17	Y	LG	5.61×10^{-3}	5.36×10^{-4}	-0.168	0.155	12.819	11.678	3.85×10^{-3}
H00-H59	Mobility	Standard	non-SRTI	16-17	N	LM	0.016	—	-7.01×10^{-3}	6.13×10^{-3}	0.167	0.321	0.994
H00-H59	Mobility	Standard	non-SRTI	18-19	Y	LG	9.87×10^{-3}	2.53×10^{-4}	0.186	0.029	-5.054	0.771	1.51×10^{-3}
H00-H59	Mobility	Standard	non-SRTI	18-19	N	LM	0.027	—	0.024	4.3×10^{-3}	-1.939	0.229	0.65
H00-H59	Mobility	Standard	non-SRTI	20-24	Y	LG	0.012	1.58×10^{-4}	0.163	0.011	-5.013	0.343	8.57×10^{-4}
H00-H59	Mobility	Standard	non-SRTI	20-24	N	LG	0.015	1.72×10^{-4}	0.108	4.09×10^{-3}	-4.275	0.145	5.94×10^{-4}
H00-H59	Mobility	Standard	non-SRTI	25-29	Y	LG	8.50×10^{-3}	1.36×10^{-4}	0.128	8.99×10^{-3}	-4.051	0.271	6.50×10^{-4}
H00-H59	Mobility	Standard	non-SRTI	25-29	N	LG	8.17×10^{-3}	9.50×10^{-5}	0.146	8.14×10^{-3}	-4.678	0.252	4.81×10^{-4}
H00-H59	Mobility	Standard	non-SRTI	30-34	Y	LG	9.88×10^{-3}	1.32×10^{-4}	0.169	0.013	-4.9	0.356	7.46×10^{-4}
H00-H59	Mobility	Standard	non-SRTI	30-34	N	LG	0.013	1.47×10^{-4}	0.118	4.85×10^{-3}	-4.301	0.164	5.91×10^{-4}
H00-H59	Mobility	Standard	non-SRTI	35-39	Y	LG	8.80×10^{-3}	1.21×10^{-4}	0.116	6.48×10^{-3}	-3.619	0.19	5.45×10^{-4}
H00-H59	Mobility	Standard	non-SRTI	35-39	N	LG	7.82×10^{-3}	1.01×10^{-4}	0.122	6.65×10^{-3}	-3.816	0.197	4.71×10^{-4}
H00-H59	Mobility	Standard	non-SRTI	40-44	Y	LG	9.14×10^{-3}	1.13×10^{-4}	0.145	8.61×10^{-3}	-4.565	0.262	5.77×10^{-4}
H00-H59	Mobility	Standard	non-SRTI	40-44	N	LG	8.14×10^{-3}	8.35×10^{-5}	0.154	8.09×10^{-3}	-4.669	0.238	4.47×10^{-4}
H00-H59	Mobility	Standard	non-SRTI	45-49	Y	LG	6.96×10^{-3}	7.33×10^{-5}	0.127	5.62×10^{-3}	-4.266	0.179	3.34×10^{-4}
H00-H59	Mobility	Standard	non-SRTI	45-49	N	LG	6.58×10^{-3}	7.47×10^{-5}	0.139	7.31×10^{-3}	-4.334	0.22	3.73×10^{-4}
H00-H59	Mobility	Standard	non-SRTI	50-54	Y	LG	8.04×10^{-3}	8.95×10^{-5}	0.175	0.011	-4.989	0.31	5.14×10^{-4}
H00-H59	Mobility	Standard	non-SRTI	50-54	N	LG	7.84×10^{-3}	6.06×10^{-5}	0.128	4.35×10^{-3}	-4.011	0.13	2.90×10^{-4}
H00-H59	Mobility	Standard	non-SRTI	55-59	Y	LG	4.76×10^{-3}	6.31×10^{-5}	0.151	0.01	-4.242	0.285	3.46×10^{-4}
H00-H59	Mobility	Standard	non-SRTI	55-59	N	LG	4.80×10^{-3}	4.75×10^{-5}	0.169	9.33×10^{-3}	-4.769	0.259	2.71×10^{-4}
H00-H59	Mobility	Standard	non-SRTI	60-64	Y	LG	6.45×10^{-3}	7.56×10^{-5}	0.156	9.37×10^{-3}	-4.758	0.278	4.04×10^{-4}
H00-H59	Mobility	Standard	non-SRTI	60-64	N	LG	6.92×10^{-3}	7.75×10^{-5}	0.163	9.82×10^{-3}	-4.826	0.284	4.29×10^{-4}
H00-H59	Mobility	Standard	non-SRTI	65-69	Y	LG	3.57×10^{-3}	2.48×10^{-5}	0.16	4.70×10^{-3}	-6.911	0.194	1.02×10^{-4}
H00-H59	Mobility	Standard	non-SRTI	65-69	N	LG	3.85×10^{-3}	2.66×10^{-5}	0.157	4.51×10^{-3}	-6.786	0.186	1.08×10^{-4}
H00-H59	Mobility	Standard	non-SRTI	70+	Y	LG	2.43×10^{-4}	3.88×10^{-6}	0.283	0.012	-19.499	0.794	6.19×10^{-6}
H00-H59	Mobility	Standard	non-SRTI	70+	N	LG	2.99×10^{-4}	1.04×10^{-5}	0.24	0.016	-16.925	1.088	1.14×10^{-5}
H60-H95	Daily living	Enhanced	SRTI	16-17	Y	No model - fewer than 3 periods with positive claims							
H60-H95	Daily living	Enhanced	SRTI	16-17	N	No model - fewer than 3 periods with positive claims							
H60-H95	Daily living	Enhanced	SRTI	18-19	Y	No model - fewer than 3 periods with positive claims							
H60-H95	Daily living	Enhanced	SRTI	18-19	N	No model - fewer than 3 periods with positive claims							
H60-H95	Daily living	Enhanced	SRTI	20-24	Y	No model - fewer than 3 periods with positive claims							
H60-H95	Daily living	Enhanced	SRTI	20-24	N	No model - fewer than 3 periods with positive claims							
H60-H95	Daily living	Enhanced	SRTI	25-29	Y	No model - fewer than 3 periods with positive claims							
H60-H95	Daily living	Enhanced	SRTI	25-29	N	No model - fewer than 3 periods with positive claims							
H60-H95	Daily living	Enhanced	SRTI	30-34	Y	No model - fewer than 3 periods with positive claims							
H60-H95	Daily living	Enhanced	SRTI	30-34	N	No model - fewer than 3 periods with positive claims							

Continued on next page

Table 1 – continued from previous page

ICD-10 code	Component	Level	Rules	Ages	Female	Model type	A	s.e.	r	s.e.	k	s.e	RSE
H60-H95	Daily living	Enhanced	SRTI	35-39	Y	No model - fewer than 3 periods with positive claims							
H60-H95	Daily living	Enhanced	SRTI	35-39	N	No model - fewer than 3 periods with positive claims							
H60-H95	Daily living	Enhanced	SRTI	40-44	Y	No model - fewer than 3 periods with positive claims							
H60-H95	Daily living	Enhanced	SRTI	40-44	N	No model - fewer than 3 periods with positive claims							
H60-H95	Daily living	Enhanced	SRTI	45-49	Y	No model - fewer than 3 periods with positive claims							
H60-H95	Daily living	Enhanced	SRTI	45-49	N	No model - fewer than 3 periods with positive claims							
H60-H95	Daily living	Enhanced	SRTI	50-54	Y	No model - fewer than 3 periods with positive claims							
H60-H95	Daily living	Enhanced	SRTI	50-54	N	No model - fewer than 3 periods with positive claims							
H60-H95	Daily living	Enhanced	SRTI	55-59	Y	No model - fewer than 3 periods with positive claims							
H60-H95	Daily living	Enhanced	SRTI	55-59	N	No model - fewer than 3 periods with positive claims							
H60-H95	Daily living	Enhanced	SRTI	60-64	Y	No model - fewer than 3 periods with positive claims							
H60-H95	Daily living	Enhanced	SRTI	60-64	N	No model - fewer than 3 periods with positive claims							
H60-H95	Daily living	Enhanced	SRTI	65-69	Y	No model - fewer than 3 periods with positive claims							
H60-H95	Daily living	Enhanced	SRTI	65-69	N	No model - fewer than 3 periods with positive claims							
H60-H95	Daily living	Enhanced	SRTI	70+	Y	No model - fewer than 3 periods with positive claims							
H60-H95	Daily living	Enhanced	SRTI	70+	N	No model - fewer than 3 periods with positive claims							
H60-H95	Daily living	Enhanced	non-SRTI	16-17	Y	LG	8.69×10^{-3}	2.76×10^{-4}	0.447	0.154	-9.217	3.17	2.02×10^{-3}
H60-H95	Daily living	Enhanced	non-SRTI	16-17	N	LG	6.77×10^{-3}	1.91×10^{-4}	0.373	0.102	-8.017	2.202	1.37×10^{-3}
H60-H95	Daily living	Enhanced	non-SRTI	18-19	Y	LG	0.012	1.54×10^{-4}	0.164	9.76×10^{-3}	-6.115	0.352	7.42×10^{-4}
H60-H95	Daily living	Enhanced	non-SRTI	18-19	N	LG	9.01×10^{-3}	1.32×10^{-4}	0.166	0.012	-6.193	0.426	6.38×10^{-4}
H60-H95	Daily living	Enhanced	non-SRTI	20-24	Y	LG	0.049	0.023	0.042	3.45×10^{-3}	-4.357	0.374	6.86×10^{-4}
H60-H95	Daily living	Enhanced	non-SRTI	20-24	N	LG	0.012	7.93×10^{-4}	0.067	3.58×10^{-3}	-4.456	0.119	3.53×10^{-4}
H60-H95	Daily living	Enhanced	non-SRTI	25-29	Y	LG	0.033	0.046	0.038	6.35×10^{-3}	-4.476	1.208	6.50×10^{-4}
H60-H95	Daily living	Enhanced	non-SRTI	25-29	N	LG	6.46×10^{-3}	5.80×10^{-4}	0.077	6.61×10^{-3}	-4.976	0.257	3.38×10^{-4}
H60-H95	Daily living	Enhanced	non-SRTI	30-34	Y	LG	9.90×10^{-3}	6.14×10^{-4}	0.066	4.55×10^{-3}	-3.819	0.149	4.84×10^{-4}
H60-H95	Daily living	Enhanced	non-SRTI	30-34	N	LG	6.34×10^{-3}	6.31×10^{-4}	0.057	4.43×10^{-3}	-3.648	0.12	2.84×10^{-4}
H60-H95	Daily living	Enhanced	non-SRTI	35-39	Y	LG	0.011	1.94×10^{-3}	0.049	3.92×10^{-3}	-3.703	0.106	3.65×10^{-4}
H60-H95	Daily living	Enhanced	non-SRTI	35-39	N	LG	3.28×10^{-3}	1.55×10^{-4}	0.078	6.48×10^{-3}	-3.876	0.234	2.42×10^{-4}
H60-H95	Daily living	Enhanced	non-SRTI	40-44	Y	LG	8.95×10^{-3}	5.53×10^{-4}	0.06	4.54×10^{-3}	-3.175	0.131	5.10×10^{-4}
H60-H95	Daily living	Enhanced	non-SRTI	40-44	N	LG	6.06×10^{-3}	7.76×10^{-4}	0.052	4.02×10^{-3}	-3.657	0.099	2.23×10^{-4}
H60-H95	Daily living	Enhanced	non-SRTI	45-49	Y	LG	8.07×10^{-3}	2.59×10^{-4}	0.071	3.74×10^{-3}	-3.523	0.126	3.67×10^{-4}
H60-H95	Daily living	Enhanced	non-SRTI	45-49	N	LG	3.71×10^{-3}	1.29×10^{-4}	0.075	4.28×10^{-3}	-3.8	0.152	1.83×10^{-4}
H60-H95	Daily living	Enhanced	non-SRTI	50-54	Y	LG	9.16×10^{-3}	5.92×10^{-4}	0.059	4.29×10^{-3}	-3.243	0.123	4.79×10^{-4}
H60-H95	Daily living	Enhanced	non-SRTI	50-54	N	LG	7.09×10^{-3}	1.59×10^{-3}	0.042	4.01×10^{-3}	-3.344	0.148	2.38×10^{-4}
H60-H95	Daily living	Enhanced	non-SRTI	55-59	Y	LG	7.04×10^{-3}	3.77×10^{-4}	0.058	3.31×10^{-3}	-3.275	0.093	2.78×10^{-4}
H60-H95	Daily living	Enhanced	non-SRTI	55-59	N	LG	4.32×10^{-3}	3.59×10^{-4}	0.054	3.15×10^{-3}	-3.641	0.081	1.32×10^{-4}
H60-H95	Daily living	Enhanced	non-SRTI	60-64	Y	LG	9.68×10^{-3}	3.21×10^{-4}	0.068	2.36×10^{-3}	-4.107	0.081	2.26×10^{-4}
H60-H95	Daily living	Enhanced	non-SRTI	60-64	N	LG	3.97×10^{-3}	2.14×10^{-4}	0.062	4.98×10^{-3}	-3.002	0.145	2.63×10^{-4}
H60-H95	Daily living	Enhanced	non-SRTI	65-69	Y	LG	4.42×10^{-3}	9.87×10^{-5}	0.107	4.86×10^{-3}	-5.875	0.223	1.70×10^{-4}
H60-H95	Daily living	Enhanced	non-SRTI	65-69	N	LG	2.89×10^{-3}	5.51×10^{-5}	0.096	3.43×10^{-3}	-5.208	0.148	8.74×10^{-5}
H60-H95	Daily living	Enhanced	non-SRTI	70+	Y	LG	3.20×10^{-4}	1.33×10^{-5}	0.216	0.01	-16.203	0.67	6.14×10^{-6}
H60-H95	Daily living	Enhanced	non-SRTI	70+	N	LG	1.99×10^{-4}	6.90×10^{-6}	0.233	0.015	-16.451	0.998	7.16×10^{-6}
H60-H95	Mobility	Enhanced	non-SRTI	16-17	Y	LG	0.016	2.56×10^{-4}	0.139	9.82×10^{-3}	-4.874	0.329	1.19×10^{-3}
H60-H95	Mobility	Enhanced	non-SRTI	16-17	N	LG	8.70×10^{-3}	1.47×10^{-4}	0.143	0.011	-4.588	0.352	7.36×10^{-4}
H60-H95	Mobility	Enhanced	non-SRTI	18-19	Y	LG	0.019	4.00×10^{-4}	0.109	6.43×10^{-3}	-4.961	0.258	1.12×10^{-3}
H60-H95	Mobility	Enhanced	non-SRTI	18-19	N	LG	0.01	1.70×10^{-4}	0.111	5.65×10^{-3}	-4.622	0.212	5.52×10^{-4}
H60-H95	Mobility	Enhanced	non-SRTI	20-24	Y	LG	0.019	3.69×10^{-4}	0.093	3.13×10^{-3}	-5.108	0.134	5.43×10^{-4}
H60-H95	Mobility	Enhanced	non-SRTI	20-24	N	LG	0.011	3.51×10^{-4}	0.081	3.82×10^{-3}	-4.543	0.151	4.08×10^{-4}
H60-H95	Mobility	Enhanced	non-SRTI	25-29	Y	LG	0.015	2.46×10^{-4}	0.095	3.24×10^{-3}	-4.786	0.133	4.73×10^{-4}
H60-H95	Mobility	Enhanced	non-SRTI	25-29	N	LG	7.72×10^{-3}	3.11×10^{-4}	0.079	4.31×10^{-3}	-4.519	0.168	3.20×10^{-4}
H60-H95	Mobility	Enhanced	non-SRTI	30-34	Y	LG	0.018	2.09×10^{-4}	0.115	3.81×10^{-3}	-5.479	0.161	5.69×10^{-4}

Continued on next page

Table 1 – continued from previous page

ICD-10 code	Component	Level	Rules	Ages	Female	Model type	A	s.e.	r	s.e.	k	s.e	RSE
H60-H95	Mobility	Enhanced	non-SRTI	30-34	N	LG	8.86×10^{-3}	2.11×10^{-4}	0.09	3.82×10^{-3}	-4.796	0.158	3.22×10^{-4}
H60-H95	Mobility	Enhanced	non-SRTI	35-39	Y	LG	0.01	1.91×10^{-4}	0.099	4.08×10^{-3}	-4.862	0.168	4.03×10^{-4}
H60-H95	Mobility	Enhanced	non-SRTI	35-39	N	LG	5.99×10^{-3}	1.84×10^{-4}	0.08	3.72×10^{-3}	-4.356	0.144	2.26×10^{-4}
H60-H95	Mobility	Enhanced	non-SRTI	40-44	Y	LG	0.013	2.90×10^{-4}	0.08	3.69×10^{-3}	-3.642	0.128	5.74×10^{-4}
H60-H95	Mobility	Enhanced	non-SRTI	40-44	N	LG	7.99×10^{-3}	1.52×10^{-4}	0.091	3.37×10^{-3}	-4.692	0.137	2.62×10^{-4}
H60-H95	Mobility	Enhanced	non-SRTI	45-49	Y	LG	0.013	2.06×10^{-4}	0.079	2.29×10^{-3}	-3.899	0.083	3.36×10^{-4}
H60-H95	Mobility	Enhanced	non-SRTI	45-49	N	LG	5.95×10^{-3}	8.12×10^{-5}	0.091	2.79×10^{-3}	-4.286	0.107	1.75×10^{-4}
H60-H95	Mobility	Enhanced	non-SRTI	50-54	Y	LG	0.013	1.75×10^{-4}	0.089	2.65×10^{-3}	-4.109	0.099	3.81×10^{-4}
H60-H95	Mobility	Enhanced	non-SRTI	50-54	N	LG	6.54×10^{-3}	1.35×10^{-4}	0.083	3.19×10^{-3}	-4.135	0.12	2.26×10^{-4}
H60-H95	Mobility	Enhanced	non-SRTI	55-59	Y	LG	0.011	2.35×10^{-4}	0.072	2.27×10^{-3}	-3.748	0.079	2.88×10^{-4}
H60-H95	Mobility	Enhanced	non-SRTI	55-59	N	LG	4.97×10^{-3}	1.15×10^{-4}	0.077	2.69×10^{-3}	-4.178	0.101	1.40×10^{-4}
H60-H95	Mobility	Enhanced	non-SRTI	60-64	Y	LG	0.011	1.16×10^{-4}	0.095	2.22×10^{-3}	-4.586	0.088	2.44×10^{-4}
H60-H95	Mobility	Enhanced	non-SRTI	60-64	N	LG	6.14×10^{-3}	9.92×10^{-5}	0.082	2.66×10^{-3}	-3.834	0.096	1.88×10^{-4}
H60-H95	Mobility	Enhanced	non-SRTI	65-69	Y	LG	6.48×10^{-3}	1.06×10^{-4}	0.115	4.59×10^{-3}	-5.987	0.209	2.33×10^{-4}
H60-H95	Mobility	Enhanced	non-SRTI	65-69	N	LG	3.97×10^{-3}	5.06×10^{-5}	0.119	3.86×10^{-3}	-6.161	0.176	1.17×10^{-4}
H60-H95	Mobility	Enhanced	non-SRTI	70+	Y	LG	4.85×10^{-4}	1.31×10^{-5}	0.237	0.012	-16.753	0.819	1.41×10^{-5}
H60-H95	Mobility	Enhanced	non-SRTI	70+	N	LG	2.89×10^{-4}	5.22×10^{-6}	0.306	0.014	-21.561	0.967	7.67×10^{-6}
H60-H95	Daily living	Standard	SRTI	16-17	Y	No model - fewer than 3 periods with positive claims							
H60-H95	Daily living	Standard	SRTI	16-17	N	No model - fewer than 3 periods with positive claims							
H60-H95	Daily living	Standard	SRTI	18-19	Y	No model - fewer than 3 periods with positive claims							
H60-H95	Daily living	Standard	SRTI	18-19	N	No model - fewer than 3 periods with positive claims							
H60-H95	Daily living	Standard	SRTI	20-24	Y	No model - fewer than 3 periods with positive claims							
H60-H95	Daily living	Standard	SRTI	20-24	N	No model - fewer than 3 periods with positive claims							
H60-H95	Daily living	Standard	SRTI	25-29	Y	No model - fewer than 3 periods with positive claims							
H60-H95	Daily living	Standard	SRTI	25-29	N	No model - fewer than 3 periods with positive claims							
H60-H95	Daily living	Standard	SRTI	30-34	Y	No model - fewer than 3 periods with positive claims							
H60-H95	Daily living	Standard	SRTI	30-34	N	No model - fewer than 3 periods with positive claims							
H60-H95	Daily living	Standard	SRTI	35-39	Y	No model - fewer than 3 periods with positive claims							
H60-H95	Daily living	Standard	SRTI	35-39	N	No model - fewer than 3 periods with positive claims							
H60-H95	Daily living	Standard	SRTI	40-44	Y	No model - fewer than 3 periods with positive claims							
H60-H95	Daily living	Standard	SRTI	40-44	N	No model - fewer than 3 periods with positive claims							
H60-H95	Daily living	Standard	SRTI	45-49	Y	No model - fewer than 3 periods with positive claims							
H60-H95	Daily living	Standard	SRTI	45-49	N	No model - fewer than 3 periods with positive claims							
H60-H95	Daily living	Standard	SRTI	50-54	Y	No model - fewer than 3 periods with positive claims							
H60-H95	Daily living	Standard	SRTI	50-54	N	No model - fewer than 3 periods with positive claims							
H60-H95	Daily living	Standard	SRTI	55-59	Y	No model - fewer than 3 periods with positive claims							
H60-H95	Daily living	Standard	SRTI	55-59	N	No model - fewer than 3 periods with positive claims							
H60-H95	Daily living	Standard	SRTI	60-64	Y	No model - fewer than 3 periods with positive claims							
H60-H95	Daily living	Standard	SRTI	60-64	N	No model - fewer than 3 periods with positive claims							
H60-H95	Daily living	Standard	SRTI	65-69	Y	No model - fewer than 3 periods with positive claims							
H60-H95	Daily living	Standard	SRTI	65-69	N	No model - fewer than 3 periods with positive claims							
H60-H95	Daily living	Standard	SRTI	70+	Y	No model - fewer than 3 periods with positive claims							
H60-H95	Daily living	Standard	SRTI	70+	N	No model - fewer than 3 periods with positive claims							
H60-H95	Daily living	Standard	non-SRTI	16-17	Y	LG	9.73×10^{-3}	2.81×10^{-3}	0.042	9.04×10^{-3}	-2.654	0.213	1.05×10^{-3}
H60-H95	Daily living	Standard	non-SRTI	16-17	N	LM	0.011	—	0.026	3.04×10^{-3}	-2.503	0.161	0.47
H60-H95	Daily living	Standard	non-SRTI	18-19	Y	LG	5.55×10^{-3}	1.19×10^{-4}	0.197	0.02	-8.889	0.889	5.21×10^{-4}
H60-H95	Daily living	Standard	non-SRTI	18-19	N	LG	3.18×10^{-3}	8.12×10^{-5}	0.186	0.024	-7.247	0.918	3.96×10^{-4}
H60-H95	Daily living	Standard	non-SRTI	20-24	Y	LG	9.17×10^{-3}	2.08×10^{-3}	0.064	6.80×10^{-3}	-5.01	0.19	3.63×10^{-4}
H60-H95	Daily living	Standard	non-SRTI	20-24	N	LG	0.011	6.53×10^{-3}	0.069	9.00×10^{-3}	-6.269	0.284	2.87×10^{-4}
H60-H95	Daily living	Standard	non-SRTI	25-29	Y	LM	5.65×10^{-3}	—	0.034	3.26×10^{-3}	-2.944	0.174	0.493
H60-H95	Daily living	Standard	non-SRTI	25-29	N	LG	1.87×10^{-3}	9.57×10^{-5}	0.124	0.012	-7.408	0.636	1.43×10^{-4}
H60-H95	Daily living	Standard	non-SRTI	30-34	Y	LG	3.99×10^{-3}	1.92×10^{-4}	0.11	0.013	-5.459	0.576	4.54×10^{-4}

Continued on next page

Table 1 – continued from previous page

ICD-10 code	Component	Level	Rules	Ages	Female	Model type	A	s.e.	r	s.e.	k	s.e	RSE
H60-H95	Daily living	Standard	non-SRTI	30-34	N	LG	1.64×10^{-3}	1.05×10^{-4}	0.237	0.037	-16.037	2.365	1.60×10^{-4}
H60-H95	Daily living	Standard	non-SRTI	35-39	Y	LG	3.40×10^{-3}	2.55×10^{-4}	0.069	6.93×10^{-3}	-3.685	0.233	2.71×10^{-4}
H60-H95	Daily living	Standard	non-SRTI	35-39	N	LG	3.02×10^{-3}	1.39×10^{-3}	0.045	7.16×10^{-3}	-3.865	0.293	1.45×10^{-4}
H60-H95	Daily living	Standard	non-SRTI	40-44	Y	LG	5.16×10^{-3}	2.92×10^{-4}	0.065	5.89×10^{-3}	-3.111	0.179	3.99×10^{-4}
H60-H95	Daily living	Standard	non-SRTI	40-44	N	LG	2.16×10^{-3}	2.43×10^{-4}	0.062	7.22×10^{-3}	-3.631	0.221	1.71×10^{-4}
H60-H95	Daily living	Standard	non-SRTI	45-49	Y	LG	5.91×10^{-3}	3.93×10^{-4}	0.058	4.33×10^{-3}	-3.233	0.123	3.11×10^{-4}
H60-H95	Daily living	Standard	non-SRTI	45-49	N	LG	2.06×10^{-3}	8.81×10^{-5}	0.08	5.25×10^{-3}	-4.349	0.203	1.10×10^{-4}
H60-H95	Daily living	Standard	non-SRTI	50-54	Y	LG	6.90×10^{-3}	5.08×10^{-4}	0.054	4.15×10^{-3}	-3.033	0.107	3.56×10^{-4}
H60-H95	Daily living	Standard	non-SRTI	50-54	N	LG	3.40×10^{-3}	9.70×10^{-4}	0.04	5.87×10^{-3}	-2.931	0.196	1.97×10^{-4}
H60-H95	Daily living	Standard	non-SRTI	55-59	Y	LG	5.45×10^{-3}	2.60×10^{-4}	0.061	3.35×10^{-3}	-3.35	0.099	2.19×10^{-4}
H60-H95	Daily living	Standard	non-SRTI	55-59	N	LG	2.23×10^{-3}	1.24×10^{-4}	0.064	4.17×10^{-3}	-3.57	0.131	1.07×10^{-4}
H60-H95	Daily living	Standard	non-SRTI	60-64	Y	LG	7.96×10^{-3}	2.35×10^{-4}	0.076	2.55×10^{-3}	-4.598	0.098	1.84×10^{-4}
H60-H95	Daily living	Standard	non-SRTI	60-64	N	LG	3.88×10^{-3}	2.43×10^{-4}	0.06	3.65×10^{-3}	-3.552	0.107	1.56×10^{-4}
H60-H95	Daily living	Standard	non-SRTI	65-69	Y	LG	3.94×10^{-3}	1.12×10^{-4}	0.107	5.01×10^{-3}	-6.334	0.241	1.40×10^{-4}
H60-H95	Daily living	Standard	non-SRTI	65-69	N	LG	2.70×10^{-3}	9.67×10^{-5}	0.088	4.08×10^{-3}	-5.36	0.177	8.78×10^{-5}
H60-H95	Daily living	Standard	non-SRTI	70+	Y	LG	2.48×10^{-4}	9.46×10^{-6}	0.252	0.014	-18.664	0.952	6.21×10^{-6}
H60-H95	Daily living	Standard	non-SRTI	70+	N	LG	1.34×10^{-4}	2.12×10^{-6}	0.322	0.014	-22.467	0.98	3.52×10^{-6}
H60-H95	Mobility	Standard	non-SRTI	16-17	Y	LM	0.03	—	0.041	5.44×10^{-3}	-3.031	0.289	0.842
H60-H95	Mobility	Standard	non-SRTI	16-17	N	LM	0.02	—	0.036	5.15×10^{-3}	-2.751	0.275	0.778
H60-H95	Mobility	Standard	non-SRTI	18-19	Y	LG	0.011	2.09×10^{-4}	0.151	0.014	-5.266	0.466	1.01×10^{-3}
H60-H95	Mobility	Standard	non-SRTI	18-19	N	LG	6.96×10^{-3}	1.20×10^{-4}	0.157	0.013	-5.604	0.446	5.85×10^{-4}
H60-H95	Mobility	Standard	non-SRTI	20-24	Y	LG	0.077	0.034	0.054	3.21×10^{-3}	-5.643	0.316	5.97×10^{-4}
H60-H95	Mobility	Standard	non-SRTI	20-24	N	LG	0.023	4.32×10^{-3}	0.06	3.44×10^{-3}	-5.155	0.102	3.47×10^{-4}
H60-H95	Mobility	Standard	non-SRTI	25-29	Y	LM	0.02	—	0.066	2.09×10^{-3}	-5.634	0.114	0.287
H60-H95	Mobility	Standard	non-SRTI	25-29	N	LG	0.022	6.61×10^{-3}	0.07	4.28×10^{-3}	-6.461	0.147	2.40×10^{-4}
H60-H95	Mobility	Standard	non-SRTI	30-34	Y	LM	0.024	—	0.059	2.09×10^{-3}	-4.978	0.116	0.275
H60-H95	Mobility	Standard	non-SRTI	30-34	N	LG	8.90×10^{-3}	7.06×10^{-4}	0.096	6.63×10^{-3}	-6.678	0.308	3.17×10^{-4}
H60-H95	Mobility	Standard	non-SRTI	35-39	Y	LM	0.018	—	0.055	1.87×10^{-3}	-4.939	0.099	0.29
H60-H95	Mobility	Standard	non-SRTI	35-39	N	LG	0.013	3.12×10^{-3}	0.062	3.48×10^{-3}	-5.68	0.125	1.53×10^{-4}
H60-H95	Mobility	Standard	non-SRTI	40-44	Y	LM	0.01	—	0.088	6.11×10^{-3}	-5.226	0.335	0.856
H60-H95	Mobility	Standard	non-SRTI	40-44	N	LM	7.14×10^{-3}	—	0.067	3.07×10^{-3}	-5.314	0.166	0.463
H60-H95	Mobility	Standard	non-SRTI	45-49	Y	LG	0.023	6.94×10^{-3}	0.043	2.88×10^{-3}	-4.157	0.228	3.19×10^{-4}
H60-H95	Mobility	Standard	non-SRTI	45-49	N	LG	0.124	0.764	0.042	4.11×10^{-3}	-6.843	6.012	2.09×10^{-4}
H60-H95	Mobility	Standard	non-SRTI	50-54	Y	LG	0.135	0.335	0.037	3.14×10^{-3}	-5.711	2.388	4.03×10^{-4}
H60-H95	Mobility	Standard	non-SRTI	50-54	N	LM	5.92×10^{-3}	—	0.064	1.97×10^{-3}	-4.615	0.107	0.284
H60-H95	Mobility	Standard	non-SRTI	55-59	Y	LG	0.01	1.06×10^{-3}	0.051	2.86×10^{-3}	-3.778	0.072	2.45×10^{-4}
H60-H95	Mobility	Standard	non-SRTI	55-59	N	LM	4.23×10^{-3}	—	0.052	2.31×10^{-3}	-3.96	0.124	0.341
H60-H95	Mobility	Standard	non-SRTI	60-64	Y	LG	0.016	2.86×10^{-3}	0.047	2.63×10^{-3}	-4.086	0.114	2.52×10^{-4}
H60-H95	Mobility	Standard	non-SRTI	60-64	N	LG	0.013	7.02×10^{-3}	0.038	3.63×10^{-3}	-4.04	0.467	1.97×10^{-4}
H60-H95	Mobility	Standard	non-SRTI	65-69	Y	LG	4.23×10^{-3}	1.10×10^{-4}	0.101	4.65×10^{-3}	-5.693	0.211	1.58×10^{-4}
H60-H95	Mobility	Standard	non-SRTI	65-69	N	LG	3.16×10^{-3}	1.06×10^{-4}	0.081	2.97×10^{-3}	-5.133	0.122	7.45×10^{-5}
H60-H95	Mobility	Standard	non-SRTI	70+	Y	LG	3.22×10^{-4}	1.48×10^{-5}	0.211	0.012	-15.608	0.775	7.91×10^{-6}
H60-H95	Mobility	Standard	non-SRTI	70+	N	LG	1.58×10^{-4}	4.64×10^{-6}	0.272	0.017	-19.332	1.172	5.54×10^{-6}
I00-I99	Daily living	Enhanced	SRTI	16-17	Y	No model - fewer than 3 periods with positive claims							
I00-I99	Daily living	Enhanced	SRTI	16-17	N	No model - fewer than 3 periods with positive claims							
I00-I99	Daily living	Enhanced	SRTI	18-19	Y	No model - fewer than 3 periods with positive claims							
I00-I99	Daily living	Enhanced	SRTI	18-19	N	No model - fewer than 3 periods with positive claims							
I00-I99	Daily living	Enhanced	SRTI	20-24	Y	No model - fewer than 3 periods with positive claims							
I00-I99	Daily living	Enhanced	SRTI	20-24	N	No model - fewer than 3 periods with positive claims							
I00-I99	Daily living	Enhanced	SRTI	25-29	Y	No model - fewer than 3 periods with positive claims							

Continued on next page

Table 1 – continued from previous page

ICD-10 code	Component	Level	Rules	Ages	Female	Model type	A	s.e.	r	s.e.	k	s.e	RSE
I00-I99	Daily living	Enhanced	SRTI	25-29	N	No model - fewer than 3 periods with positive claims							
I00-I99	Daily living	Enhanced	SRTI	30-34	Y	No model - fewer than 3 periods with positive claims							
I00-I99	Daily living	Enhanced	SRTI	30-34	N	LM	1.42×10^{-4}	—	-0.412	0.022	38.472	1.74	0.192
I00-I99	Daily living	Enhanced	SRTI	35-39	Y	No model - fewer than 3 periods with positive claims							
I00-I99	Daily living	Enhanced	SRTI	35-39	N	LG	3.57×10^{-5}	6.64×10^{-6}	0.292	0.258	-16.712	14.546	2.37×10^{-5}
I00-I99	Daily living	Enhanced	SRTI	40-44	Y	LM	6.83×10^{-5}	—	0.052	8.43×10^{-4}	0.597	0.04	0.029
I00-I99	Daily living	Enhanced	SRTI	40-44	N	LM	1.27×10^{-4}	—	9.93×10^{-3}	0.021	-0.134	1.057	1.064
I00-I99	Daily living	Enhanced	SRTI	45-49	Y	LG	4.17×10^{-5}	3.60×10^{-6}	0.238	0.154	-5.09	3.297	2.43×10^{-5}
I00-I99	Daily living	Enhanced	SRTI	45-49	N	LG	6.75×10^{-5}	6.21×10^{-6}	0.12	0.031	-5.904	1.359	1.64×10^{-5}
I00-I99	Daily living	Enhanced	SRTI	50-54	Y	LG	6.02×10^{-5}	6.93×10^{-6}	0.088	0.025	-3.805	0.872	1.69×10^{-5}
I00-I99	Daily living	Enhanced	SRTI	50-54	N	LG	1.03×10^{-4}	3.11×10^{-6}	0.23	0.045	-7.294	1.426	1.83×10^{-5}
I00-I99	Daily living	Enhanced	SRTI	55-59	Y	LG	4.63×10^{-5}	2.04×10^{-6}	0.351	0.116	-14.481	4.753	1.14×10^{-5}
I00-I99	Daily living	Enhanced	SRTI	55-59	N	LG	9.82×10^{-5}	2.36×10^{-6}	0.119	0.013	-3.023	0.325	1.21×10^{-5}
I00-I99	Daily living	Enhanced	SRTI	60-64	Y	LG	8.54×10^{-5}	2.24×10^{-6}	0.374	0.08	-14.247	3.017	1.32×10^{-5}
I00-I99	Daily living	Enhanced	SRTI	60-64	N	LG	1.65×10^{-4}	3.00×10^{-6}	0.143	0.013	-3.735	0.34	1.66×10^{-5}
I00-I99	Daily living	Enhanced	SRTI	65-69	Y	LG	4.17×10^{-5}	1.69×10^{-6}	0.267	0.068	-11.099	2.786	8.82×10^{-6}
I00-I99	Daily living	Enhanced	SRTI	65-69	N	LG	1.15×10^{-4}	3.74×10^{-6}	0.101	7.93×10^{-3}	-4.804	0.321	8.71×10^{-6}
I00-I99	Daily living	Enhanced	SRTI	70+	Y	LM	5.27×10^{-6}	—	0.109	0.124	-7.263	9.632	1.205
I00-I99	Daily living	Enhanced	SRTI	70+	N	LG	1.42×10^{-5}	5.44×10^{-7}	0.3	0.029	-21.068	1.971	7.94×10^{-7}
I00-I99	Daily living	Enhanced	non-SRTI	16-17	Y	LG	3.24×10^{-3}	9.29×10^{-5}	0.816	0.337	-20.159	8.323	6.79×10^{-4}
I00-I99	Daily living	Enhanced	non-SRTI	16-17	N	LG	3.61×10^{-3}	8.33×10^{-5}	0.37	0.079	-9.484	2.029	5.71×10^{-4}
I00-I99	Daily living	Enhanced	non-SRTI	18-19	Y	LG	3.00×10^{-3}	1.02×10^{-4}	0.138	0.019	-5.334	0.701	4.31×10^{-4}
I00-I99	Daily living	Enhanced	non-SRTI	18-19	N	LG	2.95×10^{-3}	8.89×10^{-5}	0.133	0.015	-5.672	0.584	3.30×10^{-4}
I00-I99	Daily living	Enhanced	non-SRTI	20-24	Y	LG	6.73×10^{-3}	6.86×10^{-4}	0.056	5.35×10^{-3}	-3.379	0.146	4.06×10^{-4}
I00-I99	Daily living	Enhanced	non-SRTI	20-24	N	LG	3.06×10^{-3}	1.26×10^{-4}	0.083	6.81×10^{-3}	-3.918	0.248	2.36×10^{-4}
I00-I99	Daily living	Enhanced	non-SRTI	25-29	Y	LG	3.70×10^{-3}	1.09×10^{-4}	0.088	6.81×10^{-3}	-3.55	0.228	2.96×10^{-4}
I00-I99	Daily living	Enhanced	non-SRTI	25-29	N	LG	3.27×10^{-3}	2.49×10^{-4}	0.065	5.22×10^{-3}	-3.845	0.169	1.80×10^{-4}
I00-I99	Daily living	Enhanced	non-SRTI	30-34	Y	LG	3.94×10^{-3}	8.63×10^{-5}	0.103	8.67×10^{-3}	-3.094	0.24	3.69×10^{-4}
I00-I99	Daily living	Enhanced	non-SRTI	30-34	N	LG	2.47×10^{-3}	5.58×10^{-5}	0.101	8.03×10^{-3}	-3.356	0.241	2.17×10^{-4}
I00-I99	Daily living	Enhanced	non-SRTI	35-39	Y	LG	3.45×10^{-3}	8.08×10^{-5}	0.081	5.01×10^{-3}	-3.022	0.152	2.22×10^{-4}
I00-I99	Daily living	Enhanced	non-SRTI	35-39	N	LG	2.50×10^{-3}	7.02×10^{-5}	0.076	5.24×10^{-3}	-2.878	0.155	1.75×10^{-4}
I00-I99	Daily living	Enhanced	non-SRTI	40-44	Y	LG	3.14×10^{-3}	5.42×10^{-5}	0.098	6.04×10^{-3}	-3.097	0.172	2.14×10^{-4}
I00-I99	Daily living	Enhanced	non-SRTI	40-44	N	LG	2.68×10^{-3}	3.22×10^{-5}	0.121	6.72×10^{-3}	-3.147	0.169	1.65×10^{-4}
I00-I99	Daily living	Enhanced	non-SRTI	45-49	Y	LG	2.76×10^{-3}	5.93×10^{-5}	0.082	5.09×10^{-3}	-2.824	0.146	1.81×10^{-4}
I00-I99	Daily living	Enhanced	non-SRTI	45-49	N	LG	2.65×10^{-3}	3.65×10^{-5}	0.112	6.48×10^{-3}	-3.132	0.172	1.72×10^{-4}
I00-I99	Daily living	Enhanced	non-SRTI	50-54	Y	LG	2.95×10^{-3}	3.78×10^{-5}	0.101	4.67×10^{-3}	-3.173	0.134	1.52×10^{-4}
I00-I99	Daily living	Enhanced	non-SRTI	50-54	N	LG	3.14×10^{-3}	5.64×10^{-5}	0.096	6.32×10^{-3}	-2.806	0.169	2.30×10^{-4}
I00-I99	Daily living	Enhanced	non-SRTI	55-59	Y	LG	2.48×10^{-3}	3.58×10^{-5}	0.092	4.29×10^{-3}	-3.077	0.126	1.27×10^{-4}
I00-I99	Daily living	Enhanced	non-SRTI	55-59	N	LG	3.09×10^{-3}	4.52×10^{-5}	0.093	4.65×10^{-3}	-2.951	0.131	1.70×10^{-4}
I00-I99	Daily living	Enhanced	non-SRTI	60-64	Y	LG	3.14×10^{-3}	5.15×10^{-5}	0.082	3.38×10^{-3}	-3.178	0.106	1.34×10^{-4}
I00-I99	Daily living	Enhanced	non-SRTI	60-64	N	LG	5.07×10^{-3}	6.25×10^{-5}	0.094	3.71×10^{-3}	-3.204	0.112	2.20×10^{-4}
I00-I99	Daily living	Enhanced	non-SRTI	65-69	Y	LG	2.24×10^{-3}	1.09×10^{-5}	0.131	2.07×10^{-3}	-6.109	0.089	3.45×10^{-5}
I00-I99	Daily living	Enhanced	non-SRTI	65-69	N	LG	3.98×10^{-3}	2.14×10^{-5}	0.122	1.91×10^{-3}	-5.821	0.083	6.05×10^{-5}
I00-I99	Daily living	Enhanced	non-SRTI	70+	Y	LG	2.56×10^{-4}	5.50×10^{-6}	0.249	0.011	-17.607	0.714	6.10×10^{-6}
I00-I99	Daily living	Enhanced	non-SRTI	70+	N	LG	4.96×10^{-4}	8.84×10^{-6}	0.259	9.52×10^{-3}	-18.381	0.637	1.00×10^{-5}
I00-I99	Mobility	Enhanced	non-SRTI	16-17	Y	LG	2.01×10^{-3}	1.11×10^{-4}	0.625	0.445	-13.552	9.652	8.26×10^{-4}
I00-I99	Mobility	Enhanced	non-SRTI	16-17	N	LM	3.75×10^{-3}	—	-9.34×10^{-3}	0.012	0.102	0.696	0.897
I00-I99	Mobility	Enhanced	non-SRTI	18-19	Y	LG	1.87×10^{-3}	6.57×10^{-5}	0.346	0.109	-8.755	2.76	4.49×10^{-4}
I00-I99	Mobility	Enhanced	non-SRTI	18-19	N	LG	1.82×10^{-3}	1.88×10^{-4}	0.108	0.019	-6.404	0.921	2.45×10^{-4}

Continued on next page

Table 1 – continued from previous page

ICD-10 code	Component	Level	Rules	Ages	Female	Model type	A	s.e.	r	s.e.	k	s.e	RSE
I00-I99	Mobility	Enhanced	non-SRTI	20-24	Y	LG	4.69×10^{-3}	9.07×10^{-5}	0.127	0.011	-4.107	0.324	4.24×10^{-4}
I00-I99	Mobility	Enhanced	non-SRTI	20-24	N	LG	2.72×10^{-3}	3.91×10^{-4}	0.068	9.90×10^{-3}	-4.119	0.337	2.64×10^{-4}
I00-I99	Mobility	Enhanced	non-SRTI	25-29	Y	LG	5.91×10^{-3}	9.67×10^{-5}	0.11	6.78×10^{-3}	-3.603	0.206	4.04×10^{-4}
I00-I99	Mobility	Enhanced	non-SRTI	25-29	N	LG	3.07×10^{-3}	1.71×10^{-4}	0.068	7.04×10^{-3}	-2.969	0.212	2.92×10^{-4}
I00-I99	Mobility	Enhanced	non-SRTI	30-34	Y	LG	7.71×10^{-3}	1.25×10^{-4}	0.087	3.74×10^{-3}	-3.439	0.123	3.46×10^{-4}
I00-I99	Mobility	Enhanced	non-SRTI	30-34	N	LG	4.22×10^{-3}	8.52×10^{-5}	0.095	6.01×10^{-3}	-3.427	0.19	2.89×10^{-4}
I00-I99	Mobility	Enhanced	non-SRTI	35-39	Y	LG	6.19×10^{-3}	9.05×10^{-5}	0.092	3.87×10^{-3}	-3.496	0.126	2.78×10^{-4}
I00-I99	Mobility	Enhanced	non-SRTI	35-39	N	LG	4.96×10^{-3}	4.43×10^{-5}	0.109	3.40×10^{-3}	-3.984	0.113	1.68×10^{-4}
I00-I99	Mobility	Enhanced	non-SRTI	40-44	Y	LG	6.03×10^{-3}	8.90×10^{-5}	0.092	4.04×10^{-3}	-3.339	0.127	2.87×10^{-4}
I00-I99	Mobility	Enhanced	non-SRTI	40-44	N	LG	5.96×10^{-3}	5.09×10^{-5}	0.116	3.88×10^{-3}	-3.907	0.122	2.18×10^{-4}
I00-I99	Mobility	Enhanced	non-SRTI	45-49	Y	LG	5.13×10^{-3}	6.12×10^{-5}	0.097	3.78×10^{-3}	-3.443	0.118	2.17×10^{-4}
I00-I99	Mobility	Enhanced	non-SRTI	45-49	N	LG	6.00×10^{-3}	6.70×10^{-5}	0.099	3.63×10^{-3}	-3.477	0.114	2.41×10^{-4}
I00-I99	Mobility	Enhanced	non-SRTI	50-54	Y	LG	6.06×10^{-3}	8.36×10^{-5}	0.089	3.46×10^{-3}	-3.326	0.11	2.52×10^{-4}
I00-I99	Mobility	Enhanced	non-SRTI	50-54	N	LG	8.29×10^{-3}	1.10×10^{-4}	0.092	3.68×10^{-3}	-3.406	0.117	3.55×10^{-4}
I00-I99	Mobility	Enhanced	non-SRTI	55-59	Y	LG	5.18×10^{-3}	8.30×10^{-5}	0.084	3.45×10^{-3}	-3.287	0.111	2.21×10^{-4}
I00-I99	Mobility	Enhanced	non-SRTI	55-59	N	LG	7.95×10^{-3}	9.83×10^{-5}	0.092	3.40×10^{-3}	-3.416	0.108	3.14×10^{-4}
I00-I99	Mobility	Enhanced	non-SRTI	60-64	Y	LG	7.11×10^{-3}	6.66×10^{-5}	0.092	2.31×10^{-3}	-3.807	0.08	1.84×10^{-4}
I00-I99	Mobility	Enhanced	non-SRTI	60-64	N	LG	0.013	1.23×10^{-4}	0.094	2.51×10^{-3}	-3.676	0.084	3.73×10^{-4}
I00-I99	Mobility	Enhanced	non-SRTI	65-69	Y	LG	4.33×10^{-3}	3.16×10^{-5}	0.124	2.65×10^{-3}	-6.042	0.117	8.80×10^{-5}
I00-I99	Mobility	Enhanced	non-SRTI	65-69	N	LG	8.92×10^{-3}	4.81×10^{-5}	0.128	2.12×10^{-3}	-6.148	0.093	1.42×10^{-4}
I00-I99	Mobility	Enhanced	non-SRTI	70+	Y	LG	4.35×10^{-4}	7.75×10^{-6}	0.249	8.27×10^{-3}	-17.787	0.553	7.65×10^{-6}
I00-I99	Mobility	Enhanced	non-SRTI	70+	N	LG	9.79×10^{-4}	1.86×10^{-5}	0.247	9.04×10^{-3}	-17.589	0.602	1.94×10^{-5}
I00-I99	Daily living	Standard	SRTI	16-17	Y	No model - fewer than 3 periods with positive claims							
I00-I99	Daily living	Standard	SRTI	16-17	N	No model - fewer than 3 periods with positive claims							
I00-I99	Daily living	Standard	SRTI	18-19	Y	No model - fewer than 3 periods with positive claims							
I00-I99	Daily living	Standard	SRTI	18-19	N	No model - fewer than 3 periods with positive claims							
I00-I99	Daily living	Standard	SRTI	20-24	Y	No model - fewer than 3 periods with positive claims							
I00-I99	Daily living	Standard	SRTI	20-24	N	No model - fewer than 3 periods with positive claims							
I00-I99	Daily living	Standard	SRTI	25-29	Y	No model - fewer than 3 periods with positive claims							
I00-I99	Daily living	Standard	SRTI	25-29	N	No model - fewer than 3 periods with positive claims							
I00-I99	Daily living	Standard	SRTI	30-34	Y	No model - fewer than 3 periods with positive claims							
I00-I99	Daily living	Standard	SRTI	30-34	N	LM	1.42×10^{-4}	—	-0.412	0.022	38.472	1.74	0.192
I00-I99	Daily living	Standard	SRTI	35-39	Y	No model - fewer than 3 periods with positive claims							
I00-I99	Daily living	Standard	SRTI	35-39	N	LG	3.57×10^{-5}	6.64×10^{-6}	0.292	0.258	-16.712	14.546	2.37×10^{-5}
I00-I99	Daily living	Standard	SRTI	40-44	Y	LM	6.83×10^{-5}	—	0.052	8.43×10^{-4}	0.597	0.04	0.029
I00-I99	Daily living	Standard	SRTI	40-44	N	LM	1.27×10^{-4}	—	9.93×10^{-3}	0.021	-0.134	1.057	1.064
I00-I99	Daily living	Standard	SRTI	45-49	Y	LG	4.17×10^{-5}	3.60×10^{-6}	0.238	0.154	-5.09	3.297	2.43×10^{-5}
I00-I99	Daily living	Standard	SRTI	45-49	N	LG	6.77×10^{-5}	6.31×10^{-6}	0.118	0.03	-5.815	1.312	1.62×10^{-5}
I00-I99	Daily living	Standard	SRTI	50-54	Y	LG	6.02×10^{-5}	6.93×10^{-6}	0.088	0.025	-3.805	0.872	1.69×10^{-5}
I00-I99	Daily living	Standard	SRTI	50-54	N	LG	1.04×10^{-4}	3.13×10^{-6}	0.228	0.045	-7.274	1.407	1.84×10^{-5}
I00-I99	Daily living	Standard	SRTI	55-59	Y	LG	4.58×10^{-5}	1.98×10^{-6}	0.358	0.117	-14.699	4.785	1.11×10^{-5}
I00-I99	Daily living	Standard	SRTI	55-59	N	LG	1.03×10^{-4}	2.97×10^{-6}	0.103	0.012	-2.804	0.306	1.34×10^{-5}
I00-I99	Daily living	Standard	SRTI	60-64	Y	LG	8.54×10^{-5}	2.24×10^{-6}	0.374	0.08	-14.247	3.017	1.32×10^{-5}
I00-I99	Daily living	Standard	SRTI	60-64	N	LG	1.64×10^{-4}	3.17×10^{-6}	0.145	0.015	-3.773	0.372	1.77×10^{-5}
I00-I99	Daily living	Standard	SRTI	65-69	Y	LG	3.96×10^{-5}	1.49×10^{-6}	0.238	0.052	-9.748	2.111	7.59×10^{-6}
I00-I99	Daily living	Standard	SRTI	65-69	N	LG	8.11×10^{-5}	2.40×10^{-6}	0.136	0.015	-5.6	0.597	9.40×10^{-6}
I00-I99	Daily living	Standard	SRTI	70+	Y	LM	4.56×10^{-6}	—	1.571	0.911	-119.51	70.577	2.036
I00-I99	Daily living	Standard	SRTI	70+	N	LG	1.18×10^{-5}	3.81×10^{-7}	0.313	0.03	-21.476	2.023	6.97×10^{-7}
I00-I99	Daily living	Standard	non-SRTI	16-17	Y	LG	3.46×10^{-3}	1.17×10^{-4}	0.435	0.152	-10.341	3.606	8.27×10^{-4}
I00-I99	Daily living	Standard	non-SRTI	16-17	N	LG	4.14×10^{-3}	1.45×10^{-4}	0.127	0.018	-4.398	0.594	6.40×10^{-4}

Continued on next page

Table 1 – continued from previous page

ICD-10 code	Component	Level	Rules	Ages	Female	Model type	A	s.e.	r	s.e.	k	s.e	RSE
I00-I99	Daily living	Standard	non-SRTI	18-19	Y	LG	3.34×10^{-3}	1.14×10^{-4}	0.121	0.016	-4.189	0.528	4.89×10^{-4}
I00-I99	Daily living	Standard	non-SRTI	18-19	N	LG	4.18×10^{-3}	4.51×10^{-4}	0.069	7.53×10^{-3}	-4.245	0.262	2.99×10^{-4}
I00-I99	Daily living	Standard	non-SRTI	20-24	Y	LG	7.20×10^{-3}	4.76×10^{-4}	0.058	5.07×10^{-3}	-2.891	0.138	4.88×10^{-4}
I00-I99	Daily living	Standard	non-SRTI	20-24	N	LG	3.55×10^{-3}	2.08×10^{-4}	0.066	5.76×10^{-3}	-3.339	0.182	2.55×10^{-4}
I00-I99	Daily living	Standard	non-SRTI	25-29	Y	LG	5.33×10^{-3}	1.74×10^{-4}	0.079	5.42×10^{-3}	-3.546	0.185	3.50×10^{-4}
I00-I99	Daily living	Standard	non-SRTI	25-29	N	LG	5.42×10^{-3}	1.67×10^{-3}	0.04	6.29×10^{-3}	-2.953	0.211	3.33×10^{-4}
I00-I99	Daily living	Standard	non-SRTI	30-34	Y	LG	6.46×10^{-3}	1.86×10^{-4}	0.072	4.53×10^{-3}	-2.914	0.136	3.96×10^{-4}
I00-I99	Daily living	Standard	non-SRTI	30-34	N	LG	3.87×10^{-3}	1.01×10^{-4}	0.078	4.48×10^{-3}	-3.257	0.145	2.21×10^{-4}
I00-I99	Daily living	Standard	non-SRTI	35-39	Y	LG	5.03×10^{-3}	1.30×10^{-4}	0.08	5.29×10^{-3}	-2.984	0.16	3.47×10^{-4}
I00-I99	Daily living	Standard	non-SRTI	35-39	N	LG	3.71×10^{-3}	9.10×10^{-5}	0.078	4.75×10^{-3}	-2.991	0.144	2.31×10^{-4}
I00-I99	Daily living	Standard	non-SRTI	40-44	Y	LG	5.09×10^{-3}	8.88×10^{-5}	0.085	4.02×10^{-3}	-3.159	0.125	2.55×10^{-4}
I00-I99	Daily living	Standard	non-SRTI	40-44	N	LG	4.55×10^{-3}	6.38×10^{-5}	0.088	3.68×10^{-3}	-3.068	0.11	2.06×10^{-4}
I00-I99	Daily living	Standard	non-SRTI	45-49	Y	LG	4.56×10^{-3}	1.16×10^{-4}	0.071	3.78×10^{-3}	-2.905	0.113	2.34×10^{-4}
I00-I99	Daily living	Standard	non-SRTI	45-49	N	LG	4.50×10^{-3}	7.21×10^{-5}	0.087	4.32×10^{-3}	-2.938	0.125	2.41×10^{-4}
I00-I99	Daily living	Standard	non-SRTI	50-54	Y	LG	4.91×10^{-3}	7.97×10^{-5}	0.08	3.23×10^{-3}	-3.127	0.101	2.03×10^{-4}
I00-I99	Daily living	Standard	non-SRTI	50-54	N	LG	6.42×10^{-3}	1.59×10^{-4}	0.07	3.54×10^{-3}	-2.857	0.104	3.12×10^{-4}
I00-I99	Daily living	Standard	non-SRTI	55-59	Y	LG	4.17×10^{-3}	7.98×10^{-5}	0.076	3.39×10^{-3}	-3.049	0.104	1.86×10^{-4}
I00-I99	Daily living	Standard	non-SRTI	55-59	N	LG	5.80×10^{-3}	9.04×10^{-5}	0.081	3.27×10^{-3}	-3.087	0.101	2.43×10^{-4}
I00-I99	Daily living	Standard	non-SRTI	60-64	Y	LG	5.79×10^{-3}	8.95×10^{-5}	0.077	2.46×10^{-3}	-3.435	0.082	1.77×10^{-4}
I00-I99	Daily living	Standard	non-SRTI	60-64	N	LG	0.01	1.34×10^{-4}	0.081	2.51×10^{-3}	-3.37	0.082	3.16×10^{-4}
I00-I99	Daily living	Standard	non-SRTI	65-69	Y	LG	3.68×10^{-3}	2.15×10^{-5}	0.13	2.36×10^{-3}	-6.197	0.103	6.45×10^{-5}
I00-I99	Daily living	Standard	non-SRTI	65-69	N	LG	6.94×10^{-3}	4.35×10^{-5}	0.126	2.40×10^{-3}	-6.012	0.104	1.28×10^{-4}
I00-I99	Daily living	Standard	non-SRTI	70+	Y	LG	3.93×10^{-4}	7.57×10^{-6}	0.256	0.01	-18.095	0.685	8.75×10^{-6}
I00-I99	Daily living	Standard	non-SRTI	70+	N	LG	8.48×10^{-4}	1.44×10^{-5}	0.258	9.05×10^{-3}	-18.261	0.605	1.64×10^{-5}
I00-I99	Mobility	Standard	non-SRTI	16-17	Y	LG	1.73×10^{-3}	1.65×10^{-4}	0.116	0.044	-3.711	1.329	7.33×10^{-4}
I00-I99	Mobility	Standard	non-SRTI	16-17	N	LM	2.80×10^{-3}	—	-0.02	6.81×10^{-3}	1.254	0.36	0.846
I00-I99	Mobility	Standard	non-SRTI	18-19	Y	LM	2.64×10^{-3}	—	3.98×10^{-3}	4.23×10^{-3}	0.081	0.231	0.596
I00-I99	Mobility	Standard	non-SRTI	18-19	N	LG	1.10×10^{-3}	6.37×10^{-5}	0.322	0.157	-8.969	4.353	4.19×10^{-4}
I00-I99	Mobility	Standard	non-SRTI	20-24	Y	LG	2.85×10^{-3}	5.09×10^{-5}	0.166	0.016	-4.792	0.462	2.87×10^{-4}
I00-I99	Mobility	Standard	non-SRTI	20-24	N	LG	1.71×10^{-3}	3.72×10^{-4}	0.049	0.011	-2.704	0.251	2.41×10^{-4}
I00-I99	Mobility	Standard	non-SRTI	25-29	Y	LG	2.83×10^{-3}	4.76×10^{-5}	0.213	0.025	-4.936	0.571	3.09×10^{-4}
I00-I99	Mobility	Standard	non-SRTI	25-29	N	LG	1.56×10^{-3}	4.10×10^{-5}	0.114	0.013	-3.17	0.341	1.97×10^{-4}
I00-I99	Mobility	Standard	non-SRTI	30-34	Y	LG	3.82×10^{-3}	4.53×10^{-5}	0.159	0.01	-4.076	0.264	2.63×10^{-4}
I00-I99	Mobility	Standard	non-SRTI	30-34	N	LG	2.42×10^{-3}	2.74×10^{-5}	0.282	0.026	-6.07	0.568	1.89×10^{-4}
I00-I99	Mobility	Standard	non-SRTI	35-39	Y	LG	3.49×10^{-3}	3.92×10^{-5}	0.108	4.69×10^{-3}	-3.413	0.137	1.68×10^{-4}
I00-I99	Mobility	Standard	non-SRTI	35-39	N	LG	2.80×10^{-3}	3.12×10^{-5}	0.142	8.17×10^{-3}	-3.66	0.206	1.74×10^{-4}
I00-I99	Mobility	Standard	non-SRTI	40-44	Y	LG	3.11×10^{-3}	3.49×10^{-5}	0.139	7.80×10^{-3}	-3.661	0.201	1.90×10^{-4}
I00-I99	Mobility	Standard	non-SRTI	40-44	N	LG	3.52×10^{-3}	2.90×10^{-5}	0.15	6.57×10^{-3}	-3.868	0.167	1.64×10^{-4}
I00-I99	Mobility	Standard	non-SRTI	45-49	Y	LG	2.91×10^{-3}	2.66×10^{-5}	0.139	6.34×10^{-3}	-3.812	0.169	1.43×10^{-4}
I00-I99	Mobility	Standard	non-SRTI	45-49	N	LG	3.59×10^{-3}	3.50×10^{-5}	0.126	5.75×10^{-3}	-3.421	0.15	1.80×10^{-4}
I00-I99	Mobility	Standard	non-SRTI	50-54	Y	LG	3.30×10^{-3}	2.97×10^{-5}	0.125	5.10×10^{-3}	-3.558	0.139	1.48×10^{-4}
I00-I99	Mobility	Standard	non-SRTI	50-54	N	LG	4.72×10^{-3}	4.32×10^{-5}	0.132	5.86×10^{-3}	-3.561	0.153	2.28×10^{-4}
I00-I99	Mobility	Standard	non-SRTI	55-59	Y	LG	2.78×10^{-3}	3.37×10^{-5}	0.109	5.19×10^{-3}	-3.311	0.147	1.48×10^{-4}
I00-I99	Mobility	Standard	non-SRTI	55-59	N	LG	4.55×10^{-3}	4.24×10^{-5}	0.125	5.28×10^{-3}	-3.523	0.143	2.12×10^{-4}
I00-I99	Mobility	Standard	non-SRTI	60-64	Y	LG	3.83×10^{-3}	3.61×10^{-5}	0.101	3.19×10^{-3}	-3.529	0.1	1.33×10^{-4}
I00-I99	Mobility	Standard	non-SRTI	60-64	N	LG	7.32×10^{-3}	5.22×10^{-5}	0.12	3.66×10^{-3}	-3.589	0.104	2.47×10^{-4}
I00-I99	Mobility	Standard	non-SRTI	65-69	Y	LG	2.25×10^{-3}	1.16×10^{-5}	0.129	2.18×10^{-3}	-5.932	0.092	3.75×10^{-5}
I00-I99	Mobility	Standard	non-SRTI	65-69	N	LG	5.29×10^{-3}	1.94×10^{-5}	0.126	1.45×10^{-3}	-5.834	0.062	6.00×10^{-5}
I00-I99	Mobility	Standard	non-SRTI	70+	Y	LG	2.51×10^{-4}	5.70×10^{-6}	0.236	9.39×10^{-3}	-16.932	0.624	5.22×10^{-6}

Continued on next page

Table 1 – continued from previous page

ICD-10 code	Component	Level	Rules	Ages	Female	Model type	A	s.e.	r	s.e.	k	s.e	RSE
I00-I99	Mobility	Standard	non-SRTI	70+	N	LG	5.68×10^{-4}	1.26×10^{-5}	0.244	0.01	-17.322	0.678	1.28×10^{-5}
J00-J99	Daily living	Enhanced	SRTI	16-17	Y	No model - fewer than 3 periods with positive claims							
J00-J99	Daily living	Enhanced	SRTI	16-17	N	No model - fewer than 3 periods with positive claims							
J00-J99	Daily living	Enhanced	SRTI	18-19	Y	No model - fewer than 3 periods with positive claims							
J00-J99	Daily living	Enhanced	SRTI	18-19	N	No model - fewer than 3 periods with positive claims							
J00-J99	Daily living	Enhanced	SRTI	20-24	Y	No model - fewer than 3 periods with positive claims							
J00-J99	Daily living	Enhanced	SRTI	20-24	N	No model - fewer than 3 periods with positive claims							
J00-J99	Daily living	Enhanced	SRTI	25-29	Y	No model - fewer than 3 periods with positive claims							
J00-J99	Daily living	Enhanced	SRTI	25-29	N	No model - fewer than 3 periods with positive claims							
J00-J99	Daily living	Enhanced	SRTI	30-34	Y	No model - fewer than 3 periods with positive claims							
J00-J99	Daily living	Enhanced	SRTI	30-34	N	No model - fewer than 3 periods with positive claims							
J00-J99	Daily living	Enhanced	SRTI	35-39	Y	No model - fewer than 3 periods with positive claims							
J00-J99	Daily living	Enhanced	SRTI	35-39	N	No model - fewer than 3 periods with positive claims							
J00-J99	Daily living	Enhanced	SRTI	40-44	Y	LM	6.40×10^{-5}	—	-8.49×10^{-3}	0.011	1.605	0.604	0.654
J00-J99	Daily living	Enhanced	SRTI	40-44	N	LM	1.37×10^{-4}	—	0.026	0.011	-1.803	0.753	0.684
J00-J99	Daily living	Enhanced	SRTI	45-49	Y	LM	2.86×10^{-4}	—	0.038	5.64×10^{-3}	-3.67	0.348	0.437
J00-J99	Daily living	Enhanced	SRTI	45-49	N	LM	3.04×10^{-4}	—	0.024	5.33×10^{-3}	-2.344	0.302	0.667
J00-J99	Daily living	Enhanced	SRTI	50-54	Y	LG	2.14×10^{-4}	7.65×10^{-6}	0.155	0.026	-5.558	0.903	3.69×10^{-5}
J00-J99	Daily living	Enhanced	SRTI	50-54	N	LG	2.04×10^{-4}	8.91×10^{-6}	0.117	0.023	-3.082	0.582	4.46×10^{-5}
J00-J99	Daily living	Enhanced	SRTI	55-59	Y	LG	3.37×10^{-4}	7.50×10^{-6}	0.15	0.016	-4.956	0.509	3.75×10^{-5}
J00-J99	Daily living	Enhanced	SRTI	55-59	N	LG	4.26×10^{-4}	1.08×10^{-5}	0.087	7.32×10^{-3}	-2.636	0.195	3.91×10^{-5}
J00-J99	Daily living	Enhanced	SRTI	60-64	Y	LG	9.52×10^{-4}	3.99×10^{-5}	0.069	4.72×10^{-3}	-3.312	0.151	5.68×10^{-5}
J00-J99	Daily living	Enhanced	SRTI	60-64	N	LG	1.10×10^{-3}	2.24×10^{-5}	0.085	5.20×10^{-3}	-2.901	0.15	7.20×10^{-5}
J00-J99	Daily living	Enhanced	SRTI	65-69	Y	LG	8.41×10^{-4}	3.00×10^{-5}	0.085	4.90×10^{-3}	-4.663	0.198	3.96×10^{-5}
J00-J99	Daily living	Enhanced	SRTI	65-69	N	LG	8.93×10^{-4}	1.31×10^{-5}	0.109	4.26×10^{-3}	-5.202	0.178	3.37×10^{-5}
J00-J99	Daily living	Enhanced	SRTI	70+	Y	LG	8.70×10^{-5}	1.83×10^{-6}	0.309	0.016	-21.998	1.072	2.42×10^{-6}
J00-J99	Daily living	Enhanced	SRTI	70+	N	LG	9.82×10^{-5}	1.75×10^{-6}	0.368	0.023	-25.29	1.54	3.66×10^{-6}
J00-J99	Daily living	Enhanced	non-SRTI	16-17	Y	LM	1.01×10^{-3}	—	5.81×10^{-3}	2.67×10^{-3}	-0.676	0.142	0.403
J00-J99	Daily living	Enhanced	non-SRTI	16-17	N	LG	3.99×10^{-4}	3.42×10^{-5}	-0.166	0.477	14.709	37.807	2.57×10^{-4}
J00-J99	Daily living	Enhanced	non-SRTI	18-19	Y	LM	8.78×10^{-4}	—	0.018	3.92×10^{-3}	-1.278	0.212	0.565
J00-J99	Daily living	Enhanced	non-SRTI	18-19	N	LG	5.28×10^{-4}	1.84×10^{-5}	0.223	0.054	-5.536	1.339	1.19×10^{-4}
J00-J99	Daily living	Enhanced	non-SRTI	20-24	Y	LG	7.27×10^{-4}	3.00×10^{-5}	0.071	5.93×10^{-3}	-2.99	0.18	5.79×10^{-5}
J00-J99	Daily living	Enhanced	non-SRTI	20-24	N	LG	1.09×10^{-3}	7.22×10^{-5}	0.059	3.85×10^{-3}	-3.493	0.111	4.67×10^{-5}
J00-J99	Daily living	Enhanced	non-SRTI	25-29	Y	LG	6.09×10^{-4}	1.86×10^{-5}	0.086	7.86×10^{-3}	-2.922	0.228	6.01×10^{-5}
J00-J99	Daily living	Enhanced	non-SRTI	25-29	N	LG	3.96×10^{-4}	1.72×10^{-5}	0.074	6.09×10^{-3}	-3.354	0.2	3.04×10^{-5}
J00-J99	Daily living	Enhanced	non-SRTI	30-34	Y	LG	1.08×10^{-3}	1.67×10^{-5}	0.133	0.01	-3.569	0.263	8.91×10^{-5}
J00-J99	Daily living	Enhanced	non-SRTI	30-34	N	LG	4.92×10^{-4}	1.11×10^{-5}	0.121	0.012	-3.553	0.332	5.35×10^{-5}
J00-J99	Daily living	Enhanced	non-SRTI	35-39	Y	LG	1.50×10^{-3}	3.21×10^{-5}	0.086	5.81×10^{-3}	-2.725	0.16	1.11×10^{-4}
J00-J99	Daily living	Enhanced	non-SRTI	35-39	N	LG	5.88×10^{-4}	9.25×10^{-6}	0.156	0.014	-3.899	0.335	5.39×10^{-5}
J00-J99	Daily living	Enhanced	non-SRTI	40-44	Y	LG	3.34×10^{-3}	3.04×10^{-5}	0.149	7.19×10^{-3}	-3.892	0.184	1.71×10^{-4}
J00-J99	Daily living	Enhanced	non-SRTI	40-44	N	LG	1.70×10^{-3}	2.34×10^{-5}	0.166	0.014	-3.873	0.313	1.43×10^{-4}
J00-J99	Daily living	Enhanced	non-SRTI	45-49	Y	LG	6.08×10^{-3}	8.07×10^{-5}	0.092	3.85×10^{-3}	-3.154	0.116	2.78×10^{-4}
J00-J99	Daily living	Enhanced	non-SRTI	45-49	N	LG	3.36×10^{-3}	5.28×10^{-5}	0.102	5.93×10^{-3}	-3.155	0.168	2.18×10^{-4}
J00-J99	Daily living	Enhanced	non-SRTI	50-54	Y	LG	0.012	1.36×10^{-4}	0.096	3.61×10^{-3}	-3.338	0.111	4.81×10^{-4}
J00-J99	Daily living	Enhanced	non-SRTI	50-54	N	LG	7.35×10^{-3}	9.50×10^{-5}	0.095	3.85×10^{-3}	-3.359	0.12	3.25×10^{-4}
J00-J99	Daily living	Enhanced	non-SRTI	55-59	Y	LG	0.014	2.01×10^{-4}	0.083	2.99×10^{-3}	-3.289	0.096	5.30×10^{-4}
J00-J99	Daily living	Enhanced	non-SRTI	55-59	N	LG	9.21×10^{-3}	1.07×10^{-4}	0.092	3.30×10^{-3}	-3.325	0.103	3.55×10^{-4}
J00-J99	Daily living	Enhanced	non-SRTI	60-64	Y	LG	0.025	2.10×10^{-4}	0.096	2.19×10^{-3}	-4.063	0.079	5.78×10^{-4}
J00-J99	Daily living	Enhanced	non-SRTI	60-64	N	LG	0.02	2.03×10^{-4}	0.095	2.89×10^{-3}	-3.722	0.098	6.30×10^{-4}
J00-J99	Daily living	Enhanced	non-SRTI	65-69	Y	LG	0.021	5.76×10^{-5}	0.156	1.60×10^{-3}	-7.438	0.072	2.03×10^{-4}

Continued on next page

Table 1 – continued from previous page

ICD-10 code	Component	Level	Rules	Ages	Female	Model type	A	s.e.	r	s.e.	k	s.e	RSE
J00-J99	Daily living	Enhanced	non-SRTI	65-69	N	LG	0.019	7.89×10^{-5}	0.149	2.23×10^{-3}	-7.09	0.1	2.70×10^{-4}
J00-J99	Daily living	Enhanced	non-SRTI	70+	Y	LG	3.15×10^{-3}	5.93×10^{-5}	0.252	9.28×10^{-3}	-17.932	0.62	6.28×10^{-5}
J00-J99	Daily living	Enhanced	non-SRTI	70+	N	LG	3.07×10^{-3}	6.43×10^{-5}	0.246	9.86×10^{-3}	-17.528	0.656	6.62×10^{-5}
J00-J99	Mobility	Enhanced	non-SRTI	16-17	Y	LG	3.43×10^{-4}	9.93×10^{-6}	0.151	0.021	-5.004	0.673	4.98×10^{-5}
J00-J99	Mobility	Enhanced	non-SRTI	16-17	N	LG	3.12×10^{-4}	7.92×10^{-6}	0.308	0.066	-7.393	1.592	5.40×10^{-5}
J00-J99	Mobility	Enhanced	non-SRTI	18-19	Y	LG	4.04×10^{-4}	1.61×10^{-5}	0.086	8.89×10^{-3}	-3.431	0.292	4.33×10^{-5}
J00-J99	Mobility	Enhanced	non-SRTI	18-19	N	LG	3.02×10^{-4}	6.92×10^{-6}	0.209	0.028	-7.492	0.984	3.74×10^{-5}
J00-J99	Mobility	Enhanced	non-SRTI	20-24	Y	LG	5.59×10^{-4}	1.24×10^{-5}	0.1	7.57×10^{-3}	-3.437	0.233	4.64×10^{-5}
J00-J99	Mobility	Enhanced	non-SRTI	20-24	N	LG	3.68×10^{-4}	2.91×10^{-5}	0.065	7.70×10^{-3}	-3.253	0.239	3.60×10^{-5}
J00-J99	Mobility	Enhanced	non-SRTI	25-29	Y	LG	8.25×10^{-4}	1.79×10^{-5}	0.115	0.011	-3.362	0.292	8.42×10^{-5}
J00-J99	Mobility	Enhanced	non-SRTI	25-29	N	LG	3.24×10^{-4}	5.37×10^{-6}	0.14	0.01	-4.896	0.341	2.50×10^{-5}
J00-J99	Mobility	Enhanced	non-SRTI	30-34	Y	LG	1.95×10^{-3}	4.34×10^{-5}	0.087	5.53×10^{-3}	-3.133	0.169	1.33×10^{-4}
J00-J99	Mobility	Enhanced	non-SRTI	30-34	N	LG	1.12×10^{-3}	1.17×10^{-4}	0.052	5.50×10^{-3}	-2.865	0.134	7.88×10^{-5}
J00-J99	Mobility	Enhanced	non-SRTI	35-39	Y	LG	2.74×10^{-3}	7.25×10^{-5}	0.078	5.01×10^{-3}	-2.992	0.152	1.80×10^{-4}
J00-J99	Mobility	Enhanced	non-SRTI	35-39	N	LG	1.35×10^{-3}	2.20×10^{-5}	0.092	4.60×10^{-3}	-3.229	0.141	7.37×10^{-5}
J00-J99	Mobility	Enhanced	non-SRTI	40-44	Y	LG	6.47×10^{-3}	6.21×10^{-5}	0.108	3.90×10^{-3}	-3.475	0.116	2.60×10^{-4}
J00-J99	Mobility	Enhanced	non-SRTI	40-44	N	LG	3.80×10^{-3}	3.54×10^{-5}	0.11	3.71×10^{-3}	-3.793	0.118	1.41×10^{-4}
J00-J99	Mobility	Enhanced	non-SRTI	45-49	Y	LG	0.012	1.19×10^{-4}	0.1	3.33×10^{-3}	-3.622	0.107	4.18×10^{-4}
J00-J99	Mobility	Enhanced	non-SRTI	45-49	N	LG	6.66×10^{-3}	7.02×10^{-5}	0.1	3.54×10^{-3}	-3.492	0.111	2.58×10^{-4}
J00-J99	Mobility	Enhanced	non-SRTI	50-54	Y	LG	0.023	2.85×10^{-4}	0.091	3.19×10^{-3}	-3.462	0.104	8.63×10^{-4}
J00-J99	Mobility	Enhanced	non-SRTI	50-54	N	LG	0.017	1.87×10^{-4}	0.09	2.71×10^{-3}	-3.616	0.091	5.27×10^{-4}
J00-J99	Mobility	Enhanced	non-SRTI	55-59	Y	LG	0.028	2.79×10^{-4}	0.095	2.75×10^{-3}	-3.711	0.093	8.57×10^{-4}
J00-J99	Mobility	Enhanced	non-SRTI	55-59	N	LG	0.021	2.13×10^{-4}	0.096	2.99×10^{-3}	-3.66	0.099	6.88×10^{-4}
J00-J99	Mobility	Enhanced	non-SRTI	60-64	Y	LG	0.052	4.61×10^{-4}	0.092	2.07×10^{-3}	-3.882	0.073	1.21×10^{-3}
J00-J99	Mobility	Enhanced	non-SRTI	60-64	N	LG	0.046	3.38×10^{-4}	0.099	2.21×10^{-3}	-3.902	0.076	1.08×10^{-3}
J00-J99	Mobility	Enhanced	non-SRTI	65-69	Y	LG	0.037	2.37×10^{-4}	0.13	2.55×10^{-3}	-6.334	0.114	6.85×10^{-4}
J00-J99	Mobility	Enhanced	non-SRTI	65-69	N	LG	0.037	1.71×10^{-4}	0.136	2.04×10^{-3}	-6.521	0.09	5.35×10^{-4}
J00-J99	Mobility	Enhanced	non-SRTI	70+	Y	LG	4.12×10^{-3}	9.94×10^{-5}	0.238	0.01	-17.012	0.684	9.42×10^{-5}
J00-J99	Mobility	Enhanced	non-SRTI	70+	N	LG	4.85×10^{-3}	1.16×10^{-4}	0.24	0.011	-17.058	0.709	1.16×10^{-4}
J00-J99	Daily living	Standard	SRTI	16-17	Y	No model - fewer than 3 periods with positive claims							
J00-J99	Daily living	Standard	SRTI	16-17	N	No model - fewer than 3 periods with positive claims							
J00-J99	Daily living	Standard	SRTI	18-19	Y	No model - fewer than 3 periods with positive claims							
J00-J99	Daily living	Standard	SRTI	18-19	N	No model - fewer than 3 periods with positive claims							
J00-J99	Daily living	Standard	SRTI	20-24	Y	No model - fewer than 3 periods with positive claims							
J00-J99	Daily living	Standard	SRTI	20-24	N	No model - fewer than 3 periods with positive claims							
J00-J99	Daily living	Standard	SRTI	25-29	Y	No model - fewer than 3 periods with positive claims							
J00-J99	Daily living	Standard	SRTI	25-29	N	No model - fewer than 3 periods with positive claims							
J00-J99	Daily living	Standard	SRTI	30-34	Y	No model - fewer than 3 periods with positive claims							
J00-J99	Daily living	Standard	SRTI	30-34	N	No model - fewer than 3 periods with positive claims							
J00-J99	Daily living	Standard	SRTI	35-39	Y	No model - fewer than 3 periods with positive claims							
J00-J99	Daily living	Standard	SRTI	35-39	N	No model - fewer than 3 periods with positive claims							
J00-J99	Daily living	Standard	SRTI	40-44	Y	LM	6.40×10^{-5}	—	-8.49×10^{-3}	0.011	1.605	0.604	0.654
J00-J99	Daily living	Standard	SRTI	40-44	N	LM	1.37×10^{-4}	—	0.026	0.011	-1.803	0.753	0.684
J00-J99	Daily living	Standard	SRTI	45-49	Y	LM	2.86×10^{-4}	—	0.038	5.64×10^{-3}	-3.67	0.348	0.437
J00-J99	Daily living	Standard	SRTI	45-49	N	LM	3.04×10^{-4}	—	0.024	5.33×10^{-3}	-2.344	0.302	0.667
J00-J99	Daily living	Standard	SRTI	50-54	Y	LG	2.14×10^{-4}	7.65×10^{-6}	0.155	0.026	-5.558	0.903	3.69×10^{-5}
J00-J99	Daily living	Standard	SRTI	50-54	N	LG	2.30×10^{-4}	1.08×10^{-5}	0.098	0.017	-2.899	0.465	4.48×10^{-5}
J00-J99	Daily living	Standard	SRTI	55-59	Y	LG	3.37×10^{-4}	7.50×10^{-6}	0.15	0.016	-4.956	0.509	3.75×10^{-5}
J00-J99	Daily living	Standard	SRTI	55-59	N	LG	4.25×10^{-4}	1.04×10^{-5}	0.091	7.73×10^{-3}	-2.735	0.208	3.97×10^{-5}
J00-J99	Daily living	Standard	SRTI	60-64	Y	LG	1.01×10^{-3}	4.66×10^{-5}	0.066	4.52×10^{-3}	-3.315	0.142	5.73×10^{-5}

Continued on next page

Table 1 – continued from previous page

ICD-10 code	Component	Level	Rules	Ages	Female	Model type	A	s.e.	r	s.e.	k	s.e	RSE
J00-J99	Daily living	Standard	SRTI	60-64	N	LG	1.11×10^{-3}	2.31×10^{-5}	0.084	5.18×10^{-3}	-2.897	0.15	7.28×10^{-5}
J00-J99	Daily living	Standard	SRTI	65-69	Y	LG	6.80×10^{-4}	2.06×10^{-5}	0.09	5.17×10^{-3}	-4.656	0.21	3.44×10^{-5}
J00-J99	Daily living	Standard	SRTI	65-69	N	LG	7.40×10^{-4}	1.03×10^{-5}	0.118	5.08×10^{-3}	-5.345	0.207	3.17×10^{-5}
J00-J99	Daily living	Standard	SRTI	70+	Y	LG	6.43×10^{-5}	2.39×10^{-6}	0.278	0.022	-19.85	1.495	2.75×10^{-6}
J00-J99	Daily living	Standard	SRTI	70+	N	LG	7.25×10^{-5}	1.44×10^{-6}	0.444	0.038	-30.182	2.549	3.66×10^{-6}
J00-J99	Daily living	Standard	non-SRTI	16-17	Y	LG	4.38×10^{-4}	1.24×10^{-5}	0.139	0.02	-3.798	0.519	6.67×10^{-5}
J00-J99	Daily living	Standard	non-SRTI	16-17	N	LM	6.63×10^{-4}	—	8.63×10^{-3}	3.52×10^{-3}	-0.462	0.189	0.52
J00-J99	Daily living	Standard	non-SRTI	18-19	Y	LG	4.07×10^{-4}	1.28×10^{-5}	0.133	0.018	-4.685	0.592	5.78×10^{-5}
J00-J99	Daily living	Standard	non-SRTI	18-19	N	LG	3.16×10^{-4}	7.96×10^{-6}	0.207	0.033	-6.171	0.961	4.71×10^{-5}
J00-J99	Daily living	Standard	non-SRTI	20-24	Y	LG	6.88×10^{-4}	1.90×10^{-5}	0.08	5.73×10^{-3}	-2.988	0.173	5.11×10^{-5}
J00-J99	Daily living	Standard	non-SRTI	20-24	N	LG	5.68×10^{-4}	2.80×10^{-5}	0.082	6.14×10^{-3}	-4.476	0.242	3.46×10^{-5}
J00-J99	Daily living	Standard	non-SRTI	25-29	Y	LG	1.08×10^{-3}	5.50×10^{-5}	0.063	5.65×10^{-3}	-2.785	0.16	8.48×10^{-5}
J00-J99	Daily living	Standard	non-SRTI	25-29	N	LG	4.09×10^{-4}	2.06×10^{-5}	0.076	6.86×10^{-3}	-3.703	0.24	3.31×10^{-5}
J00-J99	Daily living	Standard	non-SRTI	30-34	Y	LG	1.96×10^{-3}	5.00×10^{-5}	0.081	5.41×10^{-3}	-3.054	0.165	1.36×10^{-4}
J00-J99	Daily living	Standard	non-SRTI	30-34	N	LG	7.35×10^{-4}	3.60×10^{-5}	0.069	7.27×10^{-3}	-2.706	0.207	7.47×10^{-5}
J00-J99	Daily living	Standard	non-SRTI	35-39	Y	LG	2.52×10^{-3}	6.83×10^{-5}	0.071	4.37×10^{-3}	-2.738	0.126	1.53×10^{-4}
J00-J99	Daily living	Standard	non-SRTI	35-39	N	LG	1.28×10^{-3}	3.35×10^{-5}	0.071	4.00×10^{-3}	-2.883	0.119	6.95×10^{-5}
J00-J99	Daily living	Standard	non-SRTI	40-44	Y	LG	5.88×10^{-3}	7.45×10^{-5}	0.096	4.13×10^{-3}	-3.198	0.122	2.77×10^{-4}
J00-J99	Daily living	Standard	non-SRTI	40-44	N	LG	3.43×10^{-3}	4.25×10^{-5}	0.096	3.88×10^{-3}	-3.281	0.118	1.52×10^{-4}
J00-J99	Daily living	Standard	non-SRTI	45-49	Y	LG	0.01	1.21×10^{-4}	0.088	2.81×10^{-3}	-3.374	0.091	3.53×10^{-4}
J00-J99	Daily living	Standard	non-SRTI	45-49	N	LG	6.16×10^{-3}	8.60×10^{-5}	0.085	3.25×10^{-3}	-3.158	0.101	2.49×10^{-4}
J00-J99	Daily living	Standard	non-SRTI	50-54	Y	LG	0.021	2.82×10^{-4}	0.084	2.85×10^{-3}	-3.297	0.091	7.50×10^{-4}
J00-J99	Daily living	Standard	non-SRTI	50-54	N	LG	0.014	1.64×10^{-4}	0.086	2.45×10^{-3}	-3.49	0.082	4.26×10^{-4}
J00-J99	Daily living	Standard	non-SRTI	55-59	Y	LG	0.026	3.23×10^{-4}	0.083	2.42×10^{-3}	-3.478	0.081	7.75×10^{-4}
J00-J99	Daily living	Standard	non-SRTI	55-59	N	LG	0.018	2.22×10^{-4}	0.085	2.61×10^{-3}	-3.443	0.086	5.80×10^{-4}
J00-J99	Daily living	Standard	non-SRTI	60-64	Y	LG	0.049	4.43×10^{-4}	0.088	1.89×10^{-3}	-3.919	0.068	1.03×10^{-3}
J00-J99	Daily living	Standard	non-SRTI	60-64	N	LG	0.04	3.62×10^{-4}	0.092	2.23×10^{-3}	-3.825	0.078	9.96×10^{-4}
J00-J99	Daily living	Standard	non-SRTI	65-69	Y	LG	0.036	1.44×10^{-4}	0.147	2.03×10^{-3}	-7.045	0.091	4.78×10^{-4}
J00-J99	Daily living	Standard	non-SRTI	65-69	N	LG	0.035	1.40×10^{-4}	0.147	2.09×10^{-3}	-6.969	0.093	4.74×10^{-4}
J00-J99	Daily living	Standard	non-SRTI	70+	Y	LG	4.81×10^{-3}	1.05×10^{-4}	0.245	0.01	-17.488	0.674	1.06×10^{-4}
J00-J99	Daily living	Standard	non-SRTI	70+	N	LG	5.26×10^{-3}	1.17×10^{-4}	0.244	0.01	-17.308	0.687	1.21×10^{-4}
J00-J99	Mobility	Standard	non-SRTI	16-17	Y	LM	4.11×10^{-4}	—	-8.09×10^{-3}	9.13×10^{-3}	1.244	0.491	1.349
J00-J99	Mobility	Standard	non-SRTI	16-17	N	LM	6.16×10^{-4}	—	-0.012	4.01×10^{-3}	0.641	0.215	0.592
J00-J99	Mobility	Standard	non-SRTI	18-19	Y	LG	3.32×10^{-4}	6.44×10^{-6}	0.169	0.018	-4.936	0.517	3.62×10^{-5}
J00-J99	Mobility	Standard	non-SRTI	18-19	N	LG	3.39×10^{-4}	9.94×10^{-6}	0.203	0.038	-5.508	1.022	6.08×10^{-5}
J00-J99	Mobility	Standard	non-SRTI	20-24	Y	LG	4.67×10^{-4}	8.69×10^{-6}	0.187	0.021	-5.164	0.571	5.16×10^{-5}
J00-J99	Mobility	Standard	non-SRTI	20-24	N	LG	5.69×10^{-4}	3.16×10^{-5}	0.065	6.00×10^{-3}	-2.996	0.179	4.59×10^{-5}
J00-J99	Mobility	Standard	non-SRTI	25-29	Y	LG	6.08×10^{-4}	1.08×10^{-5}	0.123	9.85×10^{-3}	-3.415	0.263	5.41×10^{-5}
J00-J99	Mobility	Standard	non-SRTI	25-29	N	LG	3.37×10^{-4}	6.95×10^{-6}	0.129	0.011	-4.443	0.362	3.12×10^{-5}
J00-J99	Mobility	Standard	non-SRTI	30-34	Y	LG	1.04×10^{-3}	1.44×10^{-5}	0.155	0.012	-3.821	0.289	8.39×10^{-5}
J00-J99	Mobility	Standard	non-SRTI	30-34	N	LG	5.96×10^{-4}	2.45×10^{-5}	0.072	6.96×10^{-3}	-2.75	0.201	5.70×10^{-5}
J00-J99	Mobility	Standard	non-SRTI	35-39	Y	LG	1.51×10^{-3}	1.86×10^{-5}	0.181	0.013	-4.634	0.342	1.13×10^{-4}
J00-J99	Mobility	Standard	non-SRTI	35-39	N	LG	7.45×10^{-4}	8.09×10^{-6}	0.173	0.011	-4.125	0.267	4.95×10^{-5}
J00-J99	Mobility	Standard	non-SRTI	40-44	Y	LG	3.93×10^{-3}	3.43×10^{-5}	0.179	9.50×10^{-3}	-4.358	0.23	2.10×10^{-4}
J00-J99	Mobility	Standard	non-SRTI	40-44	N	LG	2.32×10^{-3}	1.43×10^{-5}	0.176	6.42×10^{-3}	-4.492	0.163	8.59×10^{-5}
J00-J99	Mobility	Standard	non-SRTI	45-49	Y	LG	6.78×10^{-3}	4.77×10^{-5}	0.143	5.09×10^{-3}	-3.956	0.137	2.59×10^{-4}
J00-J99	Mobility	Standard	non-SRTI	45-49	N	LG	3.88×10^{-3}	2.60×10^{-5}	0.168	6.53×10^{-3}	-4.192	0.161	1.55×10^{-4}
J00-J99	Mobility	Standard	non-SRTI	50-54	Y	LG	0.013	9.86×10^{-5}	0.134	4.87×10^{-3}	-3.806	0.134	5.10×10^{-4}
J00-J99	Mobility	Standard	non-SRTI	50-54	N	LG	9.09×10^{-3}	6.48×10^{-5}	0.146	5.29×10^{-3}	-4.021	0.143	3.54×10^{-4}

Continued on next page

Table 1 – continued from previous page

ICD-10 code	Component	Level	Rules	Ages	Female	Model type	A	s.e.	r	s.e.	k	s.e	RSE
J00-J99	Mobility	Standard	non-SRTI	55-59	Y	LG	0.015	1.08×10^{-4}	0.125	3.94×10^{-3}	-3.731	0.112	5.23×10^{-4}
J00-J99	Mobility	Standard	non-SRTI	55-59	N	LG	0.012	9.78×10^{-5}	0.135	5.34×10^{-3}	-3.827	0.146	5.10×10^{-4}
J00-J99	Mobility	Standard	non-SRTI	60-64	Y	LG	0.027	1.87×10^{-4}	0.117	3.26×10^{-3}	-3.866	0.101	8.19×10^{-4}
J00-J99	Mobility	Standard	non-SRTI	60-64	N	LG	0.025	1.85×10^{-4}	0.127	4.17×10^{-3}	-3.811	0.119	9.04×10^{-4}
J00-J99	Mobility	Standard	non-SRTI	65-69	Y	LG	0.02	1.14×10^{-4}	0.127	2.30×10^{-3}	-5.918	0.098	3.51×10^{-4}
J00-J99	Mobility	Standard	non-SRTI	65-69	N	LG	0.021	7.02×10^{-5}	0.127	1.36×10^{-3}	-5.813	0.057	2.24×10^{-4}
J00-J99	Mobility	Standard	non-SRTI	70+	Y	LG	2.26×10^{-3}	4.91×10^{-5}	0.243	9.99×10^{-3}	-17.32	0.664	4.99×10^{-5}
J00-J99	Mobility	Standard	non-SRTI	70+	N	LG	2.52×10^{-3}	6.03×10^{-5}	0.243	0.011	-17.268	0.735	6.21×10^{-5}
K00-K93	Daily living	Enhanced	SRTI	16-17	Y	No model - fewer than 3 periods with positive claims							
K00-K93	Daily living	Enhanced	SRTI	16-17	N	No model - fewer than 3 periods with positive claims							
K00-K93	Daily living	Enhanced	SRTI	18-19	Y	No model - fewer than 3 periods with positive claims							
K00-K93	Daily living	Enhanced	SRTI	18-19	N	No model - fewer than 3 periods with positive claims							
K00-K93	Daily living	Enhanced	SRTI	20-24	Y	No model - fewer than 3 periods with positive claims							
K00-K93	Daily living	Enhanced	SRTI	20-24	N	No model - fewer than 3 periods with positive claims							
K00-K93	Daily living	Enhanced	SRTI	25-29	Y	No model - fewer than 3 periods with positive claims							
K00-K93	Daily living	Enhanced	SRTI	25-29	N	No model - fewer than 3 periods with positive claims							
K00-K93	Daily living	Enhanced	SRTI	30-34	Y	LM	1.02×10^{-4}	—	9.14×10^{-3}	0.02	1.907	1.179	1.539
K00-K93	Daily living	Enhanced	SRTI	30-34	N	No model - fewer than 3 periods with positive claims							
K00-K93	Daily living	Enhanced	SRTI	35-39	Y	LM	8.69×10^{-5}	—	-0.019	0.016	3.101	1.009	1.024
K00-K93	Daily living	Enhanced	SRTI	35-39	N	LM	1.75×10^{-4}	—	2.53×10^{-3}	0.016	0.298	0.935	1.686
K00-K93	Daily living	Enhanced	SRTI	40-44	Y	LM	2.75×10^{-4}	—	5.41×10^{-3}	4.45×10^{-3}	-0.846	0.244	0.585
K00-K93	Daily living	Enhanced	SRTI	40-44	N	LM	4.19×10^{-4}	—	8.36×10^{-3}	6.90×10^{-3}	-0.619	0.376	0.914
K00-K93	Daily living	Enhanced	SRTI	45-49	Y	LG	7.65×10^{-5}	5.19×10^{-6}	0.196	0.09	-3.978	1.837	3.42×10^{-5}
K00-K93	Daily living	Enhanced	SRTI	45-49	N	LG	2.12×10^{-4}	7.85×10^{-6}	0.212	0.052	-5.591	1.361	4.90×10^{-5}
K00-K93	Daily living	Enhanced	SRTI	50-54	Y	LG	2.14×10^{-4}	1.04×10^{-5}	0.146	0.031	-5.572	1.123	4.64×10^{-5}
K00-K93	Daily living	Enhanced	SRTI	50-54	N	LG	4.11×10^{-4}	1.07×10^{-5}	0.167	0.025	-4.219	0.629	6.38×10^{-5}
K00-K93	Daily living	Enhanced	SRTI	55-59	Y	LG	1.91×10^{-4}	9.93×10^{-5}	0.036	0.015	-2.117	0.399	3.66×10^{-5}
K00-K93	Daily living	Enhanced	SRTI	55-59	N	LG	3.22×10^{-4}	6.27×10^{-6}	0.15	0.016	-3.622	0.381	3.65×10^{-5}
K00-K93	Daily living	Enhanced	SRTI	60-64	Y	LG	2.03×10^{-4}	1.07×10^{-5}	0.111	0.021	-4.009	0.698	4.20×10^{-5}
K00-K93	Daily living	Enhanced	SRTI	60-64	N	LG	4.72×10^{-4}	1.46×10^{-5}	0.113	0.015	-3.135	0.395	6.97×10^{-5}
K00-K93	Daily living	Enhanced	SRTI	65-69	Y	LM	1.69×10^{-4}	—	0.042	0.016	-3.498	1.094	1.014
K00-K93	Daily living	Enhanced	SRTI	65-69	N	LG	1.20×10^{-4}	9.39×10^{-6}	0.127	0.032	-5.878	1.341	2.94×10^{-5}
K00-K93	Daily living	Enhanced	SRTI	70+	Y	No model - fewer than 3 periods with positive claims							
K00-K93	Daily living	Enhanced	SRTI	70+	N	LM	1.92×10^{-5}	—	-0.258	0.142	21.6	10.892	0.922
K00-K93	Daily living	Enhanced	non-SRTI	16-17	Y	LG	3.64×10^{-3}	1.46×10^{-4}	0.092	0.012	-3.027	0.348	5.22×10^{-4}
K00-K93	Daily living	Enhanced	non-SRTI	16-17	N	LM	6.06×10^{-3}	—	-4.61×10^{-3}	4.00×10^{-3}	0.387	0.215	0.591
K00-K93	Daily living	Enhanced	non-SRTI	18-19	Y	LG	2.08×10^{-3}	4.14×10^{-5}	0.29	0.047	-6.939	1.131	2.81×10^{-4}
K00-K93	Daily living	Enhanced	non-SRTI	18-19	N	LG	2.54×10^{-3}	5.90×10^{-5}	0.24	0.038	-7.09	1.127	3.62×10^{-4}
K00-K93	Daily living	Enhanced	non-SRTI	20-24	Y	LG	2.91×10^{-3}	3.67×10^{-5}	0.177	0.014	-4.106	0.317	2.28×10^{-4}
K00-K93	Daily living	Enhanced	non-SRTI	20-24	N	LG	2.94×10^{-3}	4.47×10^{-5}	0.14	0.011	-3.878	0.285	2.40×10^{-4}
K00-K93	Daily living	Enhanced	non-SRTI	25-29	Y	LG	2.91×10^{-3}	5.05×10^{-5}	0.108	7.27×10^{-3}	-3.353	0.209	2.18×10^{-4}
K00-K93	Daily living	Enhanced	non-SRTI	25-29	N	LG	2.07×10^{-3}	4.54×10^{-5}	0.112	0.01	-3.341	0.28	2.06×10^{-4}
K00-K93	Daily living	Enhanced	non-SRTI	30-34	Y	LG	3.90×10^{-3}	3.24×10^{-5}	0.178	8.94×10^{-3}	-4.428	0.22	1.97×10^{-4}
K00-K93	Daily living	Enhanced	non-SRTI	30-34	N	LG	3.44×10^{-3}	7.93×10^{-5}	0.08	5.15×10^{-3}	-2.818	0.148	2.32×10^{-4}
K00-K93	Daily living	Enhanced	non-SRTI	35-39	Y	LG	3.91×10^{-3}	5.17×10^{-5}	0.112	6.19×10^{-3}	-3.215	0.168	2.41×10^{-4}
K00-K93	Daily living	Enhanced	non-SRTI	35-39	N	LG	2.76×10^{-3}	3.25×10^{-5}	0.156	0.01	-3.971	0.254	1.88×10^{-4}
K00-K93	Daily living	Enhanced	non-SRTI	40-44	Y	LG	5.02×10^{-3}	7.99×10^{-5}	0.12	8.61×10^{-3}	-3.203	0.222	4.01×10^{-4}
K00-K93	Daily living	Enhanced	non-SRTI	40-44	N	LG	4.13×10^{-3}	5.07×10^{-5}	0.132	7.81×10^{-3}	-3.534	0.203	2.68×10^{-4}
K00-K93	Daily living	Enhanced	non-SRTI	45-49	Y	LG	4.51×10^{-3}	4.39×10^{-5}	0.123	5.37×10^{-3}	-3.533	0.147	2.17×10^{-4}
K00-K93	Daily living	Enhanced	non-SRTI	45-49	N	LG	3.55×10^{-3}	4.09×10^{-5}	0.138	8.08×10^{-3}	-3.588	0.205	2.24×10^{-4}

Continued on next page

Table 1 – continued from previous page

ICD-10 code	Component	Level	Rules	Ages	Female	Model type	A	s.e.	r	s.e.	k	s.e	RSE
K00-K93	Daily living	Enhanced	non-SRTI	50-54	Y	LG	5.39×10^{-3}	9.25×10^{-5}	0.086	4.33×10^{-3}	-3.012	0.129	2.91×10^{-4}
K00-K93	Daily living	Enhanced	non-SRTI	50-54	N	LG	4.79×10^{-3}	4.20×10^{-5}	0.15	7.11×10^{-3}	-3.712	0.174	2.42×10^{-4}
K00-K93	Daily living	Enhanced	non-SRTI	55-59	Y	LG	3.52×10^{-3}	6.28×10^{-5}	0.089	4.97×10^{-3}	-2.979	0.144	2.14×10^{-4}
K00-K93	Daily living	Enhanced	non-SRTI	55-59	N	LG	3.61×10^{-3}	5.80×10^{-5}	0.093	5.02×10^{-3}	-2.968	0.143	2.15×10^{-4}
K00-K93	Daily living	Enhanced	non-SRTI	60-64	Y	LG	3.89×10^{-3}	6.54×10^{-5}	0.084	3.60×10^{-3}	-3.302	0.116	1.73×10^{-4}
K00-K93	Daily living	Enhanced	non-SRTI	60-64	N	LG	3.61×10^{-3}	4.48×10^{-5}	0.105	4.71×10^{-3}	-3.538	0.144	1.78×10^{-4}
K00-K93	Daily living	Enhanced	non-SRTI	65-69	Y	LG	2.34×10^{-3}	3.72×10^{-5}	0.09	2.52×10^{-3}	-4.826	0.105	5.55×10^{-5}
K00-K93	Daily living	Enhanced	non-SRTI	65-69	N	LG	2.41×10^{-3}	3.63×10^{-5}	0.095	2.79×10^{-3}	-5.02	0.118	6.15×10^{-5}
K00-K93	Daily living	Enhanced	non-SRTI	70+	Y	LG	2.47×10^{-4}	8.77×10^{-6}	0.209	9.79×10^{-3}	-15.31	0.64	5.37×10^{-6}
K00-K93	Daily living	Enhanced	non-SRTI	70+	N	LG	1.76×10^{-4}	3.79×10^{-6}	0.307	0.017	-21.647	1.141	5.47×10^{-6}
K00-K93	Mobility	Enhanced	non-SRTI	16-17	Y	LG	3.32×10^{-3}	1.12×10^{-4}	0.142	0.023	-4.165	0.663	5.85×10^{-4}
K00-K93	Mobility	Enhanced	non-SRTI	16-17	N	LG	4.64×10^{-3}	9.86×10^{-5}	0.158	0.018	-4.648	0.506	5.39×10^{-4}
K00-K93	Mobility	Enhanced	non-SRTI	18-19	Y	LG	3.04×10^{-3}	6.79×10^{-5}	0.093	6.84×10^{-3}	-3.118	0.201	2.44×10^{-4}
K00-K93	Mobility	Enhanced	non-SRTI	18-19	N	LG	2.81×10^{-3}	9.59×10^{-5}	0.103	0.012	-3.687	0.383	3.55×10^{-4}
K00-K93	Mobility	Enhanced	non-SRTI	20-24	Y	LG	4.27×10^{-3}	8.98×10^{-5}	0.13	0.013	-3.521	0.34	4.69×10^{-4}
K00-K93	Mobility	Enhanced	non-SRTI	20-24	N	LG	3.69×10^{-3}	8.61×10^{-5}	0.094	6.92×10^{-3}	-3.3	0.213	2.96×10^{-4}
K00-K93	Mobility	Enhanced	non-SRTI	25-29	Y	LG	5.25×10^{-3}	1.08×10^{-4}	0.085	5.06×10^{-3}	-3.005	0.151	3.34×10^{-4}
K00-K93	Mobility	Enhanced	non-SRTI	25-29	N	LG	3.79×10^{-3}	7.07×10^{-5}	0.085	4.11×10^{-3}	-3.378	0.134	1.90×10^{-4}
K00-K93	Mobility	Enhanced	non-SRTI	30-34	Y	LG	9.18×10^{-3}	3.13×10^{-4}	0.069	4.53×10^{-3}	-2.976	0.137	5.61×10^{-4}
K00-K93	Mobility	Enhanced	non-SRTI	30-34	N	LG	4.84×10^{-3}	8.99×10^{-5}	0.101	6.30×10^{-3}	-3.515	0.197	3.32×10^{-4}
K00-K93	Mobility	Enhanced	non-SRTI	35-39	Y	LG	7.14×10^{-3}	1.38×10^{-4}	0.083	4.18×10^{-3}	-3.206	0.132	3.73×10^{-4}
K00-K93	Mobility	Enhanced	non-SRTI	35-39	N	LG	4.79×10^{-3}	1.01×10^{-4}	0.081	4.70×10^{-3}	-2.925	0.139	2.91×10^{-4}
K00-K93	Mobility	Enhanced	non-SRTI	40-44	Y	LG	9.58×10^{-3}	2.05×10^{-4}	0.079	4.41×10^{-3}	-2.95	0.132	5.52×10^{-4}
K00-K93	Mobility	Enhanced	non-SRTI	40-44	N	LG	7.04×10^{-3}	1.28×10^{-4}	0.085	4.27×10^{-3}	-3.119	0.131	3.76×10^{-4}
K00-K93	Mobility	Enhanced	non-SRTI	45-49	Y	LG	8.87×10^{-3}	1.95×10^{-4}	0.076	3.82×10^{-3}	-3.086	0.119	4.42×10^{-4}
K00-K93	Mobility	Enhanced	non-SRTI	45-49	N	LG	6.62×10^{-3}	1.19×10^{-4}	0.078	3.20×10^{-3}	-3.206	0.102	2.71×10^{-4}
K00-K93	Mobility	Enhanced	non-SRTI	50-54	Y	LG	9.84×10^{-3}	2.03×10^{-4}	0.081	4.17×10^{-3}	-3.133	0.13	5.23×10^{-4}
K00-K93	Mobility	Enhanced	non-SRTI	50-54	N	LG	8.85×10^{-3}	1.33×10^{-4}	0.087	3.52×10^{-3}	-3.356	0.114	3.77×10^{-4}
K00-K93	Mobility	Enhanced	non-SRTI	55-59	Y	LG	7.37×10^{-3}	2.13×10^{-4}	0.07	3.90×10^{-3}	-3.067	0.12	3.82×10^{-4}
K00-K93	Mobility	Enhanced	non-SRTI	55-59	N	LG	6.18×10^{-3}	1.13×10^{-4}	0.082	3.67×10^{-3}	-3.348	0.12	2.80×10^{-4}
K00-K93	Mobility	Enhanced	non-SRTI	60-64	Y	LG	8.26×10^{-3}	2.27×10^{-4}	0.071	3.07×10^{-3}	-3.53	0.103	3.07×10^{-4}
K00-K93	Mobility	Enhanced	non-SRTI	60-64	N	LG	6.99×10^{-3}	1.58×10^{-4}	0.078	4.14×10^{-3}	-3.172	0.131	3.71×10^{-4}
K00-K93	Mobility	Enhanced	non-SRTI	65-69	Y	LG	3.60×10^{-3}	4.02×10^{-5}	0.099	2.30×10^{-3}	-5.176	0.099	7.42×10^{-5}
K00-K93	Mobility	Enhanced	non-SRTI	65-69	N	LG	4.06×10^{-3}	2.83×10^{-5}	0.109	1.77×10^{-3}	-5.583	0.078	6.01×10^{-5}
K00-K93	Mobility	Enhanced	non-SRTI	70+	Y	LG	2.62×10^{-4}	7.47×10^{-6}	0.236	0.012	-16.806	0.815	7.32×10^{-6}
K00-K93	Mobility	Enhanced	non-SRTI	70+	N	LG	3.44×10^{-4}	1.04×10^{-5}	0.249	0.012	-18.126	0.839	8.42×10^{-6}
K00-K93	Daily living	Standard	SRTI	16-17	Y	No model - fewer than 3 periods with positive claims							
K00-K93	Daily living	Standard	SRTI	16-17	N	No model - fewer than 3 periods with positive claims							
K00-K93	Daily living	Standard	SRTI	18-19	Y	No model - fewer than 3 periods with positive claims							
K00-K93	Daily living	Standard	SRTI	18-19	N	No model - fewer than 3 periods with positive claims							
K00-K93	Daily living	Standard	SRTI	20-24	Y	No model - fewer than 3 periods with positive claims							
K00-K93	Daily living	Standard	SRTI	20-24	N	No model - fewer than 3 periods with positive claims							
K00-K93	Daily living	Standard	SRTI	25-29	Y	No model - fewer than 3 periods with positive claims							
K00-K93	Daily living	Standard	SRTI	25-29	N	No model - fewer than 3 periods with positive claims							
K00-K93	Daily living	Standard	SRTI	30-34	Y	LM	1.02×10^{-4}	—	9.14×10^{-3}	0.02	1.907	1.179	1.539
K00-K93	Daily living	Standard	SRTI	30-34	N	No model - fewer than 3 periods with positive claims							
K00-K93	Daily living	Standard	SRTI	35-39	Y	LM	8.69×10^{-5}	—	-0.019	0.016	3.101	1.009	1.024
K00-K93	Daily living	Standard	SRTI	35-39	N	LM	1.75×10^{-4}	—	2.53×10^{-3}	0.016	0.298	0.935	1.686
K00-K93	Daily living	Standard	SRTI	40-44	Y	LM	2.75×10^{-4}	—	5.41×10^{-3}	4.45×10^{-3}	-0.846	0.244	0.585
K00-K93	Daily living	Standard	SRTI	40-44	N	LM	4.19×10^{-4}	—	8.36×10^{-3}	6.90×10^{-3}	-0.619	0.376	0.914

Continued on next page

Table 1 – continued from previous page

ICD-10 code	Component	Level	Rules	Ages	Female	Model type	A	s.e.	r	s.e.	k	s.e	RSE
K00-K93	Daily living	Standard	SRTI	45-49	Y	LM	1.61×10^{-4}	—	0.011	5.87×10^{-3}	-0.681	0.308	0.939
K00-K93	Daily living	Standard	SRTI	45-49	N	LG	2.12×10^{-4}	7.85×10^{-6}	0.212	0.052	-5.591	1.361	4.90×10^{-5}
K00-K93	Daily living	Standard	SRTI	50-54	Y	LG	2.16×10^{-4}	1.05×10^{-5}	0.142	0.029	-5.439	1.061	4.57×10^{-5}
K00-K93	Daily living	Standard	SRTI	50-54	N	LG	4.12×10^{-4}	1.07×10^{-5}	0.167	0.025	-4.199	0.621	6.36×10^{-5}
K00-K93	Daily living	Standard	SRTI	55-59	Y	LG	1.91×10^{-4}	9.93×10^{-5}	0.036	0.015	-2.117	0.399	3.66×10^{-5}
K00-K93	Daily living	Standard	SRTI	55-59	N	LG	3.22×10^{-4}	6.27×10^{-6}	0.15	0.016	-3.622	0.381	3.65×10^{-5}
K00-K93	Daily living	Standard	SRTI	60-64	Y	LG	2.03×10^{-4}	1.07×10^{-5}	0.111	0.021	-4.009	0.698	4.20×10^{-5}
K00-K93	Daily living	Standard	SRTI	60-64	N	LG	4.80×10^{-4}	1.61×10^{-5}	0.103	0.013	-3.017	0.363	6.96×10^{-5}
K00-K93	Daily living	Standard	SRTI	65-69	Y	LM	2.22×10^{-4}	—	0.041	0.017	-3.985	1.238	0.796
K00-K93	Daily living	Standard	SRTI	65-69	N	LG	9.62×10^{-5}	4.40×10^{-6}	0.206	0.049	-8.716	2.018	2.08×10^{-5}
K00-K93	Daily living	Standard	SRTI	70+	Y	No model - fewer than 3 periods with positive claims							
K00-K93	Daily living	Standard	SRTI	70+	N	LM	1.97×10^{-5}	—	-0.052	1.54×10^{-3}	7.105	0.118	9.95×10^{-3}
K00-K93	Daily living	Standard	non-SRTI	16-17	Y	LG	2.06×10^{-3}	7.73×10^{-5}	0.164	0.034	-4.484	0.915	4.43×10^{-4}
K00-K93	Daily living	Standard	non-SRTI	16-17	N	LM	3.30×10^{-3}	—	3.84×10^{-3}	7.45×10^{-3}	0.287	0.395	1.152
K00-K93	Daily living	Standard	non-SRTI	18-19	Y	LG	1.81×10^{-3}	1.62×10^{-4}	0.061	8.90×10^{-3}	-2.712	0.244	2.27×10^{-4}
K00-K93	Daily living	Standard	non-SRTI	18-19	N	LG	1.34×10^{-3}	4.40×10^{-5}	0.192	0.036	-6.338	1.167	2.43×10^{-4}
K00-K93	Daily living	Standard	non-SRTI	20-24	Y	LG	2.58×10^{-3}	2.05×10^{-4}	0.053	6.47×10^{-3}	-2.309	0.152	2.46×10^{-4}
K00-K93	Daily living	Standard	non-SRTI	20-24	N	LG	1.41×10^{-3}	2.28×10^{-5}	0.221	0.025	-5.379	0.6	1.47×10^{-4}
K00-K93	Daily living	Standard	non-SRTI	25-29	Y	LG	2.43×10^{-3}	6.29×10^{-5}	0.076	4.77×10^{-3}	-2.94	0.143	1.54×10^{-4}
K00-K93	Daily living	Standard	non-SRTI	25-29	N	LG	2.54×10^{-3}	2.19×10^{-4}	0.054	4.70×10^{-3}	-3.027	0.12	1.48×10^{-4}
K00-K93	Daily living	Standard	non-SRTI	30-34	Y	LG	3.39×10^{-3}	5.06×10^{-5}	0.12	7.70×10^{-3}	-3.506	0.215	2.42×10^{-4}
K00-K93	Daily living	Standard	non-SRTI	30-34	N	LG	3.42×10^{-3}	1.32×10^{-4}	0.064	3.78×10^{-3}	-3.131	0.114	1.68×10^{-4}
K00-K93	Daily living	Standard	non-SRTI	35-39	Y	LG	4.02×10^{-3}	1.13×10^{-4}	0.071	4.39×10^{-3}	-2.761	0.127	2.44×10^{-4}
K00-K93	Daily living	Standard	non-SRTI	35-39	N	LG	2.19×10^{-3}	6.39×10^{-5}	0.083	7.13×10^{-3}	-2.802	0.203	2.01×10^{-4}
K00-K93	Daily living	Standard	non-SRTI	40-44	Y	LG	4.73×10^{-3}	1.11×10^{-4}	0.086	6.29×10^{-3}	-2.808	0.177	3.75×10^{-4}
K00-K93	Daily living	Standard	non-SRTI	40-44	N	LG	3.91×10^{-3}	1.12×10^{-4}	0.076	5.49×10^{-3}	-2.778	0.159	2.90×10^{-4}
K00-K93	Daily living	Standard	non-SRTI	45-49	Y	LG	5.07×10^{-3}	1.27×10^{-4}	0.075	4.37×10^{-3}	-2.905	0.13	2.97×10^{-4}
K00-K93	Daily living	Standard	non-SRTI	45-49	N	LG	4.24×10^{-3}	1.13×10^{-4}	0.069	3.50×10^{-3}	-3.01	0.107	1.99×10^{-4}
K00-K93	Daily living	Standard	non-SRTI	50-54	Y	LG	6.75×10^{-3}	1.51×10^{-4}	0.071	2.93×10^{-3}	-3.188	0.093	2.57×10^{-4}
K00-K93	Daily living	Standard	non-SRTI	50-54	N	LG	5.04×10^{-3}	9.02×10^{-5}	0.084	4.32×10^{-3}	-2.91	0.126	2.77×10^{-4}
K00-K93	Daily living	Standard	non-SRTI	55-59	Y	LG	4.49×10^{-3}	1.22×10^{-4}	0.071	3.85×10^{-3}	-2.994	0.117	2.32×10^{-4}
K00-K93	Daily living	Standard	non-SRTI	55-59	N	LG	4.07×10^{-3}	9.81×10^{-5}	0.074	4.14×10^{-3}	-2.856	0.122	2.28×10^{-4}
K00-K93	Daily living	Standard	non-SRTI	60-64	Y	LG	5.38×10^{-3}	1.60×10^{-4}	0.066	2.92×10^{-3}	-3.339	0.093	1.96×10^{-4}
K00-K93	Daily living	Standard	non-SRTI	60-64	N	LG	4.27×10^{-3}	8.64×10^{-5}	0.086	4.91×10^{-3}	-3.14	0.151	2.59×10^{-4}
K00-K93	Daily living	Standard	non-SRTI	65-69	Y	LG	2.73×10^{-3}	4.08×10^{-5}	0.095	2.72×10^{-3}	-5.008	0.115	6.80×10^{-5}
K00-K93	Daily living	Standard	non-SRTI	65-69	N	LG	2.85×10^{-3}	3.23×10^{-5}	0.111	2.97×10^{-3}	-5.736	0.133	6.91×10^{-5}
K00-K93	Daily living	Standard	non-SRTI	70+	Y	LG	2.59×10^{-4}	1.18×10^{-5}	0.203	0.011	-14.949	0.744	6.57×10^{-6}
K00-K93	Daily living	Standard	non-SRTI	70+	N	LG	2.36×10^{-4}	5.18×10^{-6}	0.262	0.012	-18.608	0.802	5.91×10^{-6}
K00-K93	Mobility	Standard	non-SRTI	16-17	Y	LG	2.13×10^{-3}	1.77×10^{-4}	0.077	0.014	-3.16	0.453	3.95×10^{-4}
K00-K93	Mobility	Standard	non-SRTI	16-17	N	LG	2.44×10^{-3}	6.84×10^{-5}	0.254	0.053	-6.434	1.345	4.47×10^{-4}
K00-K93	Mobility	Standard	non-SRTI	18-19	Y	LM	4.33×10^{-3}	—	-6.86×10^{-3}	2.63×10^{-3}	-0.379	0.142	0.38
K00-K93	Mobility	Standard	non-SRTI	18-19	N	LG	1.36×10^{-3}	2.85×10^{-5}	0.335	0.063	-7.761	1.46	1.98×10^{-4}
K00-K93	Mobility	Standard	non-SRTI	20-24	Y	LG	2.25×10^{-3}	3.30×10^{-5}	0.162	0.014	-3.974	0.329	1.96×10^{-4}
K00-K93	Mobility	Standard	non-SRTI	20-24	N	LG	2.08×10^{-3}	4.72×10^{-5}	0.148	0.017	-4.119	0.47	2.59×10^{-4}
K00-K93	Mobility	Standard	non-SRTI	25-29	Y	LG	2.17×10^{-3}	2.73×10^{-5}	0.169	0.012	-4.242	0.306	1.63×10^{-4}
K00-K93	Mobility	Standard	non-SRTI	25-29	N	LG	1.72×10^{-3}	3.59×10^{-5}	0.13	0.012	-3.893	0.351	1.78×10^{-4}
K00-K93	Mobility	Standard	non-SRTI	30-34	Y	LG	3.54×10^{-3}	5.01×10^{-5}	0.113	6.43×10^{-3}	-3.532	0.188	2.23×10^{-4}
K00-K93	Mobility	Standard	non-SRTI	30-34	N	LG	2.41×10^{-3}	3.25×10^{-5}	0.129	7.47×10^{-3}	-4.193	0.232	1.52×10^{-4}
K00-K93	Mobility	Standard	non-SRTI	35-39	Y	LG	3.09×10^{-3}	3.90×10^{-5}	0.111	5.63×10^{-3}	-3.303	0.157	1.75×10^{-4}

Continued on next page

Table 1 – continued from previous page

ICD-10 code	Component	Level	Rules	Ages	Female	Model type	A	s.e.	r	s.e.	k	s.e	RSE
K00-K93	Mobility	Standard	non-SRTI	35-39	N	LG	2.27×10^{-3}	2.47×10^{-5}	0.139	7.58×10^{-3}	-3.724	0.198	1.34×10^{-4}
K00-K93	Mobility	Standard	non-SRTI	40-44	Y	LG	4.84×10^{-3}	1.05×10^{-4}	0.088	5.95×10^{-3}	-2.913	0.171	3.56×10^{-4}
K00-K93	Mobility	Standard	non-SRTI	40-44	N	LG	3.43×10^{-3}	4.23×10^{-5}	0.119	6.17×10^{-3}	-3.627	0.178	1.97×10^{-4}
K00-K93	Mobility	Standard	non-SRTI	45-49	Y	LG	4.19×10^{-3}	5.31×10^{-5}	0.101	4.56×10^{-3}	-3.25	0.134	2.10×10^{-4}
K00-K93	Mobility	Standard	non-SRTI	45-49	N	LG	2.93×10^{-3}	3.04×10^{-5}	0.124	5.77×10^{-3}	-3.536	0.158	1.51×10^{-4}
K00-K93	Mobility	Standard	non-SRTI	50-54	Y	LG	4.38×10^{-3}	7.11×10^{-5}	0.102	6.11×10^{-3}	-3.205	0.175	2.91×10^{-4}
K00-K93	Mobility	Standard	non-SRTI	50-54	N	LG	4.44×10^{-3}	6.45×10^{-5}	0.117	7.26×10^{-3}	-3.417	0.201	3.06×10^{-4}
K00-K93	Mobility	Standard	non-SRTI	55-59	Y	LG	3.36×10^{-3}	7.22×10^{-5}	0.081	4.41×10^{-3}	-3.127	0.137	1.89×10^{-4}
K00-K93	Mobility	Standard	non-SRTI	55-59	N	LG	3.11×10^{-3}	3.57×10^{-5}	0.106	4.44×10^{-3}	-3.564	0.136	1.43×10^{-4}
K00-K93	Mobility	Standard	non-SRTI	60-64	Y	LG	3.82×10^{-3}	6.89×10^{-5}	0.079	3.02×10^{-3}	-3.474	0.102	1.42×10^{-4}
K00-K93	Mobility	Standard	non-SRTI	60-64	N	LG	3.71×10^{-3}	5.93×10^{-5}	0.088	4.11×10^{-3}	-3.209	0.127	1.85×10^{-4}
K00-K93	Mobility	Standard	non-SRTI	65-69	Y	LG	1.67×10^{-3}	2.49×10^{-5}	0.096	3.02×10^{-3}	-4.891	0.126	4.76×10^{-5}
K00-K93	Mobility	Standard	non-SRTI	65-69	N	LG	2.10×10^{-3}	3.16×10^{-5}	0.102	3.48×10^{-3}	-5.131	0.147	6.59×10^{-5}
K00-K93	Mobility	Standard	non-SRTI	70+	Y	LG	1.45×10^{-4}	3.08×10^{-6}	0.29	0.015	-20.469	0.998	4.17×10^{-6}
K00-K93	Mobility	Standard	non-SRTI	70+	N	LG	2.19×10^{-4}	1.73×10^{-5}	0.212	0.015	-16.298	0.958	5.18×10^{-6}
L00-L99	Daily living	Enhanced	SRTI	16-17	Y	No model - fewer than 3 periods with positive claims							
L00-L99	Daily living	Enhanced	SRTI	16-17	N	No model - fewer than 3 periods with positive claims							
L00-L99	Daily living	Enhanced	SRTI	18-19	Y	No model - fewer than 3 periods with positive claims							
L00-L99	Daily living	Enhanced	SRTI	18-19	N	No model - fewer than 3 periods with positive claims							
L00-L99	Daily living	Enhanced	SRTI	20-24	Y	No model - fewer than 3 periods with positive claims							
L00-L99	Daily living	Enhanced	SRTI	20-24	N	No model - fewer than 3 periods with positive claims							
L00-L99	Daily living	Enhanced	SRTI	25-29	Y	No model - fewer than 3 periods with positive claims							
L00-L99	Daily living	Enhanced	SRTI	25-29	N	No model - fewer than 3 periods with positive claims							
L00-L99	Daily living	Enhanced	SRTI	30-34	Y	No model - fewer than 3 periods with positive claims							
L00-L99	Daily living	Enhanced	SRTI	30-34	N	No model - fewer than 3 periods with positive claims							
L00-L99	Daily living	Enhanced	SRTI	35-39	Y	No model - fewer than 3 periods with positive claims							
L00-L99	Daily living	Enhanced	SRTI	35-39	N	No model - fewer than 3 periods with positive claims							
L00-L99	Daily living	Enhanced	SRTI	40-44	Y	No model - fewer than 3 periods with positive claims							
L00-L99	Daily living	Enhanced	SRTI	40-44	N	No model - fewer than 3 periods with positive claims							
L00-L99	Daily living	Enhanced	SRTI	45-49	Y	No model - fewer than 3 periods with positive claims							
L00-L99	Daily living	Enhanced	SRTI	45-49	N	No model - fewer than 3 periods with positive claims							
L00-L99	Daily living	Enhanced	SRTI	50-54	Y	No model - fewer than 3 periods with positive claims							
L00-L99	Daily living	Enhanced	SRTI	50-54	N	No model - fewer than 3 periods with positive claims							
L00-L99	Daily living	Enhanced	SRTI	55-59	Y	No model - fewer than 3 periods with positive claims							
L00-L99	Daily living	Enhanced	SRTI	55-59	N	No model - fewer than 3 periods with positive claims							
L00-L99	Daily living	Enhanced	SRTI	60-64	Y	No model - fewer than 3 periods with positive claims							
L00-L99	Daily living	Enhanced	SRTI	60-64	N	No model - fewer than 3 periods with positive claims							
L00-L99	Daily living	Enhanced	SRTI	65-69	Y	No model - fewer than 3 periods with positive claims							
L00-L99	Daily living	Enhanced	SRTI	65-69	N	No model - fewer than 3 periods with positive claims							
L00-L99	Daily living	Enhanced	SRTI	70+	Y	No model - fewer than 3 periods with positive claims							
L00-L99	Daily living	Enhanced	SRTI	70+	N	No model - fewer than 3 periods with positive claims							
L00-L99	Daily living	Enhanced	non-SRTI	16-17	Y	LM	3.08×10^{-3}	—	-3.95×10^{-3}	3.68×10^{-3}	-0.859	0.199	0.504
L00-L99	Daily living	Enhanced	non-SRTI	16-17	N	LG	1.17×10^{-3}	1.07×10^{-4}	0.073	0.018	-2.348	0.469	2.97×10^{-4}
L00-L99	Daily living	Enhanced	non-SRTI	18-19	Y	LG	7.21×10^{-4}	3.08×10^{-5}	0.248	0.073	-7.727	2.261	1.86×10^{-4}
L00-L99	Daily living	Enhanced	non-SRTI	18-19	N	LG	7.17×10^{-4}	2.57×10^{-5}	0.33	0.088	-12.761	3.387	1.47×10^{-4}
L00-L99	Daily living	Enhanced	non-SRTI	20-24	Y	LG	1.24×10^{-3}	1.07×10^{-4}	0.069	8.64×10^{-3}	-3.626	0.29	1.25×10^{-4}
L00-L99	Daily living	Enhanced	non-SRTI	20-24	N	LG	8.84×10^{-4}	3.04×10^{-5}	0.138	0.019	-5.448	0.709	1.26×10^{-4}
L00-L99	Daily living	Enhanced	non-SRTI	25-29	Y	LG	9.31×10^{-4}	3.33×10^{-5}	0.095	0.011	-3.096	0.334	1.24×10^{-4}
L00-L99	Daily living	Enhanced	non-SRTI	25-29	N	LG	9.06×10^{-4}	2.53×10^{-5}	0.148	0.018	-5.524	0.653	1.15×10^{-4}
L00-L99	Daily living	Enhanced	non-SRTI	30-34	Y	LG	2.12×10^{-3}	1.61×10^{-4}	0.065	7.84×10^{-3}	-3.096	0.237	2.18×10^{-4}
L00-L99	Daily living	Enhanced	non-SRTI	30-34	N	LG	1.33×10^{-3}	4.45×10^{-5}	0.121	0.016	-4.099	0.518	1.96×10^{-4}
L00-L99	Daily living	Enhanced	non-SRTI	35-39	Y	LG	1.84×10^{-3}	7.78×10^{-5}	0.088	0.011	-2.979	0.331	2.57×10^{-4}

Continued on next page

Table 1 – continued from previous page

ICD-10 code	Component	Level	Rules	Ages	Female	Model type	A	s.e.	r	s.e.	k	s.e	RSE
L00-L99	Daily living	Enhanced	non-SRTI	35-39	N	LG	1.43×10^{-3}	3.47×10^{-5}	0.175	0.023	-5.821	0.738	1.85×10^{-4}
L00-L99	Daily living	Enhanced	non-SRTI	40-44	Y	LG	3.41×10^{-3}	9.06×10^{-5}	0.083	5.83×10^{-3}	-3.192	0.183	2.49×10^{-4}
L00-L99	Daily living	Enhanced	non-SRTI	40-44	N	LG	3.27×10^{-3}	2.67×10^{-4}	0.06	6.50×10^{-3}	-3	0.184	2.80×10^{-4}
L00-L99	Daily living	Enhanced	non-SRTI	45-49	Y	LG	3.12×10^{-3}	5.59×10^{-5}	0.099	5.93×10^{-3}	-3.424	0.183	2.05×10^{-4}
L00-L99	Daily living	Enhanced	non-SRTI	45-49	N	LG	2.35×10^{-3}	4.93×10^{-5}	0.125	0.011	-4.051	0.338	2.27×10^{-4}
L00-L99	Daily living	Enhanced	non-SRTI	50-54	Y	LG	4.84×10^{-3}	1.97×10^{-4}	0.067	4.72×10^{-3}	-3.092	0.145	3.02×10^{-4}
L00-L99	Daily living	Enhanced	non-SRTI	50-54	N	LG	3.11×10^{-3}	8.25×10^{-5}	0.09	6.26×10^{-3}	-3.695	0.215	2.24×10^{-4}
L00-L99	Daily living	Enhanced	non-SRTI	55-59	Y	LG	4.22×10^{-3}	1.48×10^{-4}	0.071	4.09×10^{-3}	-3.474	0.136	2.12×10^{-4}
L00-L99	Daily living	Enhanced	non-SRTI	55-59	N	LG	3.64×10^{-3}	7.15×10^{-5}	0.096	5.24×10^{-3}	-3.986	0.186	2.06×10^{-4}
L00-L99	Daily living	Enhanced	non-SRTI	60-64	Y	LG	6.48×10^{-3}	1.58×10^{-4}	0.102	5.85×10^{-3}	-5.03	0.244	3.48×10^{-4}
L00-L99	Daily living	Enhanced	non-SRTI	60-64	N	LG	5.68×10^{-3}	2.60×10^{-4}	0.071	5.11×10^{-3}	-3.589	0.173	3.46×10^{-4}
L00-L99	Daily living	Enhanced	non-SRTI	65-69	Y	LG	3.55×10^{-3}	6.40×10^{-5}	0.139	7.81×10^{-3}	-6.976	0.363	1.86×10^{-4}
L00-L99	Daily living	Enhanced	non-SRTI	65-69	N	LG	3.09×10^{-3}	7.02×10^{-5}	0.144	0.011	-7.195	0.496	2.13×10^{-4}
L00-L99	Daily living	Enhanced	non-SRTI	70+	Y	LG	5.93×10^{-4}	3.23×10^{-5}	0.235	0.019	-17.156	1.271	2.28×10^{-5}
L00-L99	Daily living	Enhanced	non-SRTI	70+	N	LG	3.72×10^{-4}	3.39×10^{-5}	0.203	0.024	-14.747	1.584	2.13×10^{-5}
L00-L99	Mobility	Enhanced	non-SRTI	16-17	Y	LM	3.3×10^{-3}	—	0.018	3.4×10^{-3}	-2.167	0.191	0.461
L00-L99	Mobility	Enhanced	non-SRTI	16-17	N	LM	1.26×10^{-3}	—	4.72×10^{-3}	8.04×10^{-3}	0.179	0.482	0.745
L00-L99	Mobility	Enhanced	non-SRTI	18-19	Y	LM	1.81×10^{-3}	—	-1.06×10^{-3}	5.42×10^{-3}	-0.734	0.311	0.593
L00-L99	Mobility	Enhanced	non-SRTI	18-19	N	LG	7.68×10^{-4}	5.15×10^{-5}	0.103	0.017	-5.035	0.691	1.17×10^{-4}
L00-L99	Mobility	Enhanced	non-SRTI	20-24	Y	LG	1.47×10^{-3}	2.63×10^{-5}	0.194	0.019	-7.193	0.683	1.35×10^{-4}
L00-L99	Mobility	Enhanced	non-SRTI	20-24	N	LG	1.02×10^{-3}	6.56×10^{-5}	0.092	0.014	-4.241	0.527	1.50×10^{-4}
L00-L99	Mobility	Enhanced	non-SRTI	25-29	Y	LG	4.07×10^{-3}	3.72×10^{-4}	0.059	4.18×10^{-3}	-3.826	0.118	1.64×10^{-4}
L00-L99	Mobility	Enhanced	non-SRTI	25-29	N	LG	8.61×10^{-4}	2.81×10^{-5}	0.188	0.031	-7.662	1.218	1.33×10^{-4}
L00-L99	Mobility	Enhanced	non-SRTI	30-34	Y	LG	3.20×10^{-3}	7.94×10^{-5}	0.112	0.011	-3.711	0.325	3.35×10^{-4}
L00-L99	Mobility	Enhanced	non-SRTI	30-34	N	LG	1.89×10^{-3}	3.61×10^{-5}	0.194	0.023	-5.216	0.613	2.19×10^{-4}
L00-L99	Mobility	Enhanced	non-SRTI	35-39	Y	LG	3.86×10^{-3}	8.72×10^{-5}	0.09	6.15×10^{-3}	-3.204	0.188	2.85×10^{-4}
L00-L99	Mobility	Enhanced	non-SRTI	35-39	N	LG	2.54×10^{-3}	3.93×10^{-5}	0.124	8.22×10^{-3}	-3.884	0.244	1.85×10^{-4}
L00-L99	Mobility	Enhanced	non-SRTI	40-44	Y	LG	6.72×10^{-3}	2.58×10^{-4}	0.069	4.77×10^{-3}	-3.075	0.147	4.26×10^{-4}
L00-L99	Mobility	Enhanced	non-SRTI	40-44	N	LG	5.74×10^{-3}	1.41×10^{-4}	0.089	5.62×10^{-3}	-3.671	0.193	3.73×10^{-4}
L00-L99	Mobility	Enhanced	non-SRTI	45-49	Y	LG	5.96×10^{-3}	1.60×10^{-4}	0.079	4.69×10^{-3}	-3.324	0.153	3.51×10^{-4}
L00-L99	Mobility	Enhanced	non-SRTI	45-49	N	LG	5.40×10^{-3}	8.15×10^{-5}	0.092	3.71×10^{-3}	-3.805	0.129	2.25×10^{-4}
L00-L99	Mobility	Enhanced	non-SRTI	50-54	Y	LG	7.93×10^{-3}	1.13×10^{-4}	0.09	3.07×10^{-3}	-3.951	0.111	2.71×10^{-4}
L00-L99	Mobility	Enhanced	non-SRTI	50-54	N	LG	7.04×10^{-3}	2.20×10^{-4}	0.077	4.55×10^{-3}	-3.561	0.156	3.89×10^{-4}
L00-L99	Mobility	Enhanced	non-SRTI	55-59	Y	LG	7.30×10^{-3}	1.47×10^{-4}	0.088	4.32×10^{-3}	-3.817	0.153	3.59×10^{-4}
L00-L99	Mobility	Enhanced	non-SRTI	55-59	N	LG	7.20×10^{-3}	1.86×10^{-4}	0.078	3.72×10^{-3}	-3.766	0.132	3.12×10^{-4}
L00-L99	Mobility	Enhanced	non-SRTI	60-64	Y	LG	0.012	2.67×10^{-4}	0.085	3.68×10^{-3}	-4.151	0.139	4.84×10^{-4}
L00-L99	Mobility	Enhanced	non-SRTI	60-64	N	LG	0.013	5.37×10^{-4}	0.066	4.12×10^{-3}	-3.298	0.129	6.58×10^{-4}
L00-L99	Mobility	Enhanced	non-SRTI	65-69	Y	LG	9.10×10^{-3}	4.71×10^{-4}	0.081	4.36×10^{-3}	-5.168	0.178	3.09×10^{-4}
L00-L99	Mobility	Enhanced	non-SRTI	65-69	N	LG	6.62×10^{-3}	1.16×10^{-4}	0.116	5.43×10^{-3}	-5.756	0.238	2.91×10^{-4}
L00-L99	Mobility	Enhanced	non-SRTI	70+	Y	LG	4.30×10^{-4}	1.22×10^{-5}	0.296	0.022	-20.599	1.486	1.89×10^{-5}
L00-L99	Mobility	Enhanced	non-SRTI	70+	N	LG	7.02×10^{-4}	1.90×10^{-5}	0.288	0.021	-19.904	1.382	3.04×10^{-5}
L00-L99	Daily living	Standard	SRTI	16-17	Y	No model - fewer than 3 periods with positive claims							
L00-L99	Daily living	Standard	SRTI	16-17	N	No model - fewer than 3 periods with positive claims							
L00-L99	Daily living	Standard	SRTI	18-19	Y	No model - fewer than 3 periods with positive claims							
L00-L99	Daily living	Standard	SRTI	18-19	N	No model - fewer than 3 periods with positive claims							
L00-L99	Daily living	Standard	SRTI	20-24	Y	No model - fewer than 3 periods with positive claims							
L00-L99	Daily living	Standard	SRTI	20-24	N	No model - fewer than 3 periods with positive claims							
L00-L99	Daily living	Standard	SRTI	25-29	Y	No model - fewer than 3 periods with positive claims							
L00-L99	Daily living	Standard	SRTI	25-29	N	No model - fewer than 3 periods with positive claims							
L00-L99	Daily living	Standard	SRTI	30-34	Y	No model - fewer than 3 periods with positive claims							

Continued on next page

Table 1 – continued from previous page

ICD-10 code	Component	Level	Rules	Ages	Female	Model type	A	s.e.	r	s.e.	k	s.e	RSE
L00-L99	Daily living	Standard	SRTI	30-34	N	No model - fewer than 3 periods with positive claims							
L00-L99	Daily living	Standard	SRTI	35-39	Y	No model - fewer than 3 periods with positive claims							
L00-L99	Daily living	Standard	SRTI	35-39	N	No model - fewer than 3 periods with positive claims							
L00-L99	Daily living	Standard	SRTI	40-44	Y	No model - fewer than 3 periods with positive claims							
L00-L99	Daily living	Standard	SRTI	40-44	N	No model - fewer than 3 periods with positive claims							
L00-L99	Daily living	Standard	SRTI	45-49	Y	No model - fewer than 3 periods with positive claims							
L00-L99	Daily living	Standard	SRTI	45-49	N	No model - fewer than 3 periods with positive claims							
L00-L99	Daily living	Standard	SRTI	50-54	Y	No model - fewer than 3 periods with positive claims							
L00-L99	Daily living	Standard	SRTI	50-54	N	No model - fewer than 3 periods with positive claims							
L00-L99	Daily living	Standard	SRTI	55-59	Y	No model - fewer than 3 periods with positive claims							
L00-L99	Daily living	Standard	SRTI	55-59	N	No model - fewer than 3 periods with positive claims							
L00-L99	Daily living	Standard	SRTI	60-64	Y	No model - fewer than 3 periods with positive claims							
L00-L99	Daily living	Standard	SRTI	60-64	N	No model - fewer than 3 periods with positive claims							
L00-L99	Daily living	Standard	SRTI	65-69	Y	No model - fewer than 3 periods with positive claims							
L00-L99	Daily living	Standard	SRTI	65-69	N	No model - fewer than 3 periods with positive claims							
L00-L99	Daily living	Standard	SRTI	70+	Y	No model - fewer than 3 periods with positive claims							
L00-L99	Daily living	Standard	SRTI	70+	N	No model - fewer than 3 periods with positive claims							
L00-L99	Daily living	Standard	non-SRTI	16-17	Y	LM	6.81×10^{-4}	—	0.033	9.74×10^{-3}	0.352	0.42	0.902
L00-L99	Daily living	Standard	non-SRTI	16-17	N	LM	1.12×10^{-3}	—	-0.03	0.033	2.284	2.144	1.587
L00-L99	Daily living	Standard	non-SRTI	18-19	Y	LM	1.03×10^{-3}	—	-9.03×10^{-3}	9.73×10^{-3}	0.408	0.484	0.802
L00-L99	Daily living	Standard	non-SRTI	18-19	N	LM	1.41×10^{-3}	—	0.037	0.02	-3.408	1.401	0.546
L00-L99	Daily living	Standard	non-SRTI	20-24	Y	LG	1.22×10^{-3}	1.30×10^{-4}	0.088	0.012	-5.41	0.522	1.15×10^{-4}
L00-L99	Daily living	Standard	non-SRTI	20-24	N	LG	0.017	0.233	0.043	0.014	-6.601	13.638	1.28×10^{-4}
L00-L99	Daily living	Standard	non-SRTI	25-29	Y	LG	8.53×10^{-4}	3.83×10^{-5}	0.112	0.018	-4.188	0.604	1.45×10^{-4}
L00-L99	Daily living	Standard	non-SRTI	25-29	N	LG	4.73×10^{-4}	2.61×10^{-5}	0.301	0.11	-12.814	4.655	1.37×10^{-4}
L00-L99	Daily living	Standard	non-SRTI	30-34	Y	LG	2.63×10^{-3}	2.72×10^{-4}	0.07	7.42×10^{-3}	-4.254	0.261	1.86×10^{-4}
L00-L99	Daily living	Standard	non-SRTI	30-34	N	LG	1.43×10^{-3}	1.64×10^{-4}	0.061	0.011	-2.875	0.302	2.08×10^{-4}
L00-L99	Daily living	Standard	non-SRTI	35-39	Y	LG	2.02×10^{-3}	5.24×10^{-5}	0.096	6.57×10^{-3}	-4.105	0.24	1.42×10^{-4}
L00-L99	Daily living	Standard	non-SRTI	35-39	N	LG	1.55×10^{-3}	3.49×10^{-5}	0.17	0.021	-5.326	0.633	1.90×10^{-4}
L00-L99	Daily living	Standard	non-SRTI	40-44	Y	LG	3.24×10^{-3}	1.19×10^{-4}	0.072	5.65×10^{-3}	-2.914	0.169	2.48×10^{-4}
L00-L99	Daily living	Standard	non-SRTI	40-44	N	LG	3.59×10^{-3}	1.48×10^{-4}	0.072	5.14×10^{-3}	-3.393	0.169	2.31×10^{-4}
L00-L99	Daily living	Standard	non-SRTI	45-49	Y	LG	4.20×10^{-3}	1.28×10^{-4}	0.077	4.59×10^{-3}	-3.581	0.158	2.33×10^{-4}
L00-L99	Daily living	Standard	non-SRTI	45-49	N	LG	3.39×10^{-3}	7.33×10^{-5}	0.1	6.01×10^{-3}	-4.284	0.223	2.09×10^{-4}
L00-L99	Daily living	Standard	non-SRTI	50-54	Y	LG	6.96×10^{-3}	3.67×10^{-4}	0.061	3.48×10^{-3}	-3.501	0.105	2.77×10^{-4}
L00-L99	Daily living	Standard	non-SRTI	50-54	N	LG	4.63×10^{-3}	8.53×10^{-5}	0.099	4.07×10^{-3}	-4.953	0.169	1.76×10^{-4}
L00-L99	Daily living	Standard	non-SRTI	55-59	Y	LG	6.30×10^{-3}	1.83×10^{-4}	0.077	3.46×10^{-3}	-4.099	0.128	2.34×10^{-4}
L00-L99	Daily living	Standard	non-SRTI	55-59	N	LG	5.35×10^{-3}	1.35×10^{-4}	0.087	4.08×10^{-3}	-4.491	0.162	2.20×10^{-4}
L00-L99	Daily living	Standard	non-SRTI	60-64	Y	LG	0.011	3.51×10^{-4}	0.082	3.92×10^{-3}	-4.57	0.156	4.03×10^{-4}
L00-L99	Daily living	Standard	non-SRTI	60-64	N	LG	0.011	4.13×10^{-4}	0.069	3.13×10^{-3}	-3.946	0.108	3.64×10^{-4}
L00-L99	Daily living	Standard	non-SRTI	65-69	Y	LG	5.86×10^{-3}	1.04×10^{-4}	0.116	4.56×10^{-3}	-6.305	0.215	1.99×10^{-4}
L00-L99	Daily living	Standard	non-SRTI	65-69	N	LG	5.63×10^{-3}	2.02×10^{-4}	0.098	6.69×10^{-3}	-5.317	0.293	3.26×10^{-4}
L00-L99	Daily living	Standard	non-SRTI	70+	Y	LG	5.53×10^{-4}	2.13×10^{-5}	0.236	0.017	-16.683	1.137	2.22×10^{-5}
L00-L99	Daily living	Standard	non-SRTI	70+	N	LG	4.38×10^{-4}	1.42×10^{-5}	0.282	0.024	-19.549	1.593	2.22×10^{-5}
L00-L99	Mobility	Standard	non-SRTI	16-17	Y	LM	6.58×10^{-4}	—	0.059	0.019	-0.673	0.992	0.873
L00-L99	Mobility	Standard	non-SRTI	16-17	N	LM	1.21×10^{-3}	—	0.074	0.04	-4.599	2.657	1.372
L00-L99	Mobility	Standard	non-SRTI	18-19	Y	LM	6.52×10^{-4}	—	6.34×10^{-3}	3.85×10^{-3}	-0.15	0.223	0.416
L00-L99	Mobility	Standard	non-SRTI	18-19	N	LM	6.18×10^{-4}	—	6.50×10^{-3}	0.017	-0.088	1.101	1.308
L00-L99	Mobility	Standard	non-SRTI	20-24	Y	LG	7.47×10^{-4}	3.12×10^{-5}	0.153	0.029	-5.677	1.037	1.45×10^{-4}
L00-L99	Mobility	Standard	non-SRTI	20-24	N	LG	5.26×10^{-4}	5.27×10^{-5}	0.096	0.025	-4.126	0.927	1.42×10^{-4}
L00-L99	Mobility	Standard	non-SRTI	25-29	Y	LG	1.58×10^{-3}	1.29×10^{-4}	0.072	7.13×10^{-3}	-4.158	0.257	1.15×10^{-4}
L00-L99	Mobility	Standard	non-SRTI	25-29	N	LG	7.23×10^{-4}	5.97×10^{-5}	0.109	0.021	-5.646	0.917	1.23×10^{-4}

Continued on next page

Table 1 – continued from previous page

ICD-10 code	Component	Level	Rules	Ages	Female	Model type	A	s.e.	r	s.e.	k	s.e	RSE
L00-L99	Mobility	Standard	non-SRTI	30-34	Y	LG	1.49×10^{-3}	3.78×10^{-5}	0.137	0.016	-4.39	0.487	1.85×10^{-4}
L00-L99	Mobility	Standard	non-SRTI	30-34	N	LG	1.11×10^{-3}	3.07×10^{-5}	0.14	0.019	-3.889	0.515	1.64×10^{-4}
L00-L99	Mobility	Standard	non-SRTI	35-39	Y	LG	2.02×10^{-3}	7.03×10^{-5}	0.091	7.70×10^{-3}	-4.067	0.283	1.69×10^{-4}
L00-L99	Mobility	Standard	non-SRTI	35-39	N	LG	1.38×10^{-3}	2.99×10^{-5}	0.139	0.014	-4.037	0.406	1.55×10^{-4}
L00-L99	Mobility	Standard	non-SRTI	40-44	Y	LG	3.29×10^{-3}	1.08×10^{-4}	0.078	5.61×10^{-3}	-3.35	0.184	2.32×10^{-4}
L00-L99	Mobility	Standard	non-SRTI	40-44	N	LG	3.01×10^{-3}	8.29×10^{-5}	0.108	0.01	-4.025	0.34	3.03×10^{-4}
L00-L99	Mobility	Standard	non-SRTI	45-49	Y	LG	2.50×10^{-3}	1.09×10^{-4}	0.077	8.78×10^{-3}	-2.792	0.254	2.94×10^{-4}
L00-L99	Mobility	Standard	non-SRTI	45-49	N	LG	2.90×10^{-3}	5.48×10^{-5}	0.103	6.38×10^{-3}	-3.841	0.212	1.94×10^{-4}
L00-L99	Mobility	Standard	non-SRTI	50-54	Y	LG	3.90×10^{-3}	1.05×10^{-4}	0.086	5.93×10^{-3}	-3.512	0.198	2.76×10^{-4}
L00-L99	Mobility	Standard	non-SRTI	50-54	N	LG	3.79×10^{-3}	1.61×10^{-4}	0.077	6.96×10^{-3}	-3.328	0.228	3.33×10^{-4}
L00-L99	Mobility	Standard	non-SRTI	55-59	Y	LG	2.45×10^{-3}	5.53×10^{-5}	0.129	0.013	-3.981	0.381	2.68×10^{-4}
L00-L99	Mobility	Standard	non-SRTI	55-59	N	LG	3.52×10^{-3}	9.13×10^{-5}	0.091	6.60×10^{-3}	-3.544	0.219	2.70×10^{-4}
L00-L99	Mobility	Standard	non-SRTI	60-64	Y	LG	5.94×10^{-3}	1.05×10^{-4}	0.095	4.21×10^{-3}	-4.215	0.158	2.64×10^{-4}
L00-L99	Mobility	Standard	non-SRTI	60-64	N	LG	4.57×10^{-3}	8.12×10^{-5}	0.124	8.53×10^{-3}	-4.536	0.292	3.38×10^{-4}
L00-L99	Mobility	Standard	non-SRTI	65-69	Y	LG	4.81×10^{-3}	3.54×10^{-4}	0.074	4.63×10^{-3}	-4.93	0.172	1.71×10^{-4}
L00-L99	Mobility	Standard	non-SRTI	65-69	N	LG	2.73×10^{-3}	5.44×10^{-5}	0.152	0.011	-6.98	0.497	1.98×10^{-4}
L00-L99	Mobility	Standard	non-SRTI	70+	Y	LG	2.73×10^{-4}	3.37×10^{-5}	0.224	0.029	-16.886	1.956	1.39×10^{-5}
L00-L99	Mobility	Standard	non-SRTI	70+	N	LG	3.50×10^{-4}	3.24×10^{-5}	0.203	0.028	-14.493	1.822	2.51×10^{-5}
M00-M99	Daily living	Enhanced	SRTI	16-17	Y	No model - fewer than 3 periods with positive claims							
M00-M99	Daily living	Enhanced	SRTI	16-17	N	No model - fewer than 3 periods with positive claims							
M00-M99	Daily living	Enhanced	SRTI	18-19	Y	No model - fewer than 3 periods with positive claims							
M00-M99	Daily living	Enhanced	SRTI	18-19	N	No model - fewer than 3 periods with positive claims							
M00-M99	Daily living	Enhanced	SRTI	20-24	Y	No model - fewer than 3 periods with positive claims							
M00-M99	Daily living	Enhanced	SRTI	20-24	N	No model - fewer than 3 periods with positive claims							
M00-M99	Daily living	Enhanced	SRTI	25-29	Y	No model - fewer than 3 periods with positive claims							
M00-M99	Daily living	Enhanced	SRTI	25-29	N	No model - fewer than 3 periods with positive claims							
M00-M99	Daily living	Enhanced	SRTI	30-34	Y	No model - fewer than 3 periods with positive claims							
M00-M99	Daily living	Enhanced	SRTI	30-34	N	No model - fewer than 3 periods with positive claims							
M00-M99	Daily living	Enhanced	SRTI	35-39	Y	No model - fewer than 3 periods with positive claims							
M00-M99	Daily living	Enhanced	SRTI	35-39	N	No model - fewer than 3 periods with positive claims							
M00-M99	Daily living	Enhanced	SRTI	40-44	Y	LG	3.10×10^{-5}	2.00×10^{-6}	0.218	0.045	-13.393	2.627	4.75×10^{-6}
M00-M99	Daily living	Enhanced	SRTI	40-44	N	No model - fewer than 3 periods with positive claims							
M00-M99	Daily living	Enhanced	SRTI	45-49	Y	LG	7.56×10^{-5}	6.00×10^{-6}	0.12	0.021	-6.63	1.034	1.15×10^{-5}
M00-M99	Daily living	Enhanced	SRTI	45-49	N	LM	6.25×10^{-5}	—	0.032	0.02	-2.226	1.371	1.031
M00-M99	Daily living	Enhanced	SRTI	50-54	Y	LG	1.23×10^{-4}	4.55×10^{-6}	0.103	9.23×10^{-3}	-5.051	0.384	1.04×10^{-5}
M00-M99	Daily living	Enhanced	SRTI	50-54	N	LG	7.80×10^{-5}	9.28×10^{-6}	0.099	0.021	-5.595	0.933	1.32×10^{-5}
M00-M99	Daily living	Enhanced	SRTI	55-59	Y	LG	2.00×10^{-4}	1.46×10^{-5}	0.073	5.94×10^{-3}	-4.444	0.22	1.10×10^{-5}
M00-M99	Daily living	Enhanced	SRTI	55-59	N	LG	9.74×10^{-5}	4.33×10^{-6}	0.099	0.012	-4.167	0.446	1.24×10^{-5}
M00-M99	Daily living	Enhanced	SRTI	60-64	Y	LG	4.89×10^{-4}	4.19×10^{-5}	0.067	4.15×10^{-3}	-4.561	0.135	1.60×10^{-5}
M00-M99	Daily living	Enhanced	SRTI	60-64	N	LG	2.63×10^{-4}	7.20×10^{-6}	0.099	6.53×10^{-3}	-4.665	0.26	1.68×10^{-5}
M00-M99	Daily living	Enhanced	SRTI	65-69	Y	LG	2.60×10^{-4}	5.77×10^{-6}	0.137	7.80×10^{-3}	-7.6	0.391	1.26×10^{-5}
M00-M99	Daily living	Enhanced	SRTI	65-69	N	LG	3.17×10^{-4}	1.63×10^{-5}	0.095	5.75×10^{-3}	-6.083	0.265	1.23×10^{-5}
M00-M99	Daily living	Enhanced	SRTI	70+	Y	LG	5.88×10^{-5}	4.59×10^{-6}	0.19	0.012	-14.528	0.78	1.33×10^{-6}
M00-M99	Daily living	Enhanced	SRTI	70+	N	LG	4.13×10^{-5}	1.27×10^{-6}	0.256	0.014	-18.436	0.959	1.18×10^{-6}
M00-M99	Daily living	Enhanced	non-SRTI	16-17	Y	LG	0.013	1.38×10^{-4}	0.132	6.28×10^{-3}	-4.23	0.193	6.63×10^{-4}
M00-M99	Daily living	Enhanced	non-SRTI	16-17	N	LG	0.013	1.52×10^{-4}	0.117	5.67×10^{-3}	-3.809	0.173	6.70×10^{-4}
M00-M99	Daily living	Enhanced	non-SRTI	18-19	Y	LG	0.01	2.20×10^{-4}	0.077	3.51×10^{-3}	-3.251	0.113	4.67×10^{-4}
M00-M99	Daily living	Enhanced	non-SRTI	18-19	N	LG	7.80×10^{-3}	8.03×10^{-5}	0.108	3.41×10^{-3}	-4.467	0.126	2.57×10^{-4}
M00-M99	Daily living	Enhanced	non-SRTI	20-24	Y	LG	0.011	1.64×10^{-4}	0.089	4.00×10^{-3}	-3.143	0.122	5.26×10^{-4}
M00-M99	Daily living	Enhanced	non-SRTI	20-24	N	LG	8.73×10^{-3}	2.07×10^{-4}	0.066	2.23×10^{-3}	-3.411	0.071	2.38×10^{-4}

Continued on next page

Table 1 – continued from previous page

ICD-10 code	Component	Level	Rules	Ages	Female	Model type	A	s.e.	r	s.e.	k	s.e	RSE
M00-M99	Daily living	Enhanced	non-SRTI	25-29	Y	LG	0.011	1.47×10^{-4}	0.089	3.48×10^{-3}	-3.155	0.106	4.74×10^{-4}
M00-M99	Daily living	Enhanced	non-SRTI	25-29	N	LG	6.33×10^{-3}	1.21×10^{-4}	0.075	3.07×10^{-3}	-3.191	0.098	2.51×10^{-4}
M00-M99	Daily living	Enhanced	non-SRTI	30-34	Y	LG	0.017	2.31×10^{-4}	0.092	3.96×10^{-3}	-3.174	0.119	7.97×10^{-4}
M00-M99	Daily living	Enhanced	non-SRTI	30-34	N	LG	8.82×10^{-3}	1.89×10^{-4}	0.071	3.14×10^{-3}	-2.985	0.095	3.72×10^{-4}
M00-M99	Daily living	Enhanced	non-SRTI	35-39	Y	LG	0.017	2.01×10^{-4}	0.091	3.22×10^{-3}	-3.31	0.101	6.52×10^{-4}
M00-M99	Daily living	Enhanced	non-SRTI	35-39	N	LG	7.81×10^{-3}	1.15×10^{-4}	0.09	4.12×10^{-3}	-3.109	0.123	3.87×10^{-4}
M00-M99	Daily living	Enhanced	non-SRTI	40-44	Y	LG	0.025	2.40×10^{-4}	0.101	3.28×10^{-3}	-3.42	0.1	9.10×10^{-4}
M00-M99	Daily living	Enhanced	non-SRTI	40-44	N	LG	0.012	1.1×10^{-4}	0.107	3.8×10^{-3}	-3.436	0.112	4.6×10^{-4}
M00-M99	Daily living	Enhanced	non-SRTI	45-49	Y	LG	0.027	2.76×10^{-4}	0.094	2.80×10^{-3}	-3.532	0.091	8.79×10^{-4}
M00-M99	Daily living	Enhanced	non-SRTI	45-49	N	LG	0.013	1.61×10^{-4}	0.09	3.24×10^{-3}	-3.366	0.103	4.98×10^{-4}
M00-M99	Daily living	Enhanced	non-SRTI	50-54	Y	LG	0.037	4.09×10^{-4}	0.089	2.62×10^{-3}	-3.57	0.088	1.12×10^{-3}
M00-M99	Daily living	Enhanced	non-SRTI	50-54	N	LG	0.018	2.10×10^{-4}	0.086	2.55×10^{-3}	-3.488	0.085	5.55×10^{-4}
M00-M99	Daily living	Enhanced	non-SRTI	55-59	Y	LG	0.035	3.78×10^{-4}	0.087	2.24×10^{-3}	-3.784	0.079	9.04×10^{-4}
M00-M99	Daily living	Enhanced	non-SRTI	55-59	N	LG	0.018	2.25×10^{-4}	0.084	2.48×10^{-3}	-3.57	0.085	5.29×10^{-4}
M00-M99	Daily living	Enhanced	non-SRTI	60-64	Y	LG	0.048	3.82×10^{-4}	0.096	1.87×10^{-3}	-4.394	0.072	9.17×10^{-4}
M00-M99	Daily living	Enhanced	non-SRTI	60-64	N	LG	0.027	2.48×10^{-4}	0.092	2.07×10^{-3}	-3.986	0.075	6.24×10^{-4}
M00-M99	Daily living	Enhanced	non-SRTI	65-69	Y	LG	0.036	1.86×10^{-4}	0.152	2.74×10^{-3}	-7.505	0.127	6.03×10^{-4}
M00-M99	Daily living	Enhanced	non-SRTI	65-69	N	LG	0.022	1.19×10^{-4}	0.139	2.36×10^{-3}	-6.896	0.108	3.57×10^{-4}
M00-M99	Daily living	Enhanced	non-SRTI	70+	Y	LG	5.09×10^{-3}	9.34×10^{-5}	0.249	8.46×10^{-3}	-17.841	0.566	9.11×10^{-5}
M00-M99	Daily living	Enhanced	non-SRTI	70+	N	LG	3.26×10^{-3}	6.95×10^{-5}	0.24	9.11×10^{-3}	-17.182	0.606	6.49×10^{-5}
M00-M99	Mobility	Enhanced	non-SRTI	16-17	Y	LG	0.013	1.35×10^{-4}	0.179	0.01	-5.178	0.295	7.79×10^{-4}
M00-M99	Mobility	Enhanced	non-SRTI	16-17	N	LG	9.36×10^{-3}	8.63×10^{-5}	0.242	0.016	-6.315	0.418	5.54×10^{-4}
M00-M99	Mobility	Enhanced	non-SRTI	18-19	Y	LG	0.013	9.88×10^{-5}	0.139	4.88×10^{-3}	-4.286	0.145	5.00×10^{-4}
M00-M99	Mobility	Enhanced	non-SRTI	18-19	N	LG	8.90×10^{-3}	6.39×10^{-5}	0.138	4.47×10^{-3}	-4.558	0.141	3.08×10^{-4}
M00-M99	Mobility	Enhanced	non-SRTI	20-24	Y	LG	0.025	2.53×10^{-4}	0.109	4.12×10^{-3}	-3.657	0.127	1.04×10^{-3}
M00-M99	Mobility	Enhanced	non-SRTI	20-24	N	LG	0.012	1.48×10^{-4}	0.101	4.00×10^{-3}	-3.666	0.13	5.23×10^{-4}
M00-M99	Mobility	Enhanced	non-SRTI	25-29	Y	LG	0.037	3.91×10^{-4}	0.099	3.40×10^{-3}	-3.606	0.11	1.37×10^{-3}
M00-M99	Mobility	Enhanced	non-SRTI	25-29	N	LG	0.015	1.29×10^{-4}	0.111	3.51×10^{-3}	-3.743	0.109	5.36×10^{-4}
M00-M99	Mobility	Enhanced	non-SRTI	30-34	Y	LG	0.06	7.65×10^{-4}	0.092	3.39×10^{-3}	-3.531	0.111	2.33×10^{-3}
M00-M99	Mobility	Enhanced	non-SRTI	30-34	N	LG	0.028	2.50×10^{-4}	0.107	3.45×10^{-3}	-3.724	0.11	9.78×10^{-4}
M00-M99	Mobility	Enhanced	non-SRTI	35-39	Y	LG	0.059	7.20×10^{-4}	0.09	2.97×10^{-3}	-3.57	0.099	2.04×10^{-3}
M00-M99	Mobility	Enhanced	non-SRTI	35-39	N	LG	0.029	3.27×10^{-4}	0.097	3.43×10^{-3}	-3.562	0.111	1.10×10^{-3}
M00-M99	Mobility	Enhanced	non-SRTI	40-44	Y	LG	0.082	7.78×10^{-4}	0.101	3.10×10^{-3}	-3.752	0.102	2.71×10^{-3}
M00-M99	Mobility	Enhanced	non-SRTI	40-44	N	LG	0.043	3.44×10^{-4}	0.109	3.10×10^{-3}	-3.875	0.101	1.33×10^{-3}
M00-M99	Mobility	Enhanced	non-SRTI	45-49	Y	LG	0.085	9.38×10^{-4}	0.092	2.74×10^{-3}	-3.742	0.094	2.62×10^{-3}
M00-M99	Mobility	Enhanced	non-SRTI	45-49	N	LG	0.044	4.03×10^{-4}	0.099	2.84×10^{-3}	-3.809	0.096	1.33×10^{-3}
M00-M99	Mobility	Enhanced	non-SRTI	50-54	Y	LG	0.105	1.20×10^{-3}	0.09	2.64×10^{-3}	-3.736	0.092	3.17×10^{-3}
M00-M99	Mobility	Enhanced	non-SRTI	50-54	N	LG	0.059	5.94×10^{-4}	0.094	2.64×10^{-3}	-3.809	0.091	1.73×10^{-3}
M00-M99	Mobility	Enhanced	non-SRTI	55-59	Y	LG	0.093	1.07×10^{-3}	0.09	2.57×10^{-3}	-3.792	0.09	2.73×10^{-3}
M00-M99	Mobility	Enhanced	non-SRTI	55-59	N	LG	0.055	5.57×10^{-4}	0.094	2.61×10^{-3}	-3.832	0.091	1.58×10^{-3}
M00-M99	Mobility	Enhanced	non-SRTI	60-64	Y	LG	0.128	1.12×10^{-3}	0.093	1.98×10^{-3}	-4.189	0.074	2.71×10^{-3}
M00-M99	Mobility	Enhanced	non-SRTI	60-64	N	LG	0.085	7.65×10^{-4}	0.094	2.19×10^{-3}	-3.997	0.079	2.04×10^{-3}
M00-M99	Mobility	Enhanced	non-SRTI	65-69	Y	LG	0.085	6.68×10^{-4}	0.131	3.01×10^{-3}	-6.57	0.138	1.82×10^{-3}
M00-M99	Mobility	Enhanced	non-SRTI	65-69	N	LG	0.062	3.49×10^{-4}	0.134	2.36×10^{-3}	-6.58	0.106	1.02×10^{-3}
M00-M99	Mobility	Enhanced	non-SRTI	70+	Y	LG	8.92×10^{-3}	1.85×10^{-4}	0.245	9.40×10^{-3}	-17.454	0.626	1.82×10^{-4}
M00-M99	Mobility	Enhanced	non-SRTI	70+	N	LG	7.33×10^{-3}	1.50×10^{-4}	0.245	9.38×10^{-3}	-17.492	0.625	1.50×10^{-4}
M00-M99	Daily living	Standard	SRTI	16-17	Y	No model - fewer than 3 periods with positive claims							
M00-M99	Daily living	Standard	SRTI	16-17	N	No model - fewer than 3 periods with positive claims							
M00-M99	Daily living	Standard	SRTI	18-19	Y	No model - fewer than 3 periods with positive claims							

Continued on next page

Table 1 – continued from previous page

ICD-10 code	Component	Level	Rules	Ages	Female	Model type	A	s.e.	r	s.e.	k	s.e	RSE
M00-M99	Daily living	Standard	SRTI	18-19	N	No model - fewer than 3 periods with positive claims							
M00-M99	Daily living	Standard	SRTI	20-24	Y	No model - fewer than 3 periods with positive claims							
M00-M99	Daily living	Standard	SRTI	20-24	N	No model - fewer than 3 periods with positive claims							
M00-M99	Daily living	Standard	SRTI	25-29	Y	No model - fewer than 3 periods with positive claims							
M00-M99	Daily living	Standard	SRTI	25-29	N	No model - fewer than 3 periods with positive claims							
M00-M99	Daily living	Standard	SRTI	30-34	Y	No model - fewer than 3 periods with positive claims							
M00-M99	Daily living	Standard	SRTI	30-34	N	No model - fewer than 3 periods with positive claims							
M00-M99	Daily living	Standard	SRTI	35-39	Y	No model - fewer than 3 periods with positive claims							
M00-M99	Daily living	Standard	SRTI	35-39	N	No model - fewer than 3 periods with positive claims							
M00-M99	Daily living	Standard	SRTI	40-44	Y	LG	2.93×10^{-5}	1.74×10^{-6}	0.237	0.05	-14.392	2.931	4.64×10^{-6}
M00-M99	Daily living	Standard	SRTI	40-44	N	No model - fewer than 3 periods with positive claims							
M00-M99	Daily living	Standard	SRTI	45-49	Y	LG	7.33×10^{-5}	5.01×10^{-6}	0.127	0.022	-6.894	1.058	1.10×10^{-5}
M00-M99	Daily living	Standard	SRTI	45-49	N	LM	6.25×10^{-5}	—	0.032	0.02	-2.226	1.371	1.031
M00-M99	Daily living	Standard	SRTI	50-54	Y	LG	1.27×10^{-4}	4.65×10^{-6}	0.104	9.25×10^{-3}	-5.124	0.388	1.06×10^{-5}
M00-M99	Daily living	Standard	SRTI	50-54	N	LG	7.80×10^{-5}	9.28×10^{-6}	0.099	0.021	-5.595	0.933	1.32×10^{-5}
M00-M99	Daily living	Standard	SRTI	55-59	Y	LG	2.02×10^{-4}	1.54×10^{-5}	0.073	6.01×10^{-3}	-4.435	0.221	1.12×10^{-5}
M00-M99	Daily living	Standard	SRTI	55-59	N	LG	9.74×10^{-5}	4.33×10^{-6}	0.099	0.012	-4.167	0.446	1.24×10^{-5}
M00-M99	Daily living	Standard	SRTI	60-64	Y	LG	4.69×10^{-4}	3.82×10^{-5}	0.069	4.33×10^{-3}	-4.651	0.147	1.61×10^{-5}
M00-M99	Daily living	Standard	SRTI	60-64	N	LG	2.68×10^{-4}	7.54×10^{-6}	0.097	6.35×10^{-3}	-4.623	0.253	1.68×10^{-5}
M00-M99	Daily living	Standard	SRTI	65-69	Y	LG	1.48×10^{-4}	5.47×10^{-6}	0.127	0.012	-6.957	0.565	1.17×10^{-5}
M00-M99	Daily living	Standard	SRTI	65-69	N	LG	2.40×10^{-4}	3.35×10^{-5}	0.071	5.82×10^{-3}	-5.27	0.194	8.47×10^{-6}
M00-M99	Daily living	Standard	SRTI	70+	Y	LG	2.72×10^{-5}	5.40×10^{-6}	0.149	0.016	-11.82	0.892	8.11×10^{-7}
M00-M99	Daily living	Standard	SRTI	70+	N	LG	1.67×10^{-5}	7.59×10^{-7}	0.284	0.025	-20.532	1.739	7.62×10^{-7}
M00-M99	Daily living	Standard	non-SRTI	16-17	Y	LG	0.014	1.78×10^{-4}	0.105	4.57×10^{-3}	-3.823	0.15	6.52×10^{-4}
M00-M99	Daily living	Standard	non-SRTI	16-17	N	LG	0.013	1.98×10^{-4}	0.102	5.27×10^{-3}	-3.524	0.164	7.42×10^{-4}
M00-M99	Daily living	Standard	non-SRTI	18-19	Y	LG	0.01	2.88×10^{-4}	0.076	4.67×10^{-3}	-3.218	0.15	6.15×10^{-4}
M00-M99	Daily living	Standard	non-SRTI	18-19	N	LG	8.20×10^{-3}	8.63×10^{-5}	0.098	2.67×10^{-3}	-4.324	0.101	2.25×10^{-4}
M00-M99	Daily living	Standard	non-SRTI	20-24	Y	LG	0.014	2.51×10^{-4}	0.076	3.11×10^{-3}	-3.21	0.099	5.39×10^{-4}
M00-M99	Daily living	Standard	non-SRTI	20-24	N	LG	9.49×10^{-3}	1.92×10^{-4}	0.069	1.97×10^{-3}	-3.642	0.066	2.18×10^{-4}
M00-M99	Daily living	Standard	non-SRTI	25-29	Y	LG	0.016	2.44×10^{-4}	0.078	2.62×10^{-3}	-3.28	0.085	5.29×10^{-4}
M00-M99	Daily living	Standard	non-SRTI	25-29	N	LG	7.96×10^{-3}	1.35×10^{-4}	0.076	2.76×10^{-3}	-3.203	0.088	2.83×10^{-4}
M00-M99	Daily living	Standard	non-SRTI	30-34	Y	LG	0.026	4.32×10^{-4}	0.077	2.73×10^{-3}	-3.272	0.088	9.11×10^{-4}
M00-M99	Daily living	Standard	non-SRTI	30-34	N	LG	0.013	2.37×10^{-4}	0.073	2.39×10^{-3}	-3.304	0.078	4.09×10^{-4}
M00-M99	Daily living	Standard	non-SRTI	35-39	Y	LG	0.027	3.49×10^{-4}	0.079	2.14×10^{-3}	-3.498	0.072	7.12×10^{-4}
M00-M99	Daily living	Standard	non-SRTI	35-39	N	LG	0.014	2.36×10^{-4}	0.075	2.54×10^{-3}	-3.324	0.083	4.42×10^{-4}
M00-M99	Daily living	Standard	non-SRTI	40-44	Y	LG	0.039	4.63×10^{-4}	0.085	2.49×10^{-3}	-3.47	0.083	1.17×10^{-3}
M00-M99	Daily living	Standard	non-SRTI	40-44	N	LG	0.02	2.42×10^{-4}	0.087	2.66×10^{-3}	-3.474	0.088	6.49×10^{-4}
M00-M99	Daily living	Standard	non-SRTI	45-49	Y	LG	0.043	5.26×10^{-4}	0.081	2.05×10^{-3}	-3.672	0.072	1.05×10^{-3}
M00-M99	Daily living	Standard	non-SRTI	45-49	N	LG	0.023	2.76×10^{-4}	0.081	2.07×10^{-3}	-3.669	0.072	5.61×10^{-4}
M00-M99	Daily living	Standard	non-SRTI	50-54	Y	LG	0.057	7.58×10^{-4}	0.078	1.92×10^{-3}	-3.744	0.068	1.28×10^{-3}
M00-M99	Daily living	Standard	non-SRTI	50-54	N	LG	0.032	3.97×10^{-4}	0.079	1.84×10^{-3}	-3.78	0.065	6.80×10^{-4}
M00-M99	Daily living	Standard	non-SRTI	55-59	Y	LG	0.053	6.87×10^{-4}	0.079	1.84×10^{-3}	-3.956	0.067	1.09×10^{-3}
M00-M99	Daily living	Standard	non-SRTI	55-59	N	LG	0.031	4.03×10^{-4}	0.08	1.91×10^{-3}	-3.96	0.07	6.60×10^{-4}
M00-M99	Daily living	Standard	non-SRTI	60-64	Y	LG	0.072	7.76×10^{-4}	0.086	1.68×10^{-3}	-4.381	0.066	1.25×10^{-3}
M00-M99	Daily living	Standard	non-SRTI	60-64	N	LG	0.047	4.79×10^{-4}	0.085	1.65×10^{-3}	-4.247	0.063	8.27×10^{-4}
M00-M99	Daily living	Standard	non-SRTI	65-69	Y	LG	0.05	3.73×10^{-4}	0.142	3.31×10^{-3}	-7.237	0.156	1.07×10^{-3}
M00-M99	Daily living	Standard	non-SRTI	65-69	N	LG	0.035	2.58×10^{-4}	0.137	3.07×10^{-3}	-6.94	0.143	7.23×10^{-4}
M00-M99	Daily living	Standard	non-SRTI	70+	Y	LG	6.33×10^{-3}	1.29×10^{-4}	0.244	8.98×10^{-3}	-17.497	0.599	1.22×10^{-4}
M00-M99	Daily living	Standard	non-SRTI	70+	N	LG	4.70×10^{-3}	9.76×10^{-5}	0.243	9.25×10^{-3}	-17.393	0.616	9.44×10^{-5}
M00-M99	Mobility	Standard	non-SRTI	16-17	Y	LG	6.52×10^{-3}	7.46×10^{-5}	0.185	0.013	-5.035	0.342	4.45×10^{-4}

Continued on next page

Table 1 – continued from previous page

ICD-10 code	Component	Level	Rules	Ages	Female	Model type	A	s.e.	r	s.e.	k	s.e	RSE
M00-M99	Mobility	Standard	non-SRTI	16-17	N	LG	4.50×10^{-3}	7.25×10^{-5}	0.326	0.046	-7.763	1.099	5.00×10^{-4}
M00-M99	Mobility	Standard	non-SRTI	18-19	Y	LG	5.97×10^{-3}	5.34×10^{-5}	0.141	6.28×10^{-3}	-3.924	0.17	2.86×10^{-4}
M00-M99	Mobility	Standard	non-SRTI	18-19	N	LG	3.99×10^{-3}	4.95×10^{-5}	0.19	0.014	-5.346	0.396	2.93×10^{-4}
M00-M99	Mobility	Standard	non-SRTI	20-24	Y	LG	0.011	7.09×10^{-5}	0.132	3.85×10^{-3}	-3.879	0.109	3.57×10^{-4}
M00-M99	Mobility	Standard	non-SRTI	20-24	N	LG	5.93×10^{-3}	8.18×10^{-5}	0.094	4.23×10^{-3}	-3.167	0.126	2.93×10^{-4}
M00-M99	Mobility	Standard	non-SRTI	25-29	Y	LG	0.016	1.44×10^{-4}	0.105	3.46×10^{-3}	-3.469	0.104	5.80×10^{-4}
M00-M99	Mobility	Standard	non-SRTI	25-29	N	LG	6.75×10^{-3}	7.44×10^{-5}	0.109	4.74×10^{-3}	-3.378	0.136	3.25×10^{-4}
M00-M99	Mobility	Standard	non-SRTI	30-34	Y	LG	0.026	2.72×10^{-4}	0.1	3.52×10^{-3}	-3.474	0.11	1.00×10^{-3}
M00-M99	Mobility	Standard	non-SRTI	30-34	N	LG	0.012	8.51×10^{-5}	0.12	3.60×10^{-3}	-3.688	0.105	3.97×10^{-4}
M00-M99	Mobility	Standard	non-SRTI	35-39	Y	LG	0.026	2.50×10^{-4}	0.099	3.14×10^{-3}	-3.532	0.1	8.87×10^{-4}
M00-M99	Mobility	Standard	non-SRTI	35-39	N	LG	0.013	1.18×10^{-4}	0.106	3.62×10^{-3}	-3.501	0.109	4.82×10^{-4}
M00-M99	Mobility	Standard	non-SRTI	40-44	Y	LG	0.037	2.80×10^{-4}	0.111	3.13×10^{-3}	-3.743	0.097	1.16×10^{-3}
M00-M99	Mobility	Standard	non-SRTI	40-44	N	LG	0.02	1.48×10^{-4}	0.113	3.35×10^{-3}	-3.658	0.101	6.39×10^{-4}
M00-M99	Mobility	Standard	non-SRTI	45-49	Y	LG	0.04	3.61×10^{-4}	0.096	2.65×10^{-3}	-3.651	0.088	1.18×10^{-3}
M00-M99	Mobility	Standard	non-SRTI	45-49	N	LG	0.02	1.70×10^{-4}	0.104	3.09×10^{-3}	-3.636	0.098	6.50×10^{-4}
M00-M99	Mobility	Standard	non-SRTI	50-54	Y	LG	0.05	4.62×10^{-4}	0.096	2.68×10^{-3}	-3.706	0.09	1.48×10^{-3}
M00-M99	Mobility	Standard	non-SRTI	50-54	N	LG	0.028	2.16×10^{-4}	0.1	2.51×10^{-3}	-3.726	0.083	7.48×10^{-4}
M00-M99	Mobility	Standard	non-SRTI	55-59	Y	LG	0.046	4.45×10^{-4}	0.093	2.53×10^{-3}	-3.72	0.086	1.32×10^{-3}
M00-M99	Mobility	Standard	non-SRTI	55-59	N	LG	0.026	2.22×10^{-4}	0.1	2.72×10^{-3}	-3.738	0.09	7.68×10^{-4}
M00-M99	Mobility	Standard	non-SRTI	60-64	Y	LG	0.064	4.48×10^{-4}	0.099	1.91×10^{-3}	-4.181	0.07	1.28×10^{-3}
M00-M99	Mobility	Standard	non-SRTI	60-64	N	LG	0.043	3.29×10^{-4}	0.098	2.24×10^{-3}	-3.877	0.077	1.03×10^{-3}
M00-M99	Mobility	Standard	non-SRTI	65-69	Y	LG	0.044	2.55×10^{-4}	0.139	2.60×10^{-3}	-6.79	0.118	7.84×10^{-4}
M00-M99	Mobility	Standard	non-SRTI	65-69	N	LG	0.033	1.30×10^{-4}	0.136	1.75×10^{-3}	-6.518	0.078	4.06×10^{-4}
M00-M99	Mobility	Standard	non-SRTI	70+	Y	LG	5.07×10^{-3}	1.02×10^{-4}	0.247	9.41×10^{-3}	-17.6	0.627	1.04×10^{-4}
M00-M99	Mobility	Standard	non-SRTI	70+	N	LG	4.09×10^{-3}	8.44×10^{-5}	0.244	9.50×10^{-3}	-17.401	0.632	8.53×10^{-5}
N00-N99	Daily living	Enhanced	SRTI	16-17	Y	No model - fewer than 3 periods with positive claims							
N00-N99	Daily living	Enhanced	SRTI	16-17	N	No model - fewer than 3 periods with positive claims							
N00-N99	Daily living	Enhanced	SRTI	18-19	Y	No model - fewer than 3 periods with positive claims							
N00-N99	Daily living	Enhanced	SRTI	18-19	N	No model - fewer than 3 periods with positive claims							
N00-N99	Daily living	Enhanced	SRTI	20-24	Y	No model - fewer than 3 periods with positive claims							
N00-N99	Daily living	Enhanced	SRTI	20-24	N	No model - fewer than 3 periods with positive claims							
N00-N99	Daily living	Enhanced	SRTI	25-29	Y	No model - fewer than 3 periods with positive claims							
N00-N99	Daily living	Enhanced	SRTI	25-29	N	No model - fewer than 3 periods with positive claims							
N00-N99	Daily living	Enhanced	SRTI	30-34	Y	No model - fewer than 3 periods with positive claims							
N00-N99	Daily living	Enhanced	SRTI	30-34	N	No model - fewer than 3 periods with positive claims							
N00-N99	Daily living	Enhanced	SRTI	35-39	Y	No model - fewer than 3 periods with positive claims							
N00-N99	Daily living	Enhanced	SRTI	35-39	N	No model - fewer than 3 periods with positive claims							
N00-N99	Daily living	Enhanced	SRTI	40-44	Y	No model - fewer than 3 periods with positive claims							
N00-N99	Daily living	Enhanced	SRTI	40-44	N	LM	1.9×10^{-4}	—	-0.173	0.051	13.835	3.186	0.565
N00-N99	Daily living	Enhanced	SRTI	45-49	Y	LM	1.3×10^{-4}	—	0.107	0.041	-2.27	1.65	0.784
N00-N99	Daily living	Enhanced	SRTI	45-49	N	LM	1.28×10^{-4}	—	-0.013	9.51×10^{-3}	2.664	0.586	0.638
N00-N99	Daily living	Enhanced	SRTI	50-54	Y	LM	2.81×10^{-4}	—	0.073	0.023	-3.425	1.443	1.511
N00-N99	Daily living	Enhanced	SRTI	50-54	N	LG	1.64×10^{-4}	1.81×10^{-5}	0.228	0.124	-11.134	5.915	7.62×10^{-5}
N00-N99	Daily living	Enhanced	SRTI	55-59	Y	LG	1.93×10^{-4}	8.69×10^{-6}	0.478	0.174	-22.936	8.305	4.50×10^{-5}
N00-N99	Daily living	Enhanced	SRTI	55-59	N	LG	3.66×10^{-4}	4.25×10^{-5}	0.152	0.041	-9.159	2.244	7.59×10^{-5}
N00-N99	Daily living	Enhanced	SRTI	60-64	Y	LG	2.56×10^{-4}	1.31×10^{-5}	0.28	0.1	-10.308	3.641	7.51×10^{-5}
N00-N99	Daily living	Enhanced	SRTI	60-64	N	LG	4.44×10^{-4}	2.10×10^{-5}	0.136	0.026	-5.383	0.953	8.63×10^{-5}
N00-N99	Daily living	Enhanced	SRTI	65-69	Y	LM	1.88×10^{-4}	—	9.26×10^{-3}	0.021	-0.234	1.369	1.095
N00-N99	Daily living	Enhanced	SRTI	65-69	N	LM	1.91×10^{-4}	—	0.049	0.05	-2.773	3.564	1.599
N00-N99	Daily living	Enhanced	SRTI	70+	Y	No model - fewer than 3 periods with positive claims							
N00-N99	Daily living	Enhanced	SRTI	70+	N	No model - fewer than 3 periods with positive claims							

Continued on next page

Table 1 – continued from previous page

ICD-10 code	Component	Level	Rules	Ages	Female	Model type	A	s.e.	r	s.e.	k	s.e	RSE
N00-N99	Daily living	Enhanced	non-SRTI	16-17	Y	LG	3.77×10^{-3}	1.46×10^{-4}	0.283	0.088	-6.941	2.157	9.77×10^{-4}
N00-N99	Daily living	Enhanced	non-SRTI	16-17	N	LM	0.012	—	9.98×10^{-4}	4.76×10^{-3}	-0.746	0.267	0.61
N00-N99	Daily living	Enhanced	non-SRTI	18-19	Y	LG	1.92×10^{-3}	5.61×10^{-5}	0.189	0.033	-5.65	0.96	3.23×10^{-4}
N00-N99	Daily living	Enhanced	non-SRTI	18-19	N	LG	2.47×10^{-3}	6.77×10^{-5}	0.177	0.027	-5.682	0.835	3.69×10^{-4}
N00-N99	Daily living	Enhanced	non-SRTI	20-24	Y	LG	2.58×10^{-3}	4.78×10^{-5}	0.151	0.015	-3.763	0.373	2.76×10^{-4}
N00-N99	Daily living	Enhanced	non-SRTI	20-24	N	LG	2.60×10^{-3}	1.02×10^{-4}	0.083	7.76×10^{-3}	-3.416	0.257	2.48×10^{-4}
N00-N99	Daily living	Enhanced	non-SRTI	25-29	Y	LG	3.04×10^{-3}	3.58×10^{-5}	0.14	7.83×10^{-3}	-4.225	0.228	1.83×10^{-4}
N00-N99	Daily living	Enhanced	non-SRTI	25-29	N	LG	1.47×10^{-3}	4.15×10^{-5}	0.114	0.013	-3.598	0.379	1.84×10^{-4}
N00-N99	Daily living	Enhanced	non-SRTI	30-34	Y	LG	4.87×10^{-3}	1.09×10^{-4}	0.087	5.67×10^{-3}	-3.168	0.174	3.38×10^{-4}
N00-N99	Daily living	Enhanced	non-SRTI	30-34	N	LG	3.05×10^{-3}	7.43×10^{-5}	0.101	9.09×10^{-3}	-3.116	0.257	3.05×10^{-4}
N00-N99	Daily living	Enhanced	non-SRTI	35-39	Y	LG	3.73×10^{-3}	5.06×10^{-5}	0.109	5.53×10^{-3}	-3.629	0.17	2.09×10^{-4}
N00-N99	Daily living	Enhanced	non-SRTI	35-39	N	LG	2.69×10^{-3}	3.78×10^{-5}	0.112	5.88×10^{-3}	-3.873	0.188	1.54×10^{-4}
N00-N99	Daily living	Enhanced	non-SRTI	40-44	Y	LG	5.02×10^{-3}	5.66×10^{-5}	0.123	6.15×10^{-3}	-3.605	0.172	2.77×10^{-4}
N00-N99	Daily living	Enhanced	non-SRTI	40-44	N	LG	4.13×10^{-3}	5.92×10^{-5}	0.112	5.87×10^{-3}	-3.904	0.189	2.38×10^{-4}
N00-N99	Daily living	Enhanced	non-SRTI	45-49	Y	LG	5.32×10^{-3}	6.18×10^{-5}	0.106	4.46×10^{-3}	-3.609	0.138	2.46×10^{-4}
N00-N99	Daily living	Enhanced	non-SRTI	45-49	N	LG	3.91×10^{-3}	7.33×10^{-5}	0.101	6.90×10^{-3}	-3.132	0.196	2.98×10^{-4}
N00-N99	Daily living	Enhanced	non-SRTI	50-54	Y	LG	8.32×10^{-3}	1.34×10^{-4}	0.089	4.09×10^{-3}	-3.375	0.131	4.04×10^{-4}
N00-N99	Daily living	Enhanced	non-SRTI	50-54	N	LG	7.20×10^{-3}	7.01×10^{-5}	0.129	5.73×10^{-3}	-3.826	0.163	3.49×10^{-4}
N00-N99	Daily living	Enhanced	non-SRTI	55-59	Y	LG	6.40×10^{-3}	1.17×10^{-4}	0.085	4.15×10^{-3}	-3.268	0.132	3.27×10^{-4}
N00-N99	Daily living	Enhanced	non-SRTI	55-59	N	LG	7.12×10^{-3}	1.04×10^{-4}	0.091	4.08×10^{-3}	-3.218	0.125	3.45×10^{-4}
N00-N99	Daily living	Enhanced	non-SRTI	60-64	Y	LG	7.43×10^{-3}	7.41×10^{-5}	0.099	2.95×10^{-3}	-3.938	0.102	2.34×10^{-4}
N00-N99	Daily living	Enhanced	non-SRTI	60-64	N	LG	9.51×10^{-3}	9.19×10^{-5}	0.113	4.00×10^{-3}	-4.04	0.132	3.66×10^{-4}
N00-N99	Daily living	Enhanced	non-SRTI	65-69	Y	LG	4.14×10^{-3}	3.71×10^{-5}	0.133	4.13×10^{-3}	-5.97	0.172	1.28×10^{-4}
N00-N99	Daily living	Enhanced	non-SRTI	65-69	N	LG	5.40×10^{-3}	2.70×10^{-5}	0.138	2.49×10^{-3}	-6.189	0.104	9.61×10^{-5}
N00-N99	Daily living	Enhanced	non-SRTI	70+	Y	LG	3.05×10^{-4}	5.16×10^{-6}	0.293	0.013	-20.43	0.861	7.83×10^{-6}
N00-N99	Daily living	Enhanced	non-SRTI	70+	N	LG	3.39×10^{-4}	8.81×10^{-6}	0.274	0.019	-18.751	1.26	1.46×10^{-5}
N00-N99	Mobility	Enhanced	non-SRTI	16-17	Y	LG	4.08×10^{-3}	1.12×10^{-4}	0.476	0.135	-13.935	3.936	7.56×10^{-4}
N00-N99	Mobility	Enhanced	non-SRTI	16-17	N	LM	9.52×10^{-3}	—	4.01×10^{-3}	6.63×10^{-3}	-0.882	0.389	0.714
N00-N99	Mobility	Enhanced	non-SRTI	18-19	Y	LG	2.44×10^{-3}	7.16×10^{-5}	0.16	0.02	-6.629	0.814	3.08×10^{-4}
N00-N99	Mobility	Enhanced	non-SRTI	18-19	N	LG	2.84×10^{-3}	1.55×10^{-4}	0.098	0.012	-4.88	0.491	3.19×10^{-4}
N00-N99	Mobility	Enhanced	non-SRTI	20-24	Y	LG	4.16×10^{-3}	5.56×10^{-5}	0.148	9.99×10^{-3}	-4.317	0.283	2.98×10^{-4}
N00-N99	Mobility	Enhanced	non-SRTI	20-24	N	LG	3.55×10^{-3}	7.93×10^{-5}	0.111	7.44×10^{-3}	-4.857	0.291	2.42×10^{-4}
N00-N99	Mobility	Enhanced	non-SRTI	25-29	Y	LG	6.31×10^{-3}	9.93×10^{-5}	0.099	4.78×10^{-3}	-3.779	0.161	3.27×10^{-4}
N00-N99	Mobility	Enhanced	non-SRTI	25-29	N	LG	2.72×10^{-3}	5.82×10^{-5}	0.098	5.96×10^{-3}	-4.093	0.214	1.71×10^{-4}
N00-N99	Mobility	Enhanced	non-SRTI	30-34	Y	LG	0.01	1.67×10^{-4}	0.087	3.58×10^{-3}	-3.582	0.121	4.39×10^{-4}
N00-N99	Mobility	Enhanced	non-SRTI	30-34	N	LG	5.03×10^{-3}	1.63×10^{-4}	0.076	5.64×10^{-3}	-3.079	0.175	3.71×10^{-4}
N00-N99	Mobility	Enhanced	non-SRTI	35-39	Y	LG	9.72×10^{-3}	3.20×10^{-4}	0.066	3.21×10^{-3}	-3.282	0.1	3.94×10^{-4}
N00-N99	Mobility	Enhanced	non-SRTI	35-39	N	LG	4.18×10^{-3}	9.38×10^{-5}	0.085	4.53×10^{-3}	-3.629	0.156	2.23×10^{-4}
N00-N99	Mobility	Enhanced	non-SRTI	40-44	Y	LG	0.013	3.31×10^{-4}	0.072	3.41×10^{-3}	-3.267	0.11	5.75×10^{-4}
N00-N99	Mobility	Enhanced	non-SRTI	40-44	N	LG	6.73×10^{-3}	9.77×10^{-5}	0.096	4.10×10^{-3}	-3.794	0.14	3.03×10^{-4}
N00-N99	Mobility	Enhanced	non-SRTI	45-49	Y	LG	0.01	2.46×10^{-4}	0.078	4.32×10^{-3}	-3.201	0.138	5.67×10^{-4}
N00-N99	Mobility	Enhanced	non-SRTI	45-49	N	LG	6.35×10^{-3}	1.12×10^{-4}	0.095	5.04×10^{-3}	-3.582	0.165	3.61×10^{-4}
N00-N99	Mobility	Enhanced	non-SRTI	50-54	Y	LG	0.017	3.18×10^{-4}	0.076	2.63×10^{-3}	-3.599	0.09	5.31×10^{-4}
N00-N99	Mobility	Enhanced	non-SRTI	50-54	N	LG	0.012	1.48×10^{-4}	0.09	2.87×10^{-3}	-3.589	0.096	4.17×10^{-4}
N00-N99	Mobility	Enhanced	non-SRTI	55-59	Y	LG	9.77×10^{-3}	1.85×10^{-4}	0.084	3.91×10^{-3}	-3.471	0.131	4.63×10^{-4}
N00-N99	Mobility	Enhanced	non-SRTI	55-59	N	LG	0.011	1.37×10^{-4}	0.087	2.63×10^{-3}	-3.731	0.092	3.32×10^{-4}
N00-N99	Mobility	Enhanced	non-SRTI	60-64	Y	LG	0.014	3.20×10^{-4}	0.072	3.03×10^{-3}	-3.372	0.099	5.15×10^{-4}
N00-N99	Mobility	Enhanced	non-SRTI	60-64	N	LG	0.015	1.79×10^{-4}	0.09	2.80×10^{-3}	-3.749	0.097	4.70×10^{-4}
N00-N99	Mobility	Enhanced	non-SRTI	65-69	Y	LG	6.01×10^{-3}	9.16×10^{-5}	0.102	3.46×10^{-3}	-5.137	0.147	1.88×10^{-4}

Continued on next page

Table 1 – continued from previous page

ICD-10 code	Component	Level	Rules	Ages	Female	Model type	A	s.e.	r	s.e.	k	s.e	RSE
N00-N99	Mobility	Enhanced	non-SRTI	65-69	N	LG	7.42×10^{-3}	6.60×10^{-5}	0.114	2.65×10^{-3}	-5.67	0.116	1.61×10^{-4}
N00-N99	Mobility	Enhanced	non-SRTI	70+	Y	LG	3.73×10^{-4}	7.17×10^{-6}	0.28	0.013	-19.625	0.863	9.87×10^{-6}
N00-N99	Mobility	Enhanced	non-SRTI	70+	N	LG	5.02×10^{-4}	1.11×10^{-5}	0.263	0.012	-18.729	0.798	1.23×10^{-5}
N00-N99	Daily living	Standard	SRTI	16-17	Y	No model - fewer than 3 periods with positive claims							
N00-N99	Daily living	Standard	SRTI	16-17	N	No model - fewer than 3 periods with positive claims							
N00-N99	Daily living	Standard	SRTI	18-19	Y	No model - fewer than 3 periods with positive claims							
N00-N99	Daily living	Standard	SRTI	18-19	N	No model - fewer than 3 periods with positive claims							
N00-N99	Daily living	Standard	SRTI	20-24	Y	No model - fewer than 3 periods with positive claims							
N00-N99	Daily living	Standard	SRTI	20-24	N	No model - fewer than 3 periods with positive claims							
N00-N99	Daily living	Standard	SRTI	25-29	Y	No model - fewer than 3 periods with positive claims							
N00-N99	Daily living	Standard	SRTI	25-29	N	No model - fewer than 3 periods with positive claims							
N00-N99	Daily living	Standard	SRTI	30-34	Y	No model - fewer than 3 periods with positive claims							
N00-N99	Daily living	Standard	SRTI	30-34	N	No model - fewer than 3 periods with positive claims							
N00-N99	Daily living	Standard	SRTI	35-39	Y	No model - fewer than 3 periods with positive claims							
N00-N99	Daily living	Standard	SRTI	35-39	N	No model - fewer than 3 periods with positive claims							
N00-N99	Daily living	Standard	SRTI	40-44	Y	No model - fewer than 3 periods with positive claims							
N00-N99	Daily living	Standard	SRTI	40-44	N	LM	1.9×10^{-4}	—	-0.173	0.051	13.835	3.186	0.565
N00-N99	Daily living	Standard	SRTI	45-49	Y	LM	1.3×10^{-4}	—	0.107	0.041	-2.27	1.65	0.784
N00-N99	Daily living	Standard	SRTI	45-49	N	LM	1.28×10^{-4}	—	-0.013	9.51×10^{-3}	2.664	0.586	0.638
N00-N99	Daily living	Standard	SRTI	50-54	Y	LM	2.81×10^{-4}	—	0.073	0.023	-3.425	1.443	1.511
N00-N99	Daily living	Standard	SRTI	50-54	N	LG	1.64×10^{-4}	1.81×10^{-5}	0.228	0.124	-11.134	5.915	7.62×10^{-5}
N00-N99	Daily living	Standard	SRTI	55-59	Y	LG	1.93×10^{-4}	8.69×10^{-6}	0.478	0.174	-22.936	8.305	4.50×10^{-5}
N00-N99	Daily living	Standard	SRTI	55-59	N	LG	3.66×10^{-4}	4.25×10^{-5}	0.152	0.041	-9.159	2.244	7.59×10^{-5}
N00-N99	Daily living	Standard	SRTI	60-64	Y	LG	2.56×10^{-4}	1.31×10^{-5}	0.28	0.1	-10.308	3.641	7.51×10^{-5}
N00-N99	Daily living	Standard	SRTI	60-64	N	LG	4.44×10^{-4}	2.10×10^{-5}	0.136	0.026	-5.383	0.953	8.63×10^{-5}
N00-N99	Daily living	Standard	SRTI	65-69	Y	LM	1.55×10^{-4}	—	7.66×10^{-3}	0.033	0.055	2.029	1.126
N00-N99	Daily living	Standard	SRTI	65-69	N	LM	1.37×10^{-4}	—	0.047	0.033	-2.402	2.367	1.062
N00-N99	Daily living	Standard	SRTI	70+	Y	No model - fewer than 3 periods with positive claims							
N00-N99	Daily living	Standard	SRTI	70+	N	No model - fewer than 3 periods with positive claims							
N00-N99	Daily living	Standard	non-SRTI	16-17	Y	LG	2.66×10^{-3}	1.11×10^{-4}	0.415	0.168	-11.589	4.687	7.52×10^{-4}
N00-N99	Daily living	Standard	non-SRTI	16-17	N	LG	2.65×10^{-3}	1.37×10^{-4}	0.364	0.168	-10.524	4.838	9.07×10^{-4}
N00-N99	Daily living	Standard	non-SRTI	18-19	Y	LG	2.03×10^{-3}	9.63×10^{-5}	0.113	0.019	-4.259	0.644	3.61×10^{-4}
N00-N99	Daily living	Standard	non-SRTI	18-19	N	LG	2.10×10^{-3}	8.64×10^{-5}	0.174	0.033	-7.365	1.346	3.82×10^{-4}
N00-N99	Daily living	Standard	non-SRTI	20-24	Y	LG	2.98×10^{-3}	9.40×10^{-5}	0.094	9.86×10^{-3}	-3.073	0.287	3.45×10^{-4}
N00-N99	Daily living	Standard	non-SRTI	20-24	N	LG	2.55×10^{-3}	9.92×10^{-5}	0.088	6.73×10^{-3}	-4.436	0.265	1.76×10^{-4}
N00-N99	Daily living	Standard	non-SRTI	25-29	Y	LG	3.51×10^{-3}	7.22×10^{-5}	0.088	4.58×10^{-3}	-3.692	0.158	1.86×10^{-4}
N00-N99	Daily living	Standard	non-SRTI	25-29	N	LG	1.65×10^{-3}	4.98×10^{-5}	0.088	7.01×10^{-3}	-3.512	0.233	1.37×10^{-4}
N00-N99	Daily living	Standard	non-SRTI	30-34	Y	LG	6.69×10^{-3}	3.03×10^{-4}	0.064	4.11×10^{-3}	-3.277	0.127	3.45×10^{-4}
N00-N99	Daily living	Standard	non-SRTI	30-34	N	LG	4.87×10^{-3}	2.89×10^{-4}	0.06	4.11×10^{-3}	-3.347	0.121	2.39×10^{-4}
N00-N99	Daily living	Standard	non-SRTI	35-39	Y	LG	5.02×10^{-3}	1.19×10^{-4}	0.077	3.50×10^{-3}	-3.573	0.12	2.13×10^{-4}
N00-N99	Daily living	Standard	non-SRTI	35-39	N	LG	3.10×10^{-3}	8.01×10^{-5}	0.079	4.02×10^{-3}	-3.637	0.14	1.49×10^{-4}
N00-N99	Daily living	Standard	non-SRTI	40-44	Y	LG	6.70×10^{-3}	1.65×10^{-4}	0.076	4.31×10^{-3}	-3.055	0.133	3.79×10^{-4}
N00-N99	Daily living	Standard	non-SRTI	40-44	N	LG	4.82×10^{-3}	7.52×10^{-5}	0.1	4.60×10^{-3}	-4.033	0.163	2.33×10^{-4}
N00-N99	Daily living	Standard	non-SRTI	45-49	Y	LG	6.91×10^{-3}	9.35×10^{-5}	0.088	3.01×10^{-3}	-3.658	0.103	2.42×10^{-4}
N00-N99	Daily living	Standard	non-SRTI	45-49	N	LG	5.02×10^{-3}	7.40×10^{-5}	0.089	3.46×10^{-3}	-3.586	0.117	2.03×10^{-4}
N00-N99	Daily living	Standard	non-SRTI	50-54	Y	LG	0.011	2.12×10^{-4}	0.076	2.74×10^{-3}	-3.511	0.093	3.80×10^{-4}
N00-N99	Daily living	Standard	non-SRTI	50-54	N	LG	9.70×10^{-3}	1.90×10^{-4}	0.079	3.63×10^{-3}	-3.233	0.116	4.47×10^{-4}
N00-N99	Daily living	Standard	non-SRTI	55-59	Y	LG	8.26×10^{-3}	1.31×10^{-4}	0.082	2.94×10^{-3}	-3.49	0.099	2.95×10^{-4}
N00-N99	Daily living	Standard	non-SRTI	55-59	N	LG	9.85×10^{-3}	2.09×10^{-4}	0.072	2.82×10^{-3}	-3.299	0.092	3.54×10^{-4}
N00-N99	Daily living	Standard	non-SRTI	60-64	Y	LG	0.011	1.37×10^{-4}	0.083	2.18×10^{-3}	-3.899	0.079	2.69×10^{-4}
N00-N99	Daily living	Standard	non-SRTI	60-64	N	LG	0.013	1.64×10^{-4}	0.091	3.12×10^{-3}	-3.7	0.107	4.57×10^{-4}

Continued on next page

Table 1 – continued from previous page

ICD-10 code	Component	Level	Rules	Ages	Female	Model type	A	s.e.	r	s.e.	k	s.e	RSE
N00-N99	Daily living	Standard	non-SRTI	65-69	Y	LG	5.36×10^{-3}	6.36×10^{-5}	0.124	4.59×10^{-3}	-5.756	0.194	1.95×10^{-4}
N00-N99	Daily living	Standard	non-SRTI	65-69	N	LG	7.17×10^{-3}	4.42×10^{-5}	0.124	2.26×10^{-3}	-5.97	0.099	1.26×10^{-4}
N00-N99	Daily living	Standard	non-SRTI	70+	Y	LG	4.75×10^{-4}	1.75×10^{-5}	0.21	0.011	-15.215	0.725	1.23×10^{-5}
N00-N99	Daily living	Standard	non-SRTI	70+	N	LG	4.66×10^{-4}	1.36×10^{-5}	0.241	0.014	-16.994	0.911	1.48×10^{-5}
N00-N99	Mobility	Standard	non-SRTI	16-17	Y	LM	4.95×10^{-3}	—	1.96×10^{-3}	5.18×10^{-3}	-0.023	0.29	0.665
N00-N99	Mobility	Standard	non-SRTI	16-17	N	LM	4.98×10^{-3}	—	-3.37×10^{-3}	6.41×10^{-3}	-0.049	0.356	0.701
N00-N99	Mobility	Standard	non-SRTI	18-19	Y	LG	1.46×10^{-3}	2.43×10^{-4}	0.082	0.022	-4.414	0.856	3.23×10^{-4}
N00-N99	Mobility	Standard	non-SRTI	18-19	N	LM	4.68×10^{-3}	—	4.24×10^{-3}	3.62×10^{-3}	-1.135	0.201	0.454
N00-N99	Mobility	Standard	non-SRTI	20-24	Y	LG	2.04×10^{-3}	2.84×10^{-5}	0.338	0.043	-7.372	0.941	2.00×10^{-4}
N00-N99	Mobility	Standard	non-SRTI	20-24	N	LG	2.05×10^{-3}	6.41×10^{-5}	0.11	0.012	-4.21	0.401	2.31×10^{-4}
N00-N99	Mobility	Standard	non-SRTI	25-29	Y	LG	3.05×10^{-3}	3.20×10^{-5}	0.171	9.76×10^{-3}	-5.229	0.293	1.77×10^{-4}
N00-N99	Mobility	Standard	non-SRTI	25-29	N	LG	1.27×10^{-3}	2.75×10^{-5}	0.142	0.015	-4.173	0.425	1.44×10^{-4}
N00-N99	Mobility	Standard	non-SRTI	30-34	Y	LG	4.53×10^{-3}	6.90×10^{-5}	0.109	6.46×10^{-3}	-3.408	0.187	2.98×10^{-4}
N00-N99	Mobility	Standard	non-SRTI	30-34	N	LG	2.56×10^{-3}	4.66×10^{-5}	0.159	0.016	-4.176	0.411	2.68×10^{-4}
N00-N99	Mobility	Standard	non-SRTI	35-39	Y	LG	4.79×10^{-3}	1.33×10^{-4}	0.073	4.06×10^{-3}	-3.208	0.13	2.52×10^{-4}
N00-N99	Mobility	Standard	non-SRTI	35-39	N	LG	2.79×10^{-3}	5.98×10^{-5}	0.097	6.80×10^{-3}	-3.444	0.213	2.12×10^{-4}
N00-N99	Mobility	Standard	non-SRTI	40-44	Y	LG	5.62×10^{-3}	7.27×10^{-5}	0.097	4.13×10^{-3}	-3.334	0.126	2.63×10^{-4}
N00-N99	Mobility	Standard	non-SRTI	40-44	N	LG	3.79×10^{-3}	5.67×10^{-5}	0.119	7.42×10^{-3}	-3.708	0.218	2.61×10^{-4}
N00-N99	Mobility	Standard	non-SRTI	45-49	Y	LG	4.50×10^{-3}	6.16×10^{-5}	0.114	6.44×10^{-3}	-3.357	0.179	2.84×10^{-4}
N00-N99	Mobility	Standard	non-SRTI	45-49	N	LG	3.50×10^{-3}	6.49×10^{-5}	0.12	0.01	-3.283	0.263	3.24×10^{-4}
N00-N99	Mobility	Standard	non-SRTI	50-54	Y	LG	8.24×10^{-3}	1.28×10^{-4}	0.087	3.65×10^{-3}	-3.428	0.119	3.60×10^{-4}
N00-N99	Mobility	Standard	non-SRTI	50-54	N	LG	6.97×10^{-3}	6.38×10^{-5}	0.134	5.74×10^{-3}	-3.948	0.163	3.25×10^{-4}
N00-N99	Mobility	Standard	non-SRTI	55-59	Y	LG	5.67×10^{-3}	1.18×10^{-4}	0.086	4.86×10^{-3}	-3.308	0.155	3.36×10^{-4}
N00-N99	Mobility	Standard	non-SRTI	55-59	N	LG	5.96×10^{-3}	5.00×10^{-5}	0.125	4.36×10^{-3}	-4.163	0.137	2.27×10^{-4}
N00-N99	Mobility	Standard	non-SRTI	60-64	Y	LG	6.12×10^{-3}	1.07×10^{-4}	0.087	4.32×10^{-3}	-3.247	0.135	3.22×10^{-4}
N00-N99	Mobility	Standard	non-SRTI	60-64	N	LG	7.58×10^{-3}	6.35×10^{-5}	0.123	4.23×10^{-3}	-4.102	0.133	2.84×10^{-4}
N00-N99	Mobility	Standard	non-SRTI	65-69	Y	LG	3.24×10^{-3}	3.08×10^{-5}	0.114	3.20×10^{-3}	-5.177	0.13	9.06×10^{-5}
N00-N99	Mobility	Standard	non-SRTI	65-69	N	LG	3.93×10^{-3}	2.77×10^{-5}	0.128	3.11×10^{-3}	-5.613	0.126	9.60×10^{-5}
N00-N99	Mobility	Standard	non-SRTI	70+	Y	LG	2.00×10^{-4}	5.91×10^{-6}	0.331	0.029	-23.035	1.97	1.04×10^{-5}
N00-N99	Mobility	Standard	non-SRTI	70+	N	LG	2.96×10^{-4}	9.90×10^{-6}	0.25	0.017	-17.595	1.141	1.14×10^{-5}
Other	Daily living	Enhanced	SRTI	16-17	Y	No model - fewer than 3 periods with positive claims							
Other	Daily living	Enhanced	SRTI	16-17	N	No model - fewer than 3 periods with positive claims							
Other	Daily living	Enhanced	SRTI	18-19	Y	No model - fewer than 3 periods with positive claims							
Other	Daily living	Enhanced	SRTI	18-19	N	No model - fewer than 3 periods with positive claims							
Other	Daily living	Enhanced	SRTI	20-24	Y	No model - fewer than 3 periods with positive claims							
Other	Daily living	Enhanced	SRTI	20-24	N	No model - fewer than 3 periods with positive claims							
Other	Daily living	Enhanced	SRTI	25-29	Y	No model - fewer than 3 periods with positive claims							
Other	Daily living	Enhanced	SRTI	25-29	N	No model - fewer than 3 periods with positive claims							
Other	Daily living	Enhanced	SRTI	30-34	Y	No model - fewer than 3 periods with positive claims							
Other	Daily living	Enhanced	SRTI	30-34	N	No model - fewer than 3 periods with positive claims							
Other	Daily living	Enhanced	SRTI	35-39	Y	No model - fewer than 3 periods with positive claims							
Other	Daily living	Enhanced	SRTI	35-39	N	No model - fewer than 3 periods with positive claims							
Other	Daily living	Enhanced	SRTI	40-44	Y	No model - fewer than 3 periods with positive claims							
Other	Daily living	Enhanced	SRTI	40-44	N	No model - fewer than 3 periods with positive claims							
Other	Daily living	Enhanced	SRTI	45-49	Y	No model - fewer than 3 periods with positive claims							
Other	Daily living	Enhanced	SRTI	45-49	N	No model - fewer than 3 periods with positive claims							
Other	Daily living	Enhanced	SRTI	50-54	Y	No model - fewer than 3 periods with positive claims							
Other	Daily living	Enhanced	SRTI	50-54	N	No model - fewer than 3 periods with positive claims							
Other	Daily living	Enhanced	SRTI	55-59	Y	No model - fewer than 3 periods with positive claims							
Other	Daily living	Enhanced	SRTI	55-59	N	No model - fewer than 3 periods with positive claims							
Other	Daily living	Enhanced	SRTI	60-64	Y	No model - fewer than 3 periods with positive claims							
Other	Daily living	Enhanced	SRTI	60-64	N	No model - fewer than 3 periods with positive claims							

Continued on next page

Table 1 – continued from previous page

ICD-10 code	Component	Level	Rules	Ages	Female	Model type	A	s.e.	r	s.e.	k	s.e	RSE
Other	Daily living	Enhanced	SRTI	65-69	Y	No model - fewer than 3 periods with positive claims							
Other	Daily living	Enhanced	SRTI	65-69	N	No model - fewer than 3 periods with positive claims							
Other	Daily living	Enhanced	SRTI	70+	Y	No model - fewer than 3 periods with positive claims							
Other	Daily living	Enhanced	SRTI	70+	N	No model - fewer than 3 periods with positive claims							
Other	Daily living	Enhanced	non-SRTI	16-17	Y	No model - fewer than 3 periods with positive claims							
Other	Daily living	Enhanced	non-SRTI	16-17	N	No model - fewer than 3 periods with positive claims							
Other	Daily living	Enhanced	non-SRTI	18-19	Y	No model - fewer than 3 periods with positive claims							
Other	Daily living	Enhanced	non-SRTI	18-19	N	LM	9.03×10^{-4}	—	-0.178	0.012	17.278	0.906	0.063
Other	Daily living	Enhanced	non-SRTI	20-24	Y	No model - fewer than 3 periods with positive claims							
Other	Daily living	Enhanced	non-SRTI	20-24	N	No model - fewer than 3 periods with positive claims							
Other	Daily living	Enhanced	non-SRTI	25-29	Y	No model - fewer than 3 periods with positive claims							
Other	Daily living	Enhanced	non-SRTI	25-29	N	No model - fewer than 3 periods with positive claims							
Other	Daily living	Enhanced	non-SRTI	30-34	Y	No model - fewer than 3 periods with positive claims							
Other	Daily living	Enhanced	non-SRTI	30-34	N	No model - fewer than 3 periods with positive claims							
Other	Daily living	Enhanced	non-SRTI	35-39	Y	No model - fewer than 3 periods with positive claims							
Other	Daily living	Enhanced	non-SRTI	35-39	N	No model - fewer than 3 periods with positive claims							
Other	Daily living	Enhanced	non-SRTI	40-44	Y	No model - fewer than 3 periods with positive claims							
Other	Daily living	Enhanced	non-SRTI	40-44	N	No model - fewer than 3 periods with positive claims							
Other	Daily living	Enhanced	non-SRTI	45-49	Y	No model - fewer than 3 periods with positive claims							
Other	Daily living	Enhanced	non-SRTI	45-49	N	No model - fewer than 3 periods with positive claims							
Other	Daily living	Enhanced	non-SRTI	50-54	Y	No model - fewer than 3 periods with positive claims							
Other	Daily living	Enhanced	non-SRTI	50-54	N	No model - fewer than 3 periods with positive claims							
Other	Daily living	Enhanced	non-SRTI	55-59	Y	No model - fewer than 3 periods with positive claims							
Other	Daily living	Enhanced	non-SRTI	55-59	N	No model - fewer than 3 periods with positive claims							
Other	Daily living	Enhanced	non-SRTI	60-64	Y	No model - fewer than 3 periods with positive claims							
Other	Daily living	Enhanced	non-SRTI	60-64	N	No model - fewer than 3 periods with positive claims							
Other	Daily living	Enhanced	non-SRTI	65-69	Y	No model - fewer than 3 periods with positive claims							
Other	Daily living	Enhanced	non-SRTI	65-69	N	No model - fewer than 3 periods with positive claims							
Other	Daily living	Enhanced	non-SRTI	70+	Y	No model - fewer than 3 periods with positive claims							
Other	Daily living	Enhanced	non-SRTI	70+	N	No model - fewer than 3 periods with positive claims							
Other	Mobility	Enhanced	non-SRTI	16-17	Y	No model - fewer than 3 periods with positive claims							
Other	Mobility	Enhanced	non-SRTI	16-17	N	No model - fewer than 3 periods with positive claims							
Other	Mobility	Enhanced	non-SRTI	18-19	Y	No model - fewer than 3 periods with positive claims							
Other	Mobility	Enhanced	non-SRTI	18-19	N	No model - fewer than 3 periods with positive claims							
Other	Mobility	Enhanced	non-SRTI	20-24	Y	No model - fewer than 3 periods with positive claims							
Other	Mobility	Enhanced	non-SRTI	20-24	N	No model - fewer than 3 periods with positive claims							
Other	Mobility	Enhanced	non-SRTI	25-29	Y	No model - fewer than 3 periods with positive claims							
Other	Mobility	Enhanced	non-SRTI	25-29	N	No model - fewer than 3 periods with positive claims							
Other	Mobility	Enhanced	non-SRTI	30-34	Y	No model - fewer than 3 periods with positive claims							
Other	Mobility	Enhanced	non-SRTI	30-34	N	No model - fewer than 3 periods with positive claims							
Other	Mobility	Enhanced	non-SRTI	35-39	Y	No model - fewer than 3 periods with positive claims							
Other	Mobility	Enhanced	non-SRTI	35-39	N	No model - fewer than 3 periods with positive claims							
Other	Mobility	Enhanced	non-SRTI	40-44	Y	No model - fewer than 3 periods with positive claims							
Other	Mobility	Enhanced	non-SRTI	40-44	N	No model - fewer than 3 periods with positive claims							
Other	Mobility	Enhanced	non-SRTI	45-49	Y	No model - fewer than 3 periods with positive claims							
Other	Mobility	Enhanced	non-SRTI	45-49	N	No model - fewer than 3 periods with positive claims							
Other	Mobility	Enhanced	non-SRTI	50-54	Y	No model - fewer than 3 periods with positive claims							
Other	Mobility	Enhanced	non-SRTI	50-54	N	No model - fewer than 3 periods with positive claims							
Other	Mobility	Enhanced	non-SRTI	55-59	Y	No model - fewer than 3 periods with positive claims							
Other	Mobility	Enhanced	non-SRTI	55-59	N	No model - fewer than 3 periods with positive claims							
Other	Mobility	Enhanced	non-SRTI	60-64	Y	No model - fewer than 3 periods with positive claims							
Other	Mobility	Enhanced	non-SRTI	60-64	N	No model - fewer than 3 periods with positive claims							
Other	Mobility	Enhanced	non-SRTI	65-69	Y	No model - fewer than 3 periods with positive claims							
Other	Mobility	Enhanced	non-SRTI	65-69	N	No model - fewer than 3 periods with positive claims							
Other	Mobility	Enhanced	non-SRTI	70+	Y	No model - fewer than 3 periods with positive claims							
Other	Mobility	Enhanced	non-SRTI	70+	N	No model - fewer than 3 periods with positive claims							
Other	Daily living	Standard	SRTI	16-17	Y	No model - fewer than 3 periods with positive claims							

Continued on next page

Table 1 – continued from previous page

ICD-10 code	Component	Level	Rules	Ages	Female	Model type	A	s.e.	r	s.e.	k	s.e	RSE
Other	Daily living	Standard	SRTI	16-17	N	No model - fewer than 3 periods with positive claims							
Other	Daily living	Standard	SRTI	18-19	Y	No model - fewer than 3 periods with positive claims							
Other	Daily living	Standard	SRTI	18-19	N	No model - fewer than 3 periods with positive claims							
Other	Daily living	Standard	SRTI	20-24	Y	No model - fewer than 3 periods with positive claims							
Other	Daily living	Standard	SRTI	20-24	N	No model - fewer than 3 periods with positive claims							
Other	Daily living	Standard	SRTI	25-29	Y	No model - fewer than 3 periods with positive claims							
Other	Daily living	Standard	SRTI	25-29	N	No model - fewer than 3 periods with positive claims							
Other	Daily living	Standard	SRTI	30-34	Y	No model - fewer than 3 periods with positive claims							
Other	Daily living	Standard	SRTI	30-34	N	No model - fewer than 3 periods with positive claims							
Other	Daily living	Standard	SRTI	35-39	Y	No model - fewer than 3 periods with positive claims							
Other	Daily living	Standard	SRTI	35-39	N	No model - fewer than 3 periods with positive claims							
Other	Daily living	Standard	SRTI	40-44	Y	No model - fewer than 3 periods with positive claims							
Other	Daily living	Standard	SRTI	40-44	N	No model - fewer than 3 periods with positive claims							
Other	Daily living	Standard	SRTI	45-49	Y	No model - fewer than 3 periods with positive claims							
Other	Daily living	Standard	SRTI	45-49	N	No model - fewer than 3 periods with positive claims							
Other	Daily living	Standard	SRTI	50-54	Y	No model - fewer than 3 periods with positive claims							
Other	Daily living	Standard	SRTI	50-54	N	No model - fewer than 3 periods with positive claims							
Other	Daily living	Standard	SRTI	55-59	Y	No model - fewer than 3 periods with positive claims							
Other	Daily living	Standard	SRTI	55-59	N	No model - fewer than 3 periods with positive claims							
Other	Daily living	Standard	SRTI	60-64	Y	No model - fewer than 3 periods with positive claims							
Other	Daily living	Standard	SRTI	60-64	N	No model - fewer than 3 periods with positive claims							
Other	Daily living	Standard	SRTI	65-69	Y	No model - fewer than 3 periods with positive claims							
Other	Daily living	Standard	SRTI	65-69	N	No model - fewer than 3 periods with positive claims							
Other	Daily living	Standard	SRTI	70+	Y	No model - fewer than 3 periods with positive claims							
Other	Daily living	Standard	SRTI	70+	N	No model - fewer than 3 periods with positive claims							
Other	Daily living	Standard	non-SRTI	16-17	Y	No model - fewer than 3 periods with positive claims							
Other	Daily living	Standard	non-SRTI	16-17	N	No model - fewer than 3 periods with positive claims							
Other	Daily living	Standard	non-SRTI	18-19	Y	No model - fewer than 3 periods with positive claims							
Other	Daily living	Standard	non-SRTI	18-19	N	No model - fewer than 3 periods with positive claims							
Other	Daily living	Standard	non-SRTI	20-24	Y	No model - fewer than 3 periods with positive claims							
Other	Daily living	Standard	non-SRTI	20-24	N	No model - fewer than 3 periods with positive claims							
Other	Daily living	Standard	non-SRTI	25-29	Y	No model - fewer than 3 periods with positive claims							
Other	Daily living	Standard	non-SRTI	25-29	N	No model - fewer than 3 periods with positive claims							
Other	Daily living	Standard	non-SRTI	30-34	Y	No model - fewer than 3 periods with positive claims							
Other	Daily living	Standard	non-SRTI	30-34	N	No model - fewer than 3 periods with positive claims							
Other	Daily living	Standard	non-SRTI	35-39	Y	No model - fewer than 3 periods with positive claims							
Other	Daily living	Standard	non-SRTI	35-39	N	No model - fewer than 3 periods with positive claims							
Other	Daily living	Standard	non-SRTI	40-44	Y	No model - fewer than 3 periods with positive claims							
Other	Daily living	Standard	non-SRTI	40-44	N	No model - fewer than 3 periods with positive claims							
Other	Daily living	Standard	non-SRTI	45-49	Y	No model - fewer than 3 periods with positive claims							
Other	Daily living	Standard	non-SRTI	45-49	N	No model - fewer than 3 periods with positive claims							
Other	Daily living	Standard	non-SRTI	50-54	Y	No model - fewer than 3 periods with positive claims							
Other	Daily living	Standard	non-SRTI	50-54	N	No model - fewer than 3 periods with positive claims							
Other	Daily living	Standard	non-SRTI	55-59	Y	No model - fewer than 3 periods with positive claims							
Other	Daily living	Standard	non-SRTI	55-59	N	No model - fewer than 3 periods with positive claims							
Other	Daily living	Standard	non-SRTI	60-64	Y	No model - fewer than 3 periods with positive claims							
Other	Daily living	Standard	non-SRTI	60-64	N	No model - fewer than 3 periods with positive claims							
Other	Daily living	Standard	non-SRTI	65-69	Y	No model - fewer than 3 periods with positive claims							
Other	Daily living	Standard	non-SRTI	65-69	N	No model - fewer than 3 periods with positive claims							
Other	Daily living	Standard	non-SRTI	70+	Y	No model - fewer than 3 periods with positive claims							
Other	Daily living	Standard	non-SRTI	70+	N	No model - fewer than 3 periods with positive claims							
Other	Mobility	Standard	non-SRTI	16-17	Y	No model - fewer than 3 periods with positive claims							
Other	Mobility	Standard	non-SRTI	16-17	N	No model - fewer than 3 periods with positive claims							
Other	Mobility	Standard	non-SRTI	18-19	Y	No model - fewer than 3 periods with positive claims							
Other	Mobility	Standard	non-SRTI	18-19	N	No model - fewer than 3 periods with positive claims							
Other	Mobility	Standard	non-SRTI	20-24	Y	No model - fewer than 3 periods with positive claims							
Other	Mobility	Standard	non-SRTI	20-24	N	No model - fewer than 3 periods with positive claims							

Continued on next page

Table 1 – continued from previous page

ICD-10 code	Component	Level	Rules	Ages	Female	Model type	A	s.e.	r	s.e.	k	s.e	RSE
Other	Mobility	Standard	non-SRTI	25-29	Y	No model - fewer than 3 periods with positive claims							
Other	Mobility	Standard	non-SRTI	25-29	N	No model - fewer than 3 periods with positive claims							
Other	Mobility	Standard	non-SRTI	30-34	Y	No model - fewer than 3 periods with positive claims							
Other	Mobility	Standard	non-SRTI	30-34	N	No model - fewer than 3 periods with positive claims							
Other	Mobility	Standard	non-SRTI	35-39	Y	No model - fewer than 3 periods with positive claims							
Other	Mobility	Standard	non-SRTI	35-39	N	No model - fewer than 3 periods with positive claims							
Other	Mobility	Standard	non-SRTI	40-44	Y	No model - fewer than 3 periods with positive claims							
Other	Mobility	Standard	non-SRTI	40-44	N	No model - fewer than 3 periods with positive claims							
Other	Mobility	Standard	non-SRTI	45-49	Y	No model - fewer than 3 periods with positive claims							
Other	Mobility	Standard	non-SRTI	45-49	N	No model - fewer than 3 periods with positive claims							
Other	Mobility	Standard	non-SRTI	50-54	Y	No model - fewer than 3 periods with positive claims							
Other	Mobility	Standard	non-SRTI	50-54	N	No model - fewer than 3 periods with positive claims							
Other	Mobility	Standard	non-SRTI	55-59	Y	No model - fewer than 3 periods with positive claims							
Other	Mobility	Standard	non-SRTI	55-59	N	No model - fewer than 3 periods with positive claims							
Other	Mobility	Standard	non-SRTI	60-64	Y	No model - fewer than 3 periods with positive claims							
Other	Mobility	Standard	non-SRTI	60-64	N	No model - fewer than 3 periods with positive claims							
Other	Mobility	Standard	non-SRTI	65-69	Y	No model - fewer than 3 periods with positive claims							
Other	Mobility	Standard	non-SRTI	65-69	N	No model - fewer than 3 periods with positive claims							
Other	Mobility	Standard	non-SRTI	70+	Y	No model - fewer than 3 periods with positive claims							
Other	Mobility	Standard	non-SRTI	70+	N	No model - fewer than 3 periods with positive claims							

Note. SRTI = special rules for the terminally ill; LG = log growth model; LM = linear model; A = long-term fraction of people who claim; r = growth rate; k = log growth model constant; s.e. = standard error; RSE = residual standard error; N = 82 for all models
