# Supplementary material for: Changing the eligibility criteria for welfare payments at the end of life – a budget impact analysis for England and Wales
Source: BMC Health Serv Res. 2021 May 4;21:421. doi: 10.1186/s12913-021-06390-8 (PMC8094542; doi:10.1186/s12913-021-06390-8)
Supplement: Supplementary file 2 — Additional file 2: Table A 1. Estimated future personal independence payment expenditure under different scenarios. [file 12913_2021_6390_MOESM2_ESM.docx]

Appendix

Table A 1: Estimated future personal independence payment expenditure under different scenarios

| Scenario | Rules | Component | Expenditure (£ millions) | | | | | |
| --- | --- | --- | --- | --- | --- | --- | --- | --- |
|  |  |  | 2020 | 2021 | 2022 | 2023 | 2024 | 2025 |
| Low increase | Non-SRTI | Daily Living Enhanced | 4637 | 4804 | 4957 | 5102 | 5250 | 5404 |
|  |  | Daily Living Standard | 3258 | 3331 | 3392 | 3445 | 3495 | 3548 |
|  |  | Mobility Enhanced | 3150 | 3299 | 3406 | 3490 | 3566 | 3641 |
|  |  | Mobility Standard | 748 | 771 | 791 | 809 | 826 | 845 |
|  | SRTI | Daily Living Enhanced | 182 | 319 | 453 | 553 | 620 | 659 |
|  |  | Mobility Enhanced | 127 | 222 | 314 | 383 | 427 | 454 |
| Medium increase | Non-SRTI | Daily Living Enhanced | 4634 | 4794 | 4935 | 5070 | 5208 | 5357 |
|  |  | Daily Living Standard | 3256 | 3324 | 3378 | 3423 | 3467 | 3516 |
|  |  | Mobility Enhanced | 3149 | 3292 | 3391 | 3467 | 3537 | 3609 |
|  |  | Mobility Standard | 747 | 768 | 785 | 800 | 815 | 833 |
|  | SRTI | Daily Living Enhanced | 206 | 424 | 672 | 878 | 1033 | 1133 |
|  |  | Mobility Enhanced | 145 | 297 | 467 | 610 | 712 | 779 |
| High increase | Non-SRTI | Daily Living Enhanced | 4633 | 4785 | 4917 | 5042 | 5171 | 5313 |
|  |  | Daily Living Standard | 3255 | 3319 | 3365 | 3404 | 3442 | 3486 |
|  |  | Mobility Enhanced | 3147 | 3286 | 3378 | 3447 | 3510 | 3578 |
|  |  | Mobility Standard | 747 | 766 | 780 | 793 | 806 | 821 |
|  | SRTI | Daily Living Enhanced | 225 | 512 | 862 | 1167 | 1416 | 1587 |
|  |  | Mobility Enhanced | 160 | 360 | 599 | 813 | 978 | 1091 |
| Low increase, fast growth | Non-SRTI | Daily Living Enhanced | 4631 | 4788 | 4941 | 5092 | 5244 | 5400 |
|  |  | Daily Living Standard | 3254 | 3320 | 3382 | 3438 | 3491 | 3545 |
|  |  | Mobility Enhanced | 3146 | 3288 | 3395 | 3483 | 3561 | 3638 |
|  |  | Mobility Standard | 747 | 767 | 787 | 806 | 825 | 844 |
|  | SRTI | Daily Living Enhanced | 242 | 486 | 614 | 659 | 681 | 701 |
|  |  | Mobility Enhanced | 169 | 337 | 423 | 454 | 470 | 484 |
| Low increase, very fast growth | Non-SRTI | Daily Living Enhanced | 4616 | 4772 | 4935 | 5087 | 5240 | 5397 |
|  |  | Daily Living Standard | 3244 | 3310 | 3377 | 3435 | 3488 | 3543 |
|  |  | Mobility Enhanced | 3136 | 3278 | 3391 | 3479 | 3558 | 3636 |
|  |  | Mobility Standard | 743 | 763 | 785 | 805 | 824 | 843 |
|  | SRTI | Daily Living Enhanced | 392 | 638 | 677 | 703 | 719 | 732 |
|  |  | Mobility Enhanced | 272 | 440 | 468 | 486 | 497 | 507 |
| Medium increase, fast growth | Non-SRTI | Daily Living Enhanced | 4625 | 4762 | 4898 | 5042 | 5192 | 5347 |
|  |  | Daily Living Standard | 3250 | 3303 | 3353 | 3404 | 3456 | 3509 |
|  |  | Mobility Enhanced | 3142 | 3270 | 3366 | 3449 | 3526 | 3601 |
|  |  | Mobility Standard | 745 | 760 | 776 | 793 | 811 | 830 |
|  | SRTI | Daily Living Enhanced | 302 | 747 | 1040 | 1153 | 1198 | 1234 |
|  |  | Mobility Enhanced | 212 | 519 | 717 | 794 | 826 | 852 |
| Medium increase, very fast growth | Non-SRTI | Daily Living Enhanced | 4596 | 4725 | 4883 | 5033 | 5184 | 5339 |
|  |  | Daily Living Standard | 3231 | 3278 | 3343 | 3398 | 3450 | 3505 |
|  |  | Mobility Enhanced | 3122 | 3245 | 3355 | 3442 | 3519 | 3596 |
|  |  | Mobility Standard | 737 | 750 | 771 | 790 | 809 | 828 |
|  | SRTI | Daily Living Enhanced | 586 | 1115 | 1196 | 1249 | 1283 | 1308 |
|  |  | Mobility Enhanced | 407 | 768 | 826 | 863 | 887 | 905 |
| High increase, fast growth | Non-SRTI | Daily Living Enhanced | 4620 | 4739 | 4859 | 4995 | 5143 | 5296 |
|  |  | Daily Living Standard | 3246 | 3287 | 3326 | 3372 | 3422 | 3474 |
|  |  | Mobility Enhanced | 3138 | 3254 | 3338 | 3415 | 3490 | 3565 |
|  |  | Mobility Standard | 744 | 754 | 765 | 781 | 798 | 816 |
|  | SRTI | Daily Living Enhanced | 352 | 976 | 1441 | 1639 | 1710 | 1762 |
|  |  | Mobility Enhanced | 249 | 679 | 993 | 1128 | 1180 | 1217 |
| High increase, very fast growth | Non-SRTI | Daily Living Enhanced | 4580 | 4679 | 4833 | 4981 | 5130 | 5284 |
|  |  | Daily Living Standard | 3219 | 3247 | 3308 | 3362 | 3413 | 3466 |
|  |  | Mobility Enhanced | 3110 | 3213 | 3319 | 3404 | 3481 | 3556 |
|  |  | Mobility Standard | 733 | 738 | 758 | 777 | 795 | 813 |
|  | SRTI | Daily Living Enhanced | 758 | 1583 | 1710 | 1790 | 1844 | 1883 |
|  |  | Mobility Enhanced | 528 | 1090 | 1180 | 1238 | 1276 | 1303 |
| Low increase, low claims from non-SRTI | Non-SRTI | Daily Living Enhanced | 4641 | 4822 | 4988 | 5143 | 5297 | 5455 |
|  |  | Daily Living Standard | 3261 | 3343 | 3413 | 3472 | 3526 | 3582 |
|  |  | Mobility Enhanced | 3154 | 3312 | 3427 | 3518 | 3598 | 3676 |
|  |  | Mobility Standard | 749 | 776 | 799 | 819 | 839 | 858 |
|  | SRTI | Daily Living Enhanced | 182 | 319 | 453 | 553 | 620 | 659 |
|  |  | Mobility Enhanced | 127 | 222 | 314 | 383 | 427 | 454 |
| Low increase, medium claims from non-SRTI | Non-SRTI | Daily Living Enhanced | 4639 | 4813 | 4972 | 5123 | 5273 | 5429 |
|  |  | Daily Living Standard | 3259 | 3337 | 3403 | 3458 | 3511 | 3565 |
|  |  | Mobility Enhanced | 3152 | 3306 | 3417 | 3504 | 3582 | 3659 |
|  |  | Mobility Standard | 749 | 773 | 795 | 814 | 832 | 852 |
|  | SRTI | Daily Living Enhanced | 182 | 319 | 453 | 553 | 620 | 659 |
|  |  | Mobility Enhanced | 127 | 222 | 314 | 383 | 427 | 454 |
| Low increase, high claims from non-SRTI | Non-SRTI | Daily Living Enhanced | 4632 | 4786 | 4926 | 5062 | 5203 | 5354 |
|  |  | Daily Living Standard | 3255 | 3320 | 3372 | 3417 | 3464 | 3514 |
|  |  | Mobility Enhanced | 3147 | 3287 | 3384 | 3462 | 3533 | 3606 |
|  |  | Mobility Standard | 747 | 766 | 783 | 798 | 814 | 832 |
|  | SRTI | Daily Living Enhanced | 182 | 319 | 453 | 553 | 620 | 659 |
|  |  | Mobility Enhanced | 127 | 222 | 314 | 383 | 427 | 454 |
| Medium increase, low claims from non-SRTI | Non-SRTI | Daily Living Enhanced | 4641 | 4822 | 4988 | 5143 | 5297 | 5455 |
|  |  | Daily Living Standard | 3261 | 3343 | 3413 | 3472 | 3526 | 3582 |
|  |  | Mobility Enhanced | 3154 | 3312 | 3427 | 3518 | 3598 | 3676 |
|  |  | Mobility Standard | 749 | 776 | 799 | 819 | 839 | 858 |
|  | SRTI | Daily Living Enhanced | 206 | 424 | 672 | 878 | 1033 | 1133 |
|  |  | Mobility Enhanced | 145 | 297 | 467 | 610 | 712 | 779 |
| Medium increase, medium claims from non-SRTI | Non-SRTI | Daily Living Enhanced | 4638 | 4808 | 4961 | 5106 | 5253 | 5406 |
|  |  | Daily Living Standard | 3258 | 3334 | 3395 | 3447 | 3497 | 3549 |
|  |  | Mobility Enhanced | 3151 | 3302 | 3409 | 3492 | 3567 | 3642 |
|  |  | Mobility Standard | 748 | 772 | 792 | 810 | 827 | 845 |
|  | SRTI | Daily Living Enhanced | 206 | 424 | 672 | 878 | 1033 | 1133 |
|  |  | Mobility Enhanced | 145 | 297 | 467 | 610 | 712 | 779 |
| Medium increase, high claims from non-SRTI | Non-SRTI | Daily Living Enhanced | 4628 | 4766 | 4888 | 5005 | 5129 | 5270 |
|  |  | Daily Living Standard | 3252 | 3305 | 3342 | 3374 | 3408 | 3451 |
|  |  | Mobility Enhanced | 3144 | 3272 | 3354 | 3416 | 3476 | 3541 |
|  |  | Mobility Standard | 746 | 761 | 772 | 783 | 795 | 809 |
|  | SRTI | Daily Living Enhanced | 206 | 424 | 672 | 878 | 1033 | 1133 |
|  |  | Mobility Enhanced | 145 | 297 | 467 | 610 | 712 | 779 |
| High increase, low claims from non-SRTI | Non-SRTI | Daily Living Enhanced | 4641 | 4822 | 4988 | 5143 | 5297 | 5455 |
|  |  | Daily Living Standard | 3261 | 3343 | 3413 | 3472 | 3526 | 3582 |
|  |  | Mobility Enhanced | 3154 | 3312 | 3427 | 3518 | 3598 | 3676 |
|  |  | Mobility Standard | 749 | 776 | 799 | 819 | 839 | 858 |
|  | SRTI | Daily Living Enhanced | 225 | 512 | 862 | 1167 | 1416 | 1587 |
|  |  | Mobility Enhanced | 160 | 360 | 599 | 813 | 978 | 1091 |
| High increase, medium claims from non-SRTI | Non-SRTI | Daily Living Enhanced | 4637 | 4804 | 4952 | 5092 | 5233 | 5383 |
|  |  | Daily Living Standard | 3258 | 3331 | 3389 | 3438 | 3484 | 3534 |
|  |  | Mobility Enhanced | 3150 | 3299 | 3402 | 3482 | 3554 | 3627 |
|  |  | Mobility Standard | 748 | 771 | 789 | 806 | 822 | 839 |
|  | SRTI | Daily Living Enhanced | 225 | 512 | 862 | 1167 | 1416 | 1587 |
|  |  | Mobility Enhanced | 160 | 360 | 599 | 813 | 978 | 1091 |
| High increase, high claims from non-SRTI | Non-SRTI | Daily Living Enhanced | 4624 | 4751 | 4863 | 4966 | 5078 | 5211 |
|  |  | Daily Living Standard | 3249 | 3294 | 3318 | 3337 | 3359 | 3392 |
|  |  | Mobility Enhanced | 3141 | 3261 | 3332 | 3383 | 3432 | 3489 |
|  |  | Mobility Standard | 745 | 757 | 766 | 773 | 780 | 792 |
|  | SRTI | Daily Living Enhanced | 225 | 512 | 862 | 1167 | 1416 | 1587 |
|  |  | Mobility Enhanced | 160 | 360 | 599 | 813 | 978 | 1091 |
| Low increase, age 20 threshold | Non-SRTI | Daily Living Enhanced | 4629 | 4795 | 4947 | 5092 | 5239 | 5393 |
|  |  | Daily Living Standard | 3252 | 3325 | 3386 | 3438 | 3488 | 3541 |
|  |  | Mobility Enhanced | 3145 | 3293 | 3399 | 3482 | 3558 | 3633 |
|  |  | Mobility Standard | 746 | 768 | 788 | 806 | 824 | 842 |
|  | SRTI | Daily Living Enhanced | 266 | 417 | 557 | 660 | 728 | 770 |
|  |  | Mobility Enhanced | 186 | 290 | 387 | 457 | 503 | 532 |
| Low increase, age 30 threshold | Non-SRTI | Daily Living Enhanced | 4633 | 4800 | 4952 | 5097 | 5244 | 5398 |
|  |  | Daily Living Standard | 3255 | 3328 | 3389 | 3441 | 3491 | 3544 |
|  |  | Mobility Enhanced | 3148 | 3296 | 3402 | 3486 | 3562 | 3637 |
|  |  | Mobility Standard | 747 | 770 | 789 | 807 | 825 | 843 |
|  | SRTI | Daily Living Enhanced | 222 | 367 | 506 | 608 | 676 | 717 |
|  |  | Mobility Enhanced | 155 | 255 | 351 | 421 | 466 | 494 |
| Low increase, age 50 threshold | Non-SRTI | Daily Living Enhanced | 4637 | 4806 | 4961 | 5108 | 5257 | 5412 |
|  |  | Daily Living Standard | 3258 | 3333 | 3395 | 3448 | 3500 | 3553 |
|  |  | Mobility Enhanced | 3151 | 3301 | 3408 | 3493 | 3570 | 3646 |
|  |  | Mobility Standard | 748 | 771 | 792 | 810 | 828 | 847 |
|  | SRTI | Daily Living Enhanced | 177 | 301 | 416 | 497 | 550 | 581 |
|  |  | Mobility Enhanced | 123 | 210 | 291 | 348 | 383 | 404 |
| Medium increase, age 20 threshold | Non-SRTI | Daily Living Enhanced | 4620 | 4776 | 4916 | 5051 | 5189 | 5337 |
|  |  | Daily Living Standard | 3246 | 3312 | 3365 | 3410 | 3454 | 3503 |
|  |  | Mobility Enhanced | 3138 | 3279 | 3377 | 3453 | 3523 | 3594 |
|  |  | Mobility Standard | 744 | 764 | 780 | 795 | 810 | 827 |
|  | SRTI | Daily Living Enhanced | 372 | 619 | 877 | 1089 | 1249 | 1354 |
|  |  | Mobility Enhanced | 261 | 433 | 610 | 757 | 863 | 933 |
| Medium increase, age 30 threshold | Non-SRTI | Daily Living Enhanced | 4627 | 4785 | 4926 | 5060 | 5198 | 5346 |
|  |  | Daily Living Standard | 3251 | 3318 | 3371 | 3416 | 3460 | 3509 |
|  |  | Mobility Enhanced | 3143 | 3286 | 3384 | 3460 | 3529 | 3601 |
|  |  | Mobility Standard | 745 | 766 | 782 | 797 | 813 | 830 |
|  | SRTI | Daily Living Enhanced | 284 | 518 | 775 | 986 | 1145 | 1247 |
|  |  | Mobility Enhanced | 200 | 362 | 539 | 685 | 790 | 859 |
| Medium increase, age 50 threshold | Non-SRTI | Daily Living Enhanced | 4620 | 4776 | 4916 | 5051 | 5189 | 5337 |
|  |  | Daily Living Standard | 3246 | 3312 | 3365 | 3410 | 3454 | 3503 |
|  |  | Mobility Enhanced | 3138 | 3279 | 3377 | 3453 | 3523 | 3594 |
|  |  | Mobility Standard | 744 | 764 | 780 | 795 | 810 | 827 |
|  | SRTI | Daily Living Enhanced | 372 | 619 | 877 | 1089 | 1249 | 1354 |
|  |  | Mobility Enhanced | 261 | 433 | 610 | 757 | 863 | 933 |
| High increase, age 20 threshold | Non-SRTI | Daily Living Enhanced | 4612 | 4761 | 4891 | 5015 | 5144 | 5284 |
|  |  | Daily Living Standard | 3241 | 3302 | 3347 | 3385 | 3422 | 3466 |
|  |  | Mobility Enhanced | 3132 | 3268 | 3359 | 3427 | 3490 | 3557 |
|  |  | Mobility Standard | 742 | 760 | 773 | 786 | 798 | 814 |
|  | SRTI | Daily Living Enhanced | 474 | 802 | 1169 | 1484 | 1739 | 1916 |
|  |  | Mobility Enhanced | 334 | 563 | 813 | 1034 | 1204 | 1321 |
| High increase, age 30 threshold | Non-SRTI | Daily Living Enhanced | 4622 | 4773 | 4903 | 5028 | 5157 | 5298 |
|  |  | Daily Living Standard | 3247 | 3310 | 3355 | 3393 | 3431 | 3475 |
|  |  | Mobility Enhanced | 3140 | 3276 | 3367 | 3436 | 3499 | 3566 |
|  |  | Mobility Standard | 744 | 763 | 777 | 789 | 802 | 817 |
|  | SRTI | Daily Living Enhanced | 342 | 651 | 1015 | 1329 | 1582 | 1757 |
|  |  | Mobility Enhanced | 242 | 457 | 706 | 926 | 1095 | 1210 |
| High increase, age 50 threshold | Non-SRTI | Daily Living Enhanced | 4634 | 4788 | 4925 | 5055 | 5189 | 5334 |
|  |  | Daily Living Standard | 3256 | 3321 | 3370 | 3412 | 3454 | 3500 |
|  |  | Mobility Enhanced | 3148 | 3288 | 3382 | 3455 | 3521 | 3591 |
|  |  | Mobility Standard | 747 | 767 | 782 | 796 | 810 | 826 |
|  | SRTI | Daily Living Enhanced | 213 | 478 | 784 | 1037 | 1237 | 1371 |
|  |  | Daily Living Standard | 151 | 338 | 550 | 733 | 869 | 955 |
